# Asymmetric Hydride Shift Reactions Catalyzed by Chiral Aluminium Complexes

**DOI:** 10.1002/anie.202521374

**Published:** 2025-12-18

**Authors:** Mostafa M. Amer, Jingyan Hou, Jinfang Wang, Akvile Mazeikaite, Timothy J. Donohoe

**Affiliations:** ^1^ Department of Chemistry University of Oxford, Chemistry Research Laboratory Mansfield Road Oxford OX1 3TA UK

**Keywords:** Aldol, Aluminium, Asymmetric synthesis, Cyclohexenes, Hydride shift

## Abstract

An asymmetric intramolecular hydride shift reaction has been developed that is catalyzed by Al Lewis acids in conjunction with a chiral BINOL‐derived ligand. Racemic THP substrates are transformed into cyclohexene products via a prochiral intermediate ring opened enone; which then undergoes a key 1,5‐hydride shift reaction. This reaction is operationally simple, works well on a gram scale, and the desired products are formed with very high enantioselectivity (up to >98:2 e.r.). Importantly, the cyclohexene products contain functionality that can be easily derivatized and this is exemplified in the paper. Finally, a model is presented for the enantioselective hydride shift that is based on previous DFT studies.

## Graphical abstract

An asymmetric intramolecular hydride shift reaction has been developed that is catalyzed by Al Lewis acids in conjunction with a chiral BINOL‐derived ligand. Racemic THP substrates are transformed into cyclohexene products via an intermediate prochiral enone; which undergoes a key 1,5‐hydride shift reaction. This reaction works well on a gram scale, the desired products are formed with very high enantioselectivity (up to >98:2 e.r.) and the chiral ligand is easy to recover. Importantly, the cyclohexene products contain functionality that can be easily derivatized as exemplified in the paper.

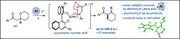

We recently reported an aluminium promoted intramolecular 1,5‐hydride shift/aldol cascade reaction that transformed substituted tetrahydropyran (THP) substrates **1** into heavily functionalized and synthetically useful cyclohexenes **2** (for an outline of the mechanism see **A**→**B**, Figure 1a).^[^ ^1^ ^]^ The development of an asymmetric version of this sequence is a key direction that we wished to follow in order to enhance and expand this methodology. In this regard, we chose to continue using Al as the promoting metal because of its effectiveness, low cost and the backdrop of exceptional work on the ability of this metal to promote and catalyze a wide range of organic transformations.

**Figure 1:**
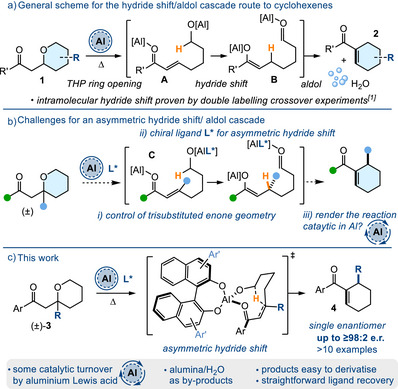
a) General scheme showing the hydride shift/aldol cascade. b) Challenges to overcome; c) This work: an asymmetric hydride shift followed by aldol ring closure. L* = chiral ligand.

However, there are some challenges to overcome if we want to render the intramolecular hydride shift^[^ ^2^, ^3^ ^]^ into an asymmetric one (see Figure 1b). For example, after THP ring opening the acceptor electron deficient alkene **C** must be trisubstituted and we will need to control its geometry during the reaction. Second, we must then utilise a chiral ligand (L*) that will coordinate to the aluminium metal promoter and impose an effective chiral environment onto the substrate during the hydride shift. Finally, we should attempt to lower the loadings of aluminium (and L*) and investigate catalytic Al transformations.

While the literature does contain examples of enantioselective aluminium catalyzed intermolecular hydride shifts (eg for a Meerwein Ponndorf Verley [MPV] carbonyl reduction^[^ ^4^, ^5^ ^]^) to the best of our knowledge setting a stereogenic centre by intramolecular hydride shift onto a trisubstituted enone, as proposed, is not known. We noted that the literature contains several examples of intramolecular asymmetric hydride shift reactions whereby the stereocontrol originates from a step (such as Mannich reaction) that occurs *after* an initiating hydride shift and not during the hydride shift as in this case.^[^ ^6^, ^7^, ^8^, ^9^, ^10^, ^11^, ^12^, ^13^, ^14^ ^]^

At the outset, we suspected that an equilibrium between the ring closed THP precursors (eg **1**, **3**) and reactive open chain enones (eg **A**, **C**) prior to hydride shift^[^ ^15^ ^]^ would be a plausible way of interconverting the enone geometric isomers in situ and this factor may allow the most reactive enone isomer to engage and react with an aluminium Lewis acid. As will be described later, we proposed to render the aluminium promoter chiral by using a suitable bidentate ligand (Figure 1c).

As part of our work we initially developed several different and convenient synthetic routes to the requisite racemic THP substrates (±)‐**3** involving conjugate addition to acetylenic ketones or aldol reactions of ketones and these are detailed in the Supporting Information.

Our studies on asymmetric synthesis began with the transformation of **3a**→**4a** using chiral aluminium Lewis acids (Scheme 1). Inspired by the work of Nguyen and Wulff on the use of BINOL and VANOL ligands for MPV reactions^[^ ^4^, ^5^ ^]^ we chose this subset of ligands for further investigation. Therefore, we screened a wide range of chiral bidentate ligands for aluminium and quickly discovered that the substituted BINOL system was the best framework, especially when using appended anthracenyl groups to introduce steric bulk at the *ortho* positions (see commercially available **L1** Scheme 1, Supporting Information and vide infra for more details). At this early stage we utilized stoichiometric aluminium and bidentate chiral ligand **L1**, together with one equivalent of the hydride transfer inactive *t*BuOH to occupy a third coordination site on the Al. The chiral aluminium complex was conveniently prepared by reaction of trimethylaluminium with the requisite alcohol ligands prior to addition of the substrate and subsequent heating. Pleasingly we were able to get reasonable yields and enantiomeric ratios (e.r.) for the formation of **4a**, but the highest e.r. that we could obtain under any set of conditions was 77:23 (Scheme 1). Changing the aryl group to the electron deficient one shown in **4b** made the overall reaction significantly faster, but again the e.r. was capped at 84:16. Subsequently we decided to increase the bulk of the R group attached to the THP starting material and moved to substrate **3c** (R = Bu). Pleasingly, this change had the desired effect and product **4c** was formed with an excellent 96:4 e.r. Changing the aryl group to make it electron rich (**4d**) or electron deficient (**4e**) retained the high levels of enantioselectivity. We also discovered that the introduction of a *t*Bu group onto the substrate gave equally high levels of selectivity in product **4f** (>96:4 e.r.). A significant breakthrough came when we switched the aryl group to the more robust Ph* motif **3** **g** (Ph* = C_6_Me_5_). Here, the yield of **4** **g** jumped to 72% with an excellent > 96:4 enantioselectivity. In this case the non‐planar nature of the aryl ketone means that the two *ortho* groups prevent nucleophilic attack at the C═O and this effect has been found to increase yields in a wide range of C─C bond forming reactions.^[^ ^16^, ^17^ ^]^ We also found that other di‐*ortho* substituted ketones (**3h**) behaved similarly. Finally, we changed the nature of the R group to include homoallyl (**4i**), *iso*‐propyl (**4j**) and protected hydroxyl groups (**4k**) and again formed products with a high level of enantioselectivity.

**Scheme 1:**
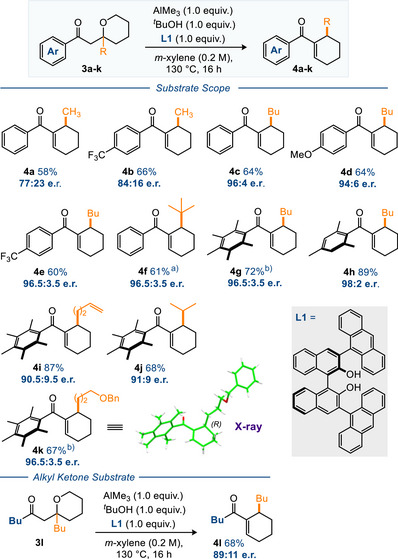
Substrate scope of the asymmetric hydride shift/aldol cascade. Yields shown are isolated material. Enantiomeric ratio (e.r.) determined by HPLC analysis using a chiral stationary phase. ^a)^ Substrate **3f** was isolated and used in the ring opened *E*‐enone form. ^b)^ The absolute stereochemistry of **4k** and a derivative of **4g** was assigned by X‐ray crystallography, vide infra; the absolute stereochemistry of the other cyclohexene products was assigned by analogy.

We were also able to gain an X‐ray crystal structure of **4k** and prove the absolute stereochemistry of this compound.^[^ ^18^, ^19^ ^]^ Interestingly, when we prepared and tested an alkyl ketone substrate **3l**, the result was only a moderate 89:11 er within product **4l**, Scheme 1. Given the much higher er ratio observed with the aromatic ketone counterparts of **4l** (ie **4c‐e** and **4g‐h**) we decided to pursue the reaction of ayl ketones further.

Our next goal was to investigate the ability of chiral Al Lewis acids to act as catalysts for the enantioselective cascade process.^[^ ^20^, ^21^, ^22^, ^23^ ^]^ However, we found that taking the reaction conditions shown in Scheme 1 and reducing the [Al] and ligand loadings gave lower conversions and irreproducible results (with respect to both yields and enantiomeric ratios), which we eventually attributed to the presence of two distinct alcohol ligands in the reaction (**L1** and *t*BuOH) which could form different species with the Al under the more demanding reaction conditions (see Supporting Information for more details). We solved this problem by omitting *t*BuOH completely and, taking the reaction of **3g**→**4g** as a model, we examined different catalytic loadings, chiral ligand ratios and times (Table 1). Using reduced loadings of Al and **L1** we found that the product **4g** was formed in good yields and e.r. after only 1.5 h (Entries 1–2). We also investigated the use of microwave irradiation instead of conventional heating, and found that comparable or slightly better results were obtained, again in 1.5 h (Entries 3–4). Therefore, for convenience we decided to optimize the microwave promoted reactions further. Reducing the ligand loadings from 80 mol% to 40 mol% showed that a ratio bidentate ligand to Al of 2:1 or 3:2 was optimal (Entries 3–5).

**Table 1:** Optimization of a catalytic enantioselective hydride shift.

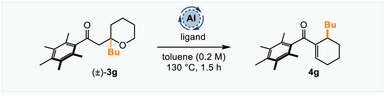					
Entry	Me_3_Al (mol%)	Ligand (mol%)	Yield **4g** (%)^a)^	Yield **3g** (%)	**4a** e.r.^b)^
1	40	**L1** (80)^c)^	76	–	97:3
2	40	**L1** (60)	72	–	94:6
3	40	**L1** (80)^d)^	82	–	97:3
4	40	**L1** (60)^d)^	74	–	97:3
5	40	**L1** (40)^d)^	76	11	88:12
6	20	**L1** (30)^d)^	40	55	96:4
7	20	**L1** (30)^d)^, ^e)^	62	31	88:12
8	40	**L2** (60)^d)^	72	4	93:7
9	40	**L3** (60)^d)^	38	50	80:20
10	40	**L4** (60)^d)^	52	10	67:33
11	40	**L5** (60)^d)^	45	50	63:37
12	40	**L6** (60)^d)^	64	–	70:30
13	40	**L1** (60)^d)^, ^f)^	75	13	98.5:1.5
14	40	**L2** (60)^d)^, ^f)^	74	–	93:7
15	40	**L1** (60)^d)^, ^g)^	56	30	87:13
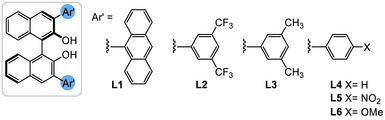					

Reaction conditions: Me_3_Al (2 M in toluene), ligand and **3g** in toluene (0.2 M) at 130 °C for 1.5 h.

^a)^ yield of isolated product.

^b)^ Determined by normal phase HPLC analysis using a chiral stationary phase.

^c)^ reaction time was 4 h.

^d)^ reaction performed using microwave irradiation.

^e)^ reaction time was 2.5 h.

^f)^ THF (9 equiv. relative to Me_3_Al) was used as an additive. RSM = recovered starting material **3g**.

^g)^ Only THF used as the solvent.

Keeping the lower 3:2 ratio constant, we then reduced the combined loadings further and found that the high enantiomeric ratio was maintained but that the yields started to fall (Entry 6); lengthening the reaction time did improve the yield but at the expense of enantiomeric ratios (Entry 7).

A screen of different aryl substituents on the BINOL backbone revealed that the sterically bulky anthracene gave the highest er, with the *meta*‐CF_3_ substituted **L2** being a good backup (Entries 8–12). Finally, we found that the addition of THF as an additive had a beneficial effect on the reaction (Entry 13):^[^ ^24^, ^25^, ^26^ ^]^ in general we found that adding small amounts of THF improved the er values slightly and gave higher overall mass recovery in the reaction. However, running the reaction in neat THF as solvent did not have a beneficial effect on the er (Entry 15).

Inspection of the data in Table 1 shows that the aluminium Lewis acid is capable of approximately two turnovers in this reaction. Our hypothesis is that the water by‐product produced from the aldol reaction may hydrolyze the chiral catalyst and lead to aluminium oxide species (such as alumina)^[^ ^27^ ^]^ which may themselves also catalyze the reaction^[^ ^1^ ^]^ (slowly) and with low to zero enantiomeric ratios (compare entries 6 & 7). In support of this we found that the addition of water (1 equiv.) to the reaction in entry 4 stopped the cascade sequence completely.

We then examined eight of the substrates from Scheme 1 using the optimized catalytic conditions in Entry 13 (Scheme 2). As before, the substrates with two *ortho*‐aryl substituents generally gave better yield and e.r. values than those without (compare **4c** and **4g**), although in all cases good levels of enantioselectivity were observed. Again, the addition of THF as an additive led to a better reaction on average, with moderately improved er value and better mass recoveries (within Scheme 2, the recovered starting material without THF ranged between 0–11% versus 11–27% with THF).

**Scheme 2:**
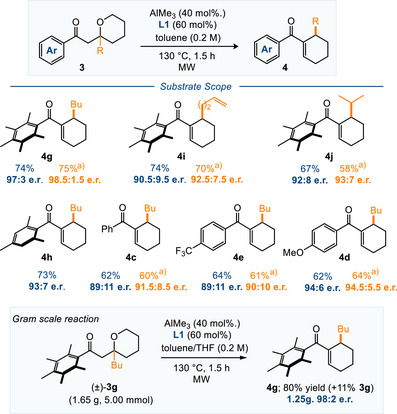
Substrate scope of the catalytic asymmetric hydride shift/aldol cascade. Yields shown are isolated material. Enantiomeric ratio (e.r.) determined by HPLC analysis using a chiral stationary phase. MW = microwave irradiation. ^a)^ THF (9 equiv. relative to Me_3_Al) used as an additive.

The only substrate that did not perform well under these conditions was **3k**, which furnished **4k** in excellent 97:3 e.r. but 35% yield (with another 35% recovered starting material, not shown). In this case we suspect that coordination of the substrate to the [Al] impedes this reaction and prevents turnover.

Pleasingly, when the reaction of **3g**→**4g** was carried out on a 5 mmol scale it performed very well, with 80% yield and 98:2 enantiomeric ratio, and furnishing 1.25 g of enantioenriched material (98:2 e.r.) for further elaboration (with 11% of recovered starting **3g** material adding to the excellent mass balance). In this reaction, 97% of the chiral ligand used was recovered easily by chromatography, and reuse of this ligand did not affect the yields or er values for subsequent experiments.

The mechanism of the asymmetric hydride shift reaction is proposed to proceed via an elimination reaction from THP **3** to give the ring opened enone **D**, followed by an intramolecular hydride shift (Scheme 3a). Previously, we have performed DFT studies on a simpler system lacking the R group sidechain and examined the role of enone geometry, conformation and aluminium aggregation state (monomers versus dimers) in facilitating the hydride shift reaction.^[^ ^1^ ^]^ Given the extreme steric bulk of the chiral ligands on Al, and their similarity to the powerful monomeric Al Lewis acids developed by Yamamoto (ie aluminium tris(2,6‐diphenylphenoxide), ATPH^[^ ^28^, ^29^, ^30^ ^]^) we assume that the reactive Al Lewis acid in this system is also monomeric.^[^ ^31^, ^32^ ^]^ In this case, reaction via the *Z*‐enone, in the *s‐cis* conformation (see **E** for an earlier DFT structure) is likely to be operative. Pleasingly, adaption of this original model by adding the chiral ligand then gives a clear explanation for the observed stereoselectivity (Scheme 3a).^[^ ^33^ ^]^ In the disfavored transition structure **F1** there is clear steric clash between the ligand Ar group (for **L1**, Ar = anthracenyl) and the substrate backbone (this unwanted steric interaction was first noted in the DFT studies for **E**).^[^ ^1^ ^]^ However, attack of hydride onto the opposite face of the alkene allows the reaction to proceed with a much reduced substrate/ligand interaction allowed by the approximately orthogonal orientation of the chiral ligand, **F2**. Models based on dimeric Al systems, with a bridging oxygen between the two metal atoms, are considered to be less likely, primarily because of the large steric crowding encountered between the substrate and the two requisite bulky chiral ligands (one on each Al centre). Moreover, our earlier DFT studies predicted that, for reaction via a dimeric Al species, the two lowest energy transition structures each contain an *E*‐configured alkene, which is opposite to that predicted for the monomeric Al pathway;^[^ ^1^ ^]^ and this leads to steric crowding in the open chain enone (eg for **3f**, which would then need to invert its enone geometry before reacting via a dimeric species). With this in mind, we also found that models based on a dimeric Al species to be much less clear cut with regards to which enantiomer of product they predicted

**Scheme 3:**
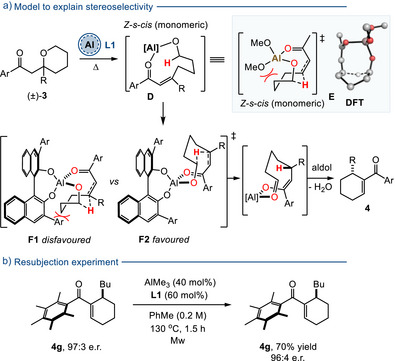
a) Model to explain the stereoselectivity imposed by ligand **L1**. In this model the R group is assumed to have the lowest CIP priority in the enone at the position β to the C═O. b) A resubjection experiment to rule out product racemization.

A control experiment whereby enantioenriched product **4g** was resubjected to the reaction conditions furnished recovered starting material with essentially the same e.r., thus showing that in situ racemization of the product was not occurring (eg over extended reaction times, Scheme 3b).

Finally, using compound **4g** produced on larger scale as essentially a single enantiomer we were able to derivatise the Ph* ketone to a variety of functional groups such as the carboxylic acid **5** and the amide **6** (Scheme 4). There are many possibilities for functionalisation of the cyclohexene core, as illustrated by the representative examples shown in Scheme 4. Treatment of **4g** with bromine effected an allylic bromination reaction to give **7** as a 70:30 mixture of diastereoisomers^[^ ^34^ ^]^ (an X‐ray crystal structure of the major isomer of **7** established both its relative [*trans*] and absolute (*S,S*) stereochemistry).^[^ ^18^, ^19^ ^]^ This mixture could then be functionalized further by a DMSO promoted oxidation to furnish enone **8**.^[^ ^35^ ^]^ Finally, extended enolate formation from **4g** was possible with LiHMDS base and quenching this enolate with excess BnI gave exclusive γ regioselectivity for product **9** (presumably this selectivity is driven by the large steric hinderance provided by the Ph* group). Compounds **5**, **8** and **9** were shown to retain very high levels of enantiomeric purity, proving that these derivatizations had proceeded without erosion of the er value.

**Scheme 4:**
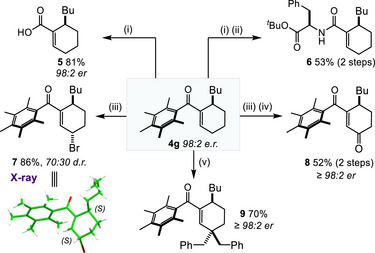
Derivatization of the cyclohexene products. (i) HCl, HFIP, 65 °C; (ii) H‐d‐Phe‐O*^t^* Bu·HCl, DMAP, EDCI, CH_2_Cl_2_, RT; (iii) Br_2_, CHCl_3_, RT; (iv) DMSO, NaHCO_3_, 80 °C; (v) LiHMDS (2.5 equiv.), BnI (4 equiv.), THF, RT. The enantiomeric ratio (e.r.) of **5** (as a benzyl ester derivative), **8** and **9** was determined by HPLC analysis using a chiral stationary phase.

In conclusion, we have utilised aluminium reagents in combination with a chiral BINOL‐derived ligand to impose absolute stereoselectivity on an intramolecular hydride shift reaction. Using this new mode of asymmetric induction, we have been able to prepare functionalized cyclohexenes with very high levels of enantioselectivity (>98:2 e.r. in some cases) and especially when using THF as an additive we were able to observe modest catalytic turnover. By modifying our original DFT calculations and introducing a chiral ligand, we have also been able to propose a model that rationalises the sense of stereochemistry in the cyclohexene products thus formed. Finally, the synthetic utility of these valuable building blocks has been validated with a set of representative derivatisation reactions which show great potential for the formation of useful synthetic intermediates.

## Conflict of Interests

The authors declare no conflict of interest.

## Supporting information



Supporting Information

Supporting Information

## Data Availability

The data that support the findings of this study are available in the supplementary material of this article.
