# Supplementary material for: Asymmetric Hydride Shift Reactions Catalyzed by Chiral Aluminium Complexes
Source: Angew Chem Int Ed Engl. 2025 Dec 18;65(5):e21374. doi: 10.1002/anie.202521374 (PMC12850996; doi:10.1002/anie.202521374)
Supplement: Supplementary file 1 — Supporting Information [file ANIE-65-e21374-s001.pdf]

Asymmetric hydride shift reactions catalysed by chiral aluminium complexes

Mostafa M. Amer,[a] Jingyan Hou,[a] Jinfang Wang,[a] Akvile Mazeikaite,[a¥]] and Timothy J. Donohoe*[a]

[a] Department of Chemistry, University of Oxford, Chemistry Research Laboratory, Mansfield Road, Oxford, OX1 3TA (UK)

E-mail: timothy.donohoe@chem.ox.ac.uk

Supporting Information

Contents

1. General Experimental 2

2. Optimisation 3

2.1 Index of ligands involved in the study 3

2.2 Representative example of irreproducibility in catalytic experiments with tBuOH additive..........3

2.3 Extended optimisation table 4

2.3.1 Screening of solvents, temperature, time on model substrate **3g**........ 4

2.3.2 Screening of different ligands on model substrate **3g** 5

2.3.3 Initial screening of different ligands on model substrate **3a**........ 5

3. General procedures and characterization data........ 6

3.1 Synthesis of THP precursors **3a-3f**........ 8

3.2 Synthesis of THP precursors **3g-3h**........ 14

3.3 Synthesis of THP precursors **3i-3j**........ 17

Synthesis of Binol ligand **L1** 28

Synthesis of cyclohexenes **4a-4k**........ 30

3.4 Synthesis of alkylketone substrate **3l** and cyclohexene **4l**........46

3. 5 Derivatization of Acyl Cyclohexenes........ 48

4. Crystallographic data for compounds **4k** and **7** 54

1. General Experimental

Reagents, solvents and techniques: All reactions were performed under an inert atmosphere with constant magnetic stirring, unless otherwise stated, using clean, oven dried glassware. Reagents and solvents were obtained from commercial supplies and were used without further purification. All inert gases were sourced from the University of Oxford's internal supplies and dried through $CaCl_2$ drying columns. Anhydrous solvents were obtained from the University of Oxford internal solvent system and dried by passing through alumina columns, manufactured by Innovative Technology Inc. PS-400-7. Temperatures of −78 °C and 0 °C were achieved with a CO_2(s)/acetone bath and ice/water bath respectively.

Thin layer chromatography was performed on Merck Kieselgel 60 F_{254} 0.25 mm pre-coated aluminium plates. Product spots were visualized under UV light (λ = 254 nm) and/or by staining with potassium permanganate solution, phosphomolybdic acid or vanillin. Flash chromatography was performed using VWR silica gel 60 (40-63 µm particle size) using head pressure by means of a nitrogen line.

NMR Spectroscopy: ^{1}H and ^{13}C NMR samples were run on a Bruker AVIIIHD 400 MHz spectrometer, where required COSY and HSQC spectra were used to assign NMR spectra. In addition, Bruker AVIIIHD 500 MHz and Bruker NEO 600 MHz spectrometers were used to obtain NOESY and HMBC data where required. All NMR spectra were recorded at ambient temperature. Chemical shifts are quoted in ppm with signal splittings recorded as singlet (s), doublet (d), triplet (t), quartet (q), quintet (qn), sextet (sext), septet (sept), and multiplet (m). The abbreviation br denotes broad.

Infrared spectra were recorded neat on a Bruker Tensor 27 spectrometer equipped with an attenuated total reflectance attachment with internal calibration. Absorption maxima (λ_{max}) are quoted in wavenumbers (cm^{-1}). The abbreviation br denotes broad.

Mass Spectroscopy: Mass spectra were obtained through the University of Oxford mass spectrometry service by electrospray ionisation (ESI) using a Bruker Daltonics microTOF spectrometer. The m/z values were all recorded in Daltons to four decimal places, the mass found was compared to the mass calculated from the monoisotopic molecular formula.

Optical rotations were recorded on a Bellingham+Stanley ADP450 polarimeter in a cell with a path length of 0.5 dm in the solvent specified, at temperature T, expressed in °C, at concentration c, expressed in g/100 mL.

Chiral normal phase HPLC was performed on an Agilent 1260 Series HPLC unit equipped with UV-vis diode-array detector, fitted with the appropriate Daicel Chiralpak column (dimensions: 0.46 cm ø x 25 cm) along with the corresponding guard column (0.4 cm ø x 1 cm). Wavelengths (λ) are reported in nm, retention times (t_R) are reported in minutes and solvent flow rates are reported in mL min^{-1}.

Melting Points: Melting points were obtained using Griffin melting point apparatus.

Microwave: Reactions in a microwave were completed on a Biotage® Initiator+.

2. Optimisation

2.1 Index of ligands involved in the study

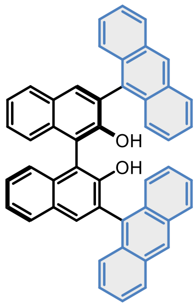

L1, CAS No: 361342-49-6

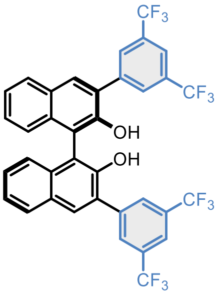

L2, CAS No: 756491-54-0

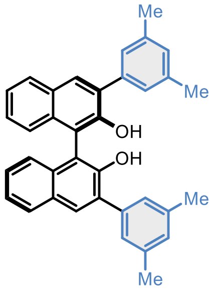

L3, CAS No: 215433-51-5

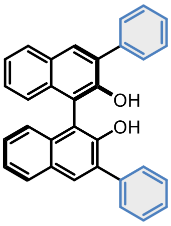

L4, CAS No: 39648-74-3

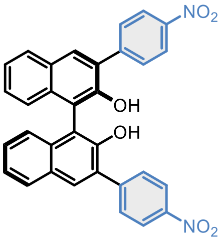

L5, CAS No: 791616-60-9

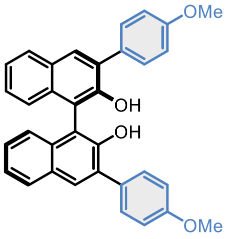

L6, CAS No: 756491-51-7

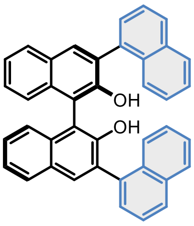

L7, CAS No: 851615-07-1

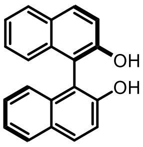

L8, CAS No: 18531-94-7

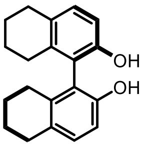

L9, CAS No: 65355-14-8

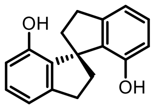

L10, CAS No: 223259-63-0

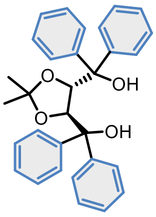

L11, CAS No: 93379-48-7

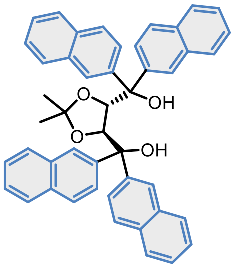

L12, CAS No: 137365-16-3

2.2 Representative example of irreproducibility in catalytic experiments with tBuOH additive.

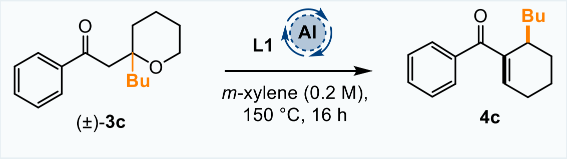

Run	Me_3Al (mol%)	Ligand (mol%)	*t*BuOH (mol%)	Yield (%)	e.r.
1	40	**L1** (40)	40	44	90:10
2	40	**L1** (40)	40	55	90:10
3	40	**L1** (40)	40	72	94:6

2.3 Extended optimisation table

2.3.1 Screening of solvents, temperature, time on model substrate 3g

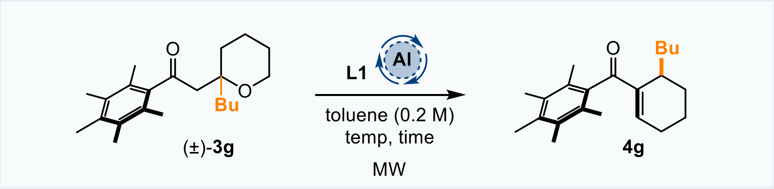

Entry	Me_3Al (mol%)	Ligand (mol%)	Time (h)	Temp (°C)	Yield (%)[a]	e.r.[b]
1	30	**L1** (45)	1.5	130	79	92:8
2	20	**L1** (40)	1.5	130	85	88:12
3	20	**L1** (30)	72	RT	-	-
4	20	**L1** (30)	2.5	90	25	95:5
5	20	**L1** (30)	1.5	110	19	94:6
6	20	**L1** (30)	1.5	130	70	84:16
7	20	**L1** (30), (0.4 M)	1.5	130	68	86:14
8	20	**L1** (30)	0.75	150	59	87:13
9	20	**L1** (30)	0.75	170	65	80:20
10[a]	20	**L1** (30)	1.5	130	40	96:4
11	10	**L1** (15)	1.5	130	19	85:15
12	20	**L1** (20)	1.5	130	68	79:21
13	20	**L1** (30) + 4A MS	1.5	130	27	81:19
14	20	**L1** (30) + 4A MS	3	130	61	85:15
15	20	**L1** (30), THF	1.5	130	56	87:13
16	20	**L1** (30), DCE	1.5	130	60	82:18
17	40	**L1** (60), DCE	1.5	130	74	86:14
18	40	**L1** (60), DCE	1.0	130	72	88:12
19	40	**L1** (60), DCE	1.0	150	78	80:20
20	40	**L1** (60), DCE	1.0	170	77	78:22
21	40	**L1** (60), DCE	0.5	190	69	79:21
22	20	**L1**,(30), THF (9.25 equiv.) [c]	1.5	130	51	92:8
23	20	**L1**,(30), THF (9.25 equiv.) [c]	1.5	130	54	90:10
24	40	L1,(60), THF (9.25 equiv.)[c]	1.5	130	75	98.5:1.5
25	40	L1,(60), THF (10 equiv.) [c]	1.5	130	75	98.3:1.7
26	20	**L2** (30)	1.5	130	70	84:16

[a] Yield of isolated product, [b] Determined by normal phase HPLC analysis using a chiral stationary phase. [c] compared to $AlMe_3$

2.3.2 Screening of different ligands on model substrate 3g

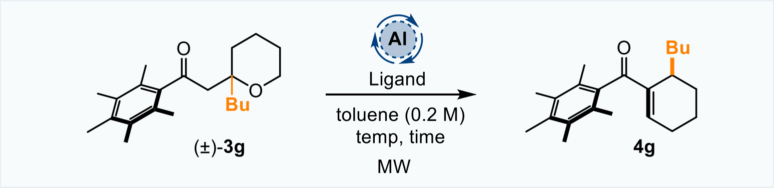

Entry	Me_3Al (mol%)	Ligand (mol%)	Time (h)	Temp (°C)	Yield (%)[a]	e.r.[c]
1	20	**L3** (30)	1.5	130	8	72:28
2	40	**L3** (60)	1.5	130	38	80:20
3	20	**L5** (30)	1.5	130	37	70:30
4	20	**L5** (30)	2.5	90	23	75:25
5	20	**L6** (30)	1.5	130	3	58:42
6	40	**L7** (60)	1.5	130	35	79:21
7	20	**L7** (30)	1.5	130	20	75:15
8	20	**L2** (20)	1.5	130	61	88:12
9	20	**L2** (20), ^{t}BuOH	1.5	130	50	85:15
8	20	**L2** (30), $Al(SO_3)_3$	1.5	130	50	80:20
9	20	**L2** (30)	1.5	130	48	88:12
10	20	**L2** (30), THF (9.25 equiv.)	1.5	130	45	92:8
11	40	**L2** (60)	1.5	130	72	90:10
12	40	**L2** (60), THF (9.25 equiv.)	1.5	130	74	93:7
13	40	**L2** (20)	1.5	130	60	87:13

2.3.3 Initial screening of different ligands on model substrate 3a

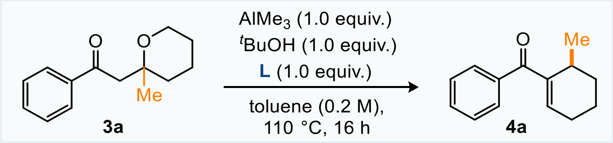

**Entry**	**Ligand**	**Yield (%)[a]**	**e.r.[c]**
1	**L8**	(12)	57:43
2	**L9**	(15)	52:48
3	**L10**	(20)	51:49
4	**L11**	(26)	56:44
5	**L12**	(15)	65:35

3. General procedures and characterization data

General Procedure 1 – Synthesis of Weinreb amides

Dimethylhydroxylamine hydrochloride (1.1 equiv.) was added to a solution of benzoyl chloride in CH_2Cl_2 (0.3 M) under N_2. The reaction mixture was cooled to 0 °C and triethylamine (3.0 equiv.) was added dropwise. The reaction was warmed to RT and stirred overnight. The mixture was quenched by the addition of sat. aq. ammonium chloride and extracted with CH_2Cl_2. The combined organic phases were dried over $MgSO_4$, filtered and concentrated *in vacuo*. For details of purification, see experimental procedures.

General Procedure 2 – Synthesis of ynones

n-BuLi in hexanes (2.4 equiv.) was added dropwise to a solution of 5-hexyn-1-ol (1.2 equiv.) in THF (0.3 M) at –78 °C under N_2. The reaction mixture was stirred for 1 h, and a solution of Weinreb amide (1.0 equiv.) in THF (3.0 M) was added dropwise. The reaction was warmed to RT and stirred for 1 h. The mixture was quenched by the addition of sat. aq. ammonium chloride and extracted with EtOAc. The combined organic phases were dried over $MgSO_4$, filtered and concentrated *in vacuo*. For details of purification, see the experimental procedures.

General Procedure 3: DMP oxidation

N-Protected diol (0.39 mmol, 1 equiv.) was dissolved in CH_2Cl_2 (3.5 mL). $NaHCO_3$ (197 mg, 2.34 mmol, 6 equiv.) and Dess-Martin Periodinane (250 mg, 0.59 mmol, 1.5 equiv.) were added, and the mixture left to stir for 30 min at RT. The reaction was diluted with saturated aqueous $NaHCO_3$ solution (5 mL) and saturated aqueous $Na_2S_2O_3$ solution, and extracted with CH_2Cl_2 (3 × 15 mL). The organic layer was dried with $MgSO_4$, filtered and the solvent removed *in vacuo.* For details of purification, see the experimental procedures.

General Procedure 4 – Synthesis of racemic cyclohexenes by aluminium promoted [1,5]-hydride shift

A 2–5 mL Biotage® microwave vial was charged with the tetrahydropyran (1.0 equiv.), basic alumina (Brockmann I, 5.0 equiv.) or $Al(O^tBu)_3$ (1.0 equiv.) and toluene (0.2 M). The vial was degassed, backfilled with N_2, sealed with a microwave vial cap containing a Reseal™ septum and heated at 110 °C for 16 h. The reaction was cooled to RT, diluted with Et_2O, filtered through a plug of silica gel, and concentrated *in vacuo*. For details of purification, see the experimental procedures.

General Procedure 5A – Asymmetric synthesis of cyclohexenes by aluminium promoted [1,5]-hydride shift

A 2–5 mL Biotage® microwave vial was charged with (*R*)-3,3'-di(anthracen-9-yl)-[1,1'-binaphthalene]-2,2'-diol (1.0 equiv.), *tert*-butanol (1.0 equiv.) and *m*-xylene (0.2 M). The vial was degassed, backfilled with N_2, and a solution of $AlMe_3$ in toluene (2.0 M, 1.0 equiv.) was added dropwise to the suspension. The mixture was stirred for 30 min, tetrahydropyran starting material (1.0 equiv.) was added, and the vial was sealed with a microwave vial cap containing a Reseal™ septum. The reaction was heated at 130 °C for 16 h, cooled to RT, diluted with Et_2O,

filtered through a plug of silica gel, and concentrated *in vacuo*. For details of purification, see the experimental procedures.

General Procedure 5B – Asymmetric synthesis of cyclohexenes by aluminium catalysed [1,5]-hydride shift

A 2–5 mL Biotage® microwave vial was charged with (*R*)-3,3'-di(anthracen-9-yl)-[1,1'-binaphthalene]-2,2'-diol (77 mg, 0.12 mmol, 0.6 equiv.) and toluene (0.2 M, 1.0 mL), and then degassed with argon. $AlMe_3$ in toluene (1.0 M, 0.08 mL, 0.08 mmol, 0.4 equiv.) was added dropwise to the suspension under N_2, and the mixture stirred for 30 min. Tetrahydropyran starting material (0.20 mmol, 1.0 equiv.) was then added, and the reaction vessel was sealed with a microwave vial cap (containing a Reseal™ septum) and heated at 130 °C for 1.5 h in the microwave. The reaction mixture was cooled to room temperature, diluted with Et_2O, filtered through a plug of silica gel, and concentrated in vacuo. For purification details, see the experimental procedures.

General Procedure 5C – Asymmetric synthesis of cyclohexenes by aluminium catalysed [1,5]-hydride shift with THF

A 2–5 mL Biotage® microwave vial was charged with (*R*)-3,3'-di(anthracen-9-yl)-[1,1'-binaphthalene]-2,2'-diol (77 mg, 0.12 mmol, 0.6 equiv.) and toluene (0.2 M, 1.0 mL), and then degassed with N_2. $AlMe_3$ in toluene (1.0 M, 0.08 mL, 0.08 mmol, 0.4 equiv.) was added dropwise to the suspension under N_2, after which dry THF (60 µL, 0.73 mmol, 9.25 equiv. compared to $AlMe_3$) was added and the mixture stirred for 30 min. Tetrahydropyran starting material (0.20 mmol, 1.0 equiv.) was then added, and the reaction vessel was sealed with a microwave vial cap (containing a Reseal™ septum) and heated at 130 °C for 1.5 h in the microwave. The reaction mixture was cooled to room temperature, diluted with Et_2O, filtered through a plug of silica gel, and concentrated in vacuo. For purification details, see the experimental procedures.

Note: In **General Procedure (5A-C)**, (*R*)-3,3'-di(anthracen-9-yl)-[1,1'-binaphthalene]-2,2'-diol (L1, CAS No: 361342-49-6) was recycled and recovered after column chromatography (Pentane:Et_2O, 60:40) as an off-white solid in (97-99% yield).

3.1 Synthesis of THP precursors 3a-3f

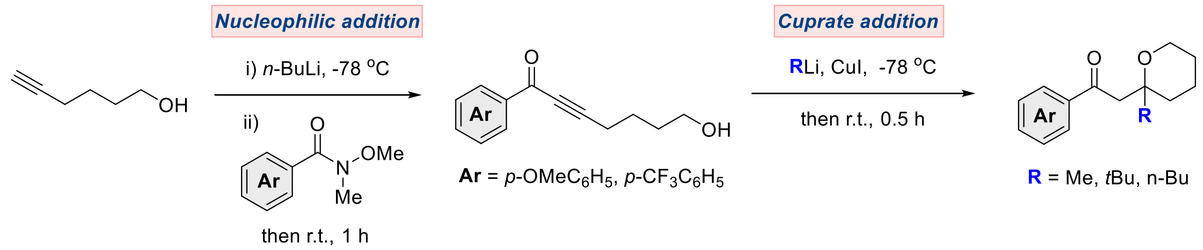

7-Hydroxy-1-phenylhept-2-yn-1-one, S1

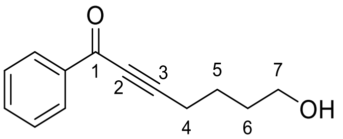

n-BuLi in hexanes (23.0 mL, 2.5 M, 58.0 mmol, 2.4 equiv.), 5-hexyn-1-ol (2.83 g, 28.8 mmol, 1.2 equiv.), THF (0.3 M, 80 mL), and *N*-methoxy-*N*-methylbenzamide (3.96 g, 24.0 mmol, 1.0 equiv.) were subjected to **General Procedure 2**. Purification by column chromatography (pentane:EtOAc, 80:20 to 60:40) afforded the title compound **S1** as a yellow oil (4.15 g, 85% yield).

^{1}H NMR (400 MHz, $CDCl_3$) δ 8.17 – 8.07 (m, 2H, ArCH x 2), 7.64 – 7.54 (m, 1H, ArCH), 7.52 – 7.41 (m, 2H, ArCH x 2), 3.78 – 3.65 (m, 2H, H-7 x 2), 2.61 – 2.49 (m, 2H, H-4 x 2), 1.85 – 1.69 (m, 4H, H-5 x 2, H-6 x 2), 1.62 (s, 1H, OH).

^{13}C NMR (101 MHz, $CDCl_3$) δ 178.4 (C-1), 137.0 (ArC), 134.1 (ArC), 129.7 (ArC x 2), 128.7(ArC x 2), 96.4 (C-3), 80.0 (C-2), 62.3 (C-7), 31.9 (C-6), 24.4 (C-5), 19.2 (C-4).

HRMS (ESI+): Found $[M+H]^+$ = 203.1071; $C_{13}H_{15}O_2$ requires 203. 1067 Δ 2.16 ppm

IR (film) v_{max}/cm^{-1}: 3389, 2938, 2869, 2236, 2200, 1639.

(±)-2-(2-Methyltetrahydro-2*H*-pyran-2-yl)-1-phenylethan-1-one, 3a

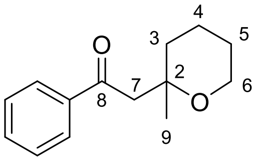

MeLi in Et_2O (1.6 M, 11.2 mL, 18.0 mmol, 6.0 equiv.) was added dropwise to a solution of CuI (1.72 g, 9.00 mmol, 3.0 equiv.) in THF (0.3 M, 25 mL) at –78 °C. The reaction mixture was warmed to 0 °C and stirred for 1 h, affording a colourless solution. The mixture was cooled to –78 °C, and a solution of **S1** (606 mg, 3.00 mmol, 1.0 equiv.) in THF (1.0 M, 3 mL) was added dropwise and stirred for 30 min. The reaction was quenched by addition of saturated aqueous ammonium chloride (30 mL) and warmed to RT. The mixture was treated with 35% aqueous ammonium hydroxide solution (30 mL), affording a clear blue aqueous phase. The solution was extracted with EtOAc (3 x 60 mL) and the combined organic phases were washed with HCl (1.0 M, 50 mL), dried over $MgSO_4$, filtered, and concentrated *in vacuo*. Purification by column chromatography (pentane:Et_2O, 95:5) afforded the title compound **3a** as a yellow oil (588 mg, 90% yield).

^{1}H NMR (400 MHz, $CDCl_3$) δ 8.02 – 7.94 (m, 2H, ArH x 2), 7.59 – 7.50 (m, 1H, ArH), 7.49 – 7.40 (m, 2H, ArH), 3.76 – 3.65 (m, 2H, H-6), 3.25 – 3.13 (m, 2H, H-7), 1.78 – 1.62 (m, 4H, H-3, H-4), 1.59 – 1.47 (m, 2H, H-5), 1.33 (s, 3H, H-9).

^{13}C NMR (101 MHz, $CDCl_3$) δ 199.2 (C-8), 138.5 (ArC), 133.0 (ArC), 128.6 (ArC x 4), 73.5 (C-2), 61.8 (C-6), 48.1 (C-7), 35.1 (C-3), 25.9 (C-5), 23.4 (C-9), 19.5 (C-4).

HRMS (ESI+): Found $[M+H]^+$ = 219.1373; $C_{14}H_{19}O_2$ requires 219.1380 Δ -3.01 ppm.

IR (film) v_{max}/cm^{-1}: 2980, 1676, 1597, 1449, 1378, 1232.

(±)-2-(2-Butyltetrahydro-*2H*-pyran-2-yl)-1-phenylethan-1-one, 3c

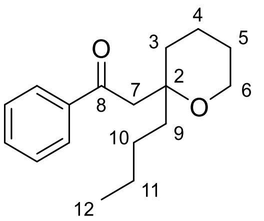

n-BuLi in hexanes (2.5 M, 1.2 mL, 3.0 mmol, 6.0 equiv.) was added dropwise to a solution of CuI (285 mg, 1.5 mmol, 3.0 equiv.) in THF (0.3 M, 5 mL) at –78 °C. The reaction mixture was warmed to 0 °C and stirred for 1 h, affording a black precipitate. The mixture was cooled to –78 °C, and a solution of **S1** (101 mg, 0.50 mmol, 1.0 equiv.) in THF (1.0 M, 0.5 mL) was added dropwise and stirred for 30 min. The reaction was quenched by addition of saturated aqueous ammonium chloride (10 mL) and warmed to RT. The mixture was treated with 35% aqueous ammonium hydroxide solution (10 mL), affording a clear blue aqueous phase. The solution was extracted with EtOAc (3 x 20 mL) and the combined organic phases were washed with HCl (1.0 M, 20 mL), dried over $MgSO_4$, filtered, and concentrated *in vacuo*. Purification by column chromatography (pentane:Et_2O, 95:5) afforded the title compound **3c** as a yellow oil (101 mg, 78% yield).

^{1}H NMR (400 MHz, $CDCl_3$) δ 8.00 – 7.93 (m, 2H, ArH), 7.58 – 7.50 (m, 1H, ArH), 7.49 – 7.40 (m, 2H, ArH), 3.66 (dd, *J* = 6.0, 5.0 Hz, 2H, H-6), 3.24 (d, *J* = 15.5 Hz, 1H, H-7), 3.14 (d, *J* = 15.5 Hz, 1H, H-7'), 1.91 – 1.56 (m, 6H, H-3, H-5, H-9), 1.55 – 1.45 (m, 2H, H-4), 1.41 – 1.13 (m, 4H, H-10, H-11), 0.86 (t, *J* = 7.0 Hz, 3H, H-12).

^{13}C NMR (101 MHz, $CDCl_3$) δ 199.3 (C-8), 138.6 (ArC), 132.9 (ArC), 128.6 (ArC x 2), 128.4 (ArC x 2), 75.4 (C-2), 61.5 (C-6), 44.8 (C-7), 34.3 (C-9), 33.7 (C-3), 25.9 (C-5), 25.4 (C-10), 23.3 (C-11), 19.2 (C-4), 14.2 (C-12).

HRMS (ESI+): Found $[M+H]^+$ = 261.1849; $C_{17}H_{25}O_2$ requires 261.1849 Δ -0.04 ppm

IR (film) v_{max}/cm^{-1}: 2933, 2860, 1677, 1448, 1350, 1085.

N-Methoxy-*N*-methyl-4-(trifluoromethyl)benzamide, S2

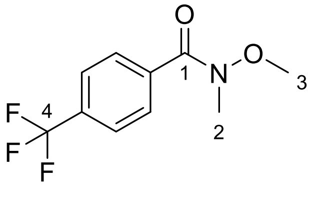

Dimethylhydroxylamine hydrochloride (3.22 g, 33.0 mmol, 1.1 equiv.), CH_2Cl_2 (0.3 M, 100 mL), 4-(trifluoromethyl)benzoyl chloride (6.26 g, 30.0 mmol, 1.0 equiv.), and triethylamine (12.6 mL, 90.0 mmol, 3.0 equiv.) were subjected to **General Procedure 1**. Purification by column chromatography (pentane:EtOAc, 80:20) afforded the title compound **S2** as a colourless oil (6.42 g, 92% yield).

^{1}H NMR (500 MHz, $CDCl_3$) δ 7.78 (d, *J* = 8.0 Hz, 2H, ArCH x 2), 7.66 (d, *J* = 8.0 Hz, 2H, ArCH x 2), 3.52 (s, 3H, H-3 x 3), 3.37 (s, 3H, H-2 x 3).

^{19}F NMR (471 MHz, $CDCl_3$) δ -63.0.

^{13}C NMR (126 MHz, $CDCl_3$) δ 168.6 (C-1), 137.7 (ArC), 132.3 (ArC), 128.7 (ArC x 2), 125.1 (ArC x 2), 122.8 (C-4), 61.4 (C-3), 33.4 (C-2).

The spectroscopic data matched that previously reported in the literature.[1]

[1] Radhoff, N.; Daniliuc, C. G.; Studer, A. *Angew. Chem., Int. Ed.* **2023**, *62*, e202304771.

***N*,4-Dimethoxy-*N*-methylbenzamide, S3**

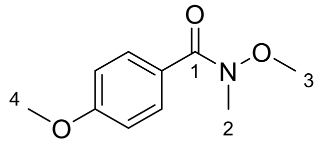

Dimethylhydroxylamine hydrochloride (1.61 g, 16.5 mmol, 1.1 equiv.), CH_2Cl_2 (0.3 M, 50 mL), 4-methoxybenzoyl chloride (2.56 g, 15.0 mmol, 1.0 equiv.), and triethylamine (6.3 mL, 45.0 mmol, 3.0 equiv.) were subjected to **General Procedure 1**. Purification by column chromatography (pentane:EtOAc, 60:40) afforded the title compound **S3** as a colourless oil (1.51 g, 51% yield).

^{1}H NMR (400 MHz, $CDCl_3$) δ 7.77 – 7.68 (m, 2H, ArCH x 2), 6.94 – 6.86 (m, 2H, ArCH x 2), 3.84 (s, 3H, H-4 x 3), 3.56 (s, 3H, H-3 x 3), 3.35 (s, 3H, H-2 x 3).

^{13}C NMR (101 MHz, $CDCl_3$) δ 169.5 (C-1), 161.6 (ArC), 130.7 (ArC x 2), 126.1 (ArC), 113.4 (ArC x 2), 61.0 (C-3), 55.4 (C-4), 34.0 (C-2).

The spectroscopic data matched that previously reported in the literature.[1]

7-Hydroxy-1-(4-(trifluoromethyl)phenyl)hept-2-yn-1-one, S2a

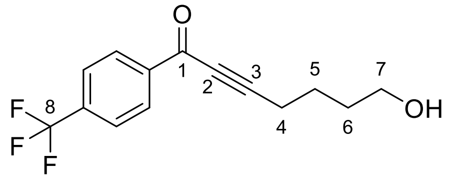

n-BuLi in hexanes (19.2 mL, 2.5 M, 48.0 mmol, 2.4 equiv.), 5-hexyn-1-ol (2.35 g, 24.0 mmol, 1.2 equiv.), THF (0.3 M, 67 mL) and *N*-methoxy-*N*-methyl-4-(trifluoromethyl)benzamide **S2** (4.66 g, 20.0 mmol, 1.0 equiv.) were subjected to **General Procedure 2**. The title compound was afforded after purification by FCC (10-40% EtOAc/Pentane) as a yellow oil (3.76 g, 13.9 mmol, 70% yield).

^{1}H NMR (500 MHz, $CDCl_3$) δ 8.23 (d, *J* = 8.0 Hz, 2H, ArCH x 2), 7.74 (d, *J* = 8.0 Hz, 2H, ArCH x 2), 3.73 (t, *J* = 6.0 Hz, 2H, H_7 x 2), 2.59 (t, *J* = 6.5 Hz, 2H, H_4 x 2), 1.86 – 1.71 (m, 4H, H_5 x 2, H_6 x 2), 1.39 (s, 1H, OH).

^{19}F NMR (471 MHz, $CDCl_3$) δ -63.1.

^{13}C NMR (126 MHz, $CDCl_3$) δ 177.0 (C_1), 139.5 (ArC), 135.2 (ArC), 129.9 (ArC x 2), 125.8 (ArC x 2), 123.7 (C_8), 97.9 (C_3), 79.8 (C_2), 62.3 (C_7), 31.9 (C_6), 24.4 (C_5), 19.2 (C_4).

HRMS (ESI+): Found $[M+H]^+$ = 271.0945; $C_{14}H_{14}F_3O_2$ requires 271. 0940 Δ 1.68 ppm

IR (film) ν_{max}/cm^{-1} 3394, 2920, 2865, 2231, 2188, 1642,1254.

7-Hydroxy-1-(4-methoxyphenyl)hept-2-yn-1-one, S3a

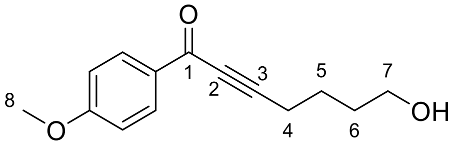

n-BuLi in hexanes (9.8 mL, 2.5 M, 24.6 mmol, 2.4 equiv.), 5-hexyn-1-ol (1.21 g, 12.3 mmol, 1.2 equiv.), THF (0.3 M, 34 mL) and *N*-methoxy-*N*-methyl-4-(trifluoromethyl)benzamide **S3** (2.00 g, 10.2 mmol, 1.0 equiv.) were subjected to **General Procedure 2**. The title compound was afforded after purification by FCC (20-60% EtOAc/Pentane) as a yellow oil (2.11 g, 9.08 mmol, 89% yield).

^1H NMR (400 MHz, $CDCl_3$) δ 8.13 – 8.04 (m, 2H, ArCH x 2), 6.98 – 6.89 (m, 2H, ArCH x 2), 3.88 (s, 3H, H_8 x 3), 3.75 – 3.67 (m, 2H, H_7 x 2), 2.58 – 2.48 (m, 2H, H_4 x 2), 1.82 – 1.69 (m, 4H, H_5 x 2, H_6 x 2).

^{13}C NMR (101 MHz, $CDCl_3$) δ 177.1 (C_1), 164.5 (ArC), 132.1 (ArC x 2), 130.4 (ArC), 113.9 (ArC x 2), 95.5 (C_3), 80.0 (C_2), 62.3 (C_7), 55.7 (C_8), 31.9 (C_6), 24.4 (C_5), 19.2 (C_4).

HRMS (ESI+): Found $[M+H]^+$ = 233.1172; $C_{14}H_{17}O_3$ requires 233. 1172 Δ -0.11 ppm

m.p.: 109-110 °C

IR (film) ν_{max}/cm^{-1} 3389, 2938, 2869, 2236, 2200, 1639, 1449, 1265, 701.

(±)-2-(2-Methyltetrahydro-2*H*-pyran-2-yl)-1-(4-(trifluoromethyl)phenyl)ethan-1-one, 3b

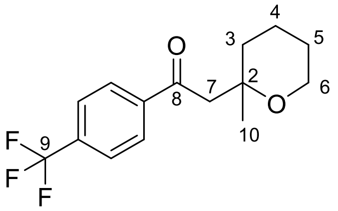

MeLi in Et_2O (13.9 mL, 1.6 M, 22.2 mmol, 6.0 equiv.) was added dropwise to a solution of CuI (2.11 g, 11.1 mmol, 3.0 equiv.) in THF (0.3 M, 37 mL) at –78 °C. The reaction mixture was warmed to 0 °C and stirred for 1 h, affording a colourless solution. The mixture was cooled to –78 °C, and a solution of **S2a** (1.00 g, 3.70 mmol, 1.0 equiv.) in THF (1.0 M, 4 mL) was added dropwise and stirred for 30 min. The reaction was quenched by addition of saturated aqueous ammonium chloride (30 mL) and warmed to RT. The mixture was treated with 35% aqueous ammonium hydroxide solution (30 mL), affording a clear blue aqueous phase. The solution was extracted with EtOAc (3 x 60 mL), and the combined organic phases were washed with HCl (1.0 M, 60 mL), dried over $MgSO_4$, filtered, and concentrated *in vacuo*. Purification by column chromatography (pentane:Et_2O, 95:5) afforded the title compound **3b** as a yellow oil (911 mg, 86% yield).

¹H NMR (500 MHz, $CDCl_3$) δ 8.08 (d, *J* = 8.0 Hz, 2H, ArH), 7.71 (d, *J* = 8.0 Hz, 2H, ArH), 3.73 – 3.62 (m, 2H, H-6), 3.22 – 3.13 (m, 2H, H-7), 1.76 – 1.59 (m, 4H, H-3, H-4), 1.55 – 1.47 (m, 2H, H-5), 1.33 (s, 3H, H-10).

¹⁹F NMR (471 MHz, $CDCl_3$) δ -63.1.

¹³C NMR (126 MHz, $CDCl_3$) δ 198.4 (C-8), 141.1 (ArC), 134.1 (ArC), 129.0 (ArC x 2), 125.6 (ArC x 2), 122.7 (C-9), 73.5 (C-2), 61.8 (C-6), 49.1 (C-7), 35.1 (C-3), 25.8 (C-5), 22.9 (C-10), 19.4 (C-4).

HRMS (ESI+): Found $[M+H]^+$ = 287.1255; $C_{15}H_{18}F_3O_2$ requires 287.1253 Δ 0.54 ppm

IR (film) ν_{max}/cm^{-1}: 3660, 2980, 1677, 1597, 1449, 1378.

(±)-2-(2-Butyltetrahydro-2*H*-pyran-2-yl)-1-(4-methoxyphenyl)ethan-1-one, 3d

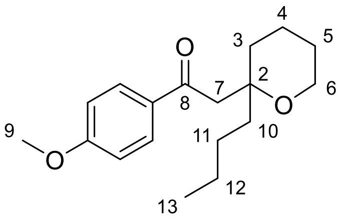

n-BuLi in hexanes (2.5 M, 7.2 mL, 18.1 mmol, 6.0 equiv.) was added dropwise to a solution of CuI (1.72 g, 9.03 mmol, 3.0 equiv.) in THF (0.3 M, 30 mL) at –78 °C. The reaction mixture was warmed to 0 °C and stirred for 1 h, affording a black precipitate. The mixture was cooled to –78 °C and a solution of **S3a** (700 mg, 3.01 mmol, 1.0 equiv.) in THF (1.0 M, 3 mL) was added dropwise and stirred for 30 min. The reaction was quenched by addition of saturated aqueous ammonium chloride (30 mL) and warmed to RT. The mixture was treated with 35% aqueous ammonium hydroxide solution (30 mL), affording a clear blue aqueous phase. The solution was extracted with EtOAc (3 x 60 mL), and the combined organic phases were washed with HCl (1.0 M, 60 mL), dried over $MgSO_4$, filtered, and concentrated *in vacuo*. Purification by column chromatography (pentane: Et_2O, 85:15) afforded the title compound **3d** as a yellow oil (812 mg, 93% yield).

¹H NMR (400 MHz, $CDCl_3$) δ 8.01 – 7.91 (m, 2H, ArH), 6.96 – 6.88 (m, 2H, ArH), 3.86 (s, 3H, H-9), 3.70 – 3.61 (m, 2H, H-6), 3.19 (d, *J* = 15.0 Hz, 1H, H-7), 3.08 (d, *J* = 15.0 Hz, 1H, H-7'), 1.87 – 1.58 (m, 6H, H-3, H-5, H-10), 1.54 – 1.44 (m, 2H, H-4), 1.39 – 1.17 (m, 4H, H-11, H-12), 0.87 (t, *J* = 7.0 Hz, 3H, H-13).

¹³C NMR (101 MHz, $CDCl_3$) δ 197.7 (C-8), 163.4 (ArC), 131.7 (ArC), 130.8 (ArC x 2), 113.7 (ArC x 2), 75.4 (C-2), 61.5 (C-6), 55.6 (C-9), 44.6 (C-7), 34.3 (C-10), 33.7 (C-3), 25.9 (C-5), 25.4 (C-11), 23.3 (C-12), 19.2 (C-4), 14.3 (C-13).

HRMS (ESI+): Found $[M+H]^+$ = 291.1957; $C_{18}H_{27}O_3$ requires 291.1955 Δ 0.77 ppm

IR (film) ν_{max}/cm^{-1}: 2957, 2869, 1670, 1601, 1576, 1510.

(±)-2-(2-Butyltetrahydro-2*H*-pyran-2-yl)-1-(4-(trifluoromethyl)phenyl)ethan-1-one, 3e

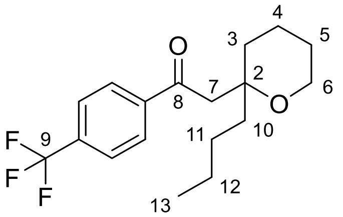

n-BuLi in hexanes (2.5 M, 8.9 mL, 22.2 mmol, 6.0 equiv.) was added dropwise to a solution of CuI (2.11 g, 11.1 mmol, 3.0 equiv.) in THF (0.3 M, 37 mL) at –78 ˚C. The reaction mixture was warmed to 0 °C and stirred for 1 h, affording a black precipitate. The mixture was cooled to –78 °C, and a solution of **S2** (1.00 g, 3.70 mmol, 1.0 equiv.) in THF (1.0 M, 4 mL) was added dropwise and stirred for 30 min. The reaction was quenched by addition of saturated aqueous ammonium chloride (30 mL) and warmed to RT. The mixture was treated with 35% aqueous ammonium hydroxide solution (30 mL), affording a clear blue aqueous phase. The solution was extracted with EtOAc (3 x 60 mL) and the combined organic phases were washed with HCl (1.0 M, 60 mL), dried over $MgSO_4$, filtered, and concentrated *in vacuo*. Purification by column chromatography (pentane: Et_2O, 95:5) afforded the title compound **3e** as a yellow oil (755 mg, 62% yield).

^{1}H NMR (500 MHz, $CDCl_3$) δ 8.07 (d, *J* = 8.0 Hz, 2H, ArH), 7.71 (d, *J* = 8.0 Hz, 2H, ArH), 3.68 – 3.58 (m, 2H, H-6), 3.26 (d, *J* = 15.0 Hz, 1H, H-7), 3.07 (d, *J* = 15.0 Hz, 1H, H-7’), 1.83 (ddd, *J* = 14.0, 12.0, 4.0 Hz, 1H, H-3), 1.77 – 1.56 (m, 5H, H-10, H-4, H-3’), 1.48 (p, *J* = 5.5 Hz, 2H, H-5), 1.42 – 1.15 (m, 4H, H-11, H-12), 0.88 (t, *J* = 7.0 Hz, 3H, H-13).

^{19}F NMR (471 MHz, $CDCl_3$) δ -63.1.

^{13}C NMR (126 MHz, $CDCl_3$) δ 198.6 (C-8), 141.3 (ArC), 134.1 (ArC), 128.9 (ArC x 2), 125.6 (ArC x 2), 123.8 (C-9), 75.5 (C-2), 61.5 (C-6), 45.8 (C-7), 34.0 (C-3), 33.4 (C-10), 25.7 (C-5), 25.5 (C-11), 23.3 (C-12), 19.2 (C-4), 14.2 (C-13).

HRMS (ESI+): Found $[M+H]^+$ = 329.1714; $C_{18}H_{24}F_3O_2$ requires 329.1723 Δ -2.72 ppm.

IR (film) v_{max}/cm^{-1}: 2980, 1685, 1582, 1511, 1461, 1408.

(*E*)-3-(*tert*-Butyl)-7-hydroxy-1-phenylhept-2-en-1-one, 3f

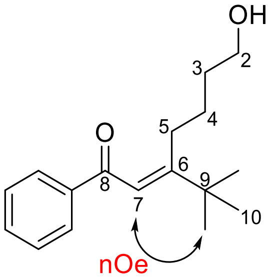

t-BuLi in pentane (1.7 M, 3.5 mL, 6.0 mmol, 6.0 equiv.) was added dropwise to a solution of CuI (572 mg, 3.0 mmol, 3.0 equiv.) in THF (0.1 M, 10 mL) at –78 °C. The reaction mixture was warmed to 0 °C and stirred for 1 h, affording a black precipitate. The mixture was cooled to –78 °C, and a solution of **S1** (202 mg, 1.0 mmol, 1.0 equiv.) in THF (1.0 M, 1 mL) was added dropwise and stirred for 30 min. The reaction was quenched by addition of saturated aqueous ammonium chloride (10 mL) and warmed to RT. The mixture was treated with 35% aqueous ammonium hydroxide solution, affording a clear blue aqueous phase. The solution was extracted with EtOAc (3 x 30 mL), and the combined organic phases were dried over $MgSO_4$, filtered, and concentrated *in vacuo*. Purification by column chromatography (Pentane:EtOAc, 80:20 to 60:40) afforded the title compound **3f** as a colourless oil (102 mg, 39% yield, >95:5 *E*/*Z*).

^{1}H NMR (400 MHz, $CDCl_3$) δ 7.95 – 7.88 (m, 2H, ArH), 7.59 – 7.49 (m, 1H, ArH), 7.50 – 7.40 (m, 2H, ArH), 6.86 (s, 1H, H-7), 3.81 – 3.73 (m, 2H, H-2), 2.67 – 2.57 (m, 2H, H-4), 2.26 (s, 1H, OH), 1.73 – 1.55 (m, 4H, H-3, H-5), 1.22 (s, 9H, H-10).

^{13}C NMR (101 MHz, $CDCl_3$) δ 192.2 (C-8), 173.5 (ArC), 140.1 (C-6), 132.4 (ArC), 128.6 (ArC x 2), 128.3 (ArC x 2), 117.1 (C-7), 61.4 (C-2), 39.0 (C-9), 32.7 (C-3), 29.5 (C-4), 29.0 (C-10), 26.3 (C-5).

HRMS (ESI+): Found $[M+H]^+$ = 261.1848; $C_{17}H_{25}O_2$ requires 261.1849 Δ -0.59 ppm

IR (film) ν_{max}/cm^{-1}: 3503, 2958, 2871, 1703, 1658, 1599.

3.2 Synthesis of THP precursors 3g-3h

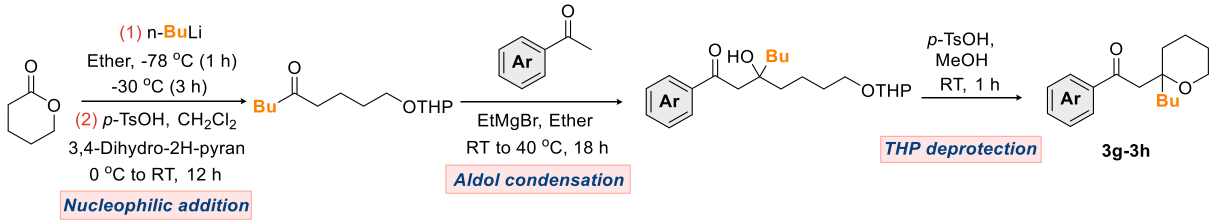

(±)-1-((Tetrahydro-2H-pyran-2-yl)oxy)nonan-5-one, S4

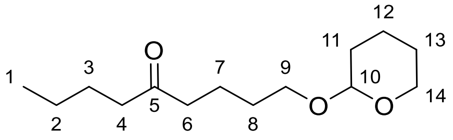

A solution of commercially available δ-valerolactone (6.00 g, 60.0 mmol, 1.0 equiv.) in dry Et_2O (120 mL) was cooled to -78 °C under Argon and n-BuLi (2.5 M, 24 mL, 60 mmol) was added dropwise. The reaction was stirred at -78 °C for 1 h and then warmed to -30 °C and stirred for 1 h, quenched by slow addition of sat. aq. NH_4Cl (100 mL) and extracted with Et_2O (3 × 80 mL). The combined organic layers were dried over Na_2SO_4 and concentrated *in vacuo.* The crude product was used as is in the next step. To a solution of crude keto-alcohol (9.00 g, 56.9 mmol, 1.0 equiv.) and *p*-TsOH·H_2O (541 mg, 2.85 mmol, 0.05 equiv.) in CH_2Cl_2 (120 mL) was added dropwise 3,4-dihydro-2H-pyran (6.00 g, 71.1 mmol, 1.25 equiv.) at 0 °C and stirred for 1.5 h at room temperature. After the addition of sat. $NaHCO_3$ solution, the reaction mixture was extracted with CH_2Cl_2. The combined organic layers were dried over $MgSO_4$, filtered and concentrated *in vacuo*. Purification by column chromatography (pentane: Et_2O, 80:20 to 90:10) afforded the title compound **S4** as a transparent oil (12.1 g, 83%). This compound contained a small impurity from which it could not be separated and was used directly in the next step.

^{1}H NMR (400 MHz, $CDCl_3$) δ 4.60 – 4.51 (m, 1H, H10), 3.84 (ddd, *J* = 11.3, 7.7, 3.2 Hz, 1H, H-14), 3.74 (dt, *J* = 9.7, 6.3 Hz, 1H, H-9), 3.54 – 3.45 (m, 1H, H-14'), 3.45 – 3.29 (m, 1H, H-9'), 2.42 (m, 4H, H-4, H-6), 1.81 (m, 1H, H-12), 1.76 – 1.40 (m, 11H, H-3, H-7, H-8, H-11, H-12, H-13), 1.30 (m, 2H, H-2), 0.87 (t, *J* = 7.3 Hz, 3H, H-1).

^{13}C NMR (101 MHz, $CDCl_3$) δ 211.3 (C-5), 99.0 (C-10), 67.2 (C-9), 62.3 (C-14), 42.6 (C-6), 42.44 (C-4), 30.7 (C-11), 29.3 (C-13), 26.0 (C-7), 25.5 (C-3), 22.3 (C-2), 20.7 (C-8), 19.6 (C-12), 14.0 (C-1).

HRMS (ESI+): Found $[M+Na]^+$ = 265.1777; $C_{14}H_{26}O_3Na$ requires 265.1774, Δ 1.07 ppm

IR (film) ν_{max}/cm^{-1}: 2939, 2872, 1714, 1467, 1383, 1121.

(±)-3-Hydroxy-1-(2,3,4,5,6-pentamethylphenyl)-3-(3-((tetrahydro-2H-pyran-2-yl)oxy)propyl)heptan-1-one, S5

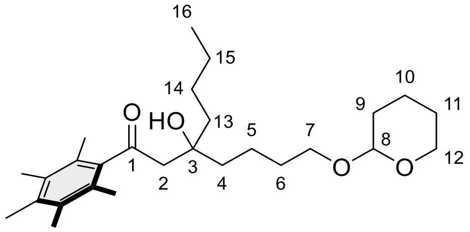

EtMgBr in Et_2O (3.0 M, 5.00 mL, 14.8 mmol, 1.4 equiv.) was added dropwise to a solution of 1-(2,3,4,5,6-pentamethylphenyl)ethan-1-one (2.0 g, 10.6 mmol, 1.0 equiv.) in dry Et_2O (0.3 M, 35 mL) at 0 °C under N_2. The reaction mixture was heated 40 °C for 1 h, which resulted in the formation of a white precipitate. **S4** (2.56 g, 10.6 mmol, 1.0 equiv.) was added dropwise over 5 min. The reaction was maintained at 40 °C for 16 h, cooled to RT and quenched with an aqueous saturated solution of NH_4Cl (10 mL). The resulting solution was extracted with Et_2O (3×20 mL) and the combined organic phases were dried over $MgSO_4$, filtered and concentrated in *vacuo*. Purification by column chromatography (pentane: Et_2O, 95:5 to 80:20) afforded the title compound **S5** as a light yellow oil (3.0 g, 66%) and a 1:1 mixture of inseparable diastereoisomers.

^{1}H NMR (400 MHz, $CDCl_3$) δ 4.57 (dd, *J* = 4.6, 2.7 Hz, 1H, H-8), 3.93 – 3.80 (m, 1H, H-12), 3.78 – 3.73 (m, 1H, H-7), 3.55 – 3.45 (m, 1H, H-12'), 3.44 – 3.36 (m, 1H, H-7'), 2.86 (s, 2H, H-2), 2.23 (s, 3H, $3\times ArCH_3$), 2.18 (s, 6H, $6\times ArCH_3$), 2.13 (s, 6H, $6\times ArCH_3$), 1.89 – 1.77 (m, 1H, H-13/H-4), 1.77 – 1.66 (m, 3H), 1.66 – 1.44 (m, 10H), 1.40 – 1.21 (m, 4H), 0.91 (t, *J* = 7.0 Hz, 3H, H-16).

^{13}C NMR (101 MHz, $CDCl_3$) δ 214.1 (C-1), 140.0 (ArC), 135.9 (ArC), 133.4 (ArC), 127.3 (ArC), 99.1 (C-8), 99.0 (C-8) 73.7 (C-3), 67.6 (C-7), 67.5 (C-7), 62.5 (C-12), 53.1 (C-2), 38.8 (C-4/C-13), 38.7 (C-4/C-13), 38.6 (C-9), 30.9 (C-6), 30.4 (C-4/C-13), 30.3 (C-4/C-13), 25.9 (C-11/C-14), 25.6 (C-11/C-14), 23.4 (C-5/C-10/C-15), 20.3 (C-5/C-10/C-15), 19.82 (C-5/C-10/C-15), 17.1 ($ArCH_3$), 16.8 ($ArCH_3$), 16.0 ($ArCH_3$), 14.2 (C-16).

IR (film) ν_{max}/cm^{-1}: 2940, 2871, 1702, 1602, 1454, 1120.

HRMS (ESI+): Found $[M+Na]^+$ = 455.3139; $C_{27}H_{44}O_4Na$ requires 455.3132, Δ 1.58 ppm.

(±)-2-(2-Butyltetrahydro-2H-pyran-2-yl)-1-(2,3,4,5,6-pentamethylphenyl)ethan-1-one, 3g

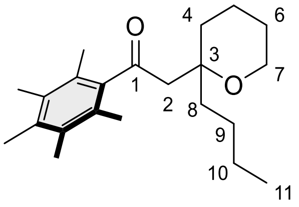

To a solution of **S5** (3.00 g, 6.93 mmol, 1.0 equiv.) in MeOH (0.2 M, 35 mL) was added *p*-TsOH·H_2O (264 mg, 1.39 mmol, 0.2 equiv.) at RT for 3 h. After addition of sat. $NaHCO_3$, extract with CH_2Cl_2. The combined organic layers

were dried over $MgSO_4$, filtered and concentrated under reduced pressure. Purification by column chromatography (Pentane: Et_2O, 98:2 to 95:5) afforded the title compound **3g** as a white solid (2.00 g, 87% yield).

^{1}H NMR (400 MHz, $CDCl_3$) δ 3.61 (qd, *J* = 11.7, 4.7 Hz, 2H, H-7), 3.02 (d, *J* = 18.2 Hz, 1H, H-2), 2.95 (d, *J* = 18.2 Hz, 1H, H-2'), 2.23 (s, 3H, 3×$ArCH_3$), 2.18 (s, 6H, 6×$ArCH_3$), 2.15 (s, 6H, 6×$ArCH_3$), 2.02 – 1.76 (m, 4H, H-4,H-6/8), 1.75 – 1.56 (m, 2H, H-6/8), 1.56 – 1.46 (m, 2H, H-5), 1.41 – 1.17 (m, 4H, H-9, H-10), 0.91 (t, *J* = 6.9 Hz, 3H, H-11).

^{13}C NMR (101 MHz, $CDCl_3$) δ 209.7 (C-1), 141.2 (ArC), 135.4 (ArC), 133.2 (ArC), 127.5 (ArC), 74.7 (C-3), 61.3 (C-7), 50.9 (C-2) , 34.7 (C-8/C-4), 33.2 (C-4/C-8) , 25.9 (C-6), 25.4 (C-9), 23.4 (C-10) , 19.2 (C-5), 17.32 ($ArCH_3$) , 16.8 ($ArCH_3$), 16.1 ($ArCH_3$), 14.3 (C-11).

IR (film) v_{max}/cm^{-1}: 2953, 2932, 2869, 1703, 1457, 1446, 1304.

HRMS (ESI+): Found $[M+H]^+$ = 331.2625; $C_{22}H_{35}O_2$ requires 331.2632 Δ -1.94 ppm.

m.p.: 47-49 °C.

(±)3-Butyl-3-hydroxy-1-mesityl-7-((tetrahydro-2H-pyran-2-yl)oxy)heptan-1-one, S6

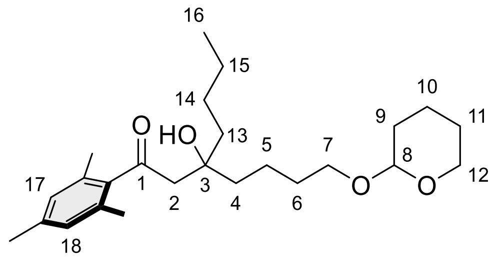

EtMgBr in Et_2O (3.0 M, 2.87 mL, 8.6 mmol, 1.4 equiv.) was added dropwise to a solution of 1-mesitylethan-1-one (1.00 g, 6.2 mmol, 1.0 equiv.) in dry Et_2O (0.3 M, 17 mL) at 0 °C under N_2. The reaction mixture was heated 40 °C for 1 h, which resulted in the formation of a white precipitate. **S4** (1.50 g, 6.2 mmol, 1.0 equiv.) was added dropwise over 5 min. The reaction was maintained at 40 °C for a further 16 h, cooled to RT and quenched with an aqueous saturated solution of NH_4Cl (10 mL). The resulting solution was extracted with Et_2O (3×20 mL) and the combined organic phases were dried over $MgSO_4$, filtered and concentrated in *vacuo*. The combined organic layers were dried ($MgSO_4$) and concentrated *in vacuo.* Purification by column chromatography (Pentane:Et_2O, 95:5) afforded the title compound **S6** as a colourless oil and a 1:1 mixture of inseparable diastereoisomers (1.53 g, 60% yield).

^{1}H NMR (400 MHz, $CDCl_3$) δ 6.82 (s, 2H, H17, H18), 4.56 (dd, *J* = 4.6, 2.7 Hz, 1H, H-8), 3.89 – 3.80 (m, 1H, H-12), 3.77 – 3.68 (m, 1H, H-7), 3.53 – 3.43 (m, 1H, H-7'), 3.42 – 3.34 (m, 1H, H-12), 2.88 (s, 2H, H-2), 2.26 (s, 3H, 3×$ArCH_3$), 2.21 (s, 6H, 6×$ArCH_3$), 1.87 – 1.75 (m, 1H, H-13/H-4), 1.72 – 1.39 (m, 12H), 1.39 – 1.17 (m, 5H), 0.90 (t, *J* = 6.9 Hz, 3H, H-16).

^{13}C NMR (101 MHz, $CDCl_3$) δ 212.8 (C-1), 139.2 (ArC), 138.8 (ArC), 132.5 (ArC), 128.8 (ArC), 99.0 (C-8), 98.9 (C-8), 73.9 (C-3), 67.5 (C-7), 67.4 (C-7), 62.4 (C-12), 52.3 (C-2), 38.9 (C-4/C-13), 38.8 (C-4/C-13), 38.7 (C-9), 30.9 (C-6), 30.4 (C-4/C-13), 30.3 (C-4/C-13), 25.9 (C-11/C-14), 25.6 (C-11/C-14), 23.3 (C-5/C-10/C-15), 21.1 (C-5/C-10/C-15), 20.5 (C-5/C-10/C-15), 19.8 ($ArCH_3$), 19.2 ($ArCH_3$), 14.2 (C-16).

FTIR (neat) v/cm^{-1} = 2940, 2871, 1702, 1600, 1460, 1338.

HRMS (ESI$^+$) m/z Found $[M+Na]^+$ = 427.2811; $C_{25}H_{40}O_4Na$ requires 427.2819, Δ -1.83 ppm.

(±)-2-(2-Butyltetrahydro-2H-pyran-2-yl)-1-mesitylethan-1-one, 3h

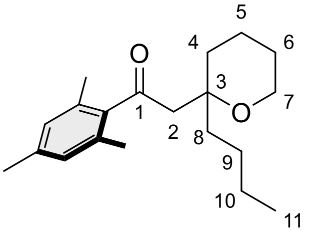

To a solution of **S6** (500 mg, 1.24 mmol, 1.0 equiv.) in MeOH (0.2 M, 6 mL) was added *p*-TsOH·H_2O (48 mg, 0.25 mmol, 0.2 equiv.) at RT for 3 h. After addition of sat. $NaHCO_3$, extract with CH_2Cl_2. The combined organic layers were dried over $MgSO_4$, filtered and concentrated under reduced pressure. Purification by column chromatography (Pentane: Et_2O, 98:2 to 95:5) afforded the title compound **3h** as a colourless oil (205 mg, 55% yield).

^{1}H NMR (400 MHz, $CDCl_3$) δ 6.82 (s, 2H, ArH), 3.66 – 3.51 (m, 2H, H-7), 3.04 (d, J = 17.0 Hz, 1H, H-2), 2.97 (d, J = 17.0 Hz, 1H, H-2'), 2.27 (s, 3H, 3 × $ArCH_3$), 2.25 (s, 6H, 6 × $ArCH_3$), 1.96 – 1.84 (m, 1H, H-4), 1.84 – 1.70 (m, 3H, H-4',H-6/8), 1.74 – 1.53 (m, 2H, H-6/8), 1.56 – 1.46 (m, 2H, H-5), 1.41 – 1.15 (m, 4H, H-9, H-10), 0.90 (t, J = 6.9 Hz, 3H, H-11).

^{13}C NMR (101 MHz, $CDCl_3$) δ 208.5 (C-1), 140.3 (ArC), 138.3 (ArC), 132.8 (ArC), 128.8 (ArC), 75.1 (C-3), 61.3 (C-7), 50.0 (C-2), 34.9 (C-4/C-8), 33.4 (C-4/C-8), 25.8 (C-6), 25.4 (C-9), 23.3 (C-10), 21.1 (C-5), 19.4 ($ArCH_3$), 19.2 ($ArCH_3$), 14.2 (C-11).

HRMS (ESI+): Found $[M+H]^+$ = 303.2324; $C_{20}H_{31}O_2$ requires 303.2319 Δ 1.79 ppm.

IR (film) ν_{max}/cm^{-1}: 2980, 2935, 2869, 2244, 1703, 1445.

3.3 Synthesis of THP precursors 3i-3j

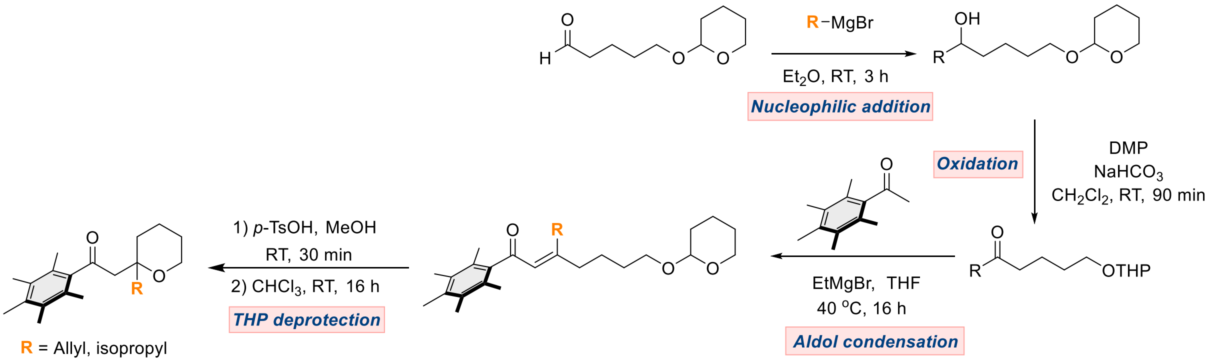

Synthesis of (±)-5-((tetrahydro-2H-pyran-2-yl)oxy)pentanal

(±)-5-((Tetrahydro-2H-pyran-2-yl)oxy)pentan-1-ol, S7

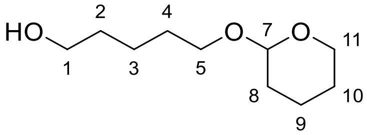

To a solution of 1,5-pentanediol (7.40 g, 71.3 mmol, 3.0 equiv.) and *p*-TsOH·H_2O (226 mg, 1.19 mmol, 0.05 equiv.) in CH_2Cl_2 (100 mL) was added dropwise 3,4-dihydro-2H-pyran (2.00 g, 23.8 mmol, 1.0 equiv.) at 0 °C and stirred for 1.5 h at room temperature. After the addition of sat. $NaHCO_3$ solution, the reaction mixture was extracted with CH_2Cl_2. The combined organic layers were dried over $MgSO_4$, filtered and concentrated under reduced pressure. Purification by column chromatography (pentane:EtOAc, 90:10 to 60:40) afforded the title compound **S7** as a colourless oil (2.79 g, 64% yield).

^{1}H NMR (400 MHz, $CDCl_3$) δ 4.57 (dd, *J* = 4.6, 2.8 Hz, 1H, H7), 3.87 (ddd, *J* = 11.1, 7.4, 3.5 Hz, 1H, H-11), 3.76 (dt, *J* = 9.6, 6.7 Hz, 1H, H-5), 3.66 (td, *J* = 6.5, 5.4 Hz, 2H, H-1), 3.50 (dtd, *J* = 10.7, 4.1, 2.4 Hz, 1H, H-11'), 3.40 (dt, *J* = 9.6, 6.5 Hz, 1H, H-5'), 1.92 – 1.37 (m, 12H, H-2, H-3, H-4, H-8, H-9 and H-10).

^{13}C NMR (101 MHz, $CDCl_3$) δ 99.1 (C-7), 67.7 (C-5), 63.1 (C-1), 62.4 (C-11), 32.7 (C-2), 30.8 (C-8), 29.5 (C-4), 25.6 (C-10), 22.6 (C-3), 19.8 (C-9).

The data matched that previously reported in the literature. [2]

[2] H. Herrera, W. Barros-Parada, M. F. Flores, E. Fuentes-Contreras, J. Bergmann, *J. Chil. Chem. Soc.* **2018**, *63*, 4019 – 4022.

(±)-5-((Tetrahydro-2H-pyran-2-yl)oxy)pentanal, S8

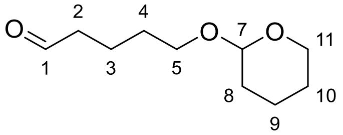

S7 (2.00 g, 10.6 mmol, 1.0 equiv.), $NaHCO_3$ (3.57 g, 42.5 mmol, 4.0 equiv.), Dess-Martin Periodinane (9.00 g, 21.2 mmol, 2.0 equiv.) and CH_2Cl_2 (11 mL) were subjected to **General Procedure 3**. Purification by column chromatography (pentane:EtOAc, 80:20) afforded the title compound **S8** as a colourless oil (1.30 g, 66% yield).

^{1}H NMR (400 MHz, $CDCl_3$) δ 9.78 (t, *J* = 1.8 Hz, 1H, H-1), 4.57 (dd, *J* = 4.5, 2.8 Hz, 1H, H-7), 3.85 (ddd, *J* = 11.0, 7.5, 3.4 Hz, 1H, H-11), 3.76 (dt, *J* = 9.7, 6.3 Hz, 1H, H-5), 3.56 – 3.45 (m, 1H, H-11'), 3.40 (dt, *J* = 9.7, 6.1 Hz, 1H, H-5'), 2.48 (td, *J* = 7.2, 1.7 Hz, 2H, H-2), 1.88 – 1.45 (m, 10H, H-3, H-4, H-8, H-9 and H-10).

^{13}C NMR (101 MHz, $CDCl_3$) δ 202.5 (C-1), 98.9 (C-7), 67.0 (C-5), 62.4 (C-11), 43.7 (C-2), 30.7 (C-8), 29.2 (C-3), 25.5 (C-4), 19.7 (C-10), 19.1 (C-9).

The data matched that previously reported in the literature. [2]

(±)-9-((Tetrahydro-2H-pyran-2-yl)oxy)non-1-en-5-ol, S9

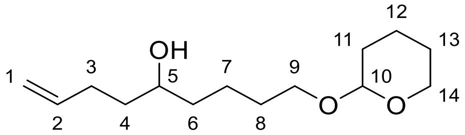

To a solution of **S8** (2.00 g, 10.8 mmol, 1.0 equiv.) in diethyl ether (25 mL) was added a solution of but-3-en-1-ylmagnesium bromide (1.0 M, 21.6 mL, 21.6 mmol, 2.0 equiv.) at 0 C. The reaction solution was stirred for 3 h at room temperature. After reaction, the solution was cooled to 0 °C. To the solution was first added an aqueous saturated solution of NH_4Cl (20 mL) dropwise and then H_2O (20 mL). After stirring for 10 min, the two phases were separated. The aqueous phase was extracted with diethyl ether (2×20 mL). The combined organic phases were dried over $MgSO_4$ and concentrated in *vacuo* to give the title compound **S9** (1.62 g, 62% yield) as a clear oil and a 1:1 mixture of inseparable diastereoisomers.

^{1}H NMR (400 MHz, $CDCl_3$) δ 5.87 (ddt, *J* = 16.9, 10.2, 6.7 Hz, 1H, H-2), 5.19 – 4.95 (m, 2H, H-1), 4.60 (dd, *J* = 4.6, 2.7 Hz, 1H, H-10), 3.89 (ddd, *J* = 11.1, 7.3, 3.4 Hz, 1H, H-14), 3.83 – 3.73 (m, 1H, H-9), 3.65 (m, 1H, H-5), 3.59 – 3.48 (m, 1H, H-14'), 3.43 (m, 1H, H-9'), 2.33 – 2.09 (m, 2H, H-3), 1.85 (tdd, *J* = 11.3, 7.1, 4.1 Hz, 1H, H-12), 1.79 – 1.40 (m, 13H, H-4, H-6, H-7, H-8, H-11, H-12', H-13).

^{13}C NMR (101 MHz, $CDCl_3$) δ 138.6 (C-2), 114.8 (C-1), 99.0 (C-10), 99.0 (C-10), 71.3 (C-5), 67.5 (C-9), 62.4 (C-14), 37.2 (C-6), 36.5 (C-4), 30.8 (C-11), 30.1 (C-3), 29.7 (C-13), 25.5 (C-4), 22.4 (C-8), 19.7 (C-12).

IR (film) ν_{max}/cm^{-1}: 2895, 2792, 1631, 1460, 1357, 1326.

HRMS (ESI+): Found $[M+K]^+$ = 281.1510; $C_{14}H_{26}O_3K$ requires 281.1514, Δ -1.26 ppm.

(±)-9-((Tetrahydro-2H-pyran-2-yl)oxy)non-1-en-5-one, S10

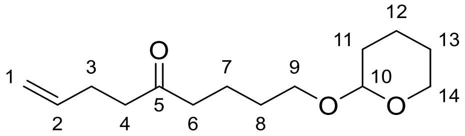

S9 (692 mg, 2.90 mmol, 1.0 equiv.), $NaHCO_3$ (1.00 g, 11.4 mmol, 4.0 equiv.), Dess-Martin Periodinane (2.4 g, 5.71 mmol, 2.0 equiv.) and CH_2Cl_2 (20 mL) were subjected to **General Procedure A**. Purification by column chromatography (pentane: EtOAc, 90:10) afforded the title compound **S10** as a colourless oil (590 mg, 86% yield).

^{1}H NMR (400 MHz, $CDCl_3$) δ 5.82 (ddt, *J* = 16.8, 10.2, 6.5 Hz, 1H, H-2), 5.10 – 4.95 (m, 2H, H-1), 4.58 (dd, *J* = 4.5, 2.8 Hz, 1H, H-10), 3.88 (ddd, *J* = 11.1, 7.5, 3.3 Hz, 1H, H-14), 3.76 (dt, *J* = 9.6, 6.3 Hz, 1H, H-9), 3.57 – 3.47 (m, 1H, H-14'), 3.40 (dt, *J* = 9.5, 6.1 Hz, 1H, H-9'), 2.50 (dt, *J* = 21.0, 7.3 Hz, 4H, H-4, H-6), 2.40 – 2.29 (m, 2H, H-3), 1.94 – 1.78 (m, 1H, H-12), 1.77 – 1.47 (m, 9H, H-7, H-8, H-11, H-12', H-13).

^{13}C NMR (101 MHz, $CDCl_3$) δ 210.2 (C-5), 137.2 (C-2), 115.2 (C-1), 98.9 (C-10), 67.2 (C9), 62.4 (C14), 42.6 (C6), 41.8 (C-4), 30.8 (C-13), 29.3 (C-8), 27.8 (C-3), 25.5 (C-11), 20.6 (C-7), 19.7 (C-12).

IR (film) ν_{max}/cm^{-1}: 2895, 2792, 1715, 1631, 1460, 1357.

HRMS (ESI+): Found $[M+H]^+$ = 241.1791; $C_{14}H_{25}O_3$ requires 241.1798, Δ -3.02 ppm.

(±)-1-(2,3,4,5,6-Pentamethylphenyl)-3-(4-((tetrahydro-2H-pyran-2-yl)oxy)butyl)hepta-2,6-dien-1-one, S11

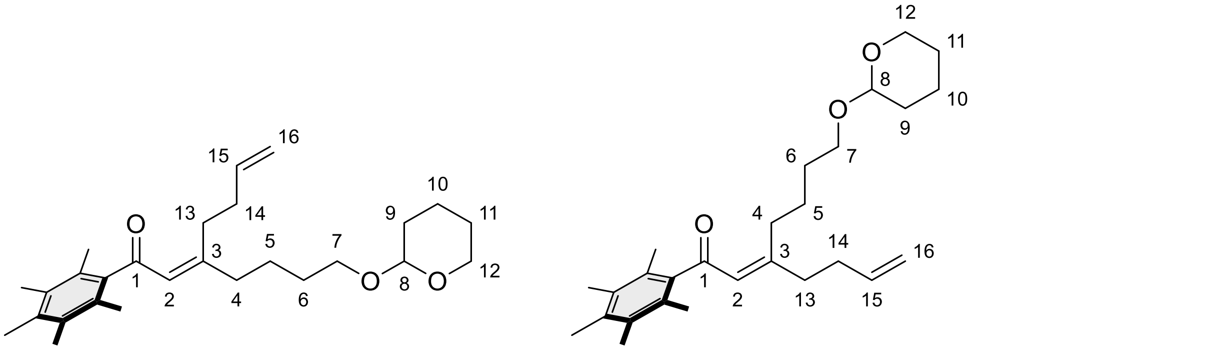

EtMgBr in Et_2O (3.0 M, 1.50 mL, 4.5 mmol, 1.8 equiv.) was added dropwise to a solution of 1-(2,3,4,5,6-pentamethylphenyl)ethan-1-one (970 mg, 5.0 mmol, 2.0 equiv.) in THF (0.2 M, 8 mL) at 0 °C under N_2. The reaction mixture was heated 40 °C for 1 h, which resulted in the formation of a white precipitate. **S10** (600 mg, 2.5 mmol, 1.0 equiv.) was added dropwise over 5 min. The reaction was maintained at 40 °C for a further 16 h, cooled to RT and quenched with an aqueous saturated solution of NH_4Cl (10 mL). The resulting solution was extracted with Et_2O (3×20 mL) and the combined organic phases were dried over $MgSO_4$, filtered and concentrated in *vacuo*. The crude (680 mg) which was obtained as a 5:2 mixture of inseparable diastereoisomers (680 mg) was put into next reaction without purification.

(±)-2-(2-(But-3-en-1-yl)tetrahydro-2H-pyran-2-yl)-1-(2,3,4,5,6-pentamethylphenyl)ethan-1-one, 3i

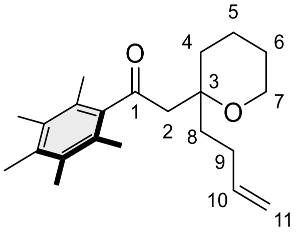

To a solution of crude **S11** (680 mg) in MeOH (0.2 M, 8 mL) was added *p*-TsOH·H_2O (64 mg, 0.33 mmol, 0.2 equiv.) portionwise at RT for 30 min. After addition of sat. $NaHCO_3$, extract with CH_2Cl_2. The combined organic layers were dried over $MgSO_4$, filtered and concentrated under reduced pressure. The resulting crude product was dissolved in chloroform (20 mL) and stirred at RT for 16 h. The solvent was removed under reduced pressure, and purification by column chromatography (Pentane: Et_2O, 97:3) afforded the title compound **3i** as a white solid (359 mg, 44% yield, over two steps).

^{1}H NMR (400 MHz, $CDCl_3$) δ 5.86 – 5.71 (m, 1H, H-10), 5.00 – 4.83 (m, 2H, H-11), 3.61 – 3.44 (m, 2H, H-7), 2.97 (d, *J* = 18.4 Hz, 1H, H-2), 2.86 (d, *J* = 18.4 Hz, 1H, H-2'), 2.15 (s, 3H, 3×$ArCH_3$), 2.11 (s, 6H, 6×$ArCH_3$), 2.07 (s, 6H, 6×$ArCH_3$), 2.06 – 1.94 (m, 3H, H-8, H-9), 1.92 – 1.83 (m, 1H, H-8'), 1.77 (dd, *J* = 6.8, 5.4 Hz, 2H, H-4), 1.70 – 1.51 (m, 2H, H-5), 1.49 – 1.39 (m, 2H, H-6).

^{13}C NMR (101 MHz, $CDCl_3$) δ 209.4 (C-1), 141.0 (ArC), 139.0 (C-10), 135.4 (ArC), 133.1 (ArC), 127.4 (ArC), 114.3 (C-11), 74.3 (C-3), 61.2 (C-7), 50.9 (C-2), 33.9 (C-8), 32.9 (C-4), 27.5 (C-9), 25.8 (C-6), 19.0 (C-5), 17.2 ($ArCH_3$), 16.71 ($ArCH_3$), 16.0 ($ArCH_3$).

IR (film) ν_{max}/cm^{-1}: 2980, 2888, 1703, 1640, 1473, 1461.

HRMS (ESI+): Found $[M+H]^+$ = 329.2484; $C_{22}H_{33}O_2$ requires 329.2475, Δ 2.71ppm.

m.p.: 53-55 °C.

(±)-2-Methyl-7-((tetrahydro-2H-pyran-2-yl)oxy)heptan-3-ol, S12

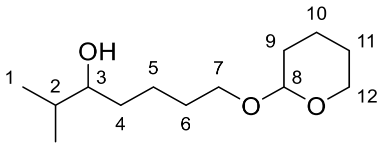

To a solution of aldehyde **S8** (376 mg, 2.00 mmol, 1.0 equiv.) in diethyl ether (20 mL) was added a solution of isopropylmagnesium bromide (2.0 M, 2.00 ml, 4.00 mmol, 2.0 equiv.) at 0 °C. The reaction solution was stirred for 3 h at room temperature. Afterwards, the solution was cooled to 0 °C. To the solution was first added an aqueous saturated solution of NH_4Cl (20 ml) dropwise and then H_2O (20 ml). After stirring for 10 min, the two phases were separated. The aqueous phase was extracted with diethyl ether (2×20 ml). The combined organic phases were dried over $MgSO_4$ and concentrated in *vacuo* to give the title compound **S12** (390 mg, 85%) as a colourless oil and a 1:1 mixture of inseparable diastereoisomers.

^{1}H NMR (400 MHz, $CDCl_3$) δ 4.51 (dd, *J* = 4.5, 2.8 Hz, 1H, H8), 3.80 (ddd, *J* = 11.1, 7.4, 3.4 Hz, 1H, H-12), 3.69 (ddt, *J* = 10.5, 6.8, 3.3 Hz, 1H, C-7), 3.49 – 3.39 (m, 1H, H-12'), 3.33 (m, 2H, H3, H-7'), 1.86 – 1.23 (m, 13H, H-2, H-4, H-5, H-6, H-9, H-10, H-11), 0.85 (d, *J* = 3.9 Hz, 3H, H-1), 0.84 (d, *J* = 3.9 Hz, 3H, H-1').

^{13}C NMR (101 MHz, $CDCl_3$) δ 99.0 (C-8), 98.9 (C-8'), 76.9 (C-3), 67.6 (C-7), 62.4 (C-12), 33.9 (C-4), 33.5 (C-2), 30.8 (C-9), 29.7 (C-6), 25.5 (C-11), 22.8 (C-5), 19.7 (C-10), 18.8 (C-1), 17.1 (C-1').

IR (film) ν_{max}/cm^{-1}: 3445, 2941, 2870, 1672, 1467, 1352.

HRMS (ESI+): Found $[M+H]^+$ = 231.1890; $C_{13}H_{27}O_3$ requires 231.1882, Δ 3.46 ppm.

(±)-2-Methyl-7-((tetrahydro-2H-pyran-2-yl)oxy)heptan-3-one, S13

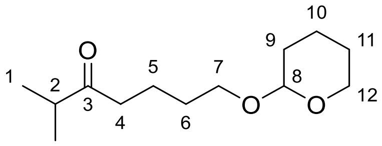

Alcohol **S12** (2.53 g, 11.0 mmol, 1.0 equiv.), $NaHCO_3$ (3.68 g, 44 mmol, 4.0 equiv.), Dess-Martin Periodinane (9.35 g, 22.0 mmol, 2.0 equiv.) and CH_2Cl_2 (17.5 mL) were subjected to **General Procedure 3**. Purification by column chromatography (pentane:Et_2O, 90:10) afforded the title compound **S13** as a colourless oil (2.13 g, 88% yield).

^{1}H NMR (400 MHz, $CDCl_3$) δ 4.57 (dd, *J* = 4.5, 2.8 Hz, 1H, H8), 3.85 (ddd, *J* = 11.1, 7.6, 3.4 Hz, 1H, H-12), 3.74 (dt, *J* = 9.6, 6.4 Hz, 1H, H-7), 3.55 – 3.45 (m, 1H, H-12'), 3.38 (dt, *J* = 9.6, 6.2 Hz, 1H, H-7'), 2.59 (hept, *J* = 6.9 Hz, 1H, H-2), 2.49 (t, *J* = 7.1 Hz, 2H, H-4), 1.90 – 1.45 (m, 10H, H-5, H6, H-9, H-10 and H-11), 1.09 (d, *J* = 6.9 Hz, 6H, H-1).

^{13}C NMR (101 MHz, $CDCl_3$) δ 214.7 (C-3), 98.9 (C-8), 67.3 (C-7), 62.3 (C-12), 40.8 (C-2), 40.0 (C-4), 30.8 (C-9), 29.3 (C-6), 25.5 (C-11), 20.6 (C-5), 19.7 (C-10), 18.3 (C-1).

IR (film) ν_{max}/cm^{-1}: 2941, 2872, 1713, 1467, 1383, 1353.

HRMS (ESI+): Found $[M+H]^+$ = 229.1721; $C_{13}H_{25}O_3$ requires 229.1725, Δ -1.75 ppm.

(±)-3-Isopropyl-1-(2,3,4,5,6-pentamethylphenyl)-7-((tetrahydro-2H-pyran-2-yl)oxy)hept-2-en-1-one, S14

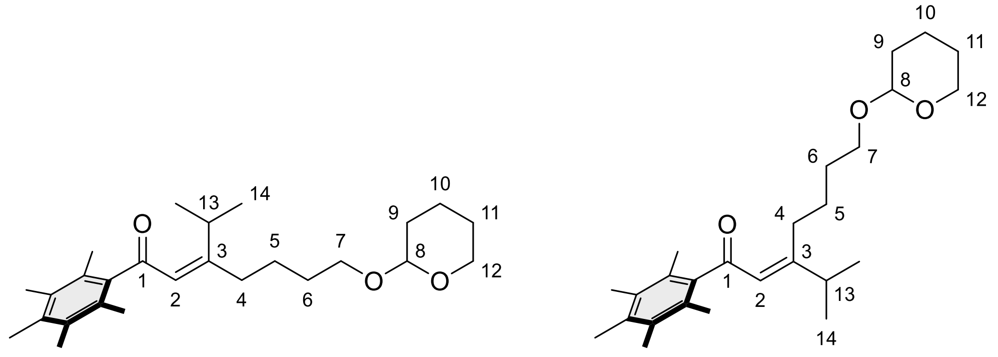

EtMgBr in Et_2O (3.0 M, 0.640 ml, 1.92 mmol, 1.4 equiv.) was added dropwise to a solution of 1-(2,3,4,5,6-pentamethylphenyl)ethan-1-one (302 mg, 1.58 mmol, 1.2 equiv.) in THF (0.2 M, 7.5 ml) at 0 °C under N_2. The reaction mixture was heated 40 °C for 1 h, which resulted in the formation of a white precipitate. **S13** (300 mg, 1.34 mmol, 1.0 equiv.) was added dropwise over 5 min. The reaction was maintained at 40 °C for a further 3 h, cooled to RT and quenched with an aqueous saturated solution of NH_4Cl (10 mL). The resulting solution was extracted with Et_2O (3×20 mL) and the combined organic phases were dried over $MgSO_4$, filtered and concentrated in *vacuo*. Purification by column chromatography (pentane:Et_2O, 90:10) afforded the title compound **S14** as a mixture of inseparable (unassigned) diastereoisomers (220 mg, 40%, 4:1) as a colourless oil. Data is reported on the mixture.

^{1}H NMR (400 MHz, $CDCl_3$) **Major diastereoisomer**: δ 6.19 (s, 1H, H-2), 4.59 (m, 1H, H-8), 3.88 (td, *J* = 7.6, 3.8 Hz, 1H, H-12), 3.76 (dt, *J* = 9.8, 6.5 Hz, 1H, H-7), 3.56 – 3.47 (m, 1H, H-12'), 3.42 (dt, *J* = 9.6, 6.5, 1H, H-7'), 2.62 (m, 2H, H-4), 2.41 (m, 1H, H-13), 2.24 (s, 3H, 3×$ArCH_3$), 2.19 (s, 6H, 6×$ArCH_3$), 2.15 (s, 6H, 6×$ArCH_3$), 1.76 – 1.46 (m, 10H, H-5, H-6, H-9, H-10, H-11), 1.07 (d, *J* = 6.9, 6H, H-14).

^{1}H NMR (400 MHz, $CDCl_3$) **Minor diastereoisomer**: δ 6.14 (s, 1H, H2), 4.55 (m, 1H, H-8), 3.88 (td, *J* = 7.6, 3.8 Hz, 1H, H-12), 3.76 (dt, *J* = 9.8, 6.5 Hz, 1H, H-7), 3.56 – 3.47 (m, 1H, H-12'), 3.42 (dt, *J* = 9.6, 6.5, 1H, H-7'), 2.62 (m, 2H, H-4), 2.41 (m, 1H, H-13), 2.24 (s, 3H, 3×$ArCH_3$), 2.19 (s, 6H, 6×$ArCH_3$), 2.15 (s, 6H, 6×$ArCH_3$), 1.76 – 1.46 (m, 10H, H-5, H-6, H-9, H-10, H-11), 1.07 (d, *J* = 6.9, 6H, H-14).

^{13}C NMR (101 MHz, $CDCl_3$) δ 215.1 (C-1), 170.5 (C-3), 142.5 (ArC), 133.3 (ArC), 132.9 (ArC), 128.4 (ArC), 123.6 (C-2), 99.3 (C-8), 67.9 (C-12), 62.8 (C-7), 36.8 (C-13), 32.6 (C-4), 31.3 (C-9), 30.6 (C-6), 26.0 (C-11), 22.2 (C-14), 20.2 (C-10), 20.1 (C-5), 17.6 ($ArCH_3$), 17.4 ($ArCH_3$), 16.4 ($ArCH_3$).

IR (film) ν_{max}/cm^{-1}: 2992, 2946, 1702, 1442, 1387, 1352.

HRMS (ESI+): Found $[M+H]^+$ = 401.3065; $C_{26}H_{41}O_3$ requires 401.3050, Δ 3.67 ppm.

7-Hydroxy-3-isopropyl-1-(2,3,4,5,6-pentamethylphenyl)hept-2-en-1-one, S15

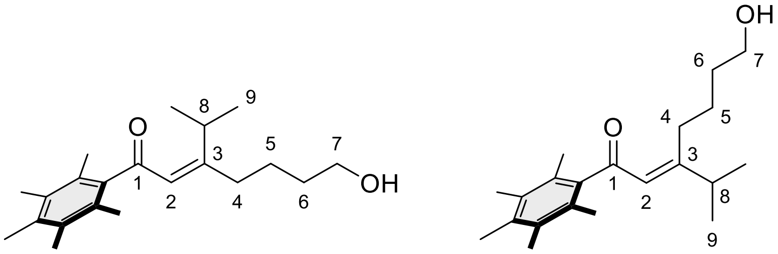

To a solution of an *E/Z* mixture (4:1) of **S14** (127 mg, 0.32 mmol, 1.0 equiv.) in MeOH (0.2 M, 2 mL) was added *p*-TsOH·H_2O (12 mg, 0.06 mmol, 0.2 equiv.) and then stirred at RT for 30 min. After addition of sat. $NaHCO_3$, the reaction was extracted with CH_2Cl_2. The combined organic layers were dried over $MgSO_4$, filtered and concentrated under reduced pressure. Purification by column chromatography (pentane:EtOAc, 90:10) afforded the title compound **S15** as a as a mixture of inseparable diastereoisomers (70 mg, 70%, 5:1) as a colourless oil. Data is reported on the mixture.

^{1}H NMR (400 MHz, $CDCl_3$) **Major diastereoisomer**: δ 6.22 (s, 1H, H-2), 3.76 (m, 2H, H-7), 2.67 – 2.58 (m, 2H, H-4), 2.41 (hept, *J* = 6.9 Hz, 1H, H-8), 2.24 (s, 3H, 3×$ArCH_3$), 2.19 (s, 6H, 6×$ArCH_3$), 2.12 (s, 6H, 6×$ArCH_3$), 1.72 – 1.59 (m, 4H, H-5, H-6), 1.08 (d, *J* = 6.8 Hz, 6H, H-9).

Minor diastereoisomer: δ 6.14 (s, 1H, H-2), 3.65 (m, 1H, H-7), 2.41 (hept, *J* = 6.9 Hz, 1H, H-8), 2.24 (s, 3H, 3×$ArCH_3$), 2.19 (s, 6H, 6×$ArCH_3$), 2.12 (s, 6H, 6×$ArCH_3$), 1.72 – 1.59 (m, 4H, H-5, H-6), 1.08 (d, *J* = 6.8 Hz, 6H, H-9).

^{13}C NMR (101 MHz, $CDCl_3$) δ 201.4 (C-1), 171.0 (C-3), 141.9 (ArC), 135.1 (ArC), 133.0 (ArC), 127.9 (ArC), 122.9 (C-2), 61.6 (C-7), 36.9 (C-8), 32.4 (C-6), 31.5 (C-4), 25.0 (C-5), 21.6 (C-9), 17.1 ($ArCH_3$), 16.7 ($ArCH_3$), 16.0 ($ArCH_3$).

IR (film) ν_{max}/cm^{-1}: 3415, 2925, 1670, 1598, 1462, 1384.

HRMS (ESI+): Found $[M+H]^+$ = 317.2475; $C_{21}H_{33}O_2$ requires 317.2470, Δ -1.47 ppm.

(±)-2-(2-Isopropyltetrahydro-2H-pyran-2-yl)-1-(2,3,4,5,6-pentamethylphenyl)ethan-1-one 3j

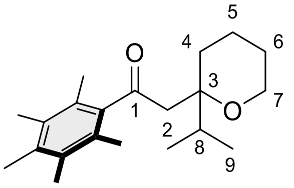

S15 (65.4 mg, 0.16 mmol, 1 equiv.) was dissolved in chloroform (20 mL) and the resulting solution was stirred at RT for 16 h. The reaction mixture was then concentrated under reduced pressure, and purification by column chromatography (Pentane: Et_2O, 97:3) afforded the title compound **3j** as a white solid (60 mg, 86% yield).

^{1}H NMR (400 MHz, $CDCl_3$) δ 3.49 (m, 2H, H-7), 3.03 (d, *J* = 17.1 Hz, 1H, H-2), 2.70 (d, *J* = 17.1 Hz, 1H, H-2'), 2.44 (hept, *J* = 6.9 Hz, 1H, H-8), 2.16 (s, 3H, 3×$ArCH_3$), 2.11 (d, *J* = 3.2 Hz, 12H, 12×$ArCH_3$), 1.91 – 1.80 (m, 1H, H-4), 1.68 – 1.32 (m, 5H, H-4', H-5, H-6), 0.85 (d, *J* = 1.5 Hz, 3H, H-9), 0.83 (d, *J* = 1.5 Hz, 3H, H-9').

^{13}C NMR (101 MHz, $CDCl_3$) δ 210.1 (C-1), 141.7 (ArC), 135.3 (ArC), 133.1 (ArC), 127.7 (ArC), 77.1 (C-3) 61.2 (C-7), 46.1 (C-2), 30.8 (C-8), 27.8 (C-4), 25.6 (C-6), 19.1 (C-5), 17.4 ($ArCH_3$), 17.2 ($ArCH_3$), 16.8 ($ArCH_3$), 16.4 (C-9), 16.0 (C-9').

IR (film) ν_{max}/cm^{-1}: 2980, 2925, 1704, 1598, 1461, 1383.

HRMS (ESI+): Found $[M+H]^+$ = 317.2475; $C_{21}H_{33}O_2$ requires 317.2472, Δ -0.97 ppm.

m.p.: 65-67 °C

HRMS (ESI+): Found $[M+H]^+$ = 317.2475; $C_{21}H_{33}O_2$ requires 317.2472, Δ -0.97 ppm.

((3-Bromopropoxy)methyl)benzene, S16

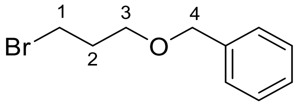

To a solution of benzyloxypropan-1-ol (3.40 g, 20.5 mmol) in dry CH_2Cl_2 (50 mL) was added CBr_4 (8.5 g, 25.6 mmol). After cooling the solution to 0 °C, PPh_3 (9.62 g, 36.7 mmol) was added in portions. The resulting red solution was stirred at room temperature for 18 h. The solvent was removed under reduced pressure, and the precipitate washed several times with ether (6 x 100 mL) and filtered. The collective ether extracts were concentrated to afford a yellow oil. Purification by column chromatography (pentane:Et_2O, 99:1) afforded the title compound **S16** as a pale yellow oil (3.46 g, 76% yield).

^{1}H NMR (400 MHz, $CDCl_3$) δ 7.47 – 7.24 (m, 5H, 5×ArH), 4.55 (s, 2H, H-4), 3.64 (t, *J* = 6.5 Hz, 2H, H-1), 3.57 (t, *J* = 6.5 Hz, 2H, H-3), 2.17 (p, *J* = 6.2 Hz, 2H, H-2).

^{13}C NMR (101 MHz, $CDCl_3$) δ 138.3 (ArC), 128.4 (ArC), 127.7 (ArC), 127.7 (ArC), 73.2 (C-4), 67.7 (C-1), 32.9 (C-2), 30.7 (C-3).

The data matched that previously reported in the literature. [3]

[3] K. J. Frankowski, J. E. Golden, Y. Zeng, Y. Lei, J. Aube, *J. Am. Chem. Soc*. **2008**, *130*, 6018 – 6024.

(±)-1-(Benzyloxy)-8-((tetrahydro-2H-pyran-2-yl)oxy)octan-4-ol, S17

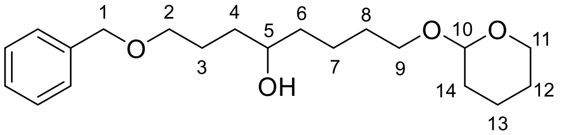

To a solution of **S8**(980 mg, 5.26 mmol, 1.0 equiv.) in diethyl ether (25 mL) was added a solution of (3-(benzyloxy)propyl)magnesium bromide (1.0 M, 10.6 mL, 10.6 mmol, 2.0 equiv.) at 0 °C. The reaction solution was stirred for 3 h at room temperature. After reaction, the solution was cooled to 0 °C. To the solution was first added an aqueous saturated solution of NH_4Cl (20 mL) dropwise and then H_2O (20 mL). After stirring for 10 min, the two phases were separated. The aqueous phase was extracted with diethyl ether (2×20 mL). The combined organic phases were dried over $MgSO_4$ and concentrated in *vacuo* to give the title compound **S17** as a mixture of diastereomers (980 mg, 55%, 70:30 d.r.) as a colourless oil.

Major diastereomer: ^{1}H NMR (400 MHz, $CDCl_3$) δ 7.30 – 7.15 (m, 5H, 5×ArH), 4.49 (t, *J* = 3.6 Hz, 1H, H-10), 4.43 (d, *J* = 1.7 Hz, 2H, H-1), 3.78 (ddd, *J* = 11.0, 7.4, 3.3 Hz, 1H, H-11), 3.71 – 3.61 (m, 1H, H-9), 3.51 (m, 1H, H-5), 3.42 (m, 3H, H2, H-11'), 3.31 (dt, *J* = 9.5, 6.5 Hz, 1H, H-9'), 1.83 – 1.24 (m, 16H, H-3, H-4 ,H-6, H-7, H-8, H-12, H-13,H-14).

^{13}C NMR (101 MHz, $CDCl_3$) δ 138.3 (ArC), 128.4 (ArC), 127.8 (ArC), 127.6 (ArC), 98.9 (C10), 73.2 (C-1), 71.4 (C-5), 70.6 (C-2), 67.6 (C-9), 62.4 (C-11), 37.3 (C-6), 34.6 (C-4), 30.8 (C-14), 29.7 (C-3), 26.2 (C-7), 25.5 (C-12), 22.5 (C-13), 19.7 (C-8).

Diagnostic peaks for minor diastereomer: ^{1}H NMR (400 MHz, $CDCl_3$) δ 3.67 (m, 2H), 3.56 (t, *J* = 5.9 Hz, 2H), 1.77 (m, 2H).

IR (film) ν_{max}/cm^{-1}: 3405, 2980, 2886, 1460, 1381, 1252.

HRMS (ESI+): Found $[M+Na]^+$ = 359.2200; $C_{20}H_{32}O_4Na$ requires 359.2193, Δ 2.00 ppm.

(±)-1-(Benzyloxy)-8-((tetrahydro-2H-pyran-2-yl)oxy)octan-4-one, S18

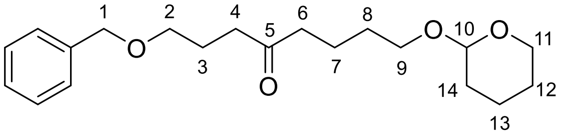

S17 (672 mg, 2.00 mmol, 1 equiv.), $NaHCO_3$ (670 mg, 8.0 mmol, 4.0 equiv.), Dess-Martin Periodinane (1.70 g, 4.01 mmol, 2.0 equiv.) and CH_2Cl_2 (20 mL) were subjected to **General Procedure 3**. Purification by column chromatography (pentane:EtOAc, 90:10) afforded the title compound **S18** as a colourless oil (493 mg, 75% yield).

^{1}H NMR (400 MHz, $CDCl_3$) δ 7.32 – 7.17 (m, 5H, 5×ArH), 4.49 (t, *J* = 3.6 Hz, 1H, H-10), 4.41 (s, 2H, H-1), 3.78 (ddd, *J* = 11.1, 7.5, 3.4 Hz, 1H, H-11), 3.66 (dt, *J* = 9.5, 6.3 Hz, 1H, H-9), 3.41 (m, 3H, H2, H-11'), 3.30 (dt, *J* = 9.5, 6.1 Hz, 1H, H-9'), 2.45 (t, *J* = 7.2 Hz, 2H, H-4), 2.37 (t, *J* = 7.2 Hz, 2H, H-6), 1.93 – 1.39 (m, 12H, H3, H7, H8, H12, H-13, H-14).

^{13}C NMR (101 MHz, $CDCl_3$) δ 210.9 (C-5), 138.4 (ArC), 128.4 (ArC), 127.7 (ArC), 127.6 (ArC), 98.9 (C-10), 72.9 (C-1), 69.4 (C-2), 67.2 (C-9), 62.3 (C-11), 42.6 (C-6), 39.4 (C-4), 30.7 (C-7), 29.3 (C-13), 25.5 (C-12), 23.9 (C-3), 20.7 (C-8), 19.7 (C-14).

IR (film) ν_{max}/cm^{-1}: 2942, 2870, 1715, 1641, 1440, 1413.

HRMS (ESI+): Found $[M+H]^+$ = 335.2229; $C_{20}H_{31}O_4$ requires 335.2217, Δ 3.71 ppm.

(±)-3-(3-(Benzyloxy)propyl)-1-(2,3,4,5,6-pentamethylphenyl)-7-((tetrahydro-2H-pyran-2-yl)oxy)hept-2-en-1-one, S19

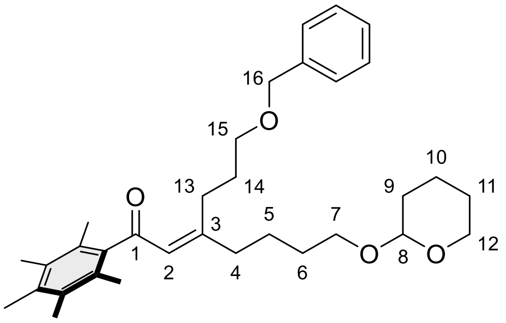

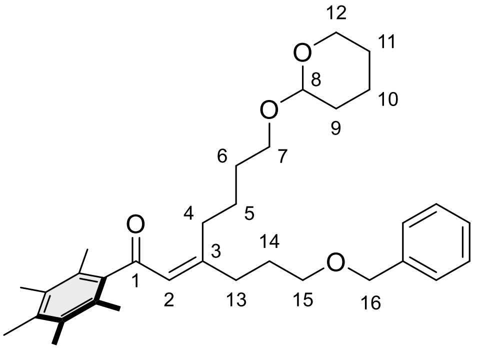

EtMgBr in Et_2O (3.0 M, 0.93 ml, 2.80 mmol, 2.0 equiv.) was added dropwise to a solution of 1-(2,3,4,5,6-pentamethylphenyl)ethan-1-one (532 mg, 2.80 mmol, 2.0 equiv.) in THF (0.2 M, 7.5 mL) at 0 °C under N_2. The reaction mixture was heated 40 °C for 1 h, which resulted in the formation of a white precipitate. **S18** (468 mg,

1.40 mmol, 1.0 equiv.) was added dropwise over 10 min. The reaction was maintained at 40 °C for a further 16 h, cooled to RT and quenched with an aqueous saturated solution of NH_4Cl (10 mL). The resulting solution was extracted with Et_2O (3×20 mL) and the combined organic phases were dried over $MgSO_4$, filtered and concentrated in *vacuo*. Purification by column chromatography (pentane:Et_2O, 90:10) afforded the title compound **S19** as a mixture of inseparable isomers (513 mg, 72% yield, 1:1) as a colourless oil. Data is reported on the mixture.

^{1}H NMR (400 MHz, $CDCl_3$) **Major diastereoisomer**: δ 7.31 – 7.14 (m, 5H, 5×ArH), 6.13 (s, 1H, H-2), 4.51 (t, *J* = 3.6 Hz, 1H, H-8), 4.44 (s, 2H, H-16), 3.77 (ddd, *J* = 15.1, 11.3, 7.8 Hz, 1H, H-12), 3.72 – 3.59 (m, 1H, H-7), 3.45 (m, 3H, H12', H-15), 3.31 (td, *J* = 9.6, 5.5 Hz, 1H, H-7'), 2.61 (m, 2H, H-13), 2.20 (t, *J* = 7.7 Hz, 2H, H-4), 2.15 (s, 3H, 3×$ArCH_3$), 2.10 (s, 6H, 6×$ArCH_3$), 2.02 (s, 6H, 6×$ArCH_3$), 1.86 – 1.68 (m, 4H, H-5, H-14), 1.50 (m, 8H, H-6, H-9, H-10, H-11).

Minor diastereoisomer: δ 7.31 – 7.14 (m, 5H, 5×ArH), 6.11 (s, 1H, H-2), 4.47 (m, 1H, H-8), 4.40 (s, 2H, H-16), 3.77 (ddd, *J* = 15.1, 11.3, 7.8 Hz, 1H, H-12), 3.72 – 3.59 (m, 1H, H-7), 3.39 (m, 3H, H-12', H-15), 3.31 (td, *J* = 9.6, 5.5 Hz, 1H, H-7'), 2.61 (m, 2H, H-13), 2.20 (t, *J* = 7.7 Hz, 2H, H-4), 2.15 (s, 3H, 3×$ArCH_3$), 2.10 (s, 6H, 6×$ArCH_3$), 2.02 (s, 6H, 6×$ArCH_3$), 1.86 – 1.68 (m, 4H, H-5, H-14), 1.50 (m, 8H, H-6, H-9, H-10, H-11).

^{13}C NMR (101 MHz, $CDCl_3$) δ 200.7 (C-1), 200.7 (C-1'), 163.3 (C-3), 141.8 (ArC), 138.6 (ArC), 138.4 (ArC'), 135.0 (ArC), 132.9 (ArC), 128.4 (ArC), 128.4 (ArC'), 127.9 (ArC), 127.8 (ArC'), 127.7 (ArC), 127.6 (ArC), 127.6 (ArC'), 127.5 (C-2), 125.8 (ArC), 125.8 (ArC'), 98.9 (C-8), 98.9 (C-8'), 73.0 (C-16), 72.9 (C-16'), 70.5 (C-15), 69.5 (C-15'), 67.4 (C-7), 67.2 (C-7'), 62.4 (C-12), 35.2 (C-13), 32.3 (C-4), 30.8 (C-9), 30.7 (C-9'), 30.0 (C-6), 29.4 (C-6'), 28.7 (C-14), 27.9 (C-5), 25.5 (C-11), 25.5 (C-11), 19.7 (C-10), 19.7 (C-10'), 17.2 ($ArCH_3$), 16.7 ($ArCH_3$), 16.0 ($ArCH_3$).

IR (film) ν_{max}/cm^{-1}: 2923, 2886, 1702, 1532, 1460, 1344.

HRMS (ESI+): Found $[M+H]^+$ = 507.3458; $C_{33}H_{47}O_4$ requires 507.3469, Δ -2.09 ppm.

3-(3-(Benzyloxy)propyl)-7-hydroxy-1-(2,3,4,5,6-pentamethylphenyl)hept-2-en-1-one, S20

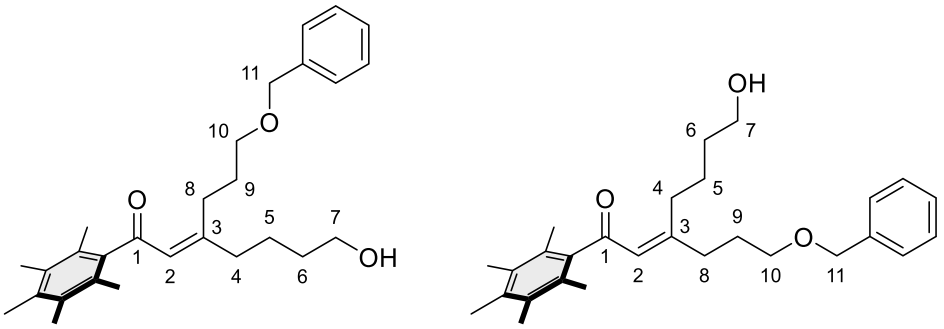

To a solution of E/Z mixture (1:1) of **S19** (140 mg, 0.28 mmol, 1.0 equiv.) in MeOH (0.2 M, 1.5 mL) was added *p*-TsOH·H_2O (10 mg, 0.06 mmol, 0.2 equiv.) portionwise at RT for 30 min. After addition of sat. $NaHCO_3$, extract with CH_2Cl_2. The combined organic layers were dried over $MgSO_4$, filtered and concentrated under reduced pressure. Purification by column chromatography (pentane:EtOAc, 90:10) afforded the title compound **S20** as a inseparable mixture diastereoisomers as a colourless oil (108 mg, 91% yield, 1:1). Data is reported on the mixture.

^{1}H NMR (400 MHz, $CDCl_3$) **Major diastereoisomer**: δ 7.30 – 7.15 (m, 5H, 5×ArH), 6.13 (s, 1H, H-2), 4.45 (s, 2H, H-11), 3.69 – 3.61 (m, 2H, H-7), 3.46 (t, *J* = 6.6 Hz, 2H, H-10), 2.67 – 2.59 (m, 2H, H-8), 2.22 (t, *J* = 7.7 Hz, 2H, H-4),

2.16 (s, 3H, 3×ArCH3), 2.11 (s, 6H, 6×ArCH3), 2.03 (s, 6H, 6×ArCH3), 1.84 – 1.66 (m, 2H, H-9), 1.61 – 1.42 (m, 4H, H-5, H-6).

Minor diastereoisomer: δ 7.30 – 7.15 (m, 5H, 5×ArH), 6.13 (s, 1H, H2), 4.40 (s, 2H, H-11), 3.58 – 3.53 (m, 2H, H-7), 3.39 (t, *J* = 6.3 Hz, 2H, H-7), 2.56 (m, 2H, H-8), 2.22 (t, *J* = 7.7 Hz, 1H, H-4), 2.16 (s, 3H, 3×ArCH3), 2.11 (s, 6H, 6×ArCH3), 2.03 (s, 6H, 6×ArCH3), 1.84 – 1.66 (m, 2H, H-9), 1.61 – 1.42 (m, 4H, H-5, H-6).

^{13}C NMR (101 MHz, $CDCl_3$) δ 200.9 (C1), 200.7 (C1'), 164.2 (C-3), 163.4 (C-3'), 141.7 (ArC), 138.3 (ArC), 135.1 (ArC), 135.1 (ArC'), 133.0 (ArC), 128.4 (ArC), 128.4 (ArC'), 127.8 (ArC), 127.8 (ArC'), 127.7 (ArC), 127.7 (ArC'), 127.5 (C-2), 125.7 (ArC), 73.0 (C-11), 70.5 (C-10), 62.6 (C-7), 38.6 (C-4), 32.3 (C-6), 29.4 (C-8), 28.7 (C-9), 24.5 (C-5), 17.1 (ArCH3), 16.7 (ArCH3), 16.0 (ArCH3).

IR (film) ν_{max}/cm^{-1}: 3405, 2923, 2886, 1670, 1597, 1460.

HRMS (ESI+): Found $[M+H]^+$ = 423.2900; $C_{28}H_{39}O_3$ requires 423.2894, Δ 1.48 ppm.

2-(2-(3-(Benzyloxy)propyl)tetrahydro-2H-pyran-2-yl)-1-(2,3,4,5,6-pentamethylphenyl)ethan-1-one, 3k

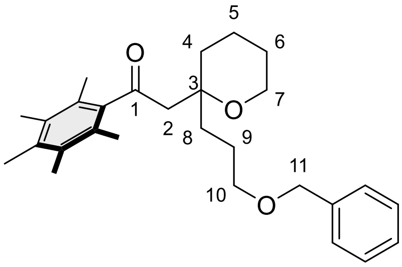

Compound **S20** (168 mg, 0.400 mmol, 1.0 equiv.) was dissolved in chloroform (20 mL) and the resulting solution was stirred at RT for 16 h. The reaction mixture was then concentrated under reduced pressure, and purification by column chromatography (Pentane: Et_2O, 90:10) afforded the title compound as a white solid (119 mg, 70% yield).

^{1}H NMR (400 MHz, $CDCl_3$) δ 7.44 – 7.05 (m, 5H, 5×ArH), 4.44 (s, 2H, H-11), 3.47 (m, 2H, H-10), 3.42 (t, *J* = 6.7 Hz, 2H, H-7), 2.96 (d, *J* = 18.2 Hz, 1H, H-2), 2.86 (d, *J* = 18.2 Hz, 1H, H-2'), 2.15 (s, 3H, 3×ArCH3), 2.10 (s, 6H, 6×ArCH3), 2.06 (s, 6H, 6×ArCH3), 1.98 (ddd, *J* = 14.1, 11.9, 4.8 Hz, 1H, H-4), 1.83 (ddd, *J* = 14.0, 11.5, 5.1 Hz, 1H, H-4'), 1.75 (t, *J* = 6.1 Hz, 2H, H-8), 1.71 – 1.46 (m, 4H, H-5, H-6), 1.44 (ddt, *J* = 10.0, 6.1, 3.7 Hz, 2H, H-9).

^{13}C NMR (101 MHz, $CDCl_3$) δ 209.5 (C-1), 141.0 (ArC), 138.8 (ArC), 135.4 (ArC), 133.1 (ArC), 128.4 (ArC), 127.6 (ArC), 127.5 (ArC), 127.4 (ArC), 74.3 (C-3), 72.8 (C-11), 70.8 (C-7), 61.2 (C-10), 50.9 (C-2), 32.9 (C-8), 31.1 (C-4), 25.7 (C-9), 23.5 (C-5), 19.1 (C-6), 17.2 (ArCH3), 16.7 (ArCH3), 16.0 (ArCH3).

IR (film) ν_{max}/cm^{-1}: 2980, 1700, 1453, 1403, 1257, 1081.

HRMS (ESI+): Found $[M+K]^+$ = 461.2463; $C_{28}H_{38}O_3K$ requires 461.2453, Δ 2.27 ppm.

m.p.: 54-56 °C.

Synthesis of Binol ligand L1

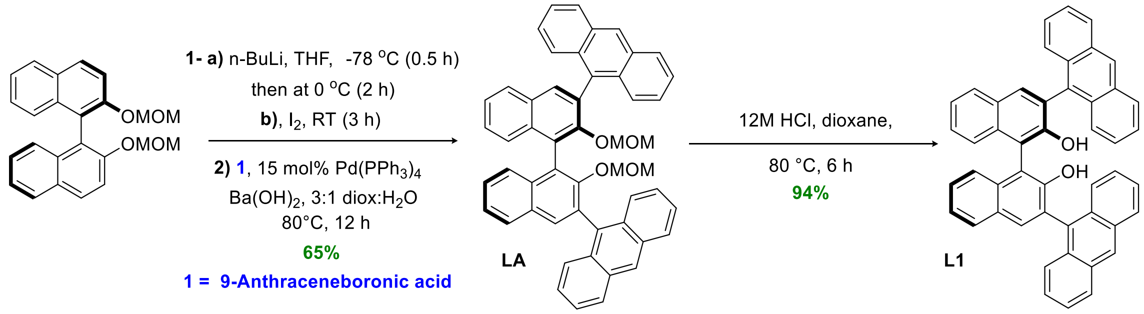

A solution of commercially available of (*R*)-OMOM BINOL (CAS number: 173831-50-0) (5.00 g, 13.4 mmol, 1.00 equiv.) in dry THF (265 mL) was cooled to -78 °C under N_2 and n-BuLi (16 mL, 2.50 M in hexanes, 40.2 mmol, 3.00 equiv.) was added dropwise. The reaction was stirred at -78 °C for 30 min and then warmed to 0 °C and stirred for 2 h. Then the reaction mixture was cooled to -78 °C, I_2 (10.2 g, 40.2 mmol, 3.00 equiv.) was added as a solid, and the reaction mixture was warmed to RT for 5 h and quenched with an aqueous saturated solution of NH_4Cl (30 mL). The resulting solution was extracted with CH_2Cl_2 (3 × 200 mL). The combined organic phases were washed with saturated solution $Na_2S_2O_3$ (3 x 200 mL), then the combined organic phases were washed with brine (1 x 200 mL) and the organic phase was dried over Na_2SO_4 and concentrated *in vacuo.* The crude product was used as is in the next step. In a flask A crude (*R*)-Iodo BINOL (8.00 g, 12.8 mmol, 1.0 equiv.), 9-anthracenylboronic acid (6.80 g, 30.7 mmol, 2.4 equiv.), $Pd(PPh_3)_4$ (2.22 g, 1.92 mmol, 0.15 equiv.), and $Ba(OH)_2$ (11.0 g, 64.0 mmol, 5.0 equiv.) were combined and purged with nitrogen for 15 min. A mixture of 3:1 dioxane:H_2O (130 mL) was added and stirred at 80 °C for 12 h. The resulting reaction was cooled to RT, then diluted with 1M AcOH and CH_2Cl_2 until a homogeneous biphasic solution resulted. The aqueous layer was extracted twice with CH_2Cl_2. The combined organic layers were washed with H_2O (3 × 200 mL) and brine (3 × 200 mL). The organic layer was then dried over Na_2SO_4 and concentrated *in vacuo*. Purification by column chromatography (Pentane: CH_2Cl_2, 95:5 to 60:40) afforded **LA** as a pale yellow solid (6.00 g, 65% yield).

^{1}H NMR (400 MHz, $CDCl_3$) δ 8.54 (s, 2H, 2 × ArH), 8.11 – 7.96 (m, 8H, 8 × ArH), 7.91 (d, *J* = 7.1 Hz, 2H, 2 × ArH), 7.80 (d, *J* = 8.8 Hz, 2H, 2 × ArH), 7.65 (d, *J* = 8.8 Hz, 2H, 2 × ArH), 7.55 – 7.37 (m, 10H, 10 × ArH), 7.27 – 7.18 (m, 2H, 2 × ArH), 4.32 – 4.20 (m, 4H, 2 × CH_2), 1.88 (s, 6H, 3 × OCH_3).

^{13}C NMR (101 MHz, $CDCl_3$) δ 152.8 (ArC), 134.3 (ArC), 133.5 (ArC), 133.2 (ArC), 132.6 (ArC), 131.5 (ArC), 131.5 (ArC) , 131.0 (ArC), 130.9 (ArC), 130.8 (ArC) , 128.6 (ArC) , 128.4 (ArC) , 128.2 (ArC) , 127.2 (ArC), 126.9 (ArC), 126.8 (ArC), 126.5 (ArC) , 126.0 (ArC), 125.8 (ArC), 125.5 (ArC), 125.32 (ArC), 125.28 (ArC), 98.38 (OCH_2), 55.53 (OCH_3). The spectral data matched that previously reported in the literature.[4]

[4] I. T. Crouch, R. K. Neff, D. E. Frantz, *J. Am. Chem. Soc.* **2013**, 135, 4970.

A solution of **LA** (6.00 g, 9.40 mmol, 1.00 equiv.) in dioxane (70 mL) and 37% HCl (9 mL, 108.7 mmol, 11.6 equiv.) and stirred at 60 °C for 6 h. The reaction was monitored by TLC (Pentane: CH_2Cl_2, 60:40). The reaction was concentrated *in vacuo* to give a crude brown solid. This solid resuspended in CH_2Cl_2 and stirred with Na_2SO_4 for 10 min to get rid of residual water and filtered off. The resulting solution was then concentrated *in vacuo* to form a yellow solid. Purification by column chromatography (Pentane: CH_2Cl_2, 87:13 to 60:40) afforded the title ligand

L1 as an off-white solid (4.90 g, 94% yield).

^{1}H NMR (400 MHz, $CDCl_3$) δ 8.58 (s, 2H, 2 × ArH), 8.13 – 8.00 (m, 6H, 6 × ArH), 7.90 (dd, *J* = 22.7, 8.2 Hz, 4H, 4 × ArH), 7.68 (d, *J* = 8.8 Hz, 2H, 2 × ArH), 7.60 (d, *J* = 8.2 Hz, 2H, 2 × ArH), 7.55 – 7.38 (m, 10H, 10 × ArH), 7.26 (d, *J* = 15.5 Hz, 2H, 2 × ArH), 5.11 (s, 2H, 2 × OH).

^{13}C NMR (101 MHz, $CDCl_3$) δ 151.2 (ArC), 134.1 (ArC), 133.2 (ArC) , 131.7 (ArC), 131.6 (ArC), 131.0 (ArC), 130.9 (ArC), 129.4 (ArC), 128.8 (ArC), 128.7 (ArC), 128.6 (ArC), 127.9 (ArC), 127.5 (ArC), 127.3 (ArC), 126.4 (ArC), 126.3 (ArC), 126.3 (ArC), 125.6 (ArC), 125.5 (ArC), 125.0 (ArC), 124.4 (ArC), 113.7 (ArC).

HPLC conditions: Enantiopurity of commercial ligand **L1** (*R*) was determined by HPLC with a Chiralpak® IA column (95:5 hexane:IPA, 1.0 mL min^{-1}, 254 nm, room temperature); t_r (minor) = 27.30 min.

HPLC conditions: Enantiopurity of commercial ligand **L1** (*S*) was determined by HPLC with a Chiralpak® IA column (95:5 hexane:IPA, 1.0 mL min^{-1}, 254 nm, room temperature); t_r = 22.45 min.

HPLC conditions: Enantiopurity of synthesised and recycled ligand L1 (*R*) was determined by HPLC with a Chiralpak® IA column (95:5 hexane:IPA, 1.0 mL min^{-1}, 254 nm, room temperature); t_r = 27.77 min.

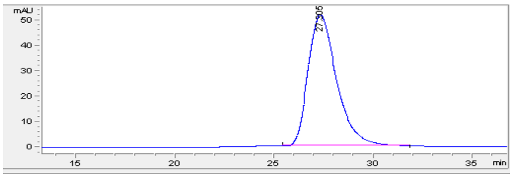

Commercial Ligand L1 *(R)*

(*R*)-3,3'-Di(anthracen-9-yl)-[1,1'-binaphthalene]-2,2'-diol,

CAS No: 361342-49-6

#	Time	Type	Area	Height	Width	Area%	Symmetry
1	27.305	BB	5231.9	52.1	1.4766	100.000	0.704

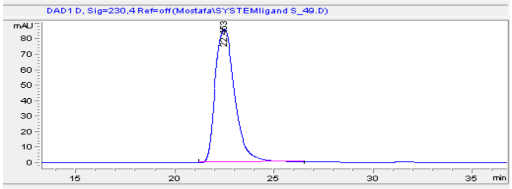

Commercial Ligand L1 *(S)*

(*S*)-3,3'-Di(anthracen-9-yl)-[1,1'-binaphthalene]-2,2'-diol,

CAS No: 361342-50-9

#	Time	Type	Area	Height	Width	Area%	Symmetry
1	22.453	BB	5952.6	86.3	1.0849	100.000	0.774

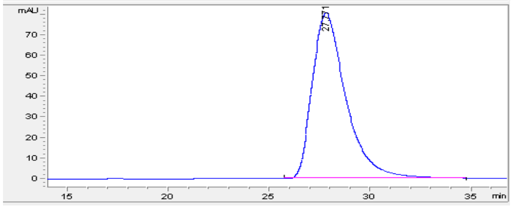

Recycled Ligand L1 *(R)*

(*R*)-3,3'-Di(anthracen-9-yl)-[1,1'-binaphthalene]-2,2'-diol

#	Time	Type	Area	Height	Width	Area%	Symmetry
1	27.771	BB	9323.3	80.4	1.743	100.000	0.645

Synthesis of cyclohexenes 4a-4k

(6-Methylcyclohex-1-en-1-yl)(phenyl)methanone, 4a

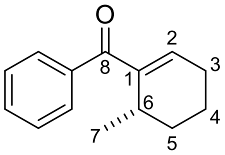

Racemic: 2-(2-Methyltetrahydro-2*H*-pyran-2-yl)-1-phenylethan-1-one **3a** (65.4 mg, 0.30 mmol, 1.0 equiv.), $Al(O^tBu)_3$ (73.9 mg, 0.30 mmol, 1.0 equiv.), and toluene (0.2 M, 1.5 mL) were subjected to **General Procedure 4**. The title compound was afforded after purification by FCC (pentane: Et_2O, 98:2) as a colourless oil (50.2 mg, 83% yield).

Enantioenriched: (*R*)-3,3'-Di(anthracen-9-yl)-[1,1'-binaphthalene]-2,2'-diol **L1** (128 mg, 0.20 mmol, 1.0 equiv.), *tert*-butanol (14.8 mg, 0.20 mmol, 1.0 equiv.), *m*-xylene (1.0 mL, 0.2 M), $AlMe_3$ in toluene (2.0 M, 0.1 mL, 0.20 mmol, 1.0 equiv.) and 2-(2-methyltetrahydro-2*H*-pyran-2-yl)-1-phenylethan-1-one **3a** (43.7 mg, 0.20 mmol, 1.0 equiv.) were subjected to **General Procedure 5A**. Purification by column chromatography (pentane:Et_2O, 98:2) afforded the title compound as a colourless oil (23.2 mg, 58% yield).

^{1}H NMR (400 MHz, $CDCl_3$) δ 7.73 – 7.62 (m, 2H, ArH), 7.55 – 7.47 (m, 1H, ArH), 7.45 – 7.37 (m, 2H, ArH), 6.40 (td, *J* = 4.0, 1.5 Hz, 1H, H-2), 3.09 – 2.88 (m, 1H, H-6), 2.37 – 2.05 (m, 2H, H-3), 1.86– 1.50 (m, 4H, H-4, H-5), 1.07 (d, *J* = 7.0 Hz, 3H, H-7).

^{13}C NMR (101 MHz, $CDCl_3$) δ 198.6 (C-8), 144.1 (C-1), 141.8 (C-2), 139.2 (ArC), 131.7 (ArC), 129.5 (ArC x 2), 128.2 (ArC x 2), 30.2 (C-5), 28.1 (C-6), 26.3 (C-3), 19.9 (C-7), 18.6 (C-4).

HRMS (ESI+): Found $[M+H]^+$ = 201.1273; $C_{14}H_{17}O$ requires 201.1274 Δ -0.45 ppm.

IR (film) ν_{max}/cm^{-1}: 2933, 2870, 1649, 1598, 1579, 1447.

$[\alpha]_D^{25}$ = +5.34 (c = 1.0, $CHCl_3$).

HPLC: Enantiomeric excess was determined by HPLC with a Chiralpak® IG column (99.5:0.5 hexane:IPA, 1.0 mL min^{-1}, 254 nm, room temperature); t_r (minor) = 21.7 min, t_r (major) = 24.9 min, 23:77 e.r.).

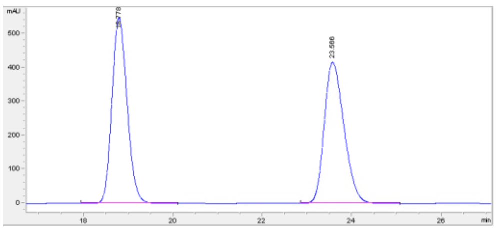

#	Time	Type	Area	Height	Width	Area%	Symmetry
1	18.778	BB	12977.7	549.7	0.3675	50.244	0.825
2	23.566	BB	12851.6	418.5	0.4782	49.756	0.709

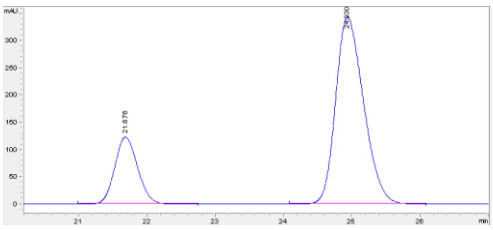

#	Time	Type	Area	Height	Width	Area%	Symmetry
1	21.676	BB	2887.8	123.7	0.3645	22.794	0.857
2	24.93	BB	9781.3	344	0.4398	77.206	0.701

(6-Methylcyclohex-1-en-1-yl)(4-(trifluoromethyl)phenyl)methanone, 4b

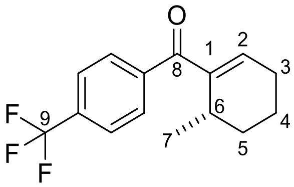

Racemic: 3b (57.3 mg, 0.20 mmol, 1.0 equiv.), Al(O^{t}Bu)$_3$ (49.3 mg, 0.20 mmol, 1.0 equiv.), and toluene (0.2 M, 1.0 mL) were subjected to **General Procedure 4**. Purification by column chromatography (pentane: Et$_2$O, 97:3) afforded the title compound as a colourless oil (38.9 mg, 72% yield).

Enantioenriched: (*R*)-3,3'-di(anthracen-9-yl)-[1,1'-binaphthalene]-2,2'-diol **L1** (128 mg, 0.20 mmol, 1.0 equiv.), *tert*-butanol (14.8 mg, 0.20 mmol, 1.0 equiv.), *m*-xylene (1.0 mL, 0.2 M), AlMe$_3$ in toluene (2.0 M, 0.1 mL, 0.20 mmol, 1.0 equiv.), and **3b** (57.3 mg, 0.20 mmol, 1.0 equiv.) were subjected to **General Procedure 5A**. Purification by column chromatography (pentane:Et$_2$O, 97:3) afforded the title compound as a colourless oil (35 mg, 66% yield).

^{1}H NMR (500 MHz, CDCl$_3$) δ 7.73 (d, *J* = 8.0 Hz, 2H, ArCH x 2), 7.67 (d, *J* = 8.0 Hz, 2H, ArCH x 2), 6.43 (td, *J* = 4.0, 1.0 Hz, 1H, H-2), 3.04 – 2.94 (m, 1H, H-6), 2.34 – 2.23 (m, 1H, H-3), 2.22 – 2.10 (m, 1H, H-3'), 1.83 – 1.68 (m, 2H, H-4), 1.68 – 1.54 (m, 2H, H-5), 1.09 (d, *J* = 7.0 Hz, 3H, H-7).

^{19}F NMR (471 MHz, CDCl$_3$) δ -63.0.

^{13}C NMR (126 MHz, CDCl$_3$) δ 197.1 (C-8), 144.0 (ArC), 143.9 (C-2), 142.4 (C-1), 133.1 (q, *J* = 33 Hz, ArC$_{ipso}$), 129.5 (ArC x 2), 125.2 (q, *J* = 4 Hz, ArC$_{ortho}$ x 2), 123.9 (q, *J* = 273 Hz, C-9), 29.9 (C-5), 27.8 (C-6), 26.5 (C-3), 19.9 (C-7), 18.2 (C-4).

HRMS (ESI+): Found [M+H]$^+$ = 269.1143; C$_{15}$H$_{16}$F$_3$O requires 269.1148 Δ -1.60 ppm.

IR (film) ν_{max}/cm^{-1}: 2937, 2873, 1654, 1579, 1512, 1457.

$[\alpha]_D^{25}$ = +4.84 (c = 1.0, CHCl$_3$).

HPLC: Enantiomeric excess was determined by HPLC with a Chiralpak® IC column (99.5:0.5 hexane:IPA, 1.0 mL min^{-1}, 230 nm, room temperature); t$_r$ (minor) = 8.5 min, t$_r$ (major) = 10.4 min, 14:86 e.r.).

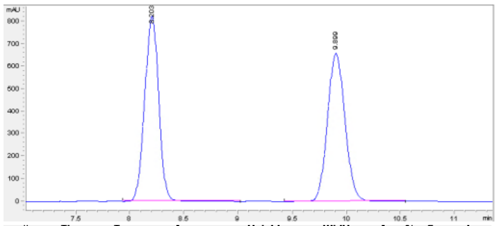

#	Time	Type	Area	Height	Width	Area%	Symmetry
1	8.203	BB	7478.8	825.5	0.1417	50.011	1.066
2	9.899	BB	7475.5	660.7	0.178	49.989	0.902

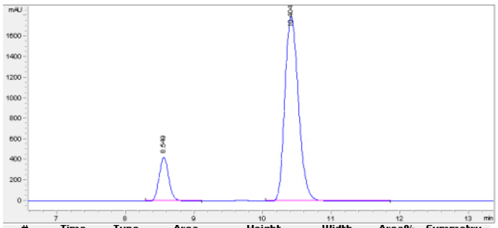

#	Time	Type	Area	Height	Width	Area%	Symmetry
1	8.549	BB	4158.6	425.9	0.1499	14.335	0.902
2	10.404	BB	24852.2	1800.4	0.215	85.665	0.798

(6-Butylcyclohex-1-en-1-yl)(phenyl)methanone, 4c

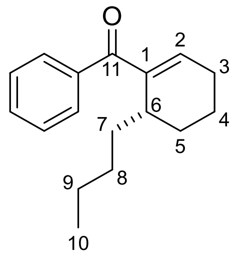

Racemic: 3c (52.1 mg, 0.20 mmol, 1.0 equiv.), Al(O^{t}Bu)$_3$ (49.3 mg, 0.20 mmol, 1.0 equiv.), and toluene (0.2 M, 1.0 mL) were subjected to **General Procedure 4**. The title compound was afforded after purification by FCC (pentane: Et_2O, 98:2) as a colourless oil (32.6 mg, 67% yield).

Enantioenriched: (*R*)-3,3'-Di(anthracen-9-yl)-[1,1'-binaphthalene]-2,2'-diol **L1** (128 mg, 0.20 mmol, 1.0 equiv.), *tert*-butanol (14.8 mg, 0.20 mmol, 1.0 equiv.), *m*-xylene (1.0 mL, 0.2 M), $AlMe_3$ in toluene (2.0 M, 0.1 mL, 0.20 mmol, 1.0 equiv.), and **3c** (52.1 mg, 0.20 mmol, 1.0 equiv.) were subjected to **General Procedure 5A**. Purification by column chromatography (pentane:Et_2O, 98:2) afforded the title compound **4c** as a colourless oil (31 mg, 64% yield).

^{1}H NMR (400 MHz, $CDCl_3$) δ 7.73 – 7.64 (m, 2H, ArH x 2), 7.54 – 7.46 (m, 1H, ArH), 7.46 – 7.36 (m, 2H, ArH x 2), 6.38 (td, *J* = 4.0, 1.0 Hz, 1H, H-2), 3.08 – 2.75 (m, 1H, H-6), 2.39 – 2.02 (m, 2H, H-3), 1.80 – 1.54 (m, 4H, H-4, H-5), 1.53 – 1.39 (m, 1H, H-7), 1.42 – 1.13 (m, 5H, H-7', H-8, H-9), 0.85 (t, *J* = 7.0 Hz, 3H, H-10).

^{13}C NMR (101 MHz, $CDCl_3$) δ 198.6 (C-11), 143.7 (C-1), 141.8 (C-2), 139.0 (ArC), 131.8 (ArC), 129.6 (ArC x 2), 128.2 (ArC x 2), 33.5 (C-7), 32.8 (C-6), 29.8 (C-9), 26.4 (C-5), 26.3 (C-3), 22.9 (C-8), 18.4 (C-4), 14.2 (C-10).

HRMS (ESI+): Found $[M+H]^+$ = 243.1741; $C_{17}H_{23}O$ requires 243.1743 Δ -0.82 ppm.

IR (film) v_{max}/cm^{-1}: 2980, 2970, 2931, 1650, 1598, 1579, 1447.

$[\alpha]_D^{25}$ = +9.80 (c = 1.0, $CHCl_3$).

HPLC: Enantiomeric excess was determined by HPLC with a Chiralpak® IG column (99.5:0.5 hexane:IPA, 1.0 mL min^{-1}, 254 nm, room temperature); t_r (minor) = 15.6 min, t_r (major) = 19.3 min, 4:96 e.r.).

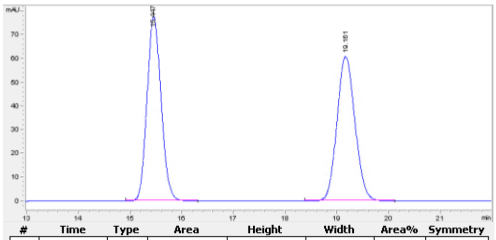

#	Time	Type	Area	Height	Width	Area%	Symmetry
1	15.447	BB	1482.7	78	0.2937	49.983	0.863
2	19.161	BB	1483.7	61	0.3776	50.017	0.881

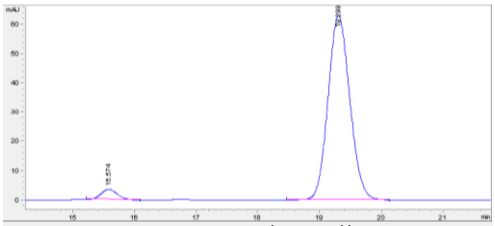

#	Time	Type	Area	Height	Width	Area%	Symmetry
1	15.574	BB	64.7	3.5	0.2839	4.032	0.859
2	19.299	BB	1540.9	63	0.3796	95.968	0.882

Enantioenriched: (*R*)- 3,3'-di(anthracen-9-yl)-[1,1'-binaphthalene]-2,2'-diol **L1** (77 mg, 0.12 mmol, 0.6 equiv.), toluene (0.2 M, 1 mL), $AlMe_3$ in toluene (1.0 M, 0.08 mL, 0.08 mmol, 0.4 equiv.) and **3c** (52.1 mg, 0.20 mmol, 1.0

equiv.) were subjected to **General procedure 5C.** Purification by column chromatography (pentane:Et_2O, 98:2) afforded the title compound **4c** as a colourless oil (30 mg, 62% yield).

HPLC: Enantiomeric excess was determined by HPLC with a Chiralpak® IG column (99.5:0.5 hexane:IPA, 1.0 mL min^{-1}, 254 nm, room temperature); t_r (minor) = 13.1 min, t_r (major) = 15.1 min, 11:89 e.r.).

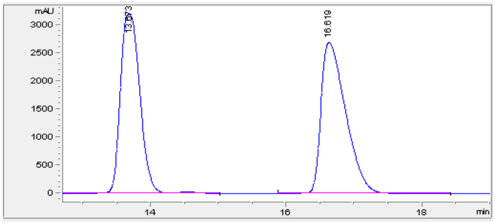

#	Time	Type	Area	Height	Width	Area%	Symmetry
1	13.673	BV R	63870.7	3193.1	0.3135	48.533	0.762
2	16.619	BB	67732.2	2700.7	0.3866	51.467	0.449

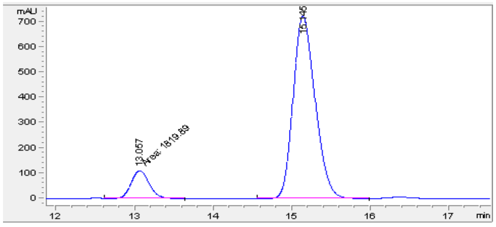

#	Time	Type	Area	Height	Width	Area%	Symmetry
1	13.057	MM T	1819.9	111.2	0.3502	11.385	3.08E-2
2	15.145	BB	14164.7	721.6	0.305	88.615	0.753

Enantioenriched: (*R*)- 3,3'-di(anthracen-9-yl)-[1,1'-binaphthalene]-2,2'-diol **L1** (77 mg, 0.12 mmol, 0.6 equiv.), toluene (0.2 M, 1 mL), $AlMe_3$ in toluene (1.0 M, 0.08 mL, 0.08 mmol, 0.4 equiv.) after which dry THF (60 µL, 0.73 mmol, 9.25 equiv.) and **3c** (52.1 mg, 0.20 mmol, 1.0 equiv.) were subjected to **General procedure 5C.** Purification by column chromatography (pentane:Et_2O, 98:2) afforded the title compound **4c** as a colourless oil (29 mg, 60% yield).

HPLC: Enantiomeric excess was determined by HPLC with a Chiralpak® IG column (99.5:0.5 hexane:IPA, 1.0 mL min^{-1}, 254 nm, room temperature); t_r (minor) = 13.7 min, t_r (major) = 16.9 min, 8.5:91.5 e.r.).

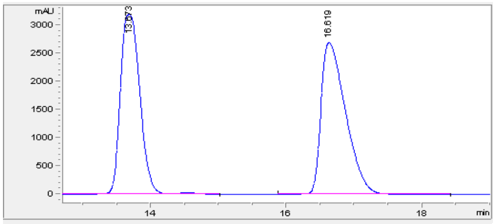

#	Time	Type	Area	Height	Width	Area%	Symmetry
1	13.673	BV R	63870.7	3193.1	0.3135	48.533	0.762
2	16.619	BB	67732.2	2700.7	0.3866	51.467	0.449

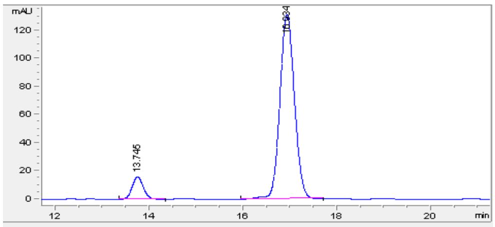

#	Time	Type	Area	Height	Width	Area%	Symmetry
1	13.745	BB	262.6	15.8	0.2581	8.493	0.901
2	16.934	BB	2829.8	130.7	0.3366	91.507	0.918

(6-Butylcyclohex-1-en-1-yl)(4-methoxyphenyl)methanone, 4d

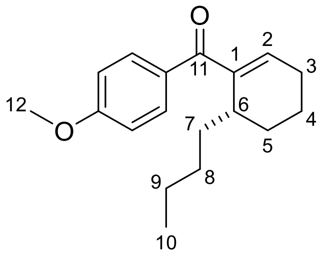

Racemic: 3d (58.1 mg, 0.20 mmol, 1.0 equiv.), Al(O^{t}Bu)$_3$ (49.3 mg, 0.20 mmol, 1.0 equiv.), and toluene (0.2 M, 1.0 mL) were subjected to **General Procedure 4**. Purification by column chromatography (Pentane:Et_2O, 98:2) afforded the title compound **4d** as a colourless oil (19.5 mg, 36% yield).

Enantioenriched: (*R*)-3,3'-di(anthracen-9-yl)-[1,1'-binaphthalene]-2,2'-diol **L1** (128 mg, 0.20 mmol, 1.0 equiv.), *tert*-butanol (14.8 mg, 0.20 mmol, 1.0 equiv.), *m*-xylene (1.0 mL, 0.2 M), $AlMe_3$ in toluene (2.0 M, 0.1 mL, 0.20 mmol, 1.0 equiv.), and **3d** (58.1 mg, 0.20 mmol, 1.0 equiv.) were subjected to **General Procedure 5A**. Purification by column chromatography (Pentane:Et_2O, 98:2) afforded the title compound **4d** as a colourless oil (34.9 mg, 64% yield).

^{1}H NMR (400 MHz, $CDCl_3$) δ 7.78 – 7.70 (m, 2H, ArH x 2), 6.95 – 6.87 (m, 2H, ArH x 2), 6.29 (td, *J* = 4.0, 1.0 Hz, 1H, H-2), 3.86 (s, 3H, H-12), 2.93 – 2.84 (m, 1H, H-6), 2.35 – 2.06 (m, 2H, H-3), 1.79 – 1.53 (m, 4H, H-4 x 2, H-5), 1.49 – 1.16 (m, 6H, H-7, H-8, H-9), 0.83 (t, *J* = 6.5 Hz, 3H, H-10).

^{13}C NMR (101 MHz, $CDCl_3$) δ 197.6 (C-11), 162.9 (ArC), 143.7 (C-1), 139.2 (C-2), 132.0 (ArC x 2), 131.4 (ArC), 113.5 (ArC x 2), 55.5 (C-12), 33.6 (C-7), 33.3 (C-6), 29.8 (C-8), 26.7 (C-5), 26.1 (C-3), 22.9 (C-9), 18.7 (C-4), 14.2 (C-10).

HRMS (ESI+): Found $[M+H]^+$ = 273.1845; $C_{18}H_{25}O_2$ requires 273.1849 Δ -1.47 ppm.

IR (film) v_{max}/cm^{-1}: 2932, 2858, 1643, 1600, 1575, 1509.

$[\alpha]_D^{25}$ = +8.08 (c = 1.0, $CHCl_3$).

HPLC: Enantiomeric excess was determined by HPLC with a Chiralpak® IA column (99.5:0.5 hexane:IPA, 0.5 mL min^{-1}, 254 nm, room temperature); t_r (minor) = 24.7 min, t_r (major) = 26.6 min, 6:94 e.r.).

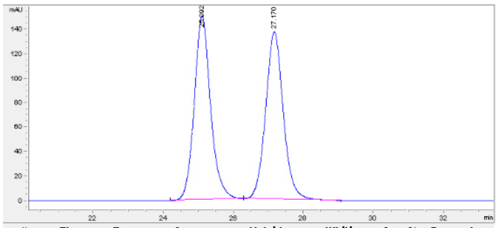

#	Time	Type	Area	Height	Width	Area%	Symmetry
1	25.092	BB	4877	150.4	0.4902	50.404	0.868
2	27.17	BB	4798.8	136.8	0.5272	49.596	0.92

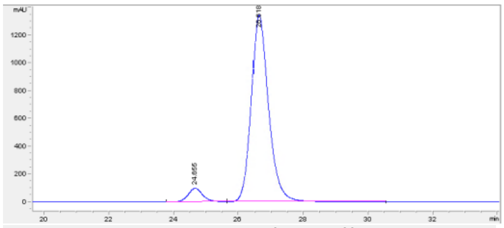

#	Time	Type	Area	Height	Width	Area%	Symmetry
1	24.655	BB	3129.4	98.4	0.483	5.995	0.899
2	26.618	BB	49071.4	1340.7	0.545	94.005	0.758

Enantioenriched: (*R*)- 3,3'-di(anthracen-9-yl)-[1,1'-binaphthalene]-2,2'-diol **L1** (77 mg, 0.12 mmol, 0.6 equiv.), toluene (0.2 M, 1 mL), $AlMe_3$ in toluene (1.0 M, 0.08 mL, 0.08 mmol, 0.4 equiv.) and **3d** (58.1 mg, 0.20 mmol, 1.0 equiv.) were subjected to **General procedure 5B.** Purification by column chromatography (Pentane:Et_2O, 98:2) afforded the title compound **4d** as a colourless oil (34 mg, 62% yield).

HPLC: Enantiomeric excess was determined by HPLC with a Chiralpak® IA column (99:1 hexane:IPA, 0.5 mL min^{-1}, 254 nm, room temperature); t_r (minor) = 24.7 min, t_r (major) = 26.6 min, 6:94 e.r.).

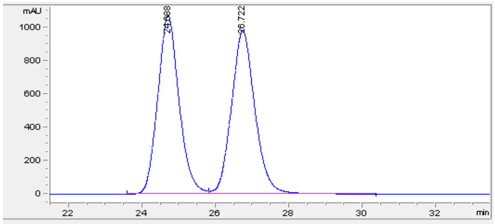

#	Time	Type	Area	Height	Width	Area%	Symmetry
1	24.688	BV	43157.3	1070	0.6025	49.790	0.872
2	26.722	VB	43520.6	979.6	0.6603	50.210	0.865

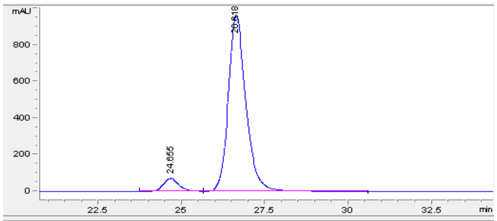

#	Time	Type	Area	Height	Width	Area%	Symmetry
1	24.655	BB	2252.4	70.9	0.4805	5.999	0.903
2	26.618	BB	35294.8	963.5	0.5434	94.001	0.758

Enantioenriched: (*R*)- 3,3'-di(anthracen-9-yl)-[1,1'-binaphthalene]-2,2'-diol **L1** (77 mg, 0.12 mmol, 0.6 equiv.), toluene (0.2 M, 1 mL), $AlMe_3$ in toluene (1.0 M, 0.08 mL, 0.08 mmol, 0.4 equiv.) after which dry THF (60 µL, 0.73

mmol, 9.25 equiv.) and **3d** (58.1 mg, 0.20 mmol, 1.0 equiv.) were subjected to **General procedure 5C.** Purification by column chromatography (Pentane:Et_2O, 98:2) afforded the title compound **4d** as a colourless oil (35 mg, 64% yield).

HPLC: Enantiomeric excess was determined by HPLC with a Chiralpak® IA column (99:1 hexane:IPA, 0.5 mL min^{-1}, 254 nm, room temperature); t_r (minor) = 24.7 min, t_r (major) = 26.6 min, 5.5:94.5 e.r.).

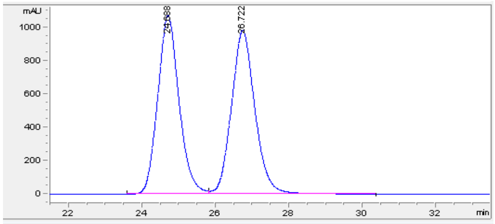

#	Time	Type	Area	Height	Width	Area%	Symmetry
1	24.688	BV	43157.3	1070	0.6025	49.790	0.872
2	26.722	VB	43520.6	979.6	0.6603	50.210	0.865

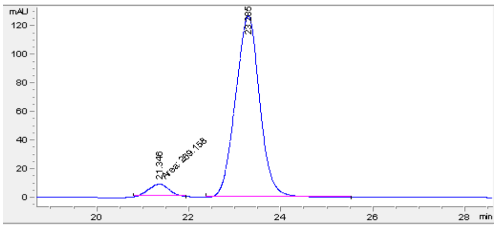

#	Time	Type	Area	Height	Width	Area%	Symmetry
1	21.346	MM T	269.2	8.7	0.5163	5.445	1.036
2	23.285	BB	4674	127.1	0.5672	94.555	1.053

(6-Butylcyclohex-1-en-1-yl)(4-(trifluoromethyl)phenyl)methanone, 4e

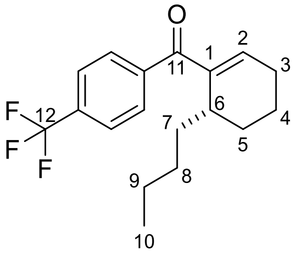

Racemic: 3e (65.7 mg, 0.20 mmol, 1.0 equiv.), $Al(O^tBu)_3$ (49.3 mg, 0.20 mmol, 1.0 equiv.), and toluene (0.2 M, 1.0 mL) were subjected to **General Procedure 4**. Purification by column chromatography (Pentane:Et_2O, 98:2) afforded the title compound **4e** as a colourless oil (40.6 mg, 65% yield).

Enantioenriched: (*R*)-3,3'-di(anthracen-9-yl)-[1,1'-binaphthalene]-2,2'-diol **L1** (128 mg, 0.20 mmol, 1.0 equiv.), *tert*-butanol (14.8 mg, 0.20 mmol, 1.0 equiv.), *m*-xylene (1.0 mL, 0.2 M), $AlMe_3$ in toluene (2.0 M, 0.1 mL, 0.20 mmol, 1.0 equiv.), and **3e** (65.7 mg, 0.20 mmol, 1.0 equiv.) were subjected to **General Procedure 5A**. Purification by column chromatography (Pentane:Et_2O, 98:2) afforded the title compound **4e** as a colourless oil (37.2 mg, 60% yield).

^{1}H NMR (500 MHz, $CDCl_3$) δ 7.75 (d, *J* = 8.0 Hz, 2H), 7.67 (d, *J* = 8.1 Hz, 2H), 6.43 – 6.38 (m, 1H), 2.94 – 2.86 (m, 1H), 2.34 – 2.23 (m, 1H), 2.21 – 2.08 (m, 1H), 1.79 – 1.56 (m, 4H), 1.53 – 1.42 (m, 1H), 1.41 – 1.17 (m, 4H), 0.91 – 0.81 (m, 3H).

^{19}F NMR (471 MHz, $CDCl_3$) δ -63.0.

^{13}C NMR (126 MHz, $CDCl_3$) δ 197.1 (C-11), 143.7 (ArC), 143.7 (C-2), 142.3 (C-1), 133.1 (q, *J* = 32 Hz, ArC_{ipso}), 129.6 (ArC x 2), 125.2 (q, *J* = 4 Hz, ArC x 2), 123.9 (q, *J* = 272 Hz, C-12), 33.4 (C-7), 32.5 (C-6), 29.8 (C-8), 26.4 (C-3), 26.2 (C-5), 22.9 (C-9), 18.1 (C-4), 14.2 (C-10).

HRMS (ESI+): Found $[M+H]^+$ = 311.1612; $C_{18}H_{22}F_3O$ requires 311.1617 Δ -1.72 ppm.

IR (film) ν_{max}/cm^{-1}: 2935, 2861, 1655, 1510, 1459, 1407.

$[\alpha]_D^{25}$ = +8.16 (c = 1.0, $CHCl_3$).

HPLC: Enantiomeric excess was determined by HPLC with a Chiralpak® IC column (99.5:0.5 hexane:IPA, 1.0 mL min^{-1}, 254 nm, room temperature); t_r (minor) = 9.5 min, t_r (major) = 11.8 min, 3.5:96.5 e.r.).

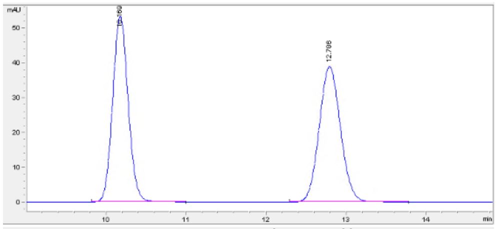

#	Time	Type	Area	Height	Width	Area%	Symmetry
1	10.169	BB	724.7	53.4	0.2122	49.876	0.901
2	12.786	BB	728.3	39.1	0.2916	50.124	0.897

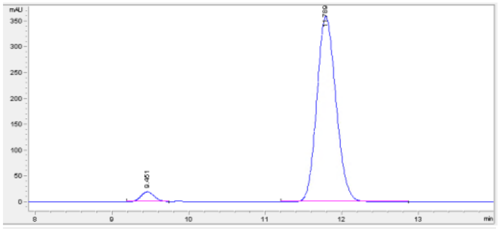

#	Time	Type	Area	Height	Width	Area%	Symmetry
1	9.451	BB	231.6	19	0.1902	3.564	0.908
2	11.789	BB	6268	360.4	0.2709	96.436	0.841

Enantioenriched: (*R*)- 3,3'-di(anthracen-9-yl)-[1,1'-binaphthalene]-2,2'-diol **L1** (77 mg, 0.12 mmol, 0.6 equiv.), toluene (0.2 M, 1 mL), $AlMe_3$ in toluene (1.0 M, 0.08 mL, 0.08 mmol, 0.4 equiv.) and **3e** (65.7 mg, 0.20 mmol, 1.0 equiv.) were subjected to **General procedure 5B.** Purification by column chromatography (Pentane:Et_2O, 98:2) afforded the title compound **4e** as a colourless oil (40 mg, 64% yield).

HPLC: Enantiomeric excess was determined by HPLC with a Chiralpak® IG column (99.5:0.5 hexane:IPA, 1.0 mL min^{-1}, 254 nm, room temperature); t_r (minor) = 7.1 min, t_r (major) = 7.9 min, 11:89 e.r.).

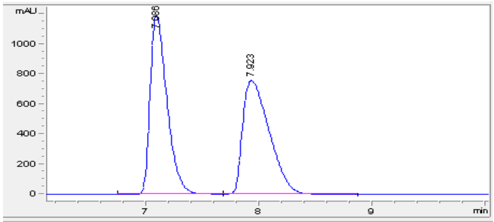

#	Time	Type	Area	Height	Width	Area%	Symmetry
1	7.086	BB	12230.4	1189.1	0.158	49.954	0.538
2	7.923	BB	12253	755.2	0.2573	50.046	0.452

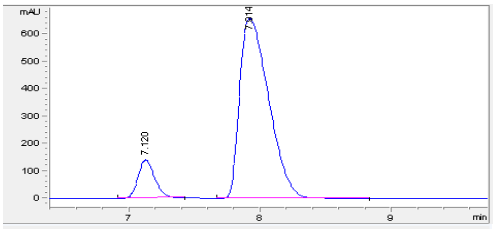

#	Time	Type	Area	Height	Width	Area%	Symmetry
1	7.12	BB	1268.4	141.8	0.1384	11.045	0.786
2	7.914	BB	10215.5	664.3	0.2454	88.955	0.506

Enantioenriched: (*R*)- 3,3'-di(anthracen-9-yl)-[1,1'-binaphthalene]-2,2'-diol **L1** (77 mg, 0.12 mmol, 0.6 equiv.), toluene (0.2 M, 1 mL), $AlMe_3$ in toluene (1.0 M, 0.08 mL, 0.08 mmol, 0.4 equiv.) after which dry THF (60 µL, 0.73 mmol, 9 equiv.) and **3e** (65.7 mg, 0.20 mmol, 1.0 equiv.) were subjected to **General procedure 5C.** Purification by column chromatography (Pentane:Et_2O, 98:2) afforded the title compound **4e** as a colourless oil (38 mg, 61% yield).

HPLC: Enantiomeric excess was determined by HPLC with a Chiralpak® IG column (99.5:0.5 hexane:IPA, 1.0 mL min^{-1}, 254 nm, room temperature); t_r (minor) = 8.2 min, t_r (major) = 10.1 min, 10:90 e.r.).

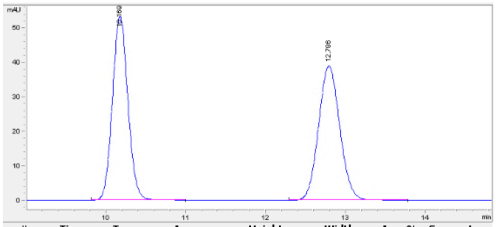

#	Time	Type	Area	Height	Width	Area%	Symmetry
1	10.169	BB	724.7	53.4	0.2122	49.876	0.901
2	12.786	BB	728.3	39.1	0.2916	50.124	0.897

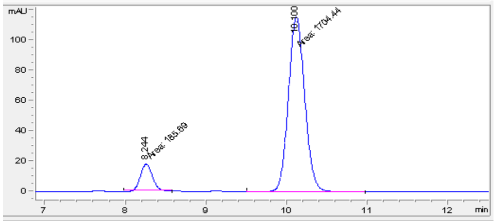

#	Time	Type	Area	Height	Width	Area%	Symmetry
1	8.244	MM T	185.7	17.9	0.1726	9.824	0.865
2	10.1	MM T	1704.4	116.9	0.243	90.176	3.59E-3

(6-(*tert*-Butyl)cyclohex-1-en-1-yl)(phenyl)methanone, 4f

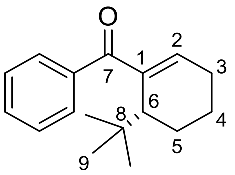

Racemic: **3f** (78.1 mg, 0.30 mmol, 1.0 equiv.), Al(O^{t}Bu)$_3$ (73.9 mg, 0.30 mmol, 1.0 equiv.), and toluene (0.2 M, 1.5 mL). were subjected to **General Procedure 4**. The title compound **4f** was afforded after purification by FCC (Pentane:Et_2O, 97:3) as a colourless oil (47.0 mg, 65% yield).

Enantioenriched: (*R*)-3,3'-di(anthracen-9-yl)-[1,1'-binaphthalene]-2,2'-diol **L1** (128 mg, 0.20 mmol, 1.0 equiv.), *tert*-butanol (14.8 mg, 0.20 mmol, 1.0 equiv.), *m*-xylene (1.0 mL, 0.2 M), $AlMe_3$ in toluene (2.0 M, 0.1 mL, 0.20 mmol, 1.0 equiv.), and **3f** (52.1 mg, 0.30 mmol, 1.0 equiv.) were subjected to **General Procedure 5A**. Purification by column chromatography (Pentane:Et_2O, 98:2) afforded the title compound **4f** as a colourless oil (29.6 mg, 61% yield).

^{1}H NMR (400 MHz, $CDCl_3$) δ 7.87 – 7.81 (m, 2H, ArH), 7.57 – 7.49 (m, 1H, ArH), 7.48 – 7.39 (m, 2H, ArH), 6.44 (ddd, *J* = 4.5, 3.5, 1.0 Hz, 1H, H-2), 3.09 – 2.95 (m, 1H, H-6), 2.32 – 2.05 (m, 2H, H-3), 2.00 – 1.78 (m, 2H, H-4, H-5), 1.77 – 1.60 (m, 1H, H-5), 1.57 – 1.47 (m, 1H, H-4), 0.93 (s, 9H, H-9).

^{13}C NMR (101 MHz, $CDCl_3$) δ 198.9 (C-7), 142.5 (C-1), 142.0 (C-2), 137.7 (ArC), 132.2 (ArC), 130.2 (ArC x 2), 128.3 (ArC x 2), 41.9 (C-6), 35.9 (C-8), 29.5 (C-9 x 3), 25.5 (C-3), 24.5 (C-5), 20.3 (C-4).

HRMS (ESI+): Found $[M+H]^+$ = 243.1741; $C_{17}H_{23}O$ requires 243.1743 Δ -0.82 ppm.

IR (film) ν_{max}/cm^{-1}: 2980, 1655, 1598, 1579, 1448.

$[\alpha]_D^{25}$ = −8.20 (c = 1.0, $CHCl_3$).

HPLC: Enantiomeric excess was determined by HPLC with a Chiralpak® OD column (99.5:0.5 hexane:IPA, 1.0 mL min^{-1}, 230 nm, room temperature); t_r (major) = 6.1 min, t_r (minor) = 8.0 min, 96.5:3.5 e.r.).

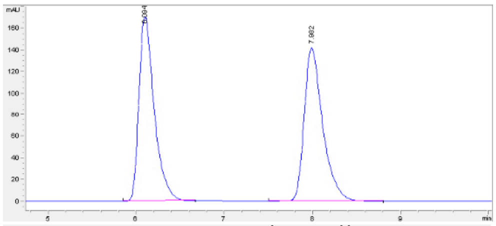

#	Time	Type	Area	Height	Width	Area%	Symmetry
1	6.094	BB	2068	171.4	0.1808	49.901	0.6
2	7.982	BB	2076.2	142.3	0.2182	50.099	0.634

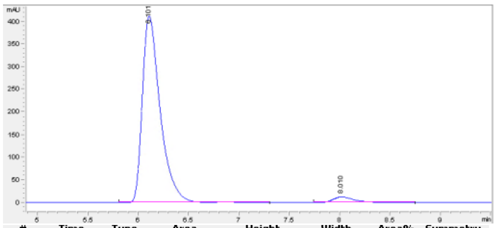

#	Time	Type	Area	Height	Width	Area%	Symmetry
1	6.101	BV R	5136.3	413.4	0.1883	96.587	0.578
2	8.01	BB	181.5	12.6	0.2167	3.413	0.631

(6-Butylcyclohex-1-en-1-yl)(2,3,4,5,6-pentamethylphenyl)methanone, 4g

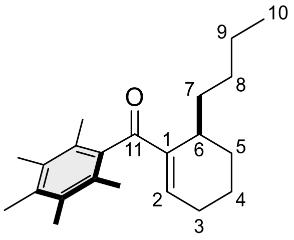

Racemic: 3g (66.1 mg, 0.20 mmol, 1.0 equiv.), Al(O^{t}Bu)$_3$ (49.3 mg, 0.20 mmol, 1.0 equiv.), and toluene (0.2 M, 1.0 mL) were subjected to **General Procedure 4**. The title compound was afforded after purification by FCC (Pentane:Et$_2$O, 98:2) as a white solid (45.9 mg, 73% yield).

Enantioenriched: (*R*)-3,3'-di(anthracen-9-yl)-[1,1'-binaphthalene]-2,2'-diol **L1** (128 mg, 0.20 mmol, 1.0 equiv.), *tert*-butanol (14.8 mg, 0.20 mmol, 1.0 equiv.), *m*-xylene (1.0 mL, 0.2 M), AlMe$_3$ in toluene (2.0 M, 0.1 mL, 0.20 mmol, 1.0 equiv.), and **3g** (66.1 mg, 0.20 mmol, 1.0 equiv.) were subjected to **General Procedure 5A**. Purification by column chromatography (Pentane:Et$_2$O, 98:2) afforded the title compound **4g** as a white solid (45 mg, 72% yield).

^{1}H NMR (400 MHz, CDCl$_3$) δ 6.46 (t, *J* = 4.0 Hz, 1H, H-2), 2.90 – 2.80 (m, 1H, H-6), 2.24 (s, 3H, ArCH$_3$), 2.21 – 2.08 (m, 8H, 2xArCH$_3$, H-3), 2.04 (s, 3H, 3×ArCH$_3$), 2.02 (s, 3H, 3×ArCH$_3$), 1.92 – 1.71 (m, 2H), 1.70 – 1.55 (m, 2H), 1.55 – 1.22 (m, 6H), 0.94 (t, *J* = 7.0 Hz, 3H, H-10).

^{13}C NMR (101 MHz, CDCl$_3$) δ 202.9 (C-11), 146.1 (C-2), 145.1 (C1), 138.4 (ArC), 134.9 (ArC), 132.6 (ArC), 132.6 (ArC), 129.4 (ArC), 129.2 (ArC), 32.7 (C$_7$), 31.1 (C-6), 30.2 (C-5), 26.6 (C-3), 25.0 (C-8), 22.9 (C-9), 17.5 (ArCH$_3$), 17.3 (ArCH$_3$), 17.1 (C-4), 16.8 (ArCH$_3$), 16.1 (ArCH$_3$), 16.0 (ArCH$_3$), 14.3 (C-10).

HRMS (ESI+): Found [M+H]$^+$ = 313.2521; $C_{22}H_{33}O$ requires 313.2526 Δ -1.55 ppm.

IR (film) ν_{max}/cm^{-1}: 2928, 2859, 1650, 1631, 1601, 1456.

$[\alpha]_D^{25}$ = −8.38 (c = 1.0, CHCl$_3$).

m.p.: 58-60 °C.

HPLC: Enantiomeric excess was determined by HPLC with a Chiralpak® IG column (99.5:0.5 hexane:IPA, 1.0 mL min^{-1}, 210 nm, room temperature); t$_r$ (major) = 25.1 min, t$_r$ (minor) = 32.9 min, 96.5:3.5 e.r.).

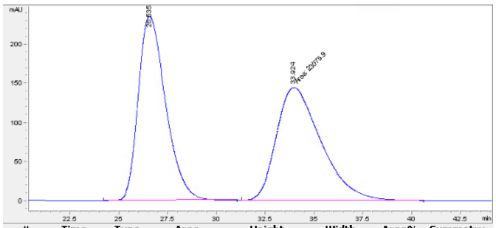

#	Time	Type	Area	Height	Width	Area%	Symmetry
1	26.535	BB	22989.6	236.2	1.4637	49.906	0.641
2	33.924	MM	23075.9	144	2.6703	50.094	0.618

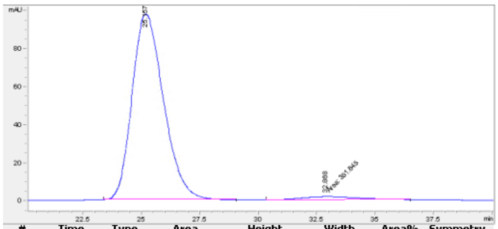

#	Time	Type	Area	Height	Width	Area%	Symmetry
1	25.157	BB	9106	97.3	1.3943	96.282	0.734
2	32.868	MM	351.6	1.8	3.1914	3.718	0.687

Enantioenriched: (*R*)- 3,3'-di(anthracen-9-yl)-[1,1'-binaphthalene]-2,2'-diol **L1** (77 mg, 0.12 mmol, 0.6 equiv.), toluene (0.2 M, 1 mL), AlMe$_3$ in toluene (1.0 M, 0.08 mL, 0.08 mmol, 0.4 equiv.) and **3g** (66 mg, 0.20 mmol, 1 equiv.) were subjected to **General procedure 5B.** Purification by column chromatography (Pentane:Et$_2$O, 98:2) afforded the title compound **4g** as a white solid (46 mg, 74% yield).

HPLC: Enantiomeric excess was determined by HPLC with a Chiralpak® IG column (99.5:0.5 hexane:IPA, 1.0 mL min^{-1}, 210 nm, room temperature); t$_r$ (major) = 25.6 min, t$_r$ (minor) = 34.6 min, 97:3 e.r.).

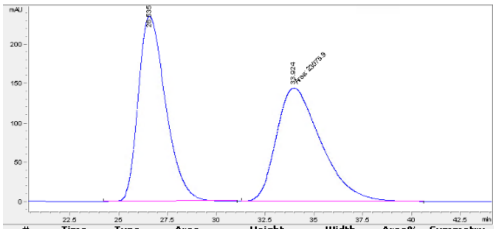

#	Time	Type	Area	Height	Width	Area%	Symmetry
1	26.535	BB	22989.6	236.2	1.4637	49.906	0.641
2	33.924	MM	23075.9	144	2.6703	50.094	0.618

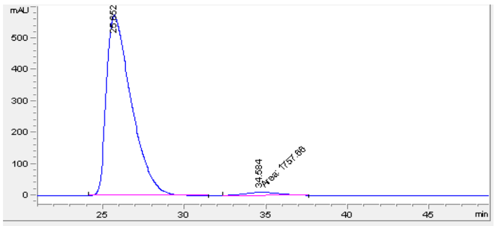

#	Time	Type	Area	Height	Width	Area%	Symmetry
1	25.652	BB	60537.9	573.9	1.5586	97.179	0.422
2	34.584	MM T	1757.7	10.7	2.7392	2.821	0.881

Enantioenriched: (*R*)- 3,3'-di(anthracen-9-yl)-[1,1'-binaphthalene]-2,2'-diol **L1** (77 mg, 0.12 mmol, 0.6 equiv.), toluene (0.2 M, 1 mL), $AlMe_3$ in toluene (1.0 M, 0.08 mL, 0.08 mmol, 0.4 equiv.), after which dry THF (60 µL, 0.73 mmol, 9.25 equiv.) and **3g** (66 mg, 0.20 mmol, 1 equiv.) were subjected to **General procedure 5C.** Purification by column chromatography (Pentane:Et_2O, 98:2) afforded the title compound **4g** as a white solid (47 mg, 75% yield).

HPLC: Enantiomeric excess was determined by HPLC with a Chiralpak® IG column (99.5:0.5 hexane:IPA, 1.0 mL min^{-1}, 210 nm, room temperature); t_r (major) = 26.6 min, t_r (minor) = 35.1 min, 98.5:1.5 e.r.).

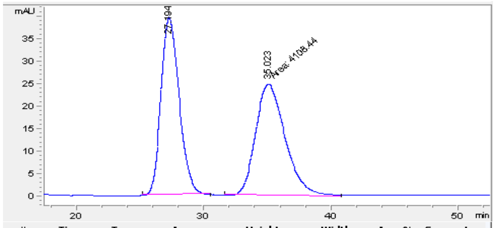

#	Time	Type	Area	Height	Width	Area%	Symmetry
1	27.194	BB	3982.2	39.8	1.4017	49.220	0.762
2	35.023	MM T	4108.4	24.9	2.7477	50.780	2.93E-5

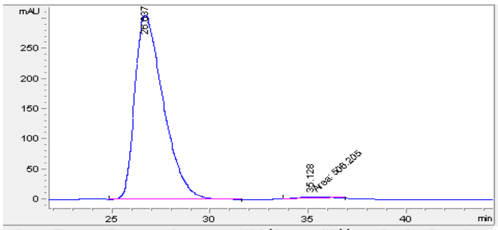

#	Time	Type	Area	Height	Width	Area%	Symmetry
1	26.637	BB	30906.5	305.3	1.521	98.389	0.535
2	35.128	MM T	506.2	3.9	2.1459	1.611	1.014

Scale-Up Procedure: (*R*)- 3,3'-di(anthracen-9-yl)-[1,1'-binaphthalene]-2,2'-diol **L1** (1.90 g, 3.0 mmol, 0.6 equiv.), toluene (0.2 M, 25 mL), $AlMe_3$ in toluene (1.0 M, 2.0 mL, 2.0 mmol, 0.4 equiv.), after which dry THF (1.50 mL, 18.5 mmol, 9.25 equiv.) and **3g** (1.65 g, 5.00 mmol, 1.0 equiv.) were subjected to **General procedure 5C.** Purification by column chromatography (Pentane:Et_2O, 98:2) afforded the title compound **4g** as a white solid (1.25 g, 80% yield). (*R*)- 3,3'-di(anthracen-9-yl)-[1,1'-binaphthalene]-2,2'-diol **L1** was recycled and recovered after column chromatography (Pentane:Et_2O, 60:40) as an off-white solid (1.85 g, 97% yield) and RSM is (183 mg, 11% yield).

The spectroscopic data were identical to that given above.

HPLC: Enantiomeric excess was determined by HPLC with a Chiralpak® IG column (99.5:0.5 hexane:IPA, 1.0 mL min^{-1}, 210 nm, room temperature); t_r (major) = 27.4 min, t_r (minor) = 35.4 min, 98:2 e.r.).

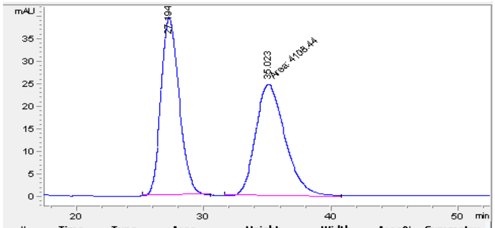

#	Time	Type	Area	Height	Width	Area%	Symmetry
1	27.194	BB	3982.2	39.8	1.4017	49.220	0.762
2	35.023	MM T	4108.4	24.9	2.7477	50.780	2.93E-5

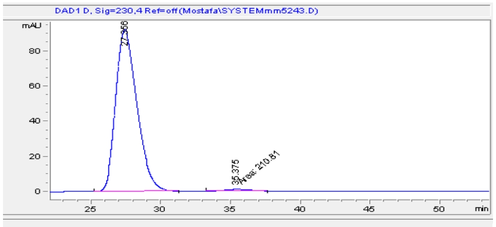

#	Time	Type	Area	Height	Width	Area%	Symmetry
1	27.356	BB	9824.7	92	1.5698	97.899	0.697
2	35.375	MM T	210.8	1.3	2.6504	2.101	1.57E-2

Control experiment: enantioenriched (6-butylcyclohex-1-en-1-yl)(2,3,4,5,6-pentamethylphenyl)methanone (63 mg, 0.20 mmol, 1.0 equiv.) was subjected to **General procedure 5B** and recovered as a white solid **4g** (44 mg, 70% yield).

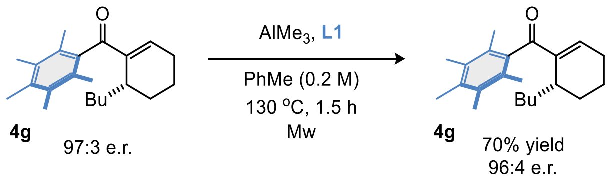

HPLC: Enantiomeric excess was determined by HPLC with a Chiralpak® IG column (99.5:0.5 hexane:IPA, 1.0 mL min^{-1}, 210 nm, room temperature); t_r (major) = 19.7 min, t_r (minor) = 25.5 min, 96:4 e.r.).

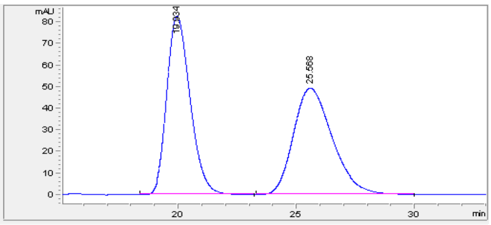

#	Time	Type	Area	Height	Width	Area%	Symmetry
1	19.934	BB	5664.1	82.6	1.0603	50.264	0.748
2	25.568	BB	5604.6	49.1	1.6492	49.736	0.722

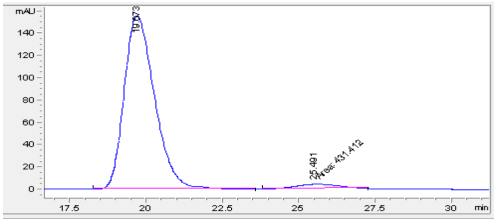

#	Time	Type	Area	Height	Width	Area%	Symmetry
1	19.673	BB	10770.9	155.4	1.0665	96.149	0.689
2	25.491	MM T	431.4	4.1	1.7299	3.851	3.19E-2

(6-Butylcyclohex-1-en-1-yl)(mesityl)methanone, 4h

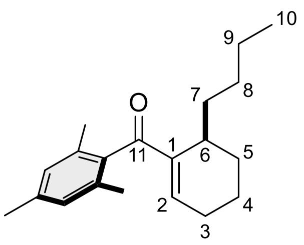

Racemic: 3h (60.5 mg, 0.20 mmol, 1.0 equiv.), Al(O^{t}Bu)$_3$ (49.3 mg, 0.20 mmol, 1.0 equiv.), and toluene (0.2 M, 1.0 mL) were subjected to **General Procedure 4**. The title compound was afforded after purification by FCC (Pentane:Et_2O, 98:2) as a colourless oil **4h** (37.0 mg, 65% yield).

Enantioenriched: (*R*)-3,3'-di(anthracen-9-yl)-[1,1'-binaphthalene]-2,2'-diol **L1** (128 mg, 0.20 mmol, 1.0 equiv.), *tert*-butanol (14.8 mg, 0.20 mmol, 1.0 equiv.), *m*-xylene (1.0 mL, 0.2 M), $AlMe_3$ in toluene (2.0 M, 0.1 mL, 0.20 mmol, 1.0 equiv.), and **3h** (60.5 mg, 0.20 mmol, 1.0 equiv.) were subjected to **General Procedure 5A**. Purification by column chromatography (Pentane:Et_2O, 98:2) afforded the title compound **4h** as a colourless oil (50.5 mg, 89% yield).

^{1}H NMR (400 MHz, $CDCl_3$) δ 6.81 (s, 2H, ArH), 6.45 (t, *J* = 3.9 Hz, 1H, H-2), 2.90 – 2.78 (m, 1H, H-6), 2.28 (s, 3H, 3×$ArCH_3$), 2.22 – 2.12 (m, 2H), 2.10 (s, 6H, 2x$ArCH_3$), 1.88-1.78 (m, 1H), 1.74 – 1.57 (m, 3H), 1.54 – 1.36 (m, 4H), 1.35 – 1.29 (m, 1H), 1.25 – 1.19 (m, 1H), 0.93 (t, *J* = 6.8 Hz, 3H, H-10).

^{13}C NMR (101 MHz, $CDCl_3$) δ 201.7 (C-11), 145.7 (C-2), 145.0 (C1), 137.8 (ArC), 137.8 (ArC), 134.3 (ArC), 128.2 (ArC), 32.8, 31.2, 30.2, 26.6, 25.09, 22.9, 21.2, 19.3 ($ArCH_3$), 17.2 ($ArCH_3$), 14.3 (C-10).

HRMS (ESI+): Found $[M+Na]^+$ = 307.2046; $C_{20}H_{28}ONa$ requires 307.2032 Δ 4.44 ppm.

IR (film) ν_{max}/cm^{-1}: 2926, 2855, 1654, 1629, 1612, 1457, 1262.

$[\alpha]_D^{20}$ = -66.6 (c = 0.4, $CHCl_3$).

HPLC: Enantiomeric excess was determined by HPLC with a Chiralpak® IG column (99.5:0.5 hexane:IPA, 1.0 mL min^{-1}, 210 nm, room temperature); t_r (major) = 15.3 min, t_r (minor) = 19.7 min, 98:2 e.r.).

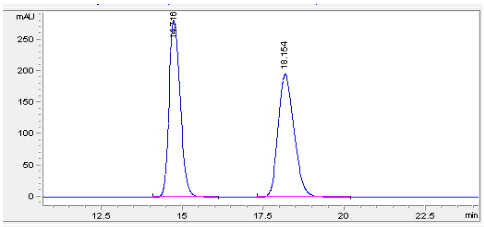

#	Time	Type	Area	Height	Width	Area%	Symmetry
1	14.716	BB	6702.1	280.7	0.3705	50.004	0.746
2	18.154	BB	6701	195.4	0.5321	49.996	0.758

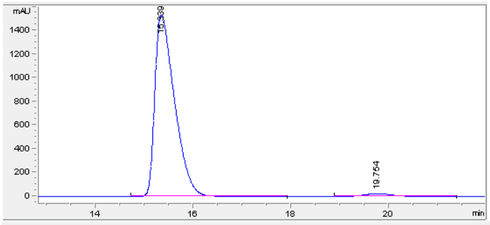

#	Time	Type	Area	Height	Width	Area%	Symmetry
1	15.339	BB	42824.5	1530.8	0.4264	97.924	0.466
2	19.754	BB	907.7	23.5	0.5974	2.076	0.856

Enantioenriched: (*R*)- 3,3'-di(anthracen-9-yl)-[1,1'-binaphthalene]-2,2'-diol **L1** (77 mg, 0.12 mmol, 0.6 equiv.), toluene (0.2 M, 1 mL), $AlMe_3$ in toluene (1.0 M, 0.08 mL, 0.08 mmol, 0.4 equiv.) and **3h** (60.5 mg, 0.20 mmol, 1 equiv.) were subjected to **General procedure 5B.** Purification by column chromatography (Pentane:Et_2O, 98:2) afforded the title compound as a white solid **4h** (41.5 mg, 73% yield).

HPLC: Enantiomeric excess was determined by HPLC with a Chiralpak® IG column (99.5:0.5 hexane:IPA, 1.0 mL min^{-1}, 210 nm, room temperature); t_r (major) = 14.0 min, t_r (minor) = 17.5 min, 93:7 e.r.).

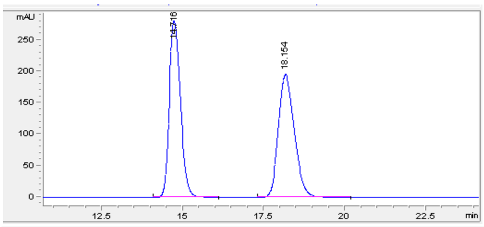

#	Time	Type	Area	Height	Width	Area%	Symmetry
1	14.716	BB	6702.1	280.7	0.3705	50.004	0.746
2	18.154	BB	6701	195.4	0.5321	49.996	0.758

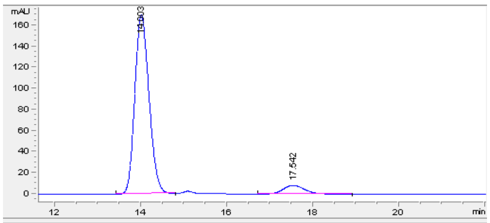

#	Time	Type	Area	Height	Width	Area%	Symmetry
1	14.003	BB	3933	170.9	0.3585	93.002	0.798
2	17.542	BB	296	8.3	0.5419	6.998	0.843

(6-(But-3-en-1-yl)cyclohex-1-en-1-yl)(2,3,4,5,6-pentamethylphenyl)methanone, 4i

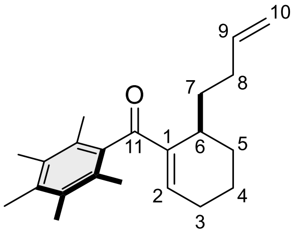

Racemic: *tert*-Butanol (22 mg, 0.30 mmol, 3.0 equiv.), toluene (0.2 M, 0.5 mL), $AlMe_3$ in toluene (1.0 M, 0.10 mL, 0.10 mmol, 1.0 equiv.) and **3i** (66 mg, 0.20 mmol, 1.0 equiv.) were subjected to **General procedure 4.** Purification

by column chromatography (Pentane:Et_2O, 98:2) afforded the title compound **4i** as a white solid (30 mg, 50% yield).

Enantioenriched: (*R*)-3,3'-di(anthracen-9-yl)-[1,1'-binaphthalene]-2,2'-diol **L1** (128 mg, 0.20 mmol, 1.0 equiv.), *tert*-butanol (14.8 mg, 0.20 mmol, 1.0 equiv.), *m*-xylene (1.0 mL, 0.2 M), $AlMe_3$ in toluene (2.0 M, 0.1 mL, 0.20 mmol, 1.0 equiv.) and **3i** (66 mg, 0.20 mmol, 1.0 equiv.) were subjected to **General procedure 5A.** Purification by column chromatography (Pentane:Et_2O, 98:2) afforded the title compound **4i** as a white solid (57 mg, 87% yield, 90.5:9.5 e.r.).

^{1}H NMR (400 MHz, $CDCl_3$) δ 6.40 (t, *J* = 3.9 Hz, 1H, H-2), 5.84 (ddt, *J* = 16.9, 10.2, 6.6 Hz, 1H, H-9), 5.00 (m, 1H, H-10), 4.91 (dd, *J* = 10.1, 2.4, 1H, H-10’), 2.81 (m, 1H, H-6), 2.16 (m, 5H, H-8, 3×$ArCH_3$), 2.12 – 1.99 (m, 8H, H-3, 6×$ArCH_3$), 1.95 (s, 3H, 3×$ArCH_3$), 1.93 (s, 3H, 3×$ArCH_3$), 1.81 (m, 2H, H-5, H-7), 1.64 – 1.51 (m, 2H, H-4), 1.42 (tt, *J* = 18.4, 5.8 Hz, 1H, H-5’), 1.34 – 1.11 (m, 1H, H7’).

^{13}C NMR (101 MHz, $CDCl_3$) δ 202.7 (C-11), 146.2 (C-2), 144.7 (ArC), 139.0 (C-9), 138.2 (ArC), 134.8 (C-1), 132.5 (ArC), 132.5 (ArC’), 129.3 (ArC), 129.1 (ArC’), 114.4 (C-10), 32.08 (C-8), 32.1 (C-7), 30.7 (C-6), 26.4 (C-3), 24.8 (C-5), 17.4 ($ArCH_3$), 17.2 ($ArCH_3$’), 16.9 (C-4), 16.7 ($ArCH_3$), 16.0 ($ArCH_3$), 15.9 ($ArCH_3$’).

IR (film) ν_{max}/cm^{-1}: 2933, 2858, 1653, 1630, 1452, 1420.

HRMS (ESI+): Found $[M+K]^+$ = 349.1932; $C_{22}H_{30}OK$ requires 349.1928, Δ 1.08 ppm.

m.p.: 82-84 °C.

$[\alpha]_D^{20}$ =+ 27.0 (c = 0.7, $CHCl_3$).

HPLC: Enantiomeric excess was determined by HPLC with a Chiralpak® IG column (99:1 hexane: IPA, 1.0 ml min^{-1}, 254 nm, room temperature); t_r (minor) = 21.1 min, t_r (major) = 35.7 min, 90.5:9.5 e.r.

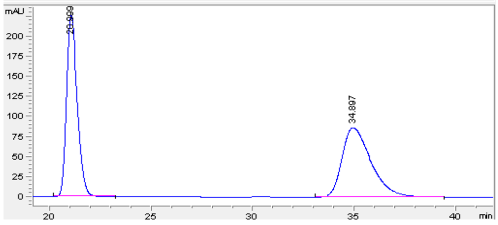

#	Time	Type	Area	Height	Width	Area%	Symmetry
1	20.999	BB	8533.2	225.2	0.5882	50.230	0.677
2	34.897	BB	8455	86.2	1.4239	49.770	0.583

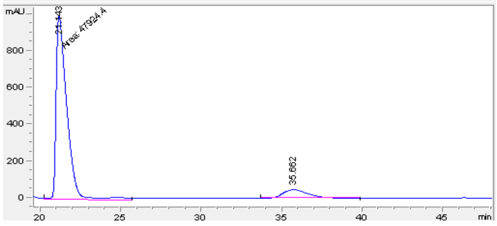

#	Time	Type	Area	Height	Width	Area%	Symmetry
1	21.143	MM T	47924.4	998	0.8004	90.665	0.352
2	35.662	BB	4934.2	46	1.5391	9.335	0.647

Enantioenriched: (*R*)- 3,3'-di(anthracen-9-yl)-[1,1'-binaphthalene]-2,2'-diol **L1** (77 mg, 0.12 mmol, 0.6 equiv.), toluene (0.2 M, 1 mL), $AlMe_3$ in toluene (1.0 M, 0.080 mL, 0.080 mmol, 0.4 equiv.) and **3i** (66 mg, 0.20 mmol, 1.0 equiv.) were subjected to **General procedure 5B.** Purification by column chromatography (Pentane:Et_2O, 98:2) afforded the title compound **4i** as a white solid (47 mg, 74% yield, 90.5:9.5 e.r.).

HPLC: Enantiomeric excess was determined by HPLC with a Chiralpak® IG column (99:1 hexane: IPA, 1.0 ml min^{-1}, 254 nm, room temperature); t_r (minor) = 21.4 min, t_r (major) = 38.0 min, 90.5:9.5 e.r.

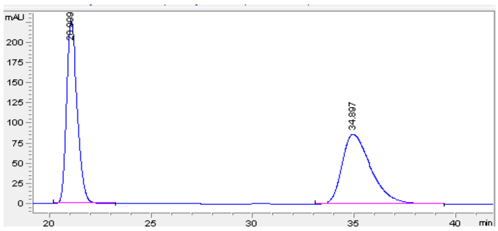

#	Time	Type	Area	Height	Width	Area%	Symmetry
1	20.999	BB	8533.2	225.2	0.5882	50.230	0.677
2	34.897	BB	8455	86.2	1.4239	49.770	0.583

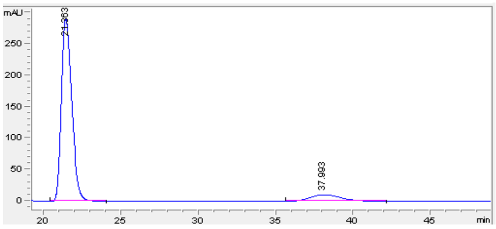

#	Time	Type	Area	Height	Width	Area%	Symmetry
1	21.363	BB	13342.2	292.3	0.7086	90.405	0.663
2	37.993	BB	1416.1	10.4	1.6059	9.595	0.759

Enantioenriched: (*R*)- 3,3'-di(anthracen-9-yl)-[1,1'-binaphthalene]-2,2'-diol **L1** (77 mg, 0.12 mmol, 0.6 equiv.), toluene (0.2 M, 1 mL), $AlMe_3$ in toluene (1.0 M, 0.080 mL, 0.080 mmol, 0.4 equiv.) and **3i** (66 mg, 0.20 mmol, 1.0 equiv.) were subjected to **General procedure 5C.** Purification by column chromatography (Pentane:Et_2O, 98:2) afforded the title compound **4i** as a white solid (43 mg, 70% yield, 92.5:7.5 e.r.).

HPLC: Enantiomeric excess was determined by HPLC with a Chiralpak® IG column (99:1 hexane: IPA, 1.0 ml min^{-1}, 254 nm, room temperature); t_r (minor) = 22.7 min, t_r (major) = 39.1 min, 92.5:7.5 e.r.

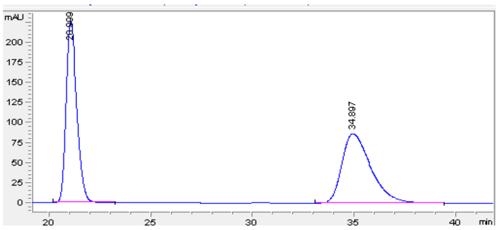

#	Time	Type	Area	Height	Width	Area%	Symmetry
1	20.999	BB	8533.2	225.2	0.5882	50.230	0.677
2	34.897	BB	8455	86.2	1.4239	49.770	0.583

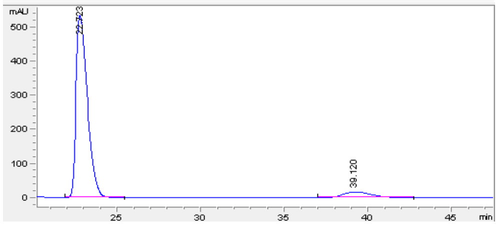

#	Time	Type	Area	Height	Width	Area%	Symmetry
1	22.723	BB	24959.5	532.5	0.7148	92.432	0.525
2	39.12	BB	2043.5	17.2	1.3992	7.568	0.715

(6-Isopropylcyclohex-1-en-1-yl)(2,3,4,5,6-pentamethylphenyl)methanone 4j

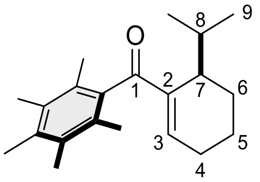

Racemic: *tert*-Butanol (22 mg, 0.30 mmol, 3.0 equiv.), toluene (0.2 M, 0.5 ml), $AlMe_3$ in toluene (1.0 M, 0.1 mL, 0.1 mmol, 1.0 equiv.) and mixture of E/Z (5:1) 7-hydroxy-3-isopropyl-1-(2,3,4,5,6-pentamethylphenyl)hept-2-en-1-one (32 mg, 0.10 mmol, 1.0 equiv.) were subjected to **General procedure 4.** Purification by column chromatography (Pentane:Et_2O, 98:2) afforded the title compound as a colourless oil (5.4 mg, 0.017 mmol, 17%).

Enantioenriched A: (*R*)-3,3'-di(anthracen-9-yl)-[1,1'-binaphthalene]-2,2'-diol **L1** (128 mg, 0.20 mmol, 1.0 equiv.), *tert*-butanol (14.8 mg, 0.20 mmol, 1.0 equiv.), *m*-xylene (1.0 mL, 0.2 M), $AlMe_3$ in toluene (2.0 M, 0.1 mL, 0.20 mmol, 1.0 equiv.) and **3j** (63 mg, 0.20 mmol, 1.0 equiv.) were subjected to **General procedure 5A.** Purification by column chromatography (Pentane:Et_2O, 98:2) afforded the title compound **4j** as a white solid (41 mg, 68% yield, 91:9 e.r.).

^{1}H NMR (400 MHz, $CDCl_3$) δ 6.63 (t, *J* = 4.2 Hz, 1H, H-3), 2.84 (q, *J* = 5.2 Hz, 1H, H-7), 2.41 – 2.28 (m, 1H, H-8), 2.24 (s, 3H, 3×$ArCH_3$), 2.18 (s, 6H, 6×$ArCH_3$), 2.16 – 2.09 (m, 2H, H-4), 2.06 (s, 3H, 3×$ArCH_3$), 2.00 (s, 3H, 3×$ArCH_3$), 1.81 (ddt, *J* = 12.8, 9.2, 4.9 Hz, 1H, H-6), 1.71 (qd, *J* = 10.1, 4.4 Hz, 1H, H-5), 1.62 – 1.45 (m, 2H, H-5’, H-6’), 1.07 (d, *J* =

6.9 Hz, 3H, H-9), 0.88 (d, *J* = 6.9, 3H, H-9).

^{13}C NMR (101 MHz, $CDCl_3$) δ 203.1 (C-1), 148.3 (C-3), 143.8 (ArC), 138.6 (ArC), 134.7 (C-2), 132.5 (ArC), 129.12 (ArC), 128.9 (ArC') 36.8 (C-7), 29.5 (C-8), 26.2 (C-4), 22.5 (C-6), 21.6 (C-9), 19.3 (C-5), 18.8 (C-9'), 17.4 ($ArCH_3$), 17.3 ($ArCH_3$'), 16.7 ($ArCH_3$), 16.0 ($ArCH_3$), 16.0 ($ArCH_3$').

IR (film) ν_{max}/cm^{-1}: 2947, 2870, 1710, 1662, 1462, 1358.

HRMS (ESI+): Found $[M+H]^+$ = 299.2366; $C_{21}H_{31}O$ requires 299.2369, Δ -1.23 ppm.

m.p.: 100 - 102 °C.

$[\alpha]_D^{20}$ = -30.7 (c = 0.3, $CHCl_3$).

HPLC: Enantiomeric excess was determined by HPLC with a Chiralpak® IG column (99:1 hexane: IPA, 1.0 ml min^{-1}, 254 nm, room temperature); t_r (minor) = 19.2 min, t_r (major) = 28.8 min, 91:9 e.r.

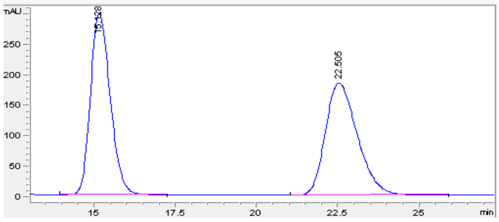

#	Time	Type	Area	Height	Width	Area%	Symmetry
1	15.128	BB	12573.4	296.8	0.6563	50.037	0.75
2	22.505	BB	12555	183.2	1.0554	49.963	0.695

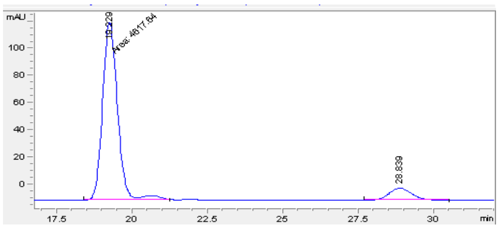

#	Time	Type	Area	Height	Width	Area%	Symmetry
1	19.229	MM T	4617.6	131.4	0.5857	90.947	2.31E-4
2	28.839	BB	459.7	8.8	0.6938	9.053	0.848

Enantioenriched B: (*R*)- 3,3'-di(anthracen-9-yl)-[1,1'-binaphthalene]-2,2'-diol (77 mg, 0.12 mmol, 0.6 equiv.), toluene (0.2 M, 1 ml), $AlMe_3$ in toluene (1.0 M, 0.08 mL, 0.08 mmol, 0.4 equiv.) and **3j** (63 mg, 0.20 mmol, 1.0 equiv.) were subjected to **General procedure 5B.** Purification by column chromatography (Pentane:Et_2O, 98:2) afforded the title compound **4j** as a colourless oil (40 mg, 67% yield, 92:8 e.r.).

HPLC: Enantiomeric excess was determined by HPLC with a Chiralpak® IG column (99:1 hexane:IPA, 1.0 mL min^{-1}, 210 nm, room temperature); t_r (major) = 14.5 min, t_r (minor) = 22.1 min, 92:8 e.r.).

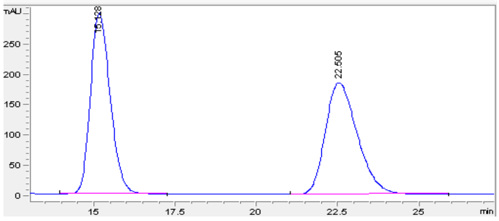

#	Time	Type	Area	Height	Width	Area%	Symmetry
1	15.128	BB	12573.4	296.8	0.6563	50.037	0.75
2	22.505	BB	12555	183.2	1.0554	49.963	0.695

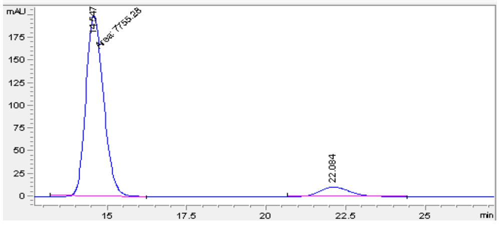

#	Time	Type	Area	Height	Width	Area%	Symmetry
1	14.547	MM T	7755.3	198.8	0.65	91.801	0.781
2	22.084	BB	692.7	10.5	0.8686	8.199	0.804

Enantioenriched C: (*R*)- 3,3'-di(anthracen-9-yl)-[1,1'-binaphthalene]-2,2'-diol **L1** (77 mg, 0.12 mmol, 0.6 equiv.), toluene (0.2 M, 1 ml), $AlMe_3$ in toluene (1.0 M, 0.08 mL, 0.08 mmol, 0.4 equiv.) and **3j** (63 mg, 0.20 mmol, 1.0 equiv.) were subjected to **General procedure 5C.** Purification by column chromatography (Pentane:Et_2O, 98:2) afforded the title compound **4j** as a colourless oil (35 mg, 58% yield, 93:7 e.r.).

HPLC: Enantiomeric excess was determined by HPLC with a Chiralpak® IG column (99:1 hexane:IPA, 1.0 mL min^{-1}, 210 nm, room temperature); t_r (major) = 14.5 min, t_r (minor) = 22.1 min, 93:7 e.r.).

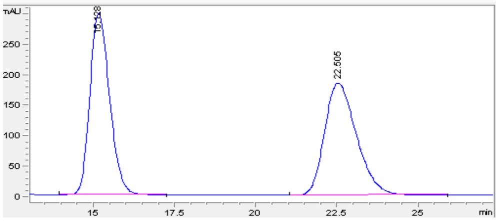

#	Time	Type	Area	Height	Width	Area%	Symmetry
1	15.128	BB	12573.4	296.8	0.6563	50.037	0.75
2	22.505	BB	12555	183.2	1.0554	49.963	0.695

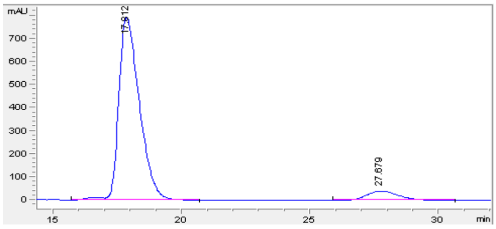

#	Time	Type	Area	Height	Width	Area%	Symmetry
1	17.812	VB R	44401.8	797.6	0.8452	92.790	0.601
2	27.679	BB	3450	40.5	1.2748	7.210	0.79

(6-(3-(Benzyloxy)propyl)cyclohex-1-en-1-yl)(2,3,4,5,6-pentamethylphenyl)methanone, 4k

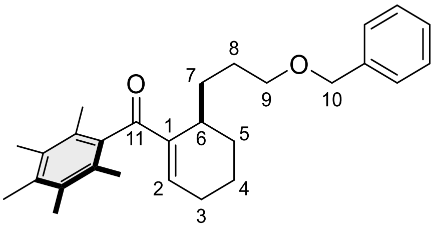

Racemic: *tert*-Butanol (22 mg, 0.30 mmol, 3 equiv.), toluene (0.2 M, 0.5 ml), $AlMe_3$ in toluene (1.0 M, 0.10 mL, 0.10 mmol, 1.0 equiv.) and mixture of E/Z (1:1) **S20** (84 mg, 0.20 mmol, 1.0 equiv.) were subjected to **General procedure 4.** Purification by column chromatography (Pentane:Et_2O, 98:2) afforded the title compound **4k** as a colourless oil (25 mg, 31% yield).

Enantioenriched: (*R*)-3,3'-di(anthracen-9-yl)-[1,1'-binaphthalene]-2,2'-diol **L1** (128 mg, 0.20 mmol, 1.0 equiv.), *tert*-butanol (14.8 mg, 0.20 mmol, 1.0 equiv.), *m*-xylene (1.0 mL, 0.2 M), $AlMe_3$ in toluene (2.0 M, 0.1 mL, 0.20 mmol, 1.0 equiv.), and **3k** (84 mg, 0.20 mmol, 1.0 equiv.) were subjected to **General Procedure 5A**. Purification by column chromatography (Pentane:Et_2O, 95:5) afforded the title compound **4k** as a white solid (54 mg, 67% yield, 96.5:3.5 e.r.).

^{1}H NMR (400 MHz, $CDCl_3$) δ 7.33 – 7.16 (m, 5H, 5×ArH), 6.40 (t, *J* = 3.9 Hz, 1H, H-2), 4.46 (s, 2H, H-10), 3.57 – 3.38 (m, 2H, H-9), 2.83 – 2.75 (m, 1H, H-6), 2.16 (s, 3H, 3×$ArCH_3$), 2.10 (s, 6H, 6×$ArCH_3$), 2.10 – 2.02 (m, 2H, H-3), 1.95 (s, 3H, 3×$ArCH_3$), 1.93 (s, 3H, 3×$ArCH_3$), 1.82 – 1.61 (m, 3H, H-7, H-8), 1.59 – 1.50 (m, 2H, H-4), 1.48 – 1.12 (m, 3H, H-5, H-7’).

^{13}C NMR (101 MHz, $CDCl_3$) δ 202.8 (C-11), 146.3 (C-2), 144.7 (ArC), 138.8 (ArC), 138.2 (ArC), 134.8 (ArC), 132.5 (ArC), 129.3 (ArC), 129.1 (ArC’), 128.3 (ArC), 127.7 (ArC), 127.4 (C-1), 72.8 (C-10), 70.7 (C-9), 31.0 (C-6), 29.3 (C-5), 28.1 (C-8), 26.4 (C-3), 25.0 (C-7), 17.4 ($ArCH_3$), 17.2 ($ArCH_3$’), 17.0 (C-4), 16.7 ($ArCH_3$), 16.0 ($ArCH_3$), 15.9 ($ArCH_3$’).

IR (film) ν_{max}/cm^{-1}: 2978, 2923, 1660, 1423, 1336, 1197.

HRMS (ESI+): Found $[M+H]^+$ = 405.2782; $C_{28}H_{37}O_2$ requires 405.2788, Δ -1.50 ppm.

m.p.: 64 - 65 °C.

$[\alpha]_D^{20}$ = -40.0 (c = 0.1, $CHCl_3$).

HPLC: Enantiomeric excess was determined by HPLC with a Chiralpak® IA column (99.5:0.5 hexane: IPA, 1.0 ml min^{-1}, 254 nm, room temperature); t_r (minor) = 18.6 min, t_r (major) = 25.7 min, 96.5:3.5 e.r.

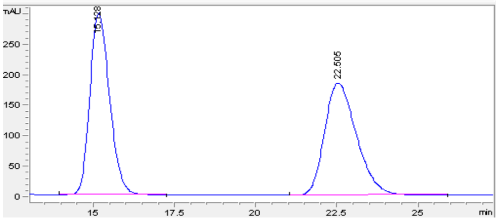

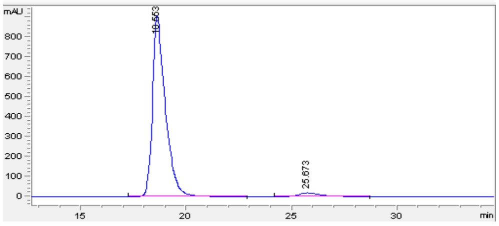

#	Time	Type	Area	Height	Width	Area%	Symmetry
1	15.128	BB	12573.4	296.8	0.6563	50.037	0.75
2	22.505	BB	12555	183.2	1.0554	49.963	0.695

#	Time	Type	Area	Height	Width	Area%	Symmetry
1	18.553	BB	38268.7	910.4	0.5944	96.356	0.502
2	25.673	BB	1447.3	19.1	1.0467	3.644	0.618

Enantioenriched: (*R*)-3,3'-di(anthracen-9-yl)-[1,1'-binaphthalene]-2,2'-diol **L1** (77 mg, 0.12 mmol, 0.6 equiv.), toluene (0.2 M, 1 ml), $AlMe_3$ in toluene (1.0 M, 0.080 mL, 0.08 mmol, 0.4 equiv.) and **3k** (84 mg, 0.20 mmol, 1.0 equiv.) were subjected to **General procedure 5C.** Purification by column chromatography (Pentane:Et_2O, 95:5) afforded the title compound **4k** as a white solid (28 mg, 35% yield, 97:3 e.r.) and RSM is (29 mg, 35% yield).

HPLC: Enantiomeric excess was determined by HPLC with a Chiralpak® IA column (99.5:0.5 hexane: IPA, 1.0 ml min^{-1}, 254 nm, room temperature); t_r (minor) = 21.0 min, t_r (major) = 28.5 min, 97:3 e.r.

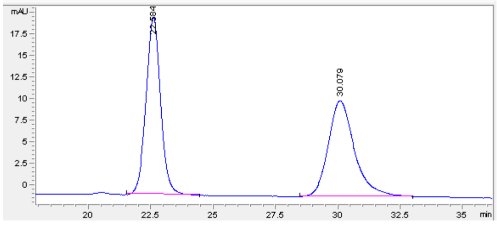

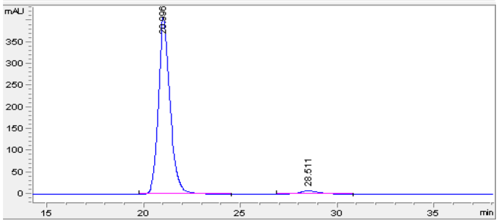

#	Time	Type	Area	Height	Width	Area%	Symmetry
1	22.584	BB	836.9	20.6	0.6132	50.750	0.999
2	30.079	BB	812.2	11.1	0.9307	49.250	0.784

#	Time	Type	Area	Height	Width	Area%	Symmetry
1	20.996	BB	17333.1	410.9	0.6202	96.847	0.744
2	28.511	BB	564.3	7.8	0.8711	3.153	0.812

3.4 Synthesis of alkylketone substrate 3l and cyclohexene 4l

11-Hydroxyundec-6-yn-5-one S21

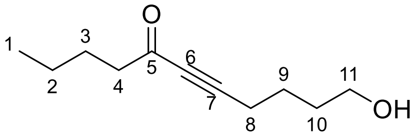

n-BuLi in hexanes (14.4 mL, 2.5 M, 36.0 mmol, 2.4 equiv.) was added dropwise to a solution of 5-hexyn-1-ol (1.77 g, 18.0 mmol, 1.2 equiv.) in THF (0.3 M, 50 mL) at –78 °C. The reaction mixture was stirred for 1 h, after which a solution of *N*-methoxy-*N*-methylpentanamide (2.18 g, 15.0 mmol, 1.0 equiv.) in THF (3.0 M) was added dropwise. The mixture was then warmed to RT and stirred for a further 1 h. The reaction was quenched by the addition of saturated aqueous ammonium chloride and extracted with EtOAc. The combined organic phases were dried over $MgSO_4$, filtered and concentrated *in vacuo*. The title compound was afforded after purification by FCC (20→60% EtOAc/Pentane) as a yellow oil (1.92 g, 10.5 mmol, 70% yield).

^{1}H NMR (400 MHz, $CDCl_3$) δ 3.72 – 3.64 (m, 2H, H_{11} x 2), 2.52 (t, *J* = 7.5 Hz, 2H, H_4 x 2), 2.47 – 2.37 (m, 2H, H_8 x 2), 1.75 – 1.56 (m, 6H, H_9 x 2, H_{10} x 2, H_3 x 2), 1.46 (s, 1H, OH), 1.41 – 1.27 (m, 2H, H_2 x 2), 0.91 (t, *J* = 7.5 Hz, 3H, H_1 x 3).

^{13}C NMR (101 MHz, $CDCl_3$) δ 188.7 (C_5), 93.7 (C_7), 81.3 (C_6), 62.3 (C_{11}), 45.4 (C_4), 31.8 (C_{10}), 26.3 (C_3), 24.3 (C_9), 22.3 (C_2), 18.9 (C_8), 13.9 (C_1).

HRMS (ESI+): Found $[M+H]^+$ = 183.1383; $C_{11}H_{19}O_2$ requires 183. 1380 Δ 1.85 ppm

IR (film) v_{max}/cm^{-1}: 3432, 2980, 2214, 1672, 1618, 1461.

1-(2-Butyltetrahydro-2*H*-pyran-2-yl)hexan-2-one 3l

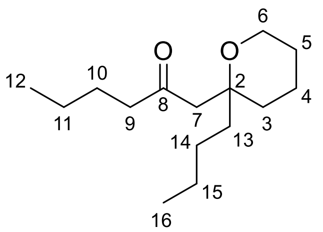

n-BuLi in hexanes (2.5 M, 13.2 mL, 3.0 mmol, 6.0 equiv.) was added dropwise to a solution of CuI (3.13 g, 16.5 mmol, 3.0 equiv.) in THF (0.3 M, 55 mL) at –78 °C. The reaction mixture was warmed to 0 °C and stirred for 1 h, affording a black precipitate. The mixture was cooled to –78 °C and a solution of 11-hydroxyundec-6-yn-5-one **S21** (1.00 g, 5.49 mmol, 1.0 equiv.) in THF (1.0 M, 4 mL) was added dropwise and stirred for 30 min. The reaction was quenched by the addition of saturated aqueous ammonium chloride (50 mL) and warmed to RT. The reaction was treated with 35% aqueous ammonium hydroxide solution (50 mL), affording a clear blue aqueous phase. The solution was extracted with EtOAc (3 x 100 mL), and the combined organic phases were washed with HCl (1.0 M, 100 mL), dried over $MgSO_4$, filtered, and concentrated *in vacuo*. The title compound was afforded after purification by FCC (5% Et_2O/pentane) as a yellow oil (536 mg, 2.23 mmol, 41% yield).

^{1}H NMR (500 MHz, $CDCl_3$) δ 3.69 – 3.60 (m, 2H, H_6 x 2), 2.60 (d, *J* = 2.5 Hz, 2H, H_7 x 2), 2.47 (td, *J* = 7.0, 1.0 Hz, 2H, H_9 x 2), 1.74 – 1.45 (m, 10H, H_3 x 2, H_4 x 2, H_{13} x 2, H_{10} x 2, H_5 x 2), 1.38 – 1.10 (m, 6H, H_{14} x 2, H_{11} x 2, H_{15} x 2), 0.94 – 0.84 (m, 6H, H_{12} x 3, H_{16} x 3).

^{13}C NMR (126 MHz, $CDCl_3$) δ 210.5 (C_8), 74.9 (C_2), 61.5 (C_6), 49.2 (C_7), 44.7 (C_9), 35.1 (C_3), 33.4 (C_{13}), 25.9 (C_5 or C_{10}), 25.8 (C_5 or C_{10}), 25.4 (C_{14}), 23.3 (C_{15}), 22.4 (C_{11}), 19.2 (C_4), 14.2 (C_{12} or C_{16}), 14.0 (C_{12} or C_{16}).

HRMS (ESI+): Found $[M+H]^+$ = 241.2161; $C_{15}H_{29}O_2$ requires 241.2162 Δ -0.34 ppm.

IR (film) v_{max}/cm^{-1}: 2957, 2871, 1709, 1460, 1379, 1284.

1-(6-Butylcyclohex-1-en-1-yl)pentan-1-one 4l

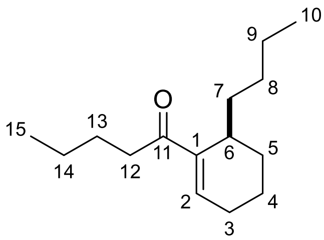

Racemic: 1-(2-Butyltetrahydro-2*H*-pyran-2-yl)hexan-2-one **3l** (48.1 mg, 0.200 mmol, 1.0 equiv.), Al(O*t*-Bu)$_3$ (49.3 mg, 0.200 mmol, 1.0 equiv.), and PhMe (0.2 M, 1.0 mL) were subjected to **General Procedure 4**. The title

compound was afforded after purification by FCC (5% Et_2O/pentane) as a colourless oil (32.2 mg, 0.145 mmol, 72% yield).

Enantioenriched: (*R*)-3,3'-di(anthracen-9-yl)-[1,1'-binaphthalene]-2,2'-diol **L1** (128 mg, 0.200 mmol, 1.0 equiv.), *tert*-butanol (14.8 mg, 0.200 mmol, 1.0 equiv.), *m*-xylene (1.0 mL, 0.2 M), $AlMe_3$ in PhMe (2.0 M, 0.1 mL, 0.200 mmol, 1.0 equiv.), and1-(2-butyltetrahydro-2*H*-pyran-2-yl)hexan-2-one **3l** (48.1 mg, 0.200 mmol, 1.0 equiv.) were subjected to **General Procedure 5A**. The title compound was afforded after purification by FCC (3% Et_2O/pentane) as a colourless oil (30.1 mg, 0.135 mmol, 68% yield).

^{1}H NMR (400 MHz, $CDCl_3$) δ 6.78 (s, 1H, H_2), 2.70 – 2.50 (m, 3H, H_6, H_{12} x 2), 2.31 – 2.10 (m, 2H, H_3 x 2), 1.77 – 1.67 (m, 1H, H_{5a}), 1.67 – 1.53 (m, 4H, H_4 x 2, H_{13} x 2), 1.51 – 1.06 (m, 9H, H_{5b}, H_7 x 2, H_8 x 2, H_9 x 2, H_{14} x 2), 0.95 – 0.84 (m, 6H, H_{10} x 3, H_{15} x 3).

^{13}C NMR (101 MHz, $CDCl_3$) δ 202.1 (C_{11}), 144.4 (C_1), 138.9 (C_2), 37.5 (C_{12}), 33.3 (C_7), 31.6 (C_6), 30.1 (C_8), 27.2 (C_{13}), 26.2 (C_3), 25.4 (C_5), 22.9 (C_{14} or C_9), 22.7 (C_{14} or C_9), 17.3 (C_4), 14.2 (C_{15} or C_{10}), 14.1 (C_{15} or C_{10}).

HRMS (ESI+): Found $[M+H]^+$ = 223.2054; $C_{15}H_{27}O$ requires 223.2056 Δ −1.16 ppm.

IR (film) ν_{max}/cm^{-1}: 2957, 2933, 2872, 1671, 1464, 1381.

$[\alpha]_D^{25}$ = −5.86 (c = 1.0, $CHCl_3$).

HPLC: Enantiomeric excess was determined by HPLC with a Chiralpak® IG column (99.5:0.5 hexane:IPA, 1.0 mL min^{-1}, 230 nm, room temperature); t_r (minor) = 7.12 min, t_r (major) = 7.91 min, 11:89 e.r.

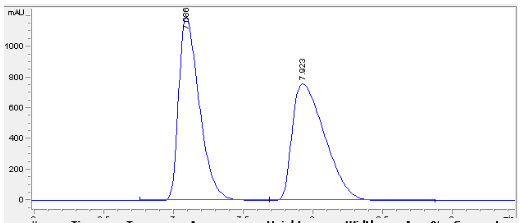

#	Time	Type	Area	Height	Width	Area%	Symmetry
1	7.086	BB	12230.4	1189.1	0.158	49.954	0.538
2	7.923	BB	12253	755.2	0.2573	50.046	0.452

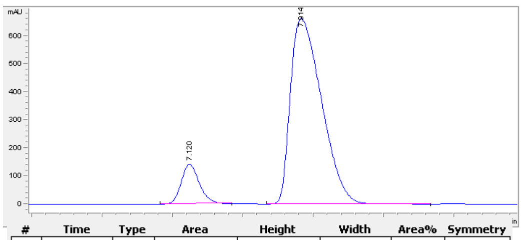

#	Time	Type	Area	Height	Width	Area%	Symmetry
1	7.12	BB	1268.4	141.8	0.1384	11.045	0.786
2	7.914	BB	10215.5	664.3	0.2454	88.955	0.506

3.5 Derivatization of the Acyl Cyclohexenes

6-Butylcyclohex-1-ene-1-carboxylic acid, 5

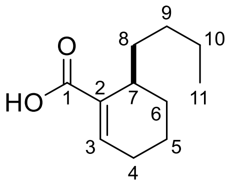

A 2–5 mL Biotage® microwave vial was charged with **4g** (31 mg, 0.10 mmol, 1.0 equiv.), hexafluoroisopropanol (0.88 mL) and 37% aq. HCl (12 M, 0.13 mL). The reaction vessel was sealed with a microwave vial cap (containing a Reseal™ septum) and heated at 65 °C for 15 h. The reaction was then cooled to RT, diluted with water (5 ml) and extracted with CH_2Cl_2 (3 x 10 mL), dried over $MgSO_4$, filtered and concentrated in vacuo. Purification by column chromatography (CH_2Cl_2:MeOH, 98:2) afforded the title compound **5** as brown oil (15 mg, 81% yield).

^{1}H NMR (400 MHz, $CDCl_3$) δ 7.07 (t, *J* = 4.0 Hz, 1H, H-3), 2.53 (tt, *J* = 6.6, 3.1 Hz, 1H, H-7), 2.38 – 1.94 (m, 2H, H-4), 1.80 – 1.70 (m, 1H, H-6), 1.70 – 1.46 (m, 4H, H-6', H-8, H-9), 1.46 – 1.16 (m, 5H, H-5, H-8', H-10), 0.97 – 0.82 (m, 3H, H-11).

^{13}C NMR (101 MHz, $CDCl_3$) δ 173.2 (C-1), 142.1 (C-3), 134.5 (C-2), 33.1 (C-8), 32.4 (C-7), 29.9 (C-5 or C-9), 26.1 (C-4), 25.2 (C-6), 22.8 (C-5 or C-9), 17.0 (C-10), 14.1 (C-11).

IR (film) ν_{max}/cm^{-1}: 2933, 1683, 1638, 1421, 1273, 1155.

HRMS (ESI+): Found $[M+H]^+$ = 183.1377; $C_{11}H_{19}NO_2$ requires 183.1380 Δ -1.40 ppm.

$[\alpha]_D^{20}$ = −30.0 (c = 0.1, $CHCl_3$).

Benzyl 6-butylcyclohex-1-ene-1-carboxylate, S22

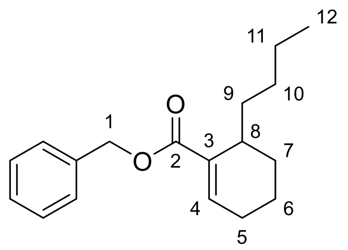

To a solution of **5** (8.0 mg, 0.040 mmol, 1.0 equiv) in anhydrous EtOH (0.5 mL) was added Cs_2CO_3 (7.8 mg, 0.024 mmol, 0.6 equiv) and the mixture was stirred at RT for 15 min. Benzyl bromide (5 µL, 0.044 mmol, 1.1 equiv) was then added, and the reaction mixture was stirred at RT for 16 h. The solvent was removed under reduced pressure, and the residue was extracted with EtOAc (3 × 10 mL). The combined organic layers were washed with brine (2 × 10 mL), dried over $MgSO_4$, and concentrated in vacuo. Purification by column chromatography (pentane/Et_2O = 97:3) afforded the title compound **S22** as a yellow oil (6 mg, 55% yield).

^{1}H NMR (400 MHz, $CDCl_3$) δ 7.47 – 7.17 (m, 5H, 5×ArH), 6.98 (d, *J* = 3.5 Hz, 1H, H-4), 5.24 (d, *J* = 12.5 Hz, 1H, H-1), 5.16 (d, *J* = 12.5 Hz, 1H, H-1'), 2.58 (m, 1H, H-8), 2.29 – 2.07 (m, 2H, H-5), 1.80 – 1.69 (m, 1H, H-7), 1.65 – 1.47 (m, 4H, H-7', H-9, H-10), 1.44 – 1.16 (m, 5H, H-6, H-9', H-11), 0.95 – 0.83 (m, 3H, H-12).

^{13}C NMR (101 MHz, $CDCl_3$) δ 167.5 (C-2), 139.7 (C4), 136.4 (C-3), 135.0 (ArC), 128.4 (ArC), 128.0 (ArC), 128.0 (ArC), 65.9 (C-1), 33.2 (C-9), 32.7 (C-8), 29.9 (C-11), 25.9 (C-5), 25.4 (C-7), 22.7 (C-6), 17.1 (C-10), 14.0 (C12).

IR (film) ν_{max}/cm^{-1}: 3020, 2918, 2360, 1712, 1498, 1230.

HRMS (ESI+): Found $[M+Na]^+$ = 273.1848; $C_{18}H_{24}O_2$ requires 273.1849 Δ -0.39 ppm.

$[\alpha]_D^{20}$ = - 50.4 (c = 0.1, $CHCl_3$)

To enable determination of the enantiomeric ratio of the title compound, a racemic sample was prepared following the same synthetic procedure.

HPLC: Enantiomeric excess was determined by HPLC with a Chiralpak® IG column (99.5:0.5 hexane: IPA, 1.0 ml min^{-1}, 254 nm, room temperature); t_r (minor) = 9.3 min, t_r (major) = 10.2 min, 98:2 e.r.

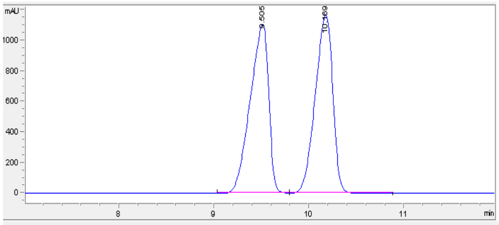

#	Time	Type	Area	Height	Width	Area%	Symmetry
1	9.505	BV	15061	1108	0.2166	49.991	2.022
2	10.169	VB	15066.4	1173.4	0.2018	50.009	1.451

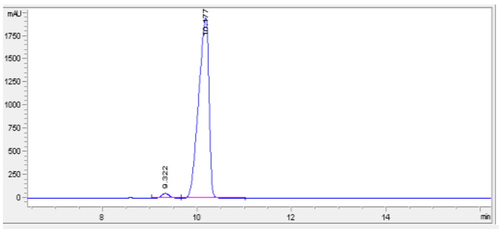

#	Time	Type	Area	Height	Width	Area%	Symmetry
1	9.322	VB	591.3	51.2	0.1787	1.964	0.903
2	10.177	BB	29520.4	1940.6	0.2455	98.036	2.149

***tert*-Butyl (6-butylcyclohex-1-ene-1-carbonyl)-D-phenylalaninate, 6**

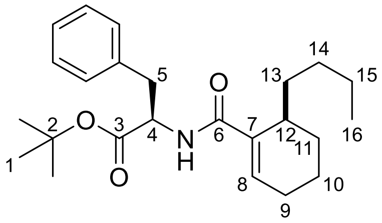

A solution of **5** (18 mg, 0.10 mmol, 1 equiv.) in CH_2Cl_2 (2 mL) was added to a solution of D-Phenylalanine tert-butyl ester hydrochloride (77 mg, 0.30 mmol, 3 equiv.) and DMAP (0.1 g, 0.8 mmol, 8 equiv.) in CH_2Cl_2 (3 mL) at 25 °C. Then EDCI · HCl (77 mg, 0.40 mmol, 4 equiv.) was added and the mixture was stirred under inert conditions for 24 h. Saturated $NaHCO_3$ solution (10 mL) was added, the layers separated and the aqueous layer was extracted with chloroform (3 x 10 mL). The combined organic extracts were washed with brine (2 x 10 mL), dried ($MgSO_4$) and the solvent was removed under reduced pressure. Purification by column chromatography (pentane:Et_2O, 90:10) afforded the title compound as white solid (25 mg, 65% yield).

^{1}H NMR (400 MHz, $CDCl_3$) δ 7.33 – 7.24 (m, 3H, 3×ArH), 7.21 – 7.16 (m, 2H, 2×ArH), 6.37 (t, *J* = 3.9, 1H, H-8), 6.15 (d, *J* = 7.9 Hz, 1H, N-H), 4.84 (dt, *J* = 7.9, 6.0 Hz, 1H, H-4), 3.15 (d, *J* = 5.9 Hz, 2H, H-5), 2.54 (m, 1H, H-12), 2.14 – 2.07 (m, 2H, H-9), 1.75 – 1.60 (m, 4H, H-10, H-11), 1.43 (m, 10H, H-1, H-13), 1.38 – 1.17 (m, 5H, H-13', H-14, H-15), 0.93 – 0.85 (m, 3H, H-16).

^{13}C NMR (101 MHz, $CDCl_3$) δ 170.9 (C-4), 169.0 (C-6), 139.5 (ArC), 136.4 (C-7), 131.8 (C-8), 129.5 (ArC), 128.3 (ArC), 126.9 (ArC), 82.2 (C-2), 53.4 (C-4), 38.2 (C-5), 33.3 (C-13), 33.1 (C-6), 29.7 (C-14), 28.0 (C-1), 26.0 (C-11), 25.4 (C-9), 22.8 (C-15), 18.1 (C-10), 14.1 (C-16).

IR (film) ν_{max}/cm^{-1}: 2980, 2360, 1746, 1660, 1626, 1381.

HRMS (ESI+): Found $[M+H]^+$ = 386.2703; $C_{24}H_{36}NO_3$ requires 386.2690 Δ 3.44 ppm.

m.p.: 110-111 °C.

$[\alpha]_D^{20}$ = −72.0 (c = 0.1, $CHCl_3$).

(3-Bromo-6-butylcyclohex-1-en-1-yl)(2,3,4,5,6-pentamethylphenyl)methanone, 7

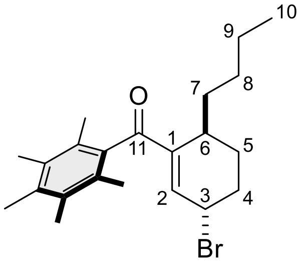

A stirred solution of **4g** (33 mg, 0.10 mmol, 1.0 equiv.) in $CHCl_3$ (1 mL) was cooled to –17 °C and Br_2 (5 µL, 1.0 equiv.) was added dropwise. The resulting solution was stirred at –17 °C for 15 min and then was warmed to RT and stirred for 3 h. The reaction mixture was concentrated *in vacuo*, and purification by column chromatography (Pentane:Et_2O, 99:1) afforded the title compound **7** as a mixture of diastereomers (31.5 mg, 86%, 70:30 d.r.) as a white solid. NMR of the crude reaction mixture showed a ratio of 80:20 d.r. Careful chromatography allowed isolation of a small sample of the major diastereoisomer for X-ray analysis.

^{1}H NMR (400 MHz, $CDCl_3$) **Major diastereoisomer:** δ 6.42 (d, *J* = 4.7 Hz, 1H, H-2), 4.83 (td, *J* = 4.7, 1.8 Hz, 1H, H-3), 3.01 – 2.86 (m, 1H, H-6), 2.24 (s, 3H, 3×$ArCH_3$), 2.18 (s, 6H, 6×$ArCH_3$), 2.14 – 2.05 (m, 2H,), 2.01 (brs, *J* = 5.6 Hz, 6H, 6×$ArCH_3$), 1.98 – 1.90 (m, 1H), 1.84 (brd, *J* = 13.8 Hz, 1H), 1.68 (s, 1H), 1.50 – 1.34 (m, 4H), 1.23 – 1.17 (m, 1H), 0.93 (t, *J* = 7.0 Hz, 3H, H-10).

^{13}C NMR (101 MHz, $CDCl_3$) **Major diastereoisomer:** δ 203.1 (C-11), 144.5, 141.9 (C-2), 137.4 (ArC), 135.5 (ArC), 133.0 (ArC), 132.8 (ArC), 47.3 (C-3), 32.1, 30.7 (C-6), 30.2, 27.2, 22.8, 20.0, 17.5 ($ArCH_3$), 16.8 ($ArCH_3$), 16.1 ($ArCH_3$), 14.3 (C-10).

IR (film) ν_{max}/cm^{-1}: 2958, 2918, 2854, 1654, 1625, 1458, 1186.

HRMS (ESI+): Found $[M+Na]^+$ = 413.1462.; $C_{22}H_{31}BrONa$ requires 413.1450, Δ 2.79 ppm

m.p.: 105-107 °C.

$[\alpha]_D^{20}$ = −163.3 (c = 0.1, $CHCl_3$).

Diagnostic peaks for minor diastereomer: ^{1}H NMR (400 MHz, $CDCl_3$) δ 4.73 (td, *J* = 6.6, 1.4 Hz, 1H, H-3), 2.89 – 2.79 (m, 1H, H-6).

4-Butyl-3-(2,3,4,5,6-pentamethylbenzoyl)cyclohex-2-en-1-one, 8

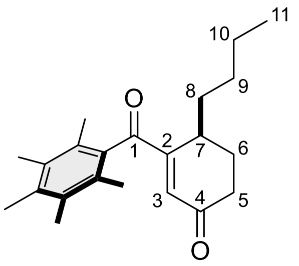

To a suspension of sodium bicarbonate (42 mg, 0.50 mmol, 5.0 equiv.) in a mixture of dry DMSO (0.25 mL) and dry MeCN (0.25 mL) was added mixture (ca 4:1 d.r.) of the bromide substrate **7** (39 mg, 0.10 mmol, 1.0 equiv.) under nitrogen. The reaction mixture was stirred at 80 °C for 8 h. After cooling to RT, the mixture was diluted with H_2O (10 mL) and extracted with EtOAc (3 × 10 mL). The combined organic layers were washed with saturated aqueous $NaHCO_3$ (10 mL) and brine (10 mL), dried (Na_2SO_4), filtered, and concentrated *in vacuo*. Purification by column chromatography (pentane:Et_2O, 90:10) afforded the title compound **8** as yellow solid (20 mg, 61% yield).

^{1}H NMR (400 MHz, $CDCl_3$) δ 6.08 (s, 1H, H-3), 3.09 – 3.06 (m, 1H, H-7), 2.57 (ddd, *J* = 17.9, 14.5, 5.2 Hz, 1H, H-5), 2.48 – 2.36 (m, 1H, H-5'), 2.23 (m, 4H, H-6, $ArCH_3$), 2.16 (s, 6H, 2×$ArCH_3$), 2.04 (dt, *J* = 14.2, 4.8 Hz, 1H, H-6'), 1.98 (s, 6H, 6×$ArCH_3$), 1.80 – 1.68 (m, 2H, H-8), 1.56 – 1.30 (m, 4H, H-9, H-10), 0.95 (t, *J* = 7.1 Hz, 3H, H-11).

^{13}C NMR (101 MHz, $CDCl_3$) δ 204.2 (C-4), 202.39 (C-1), 159.05 (ArC), 136.2 (ArC), 136.1 (ArC'), 134.6 (C-3), 133.0

(ArC), 129.0 (ArC), 33.4 (C-5), 31.4 (C-7), 30.7 (C-8), 30.5 (C-9), 24.8 (C-6), 22.6 (C-10), 17.4 (ArCH_3), 16.7 (ArCH_3), 15.9 (ArCH_3), 14.0 (C-11).

IR (film) ν_{max}/cm^{-1}: 2980, 2360, 1746, 1660, 1626, 1381.

HRMS (ESI+): Found $[M+H]^+$ = 327.2331; $C_{22}H_{31}NO_2$ requires 327.2319 Δ 1.83 ppm.

m.p.: 62-64 °C.

$[\alpha]_D^{20}$ = −126.0 (c = 0.1, $CHCl_3$).

To enable determination of the enantiomeric ratio of the title compound, a racemic sample was prepared following the same synthetic procedure.

HPLC: Enantiomeric excess was determined by HPLC with a Chiralpak® IA column (99.9:0.1 hexane: IPA, 1.0 ml min^{-1}, 254 nm, room temperature); t_r (minor) = 15.7 min, t_r (major) = 19.8 min, 98.5:1.5 e.r.

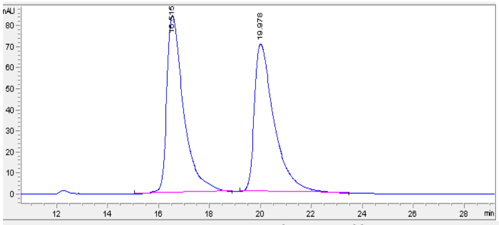

#	Time	Type	Area	Height	Width	Area%	Symmetry
1	16.515	BB	3877	84.2	0.6693	50.633	0.47
2	19.978	BB	3780	70	0.7861	49.367	0.416

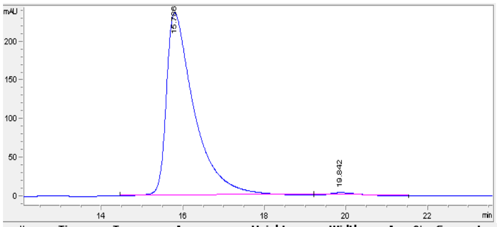

#	Time	Type	Area	Height	Width	Area%	Symmetry
1	15.786	BB	11370.5	236.6	0.6927	98.476	0.39
2	19.842	BB	176	3.6	0.5873	1.524	0.543

(3,3-Dibenzyl-6-butylcyclohex-1-en-1-yl)(2,3,4,5,6-pentamethylphenyl)methanone, 9

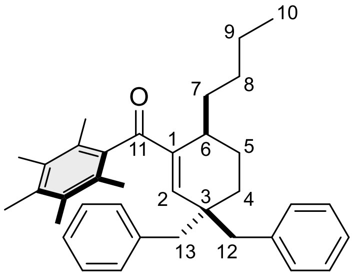

A 2–5 mL Biotage® microwave vial was charged with **4g** (33 mg, 0.10 mmol, 1.0 equiv.) in dry THF (1 mL) under N_2 and LiHMDS (0.25 mL, 1.0 M in THF, 0.25 mmol, 2.5 equiv.) was added dropwise the resulting solution was stirred at RT for 1 h. Then a solution of benzyl iodide (87 mg, 0.4 mmol, 4.0 equiv.) in dry THF (1 mL) was added and was stirred at RT for 4 h until the full consumption of starting material **4g**. The reaction mixture was quenched by addition of sat. aq. NH_4Cl. The layers were separated and the aqueous layer was extracted with Et_2O (3 x 10 mL), dried over $MgSO_4$, filtered and concentrated in vacuo. The title compound **9** was afforded after purification by FCC (Pentane:Et_2O, 98:2) as a colourless oil (36.4 mg, 70% yield).

^{1}H NMR (400 MHz, $CDCl_3$) δ 7.21 (dd, *J* = 5.0, 1.9 Hz, 3H), 7.18 – 7.12 (m, 3H), 7.10 – 6.95 (m, 2H), 6.90 – 6.80 (m, 2H), 6.33 (s, 1H), 2.73 – 2.60 (m, 4H), 2.47 (d, *J* = 13.2 Hz, 1H), 2.29 (s, 3H, ArCH_3), 2.25 (s, 6H, 2×ArCH_3), 2.08 (s, 3H, ArCH_3), 2.00 (s, 3H, ArCH_3), 1.68 – 1.56 (m, 2H), 1.45 – 1.26 (m, 4H), 1.22 – 1.02 (m, 3H), 0.79 (t, *J* = 6.9 Hz, 3H), 0.59 – 0.45 (m, 1H, H-12'/13').

^{13}C NMR (101 MHz, $CDCl_3$) δ 202.9 (C-11), 151.8 (ArC), 145.4 (ArC), 138.2 (ArC), 137.9 (ArC), 137.6 (ArC), 135.1 (ArC), 132.8 (ArC), 132.7 (ArC), 130.8 (ArC), 130.6 (ArC), 129.2 (ArC), 129.1 (ArC), 128.0 (ArC), 127.8 (ArC), 126.5 (ArC), 126.3 (ArC), 46.9 (C-12/C-13), 46.4 (C12/C-13), 42.1 (C-3), 31.8 (C-12/13), 30.9 (C-6), 25.3, 22.7, 21.8, 17.9 ($ArCH_3$), 17.5 ($ArCH_3$), 16.9 ($ArCH_3$), 16.2 ($ArCH_3$), 16.1 ($ArCH_3$), 14.1 (C-10).

IR (film) ν_{max}/cm^{-1}: 3028, 2931, 2359, 1652, 1496, 1218.

HRMS (ESI+): Found $[M+Na]^+$ = 515.3298; $C_{36}H_{44}O$ requires 515.3284 Δ 2.65 ppm.

$[\alpha]_D^{20}$ = - 16.8 (c = 0.2, $CHCl_3$).

To enable determination of the enantiomeric ratio of the title compound, a racemic sample was prepared following the same synthetic procedure.

HPLC: Enantiomeric excess was determined by HPLC with a Chiralpak® IA column (99.9:0.1 hexane: IPA, 1.0 ml min^{-1}, 254 nm, room temperature); t_r (minor) = 18.7 min, t_r (major) = 25.3 min, 98.5:1.5 e.r.

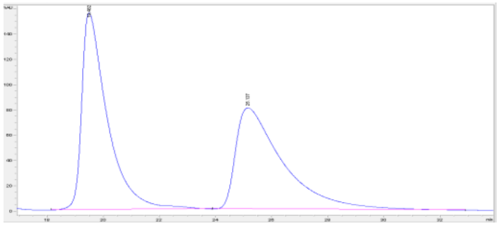

#	Time	Type	Area	Height	Width	Area%	Symmetry
1	19.482	BB	9914.1	155.7	0.907	51.402	0.377
2	25.137	BB	9373.2	80	1.6045	48.598	0.319

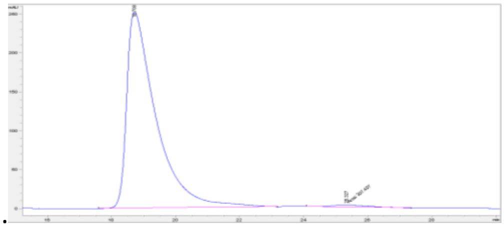

#	Time	Type	Area	Height	Width	Area%	Symmetry
1	18.708	BB	16051.2	252.8	0.8986	98.422	0.332
2	25.327	MM T	257.4	2.8	1.5396	1.578	0

4. Crystallographic data

Single crystal X-ray data collection and structure determination were performed by Akvile Mazeikaite in the Chemistry Research Laboratory, University of Oxford. Crystals were mounted on MiTeGen loops using perfluoropolyether oil and rapidly transferred to a goniometer head on a diffractometer fitted with an Oxford CryoSystems CryoStream open-flow nitrogen cooling device. [1] Data collections were either carried out at 100 K using a high flux Rigaku Synergy DW rotating anode diffractometer using mirror-monochromated Cu Kα radiation (λ = 1.54184 Å). Data were processed using CryAlisPro, the structure was then solved using charge-flipping algorithm SUPERFLIP[2] and refined by full-matrix least squares using CRYSTALS. [3, 4] Structures are visualised and represented using Mercury.

1. Cosier, J.; Glazer, A. M., J. Appl. Cryst. 1986, 19, 105-107.

2. Palatinus, L.; Chapuis, G., J. Appl. Cryst. 2007, 40, 786-790.

3. Cooper, R. I. T., A. L.; Watkin, D. J., J. Appl. Cryst. 2010, 43, 1100-1107.

4. Parois, P.; Cooper, R. I.; Thompson, A. L., Chem. Cent. J. 2015, 9 (1), 30.

Crystallographic data:

(*R*)-(6-(2-(benzyloxy)ethyl)cyclohex-1-en-1-yl)(2,3,4,5,6-pentamethylphenyl)methanone (4k)

Recrystallised via vapour diffusion using THF/EtOH

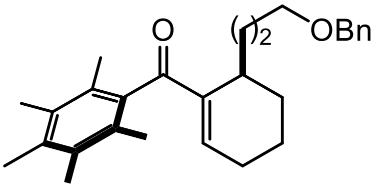

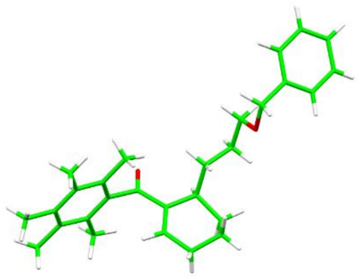

Formula C28 H36 O2

Crystal class	monoclinic	Space group	$P2_1$
a	8.4999(1)	alpha	90
b	31.6705(1)	beta	93.0936(4)
c	8.6223(1)	gamma	90
Volume	2317.71(4)	Z	4
Radiation type	Cu K\a	Wavelength	1.54184
Dx	1.159	Mr	404.57
Mu	0.543	Temperature (K)	100
Size	0.076 × 0.112 × 0.152		
Colour	Clear pale colourless	Shape	block
Cell from	70363 reflections	Theta range	3 to 76
Diffractometer type	multi-scan	Scan type	OMEGA
Absorption type	multi-scan	Transmission range	0.81 – 0.96
Rint	0.030	Independent reflections	9420
Hmin, Hmax	-10, 10	Theta max	75.96
Kmin, Kmax	-39, 36		
Lmin, Lmax	-10, 10		
Refinement on Fsqd			
R-factor	0.0308	Weighted R-factor	0.0792
Max shift/su	0.0007365		
Delta Rho min	-0.14	Delta Rho max	0.13
Reflections used	9085	sigma(I) limit	-3.00
Number of parameters	580	Goodness of fit	0.994
Flack parameter	0.03(4)		

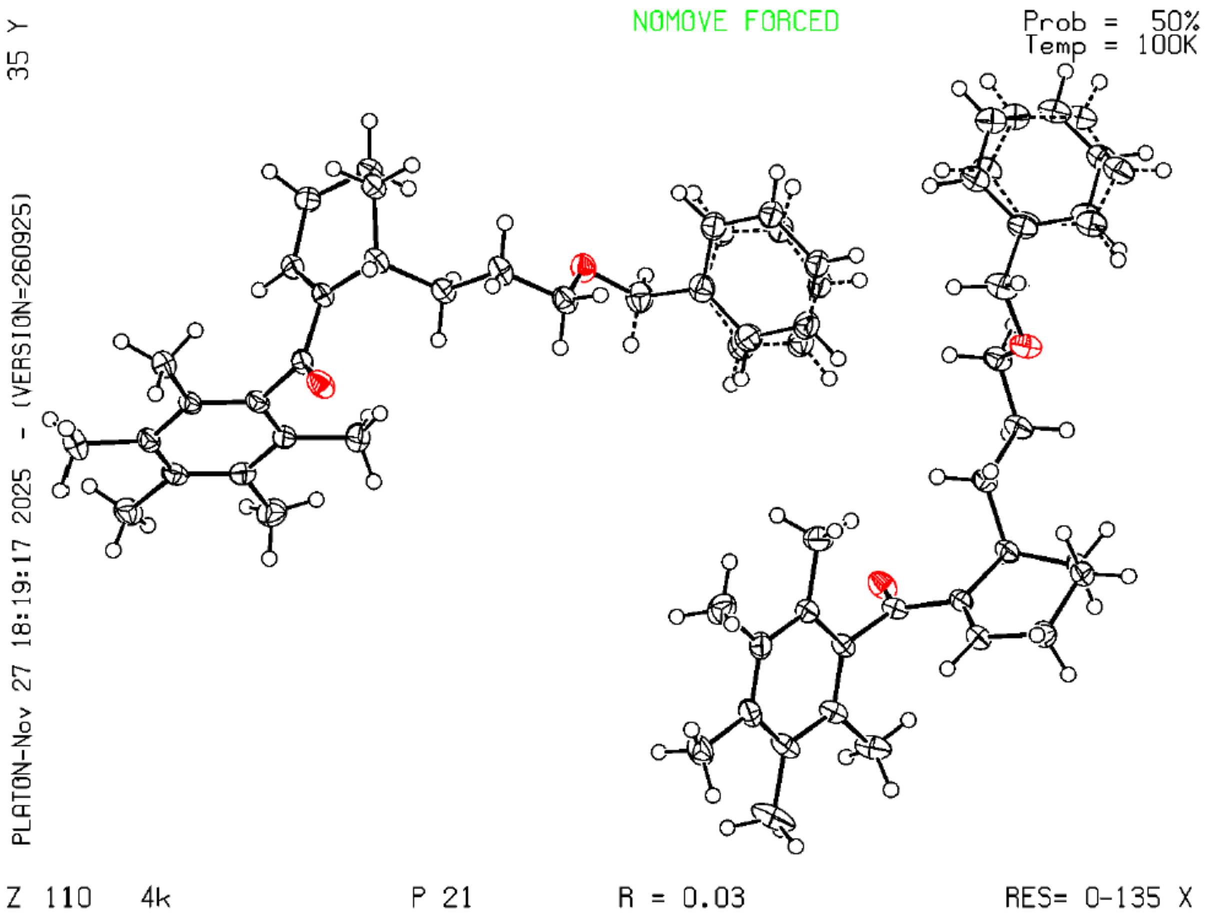

Thermal ellipsoids are drawn at the 50% probability level.

((3*S*,6*S*)-3-bromo-6-butylcyclohex-1-en-1-yl)(2,3,4,5,6-pentamethylphenyl)methanone (7)

Recrystallised via vapour diffusion using CH_2Cl_2/ DMSO-EtOH (9:1)

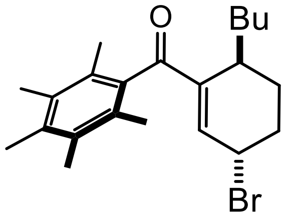

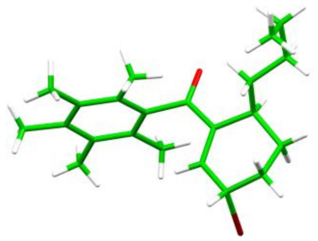

Formula C22 H31 Br O

Crystal class	orthorhombic	Space group	$P2_12_12_1$
a	7.3986(1)	alpha	90
b	8.6217(1)	beta	90
c	31.2132(2)	gamma	90
Volume	1991.04(4)	Z	4
Radiation type	Cu K\a	Wavelength	1.54184
Dx	1.306	Mr	391.39
Mu	2.833	Temperature (K)	100
Size	0.014 × 0.071 × 0.086		
Colour	Clear pale colourless	Shape	plate
Cell from	75898 reflections	Theta range	3 to 76
Diffractometer type	multi-scan	Scan type	OMEGA
Absorption type	multi-scan	Transmission range	0.74 – 0.96
Rint	0.063	Independent reflections	4132
Hmin, Hmax	-9, 9	Theta max	76.078
Kmin, Kmax	-10, 9		
Lmin, Lmax	-38, 39		
Refinement on Fsqd			
R-factor	0.0351	Weighted R-factor	0.0965
Max shift/su	0.0007464		
Delta Rho min	-0.33	Delta Rho max	0.62
Reflections used	4132	sigma(I) limit	-3.00
Number of parameters	218	Goodness of fit	1.0017
Flack parameter	-0.021(5)		

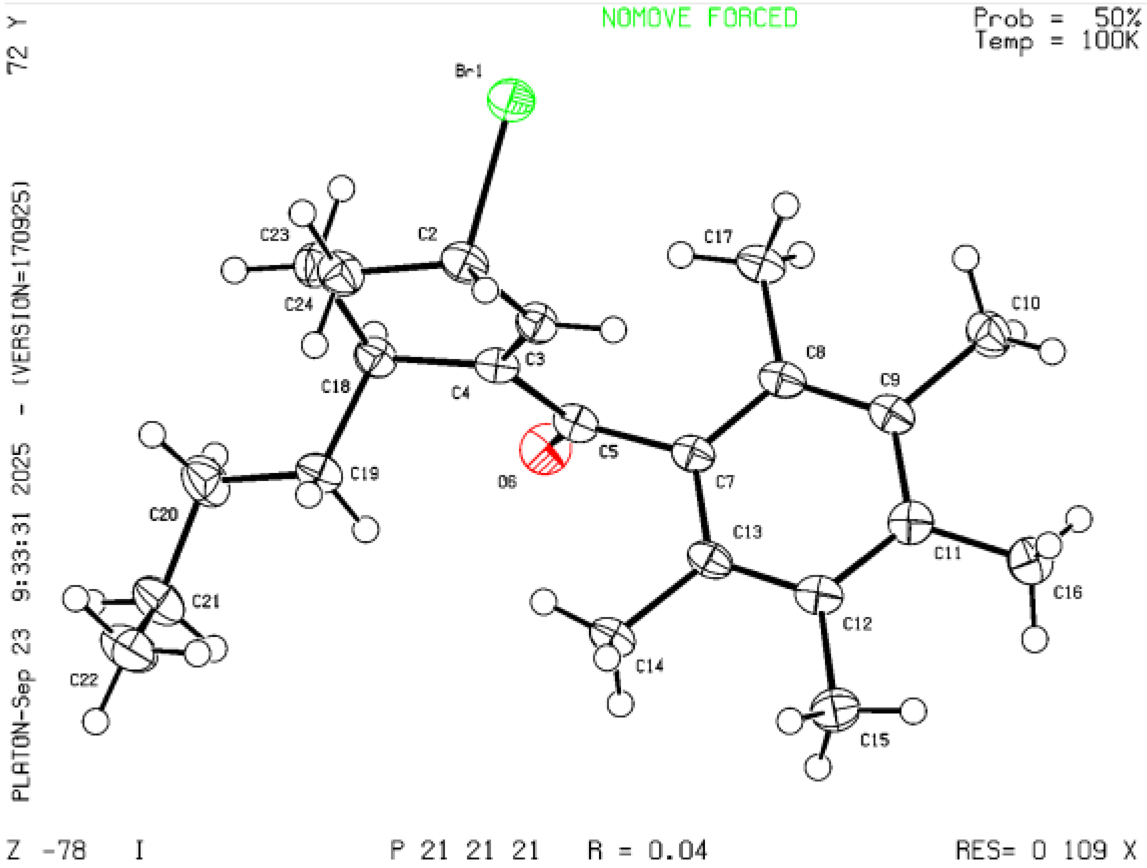

Thermal ellipsoids are drawn at the 50% probability level.
