# Supplementary material for: Asymmetric Hydride Shift Reactions Catalyzed by Chiral Aluminium Complexes
Source: Angew Chem Int Ed Engl. 2025 Dec 18;65(5):e21374. doi: 10.1002/anie.202521374 (PMC12850996; doi:10.1002/anie.202521374)
Supplement: Supplementary file 2 — Supporting Information [file ANIE-65-e21374-s002.pdf]

NMR Spectra

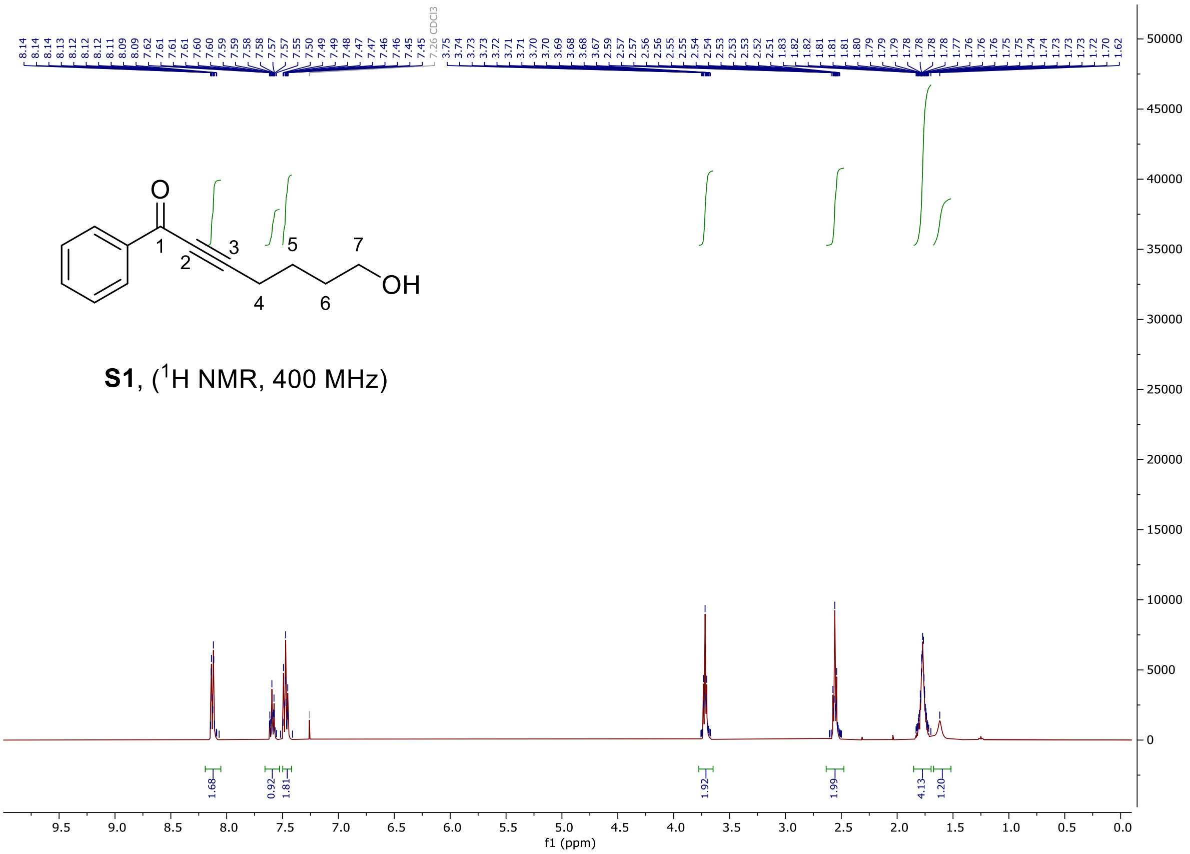

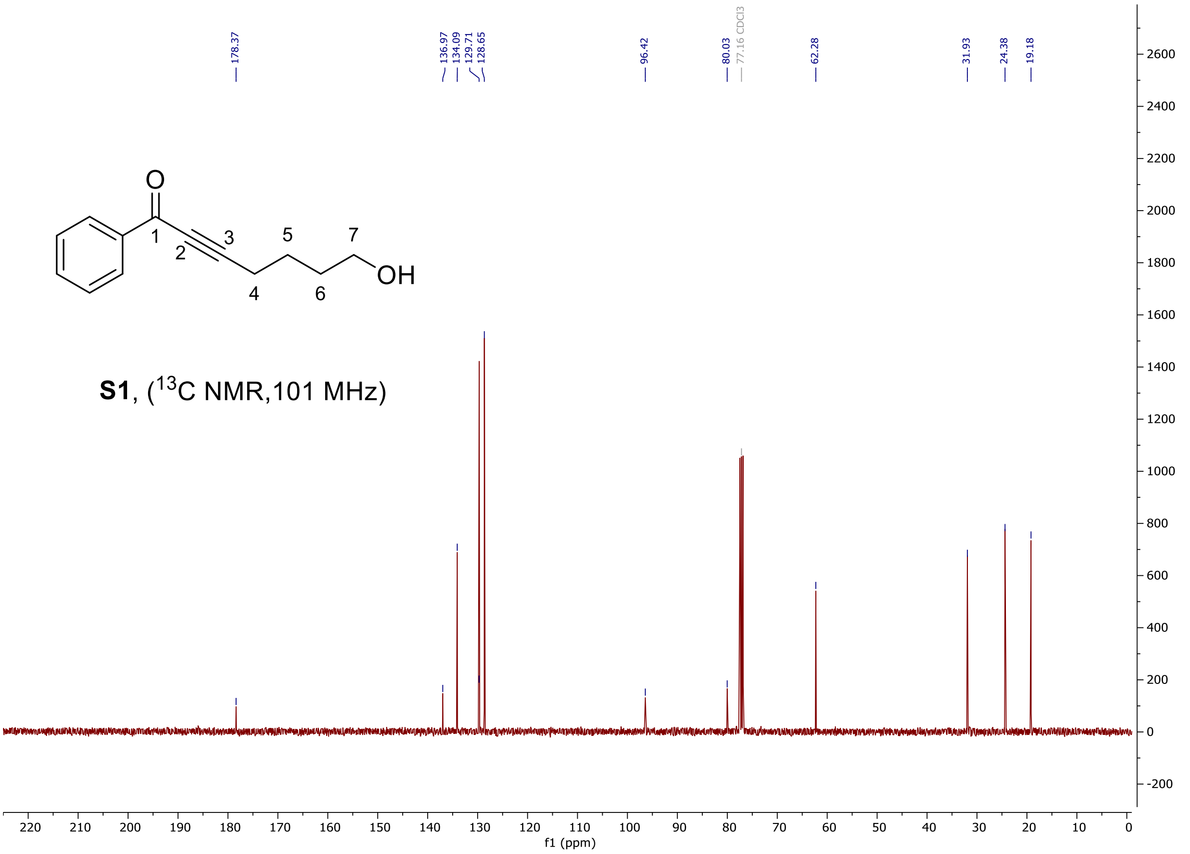

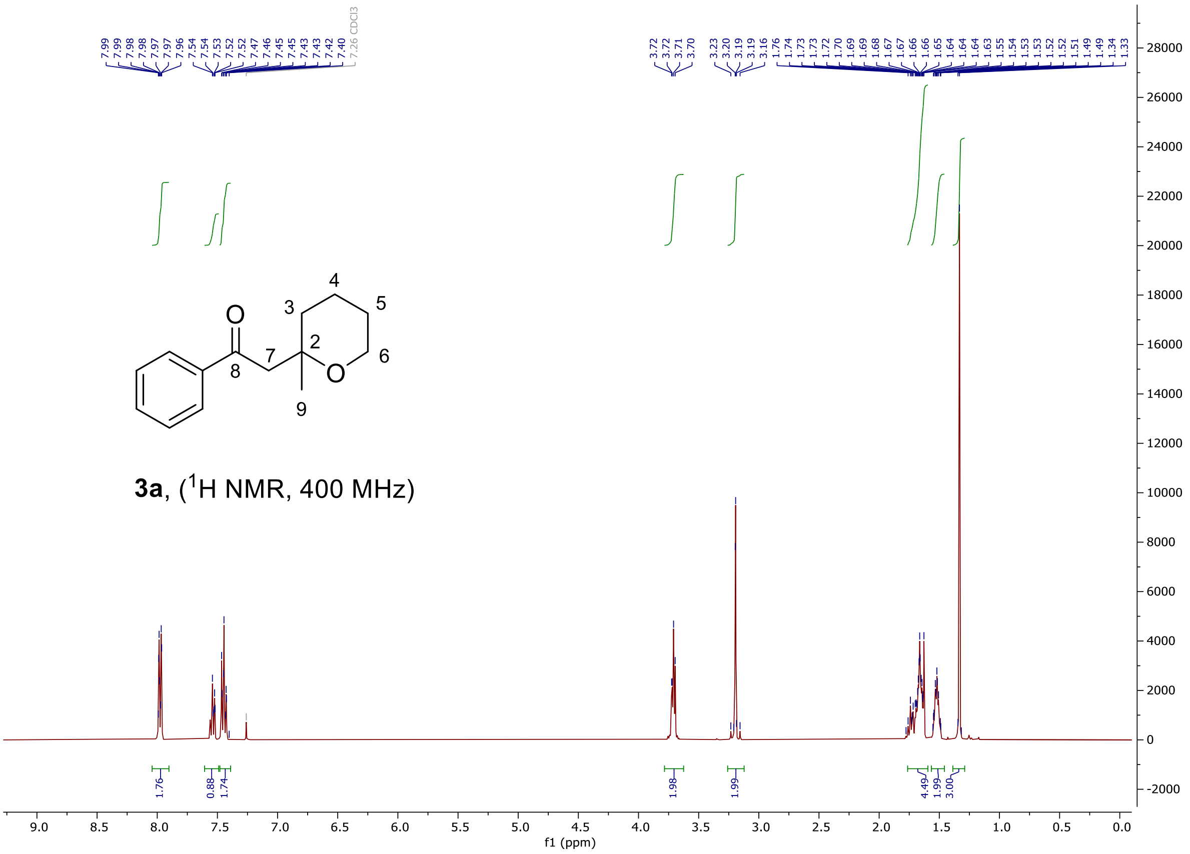
3a, (^{1}H NMR, 400 MHz)
f1 (ppm)

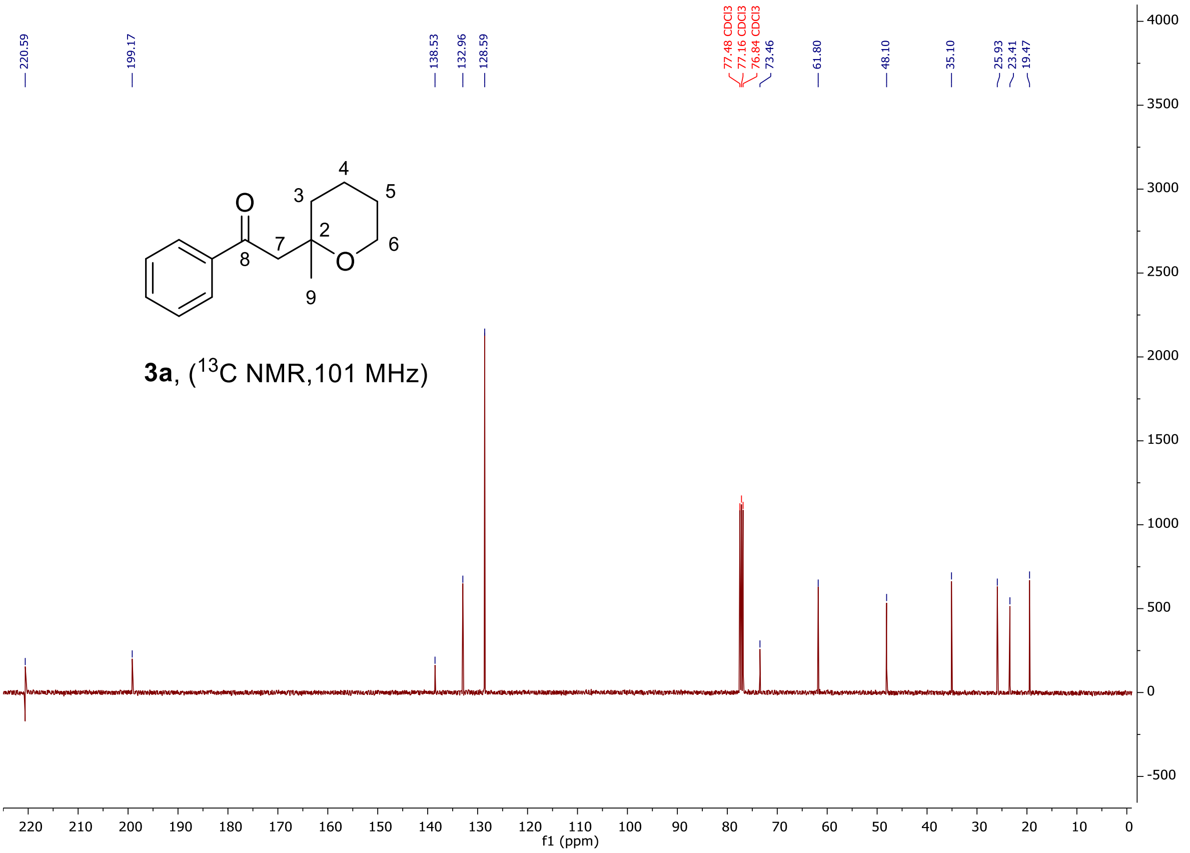
220.59
199.17
138.53
132.96
128.59
77.48 CDCl3
77.16 CDCl3
76.84 CDCl3
73.46
61.80
48.10
35.10
25.93
23.41
19.47
3a, (^{13}C NMR,101 MHz)
f1 (ppm)

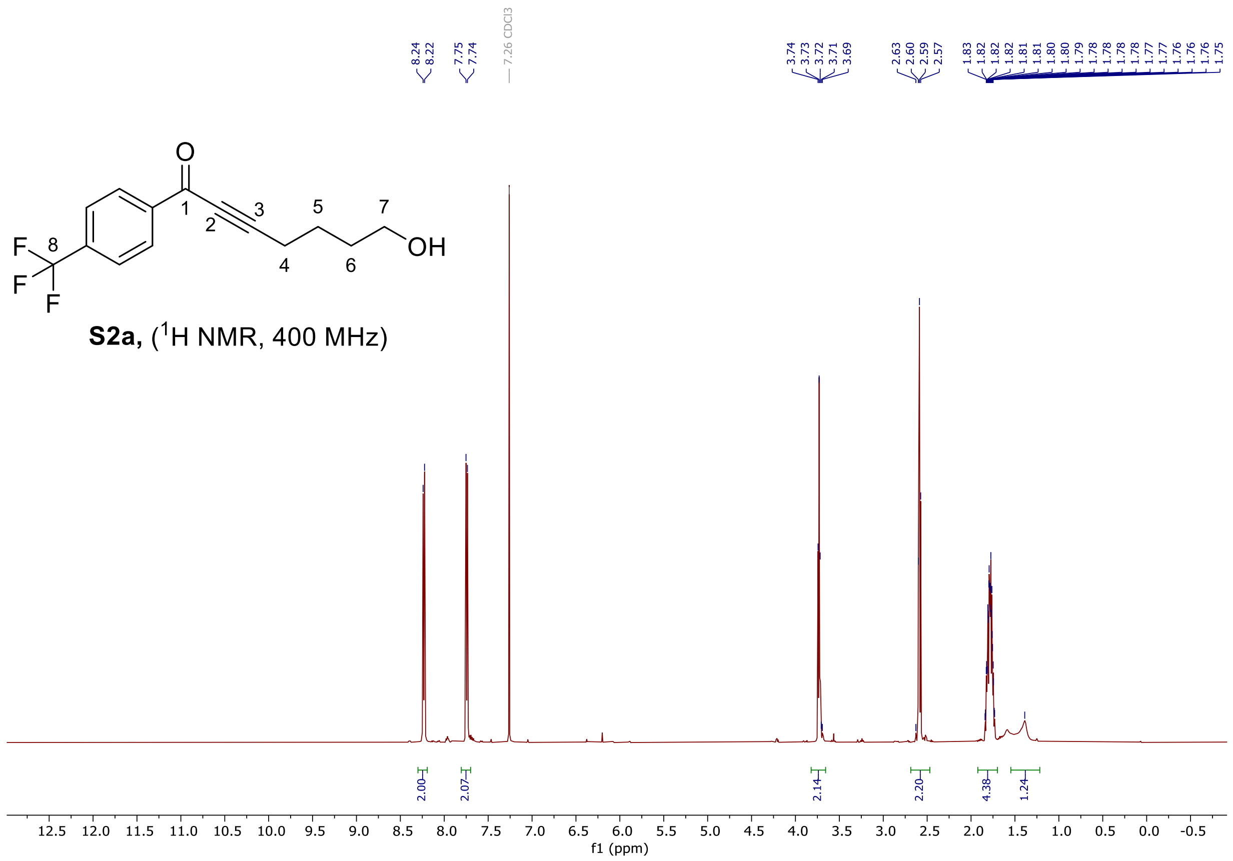
8.24
8.22
7.75
7.74
7.26 CDCl3
3.74
3.73
3.72
3.71
3.69
2.63
2.60
2.59
2.57
1.83
1.82
1.82
1.82
1.81
1.81
1.80
1.80
1.79
1.78
1.78
1.78
1.78
1.77
1.77
1.76
1.76
1.75
S2a, (1H NMR, 400 MHz)
2.00
2.07
2.14
2.20
4.38
1.24
f1 (ppm)

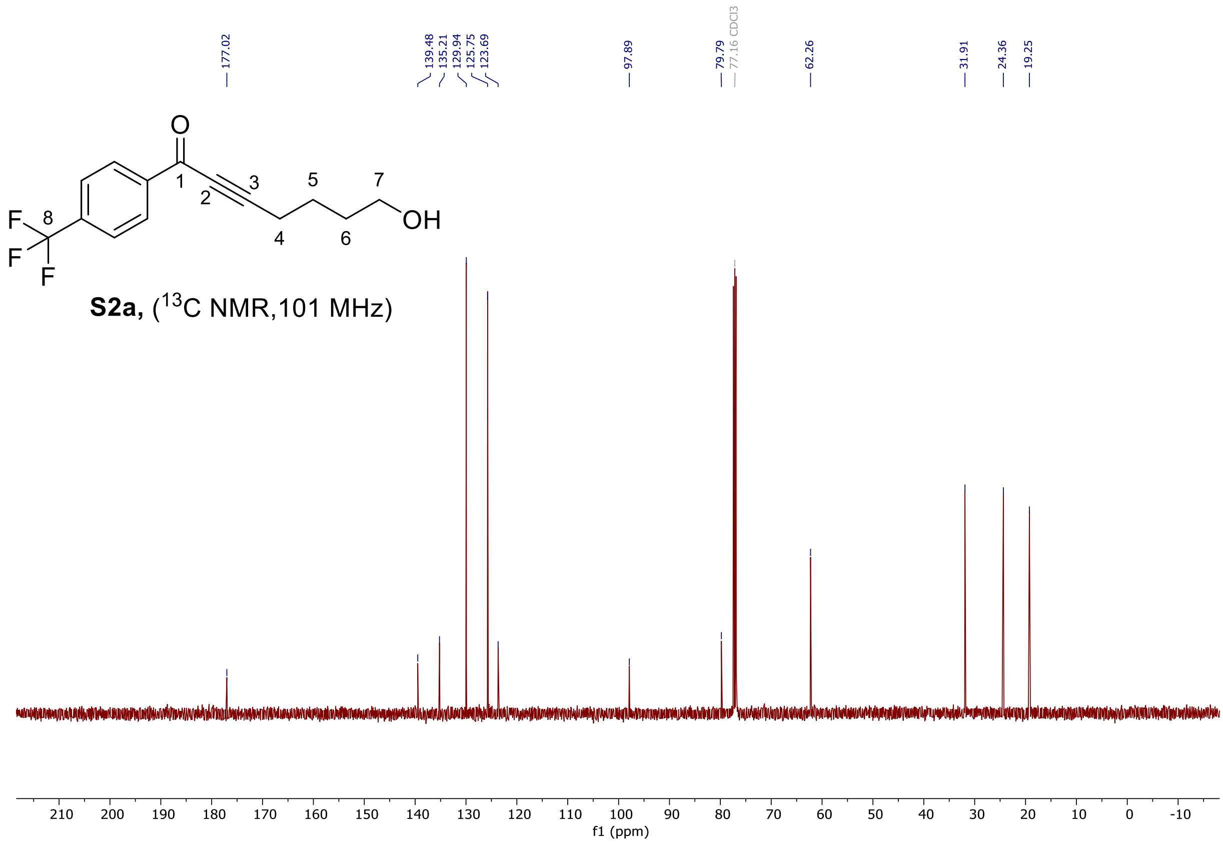
177.02
139.48
135.21
129.94
125.75
123.69
97.89
79.79
77.16 CDCl3
62.26
31.91
24.36
19.25
S2a, (13C NMR,101 MHz)
f1 (ppm)

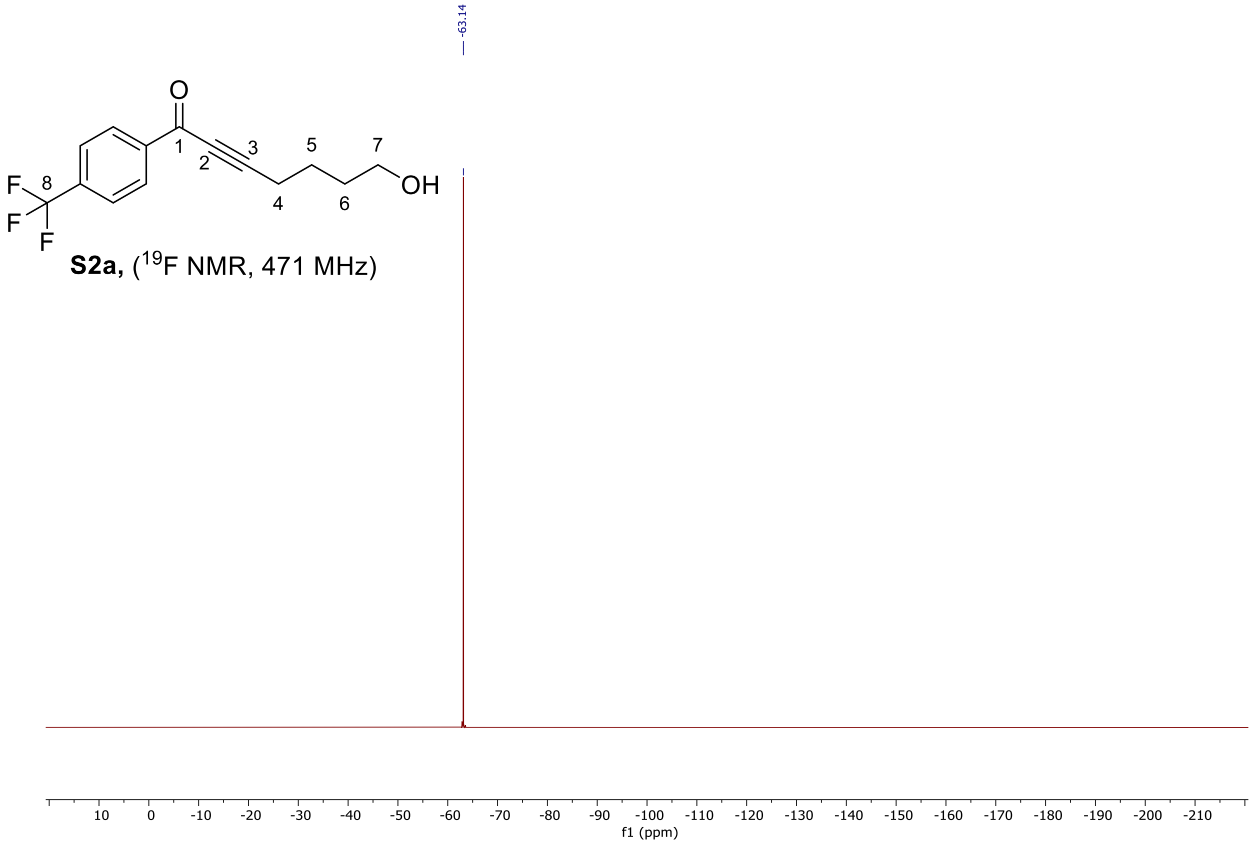

S2a, (^{19}F NMR, 471 MHz)

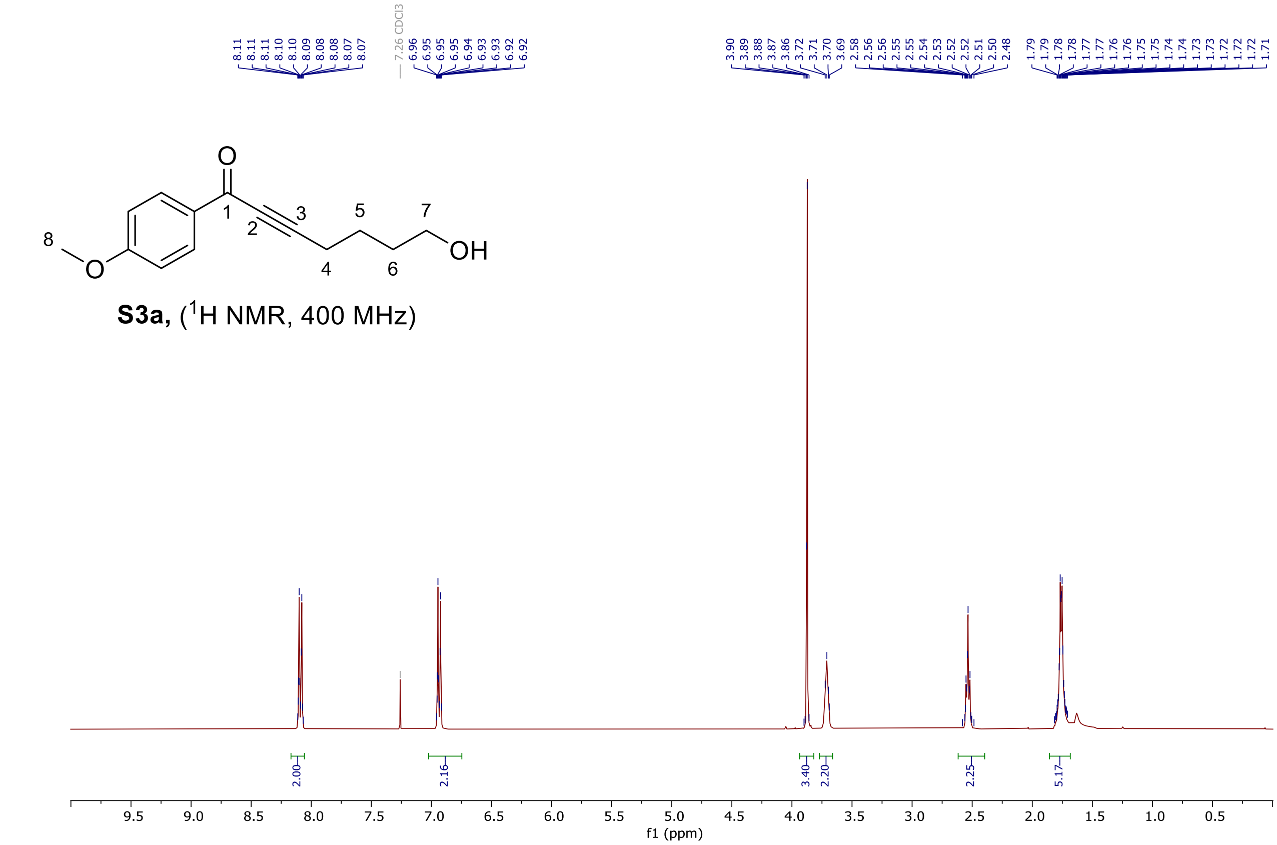

S3a, (1H NMR, 400 MHz)
7.26 CDCl3
f1 (ppm)

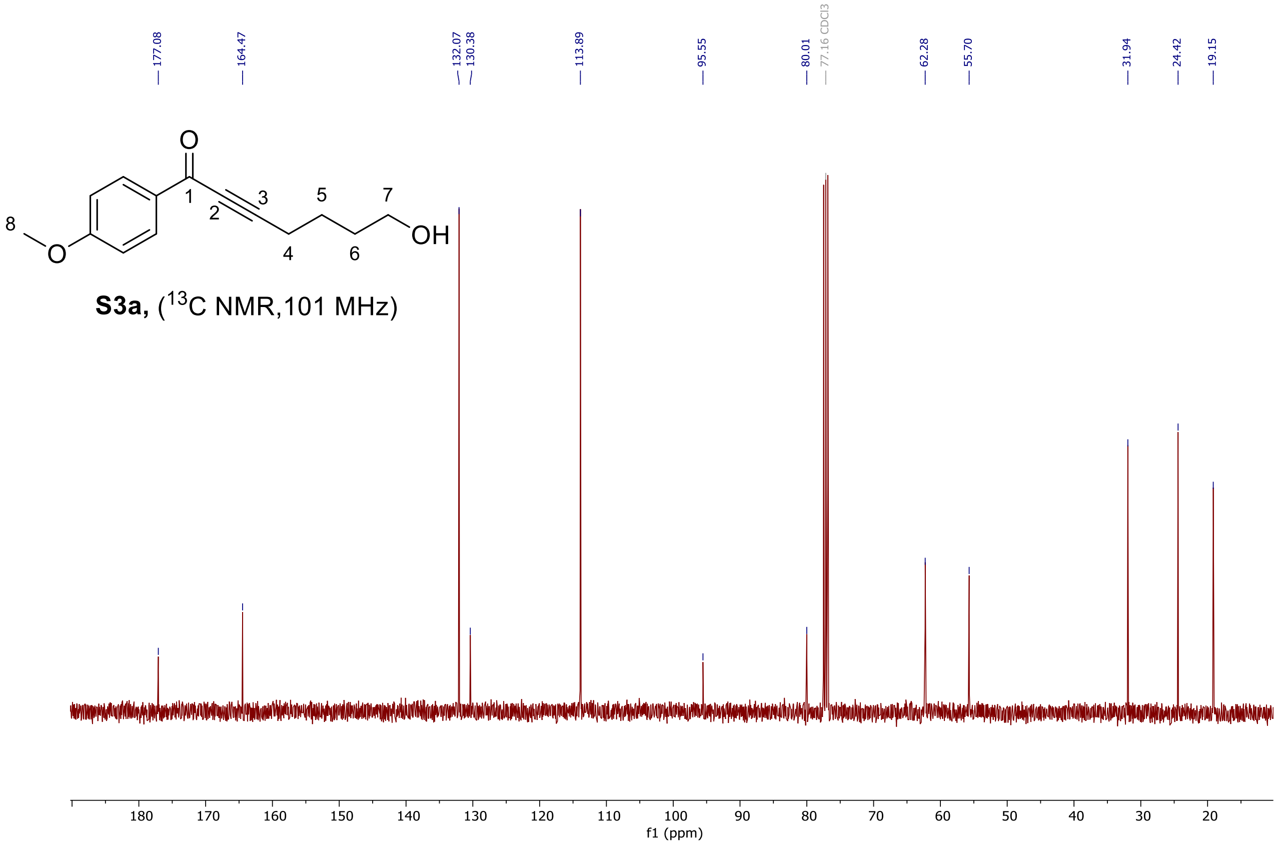

S3a, (13C NMR,101 MHz)
177.08
164.47
132.07
130.38
113.89
95.55
80.01
77.16 CDCl3
62.28
55.70
31.94
24.42
19.15
f1 (ppm)

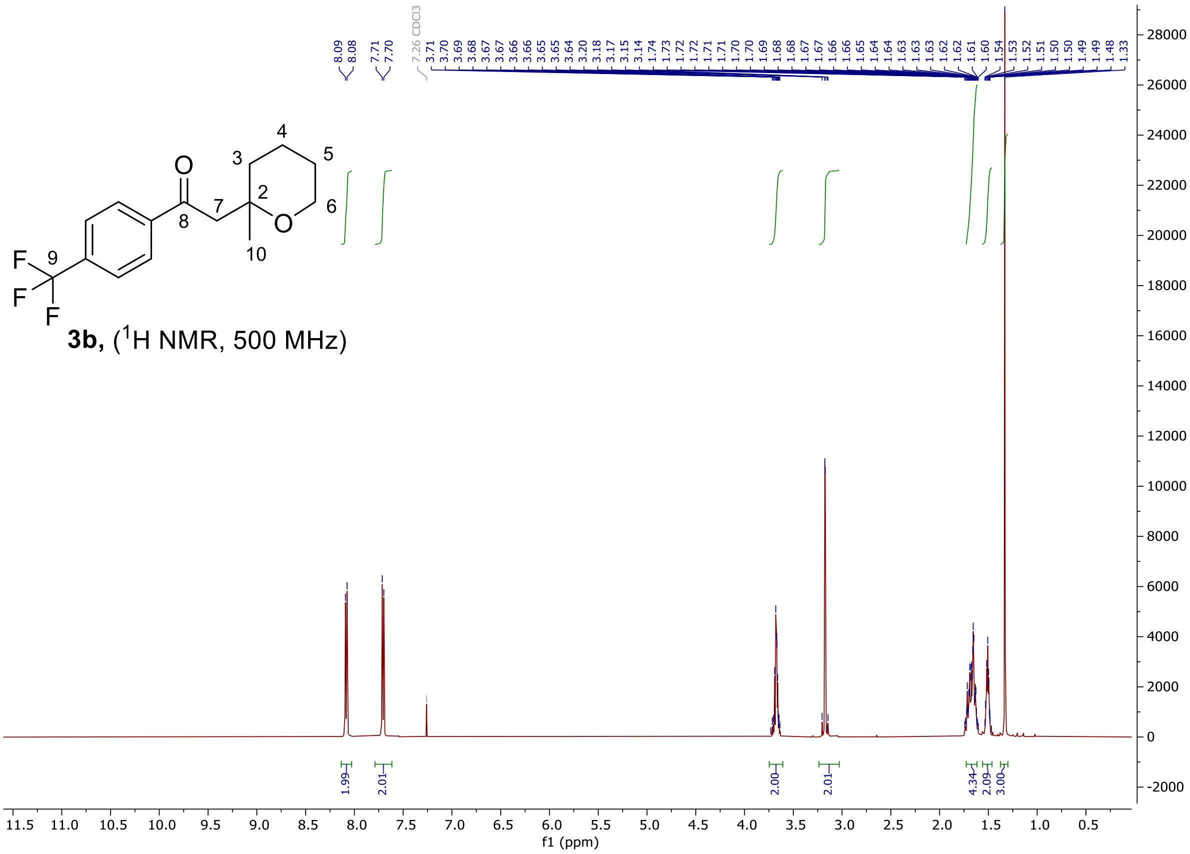

3b, (^{1}H NMR, 500 MHz)

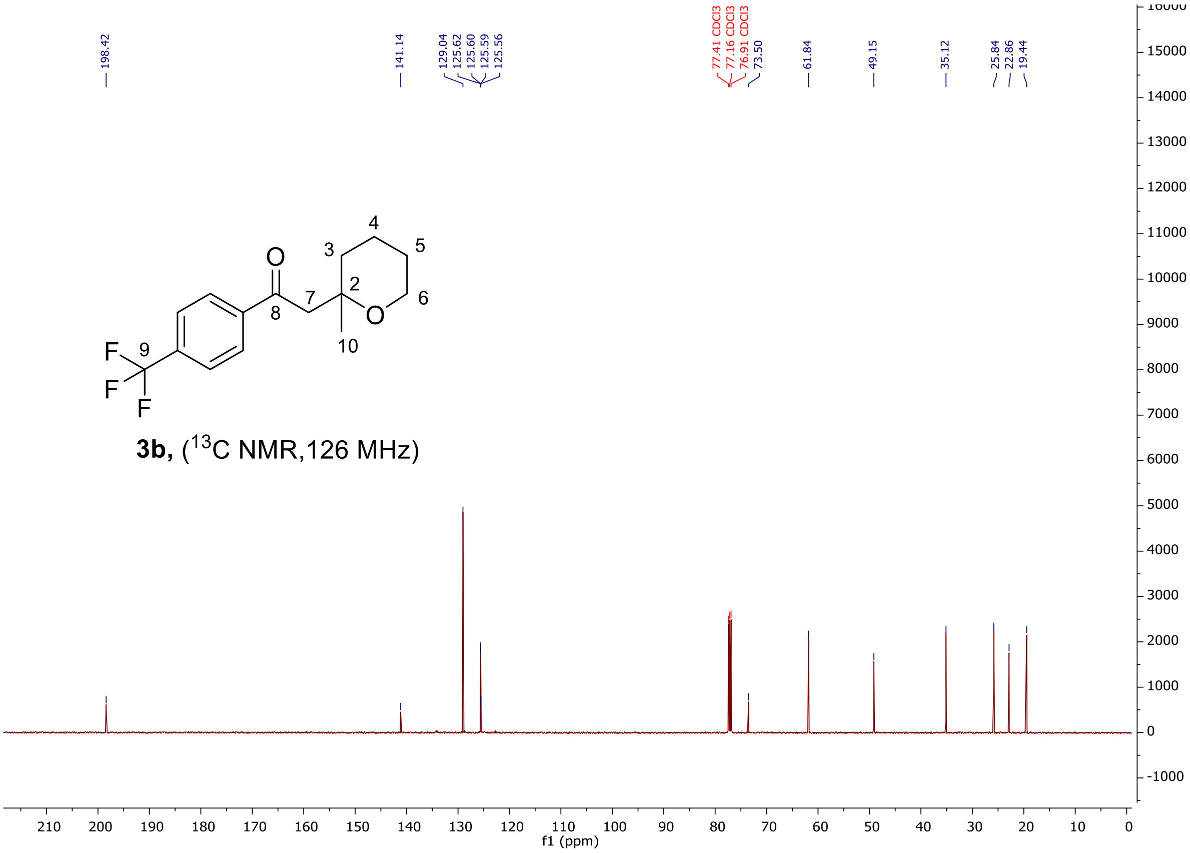

3b, (^{13}C NMR,126 MHz)

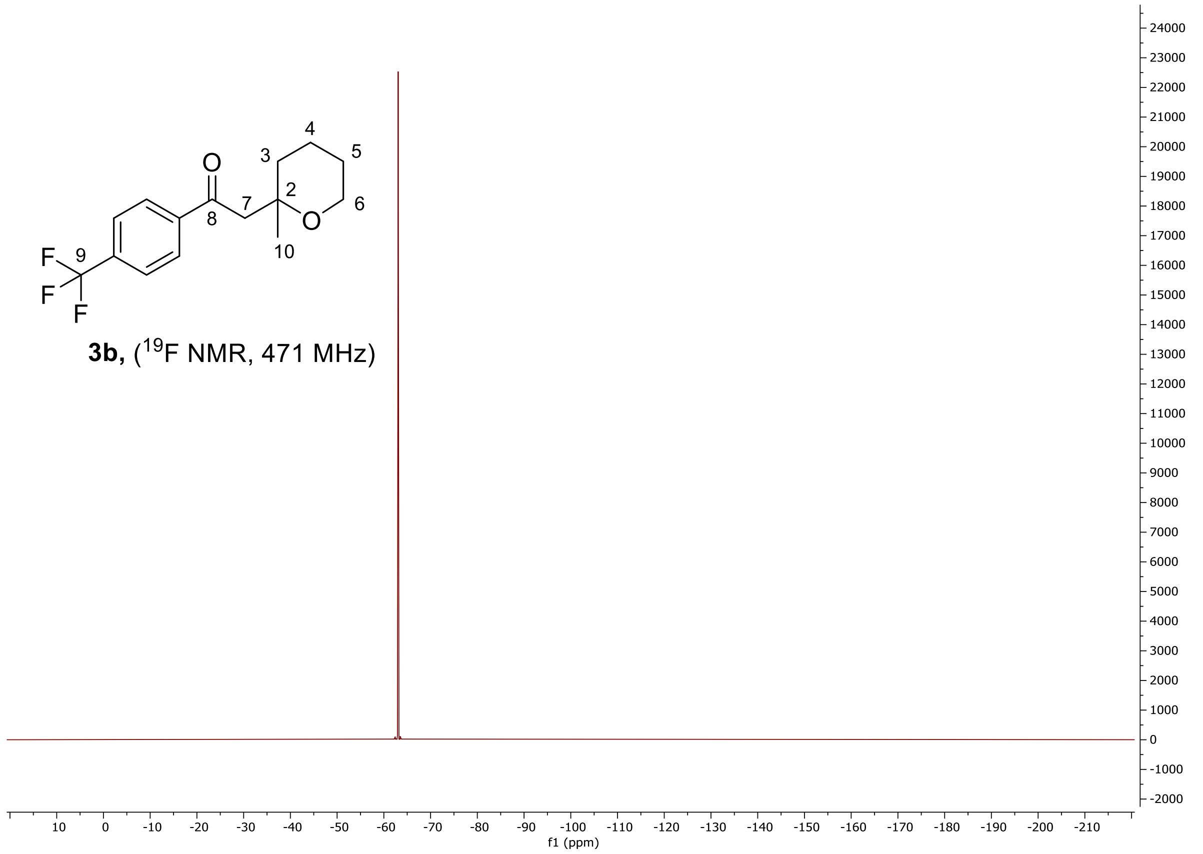
3b, (^{19}F NMR, 471 MHz)
f1 (ppm)

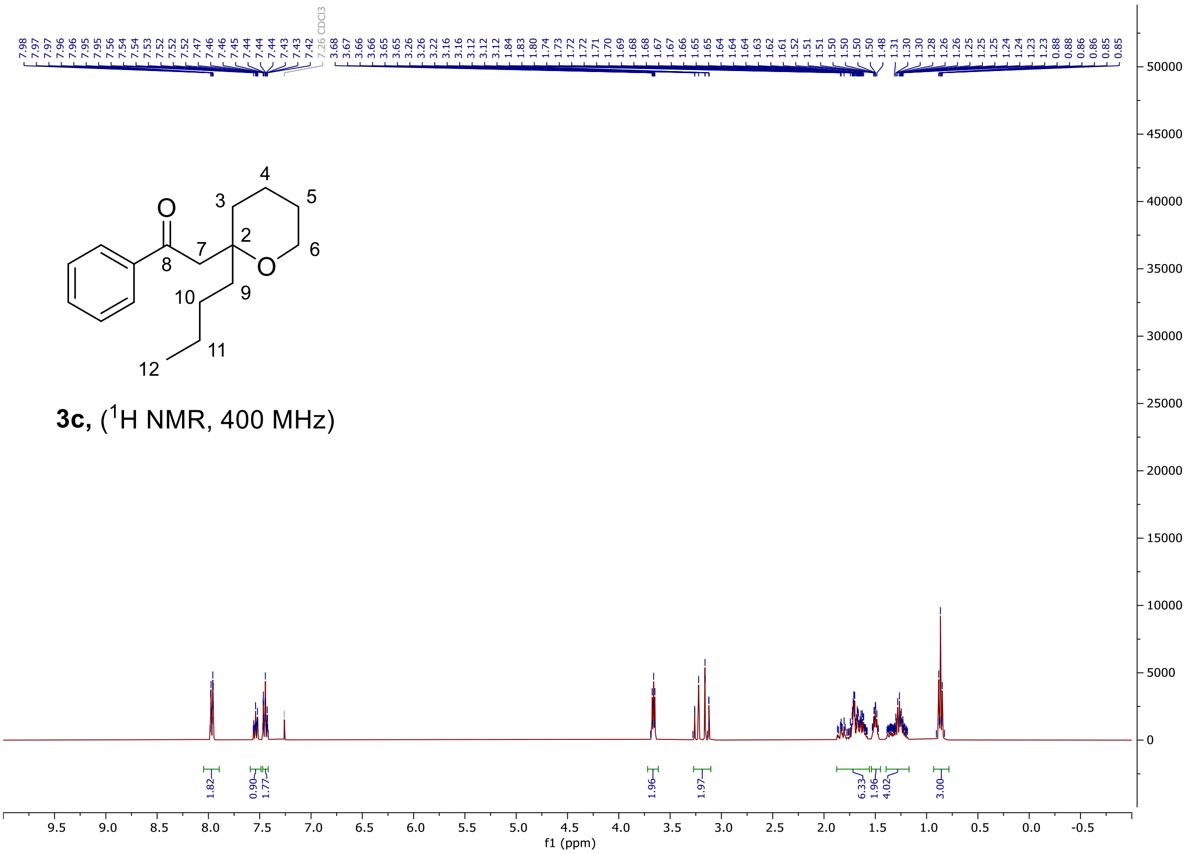
3c, (1H NMR, 400 MHz)
f1 (ppm)

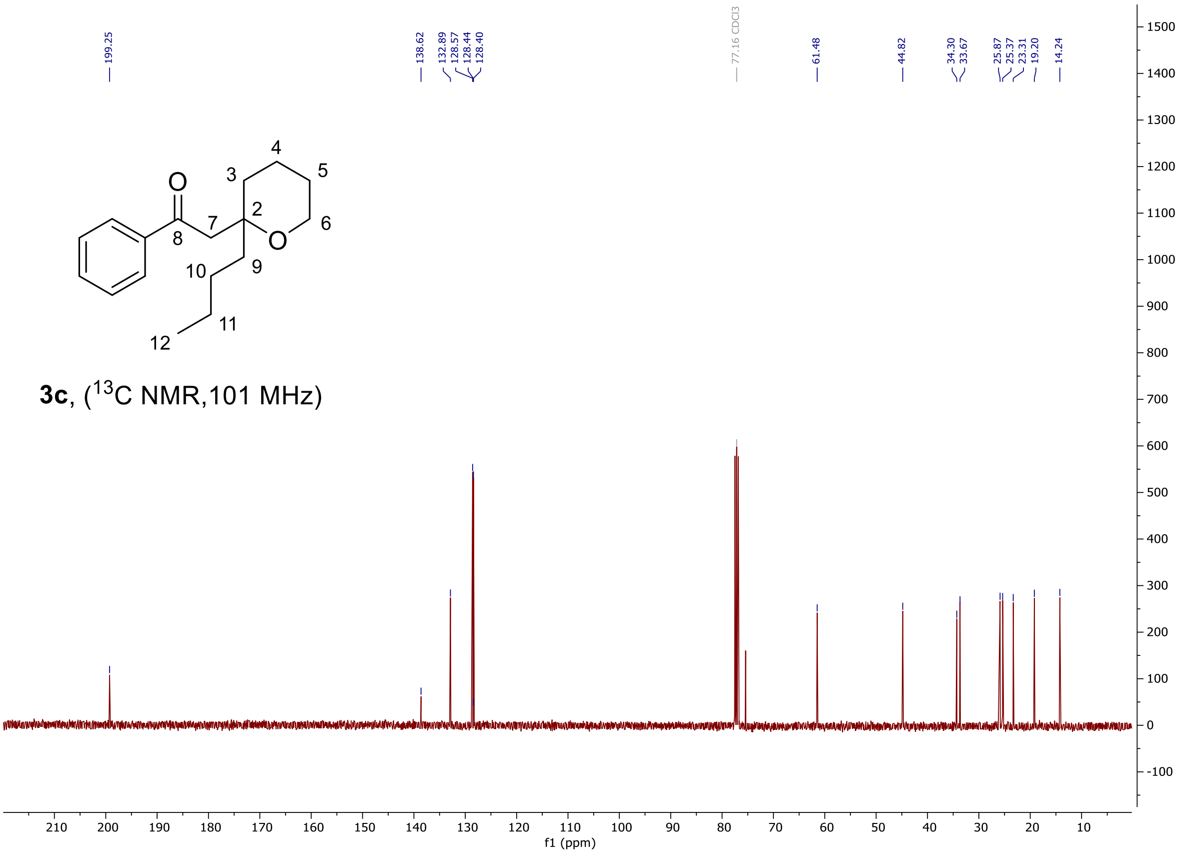
3c, (13C NMR,101 MHz)
199.25
138.62
132.89
128.57
128.44
128.40
77.16 CDCl3
61.48
44.82
34.30
33.67
25.87
25.37
23.31
19.20
14.24
f1 (ppm)

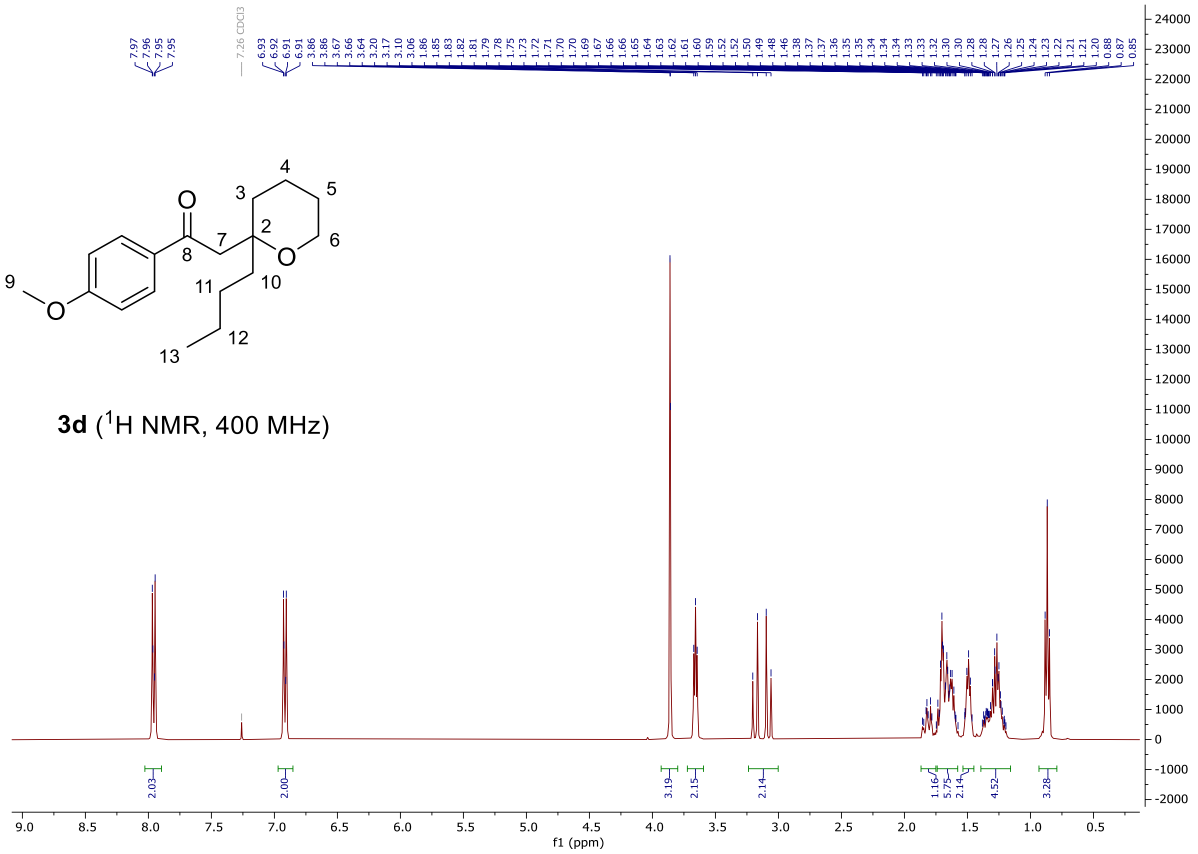
3d (¹H NMR, 400 MHz)
f1 (ppm)

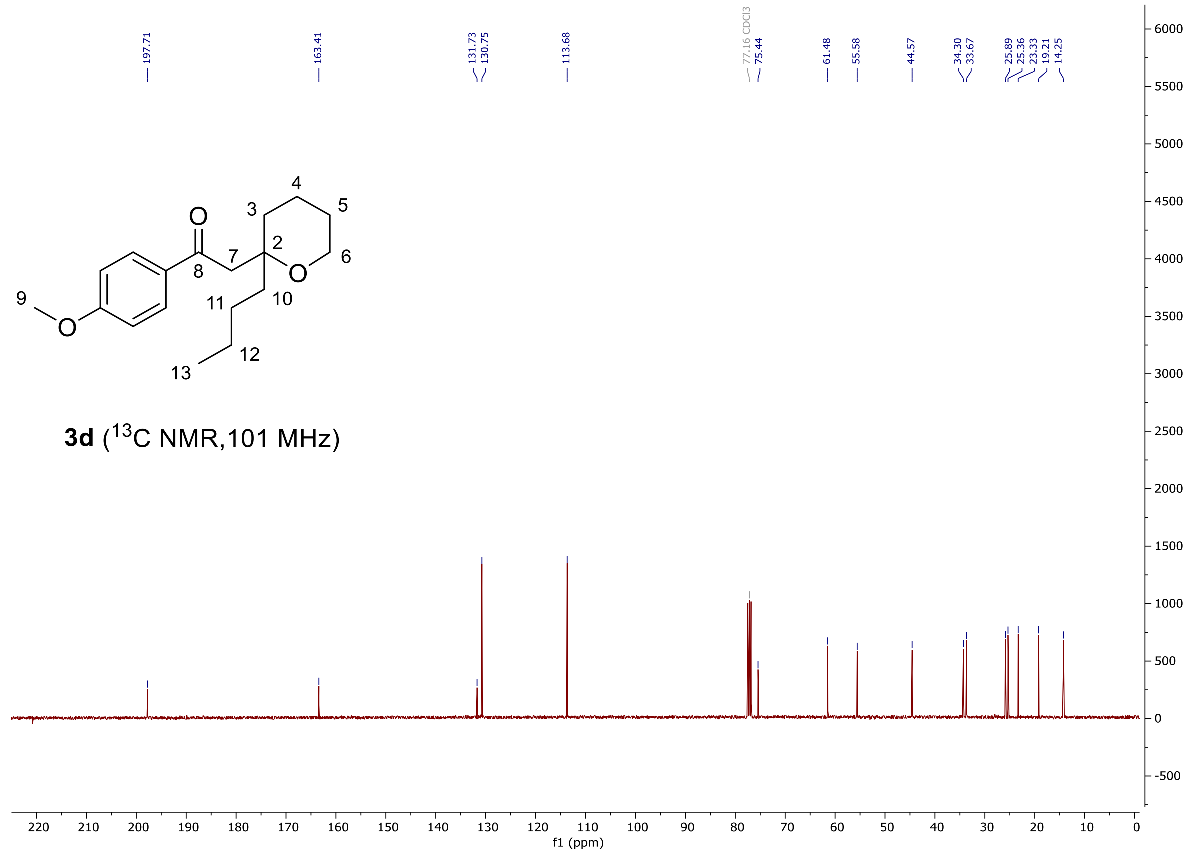
3d (¹³C NMR,101 MHz)
f1 (ppm)

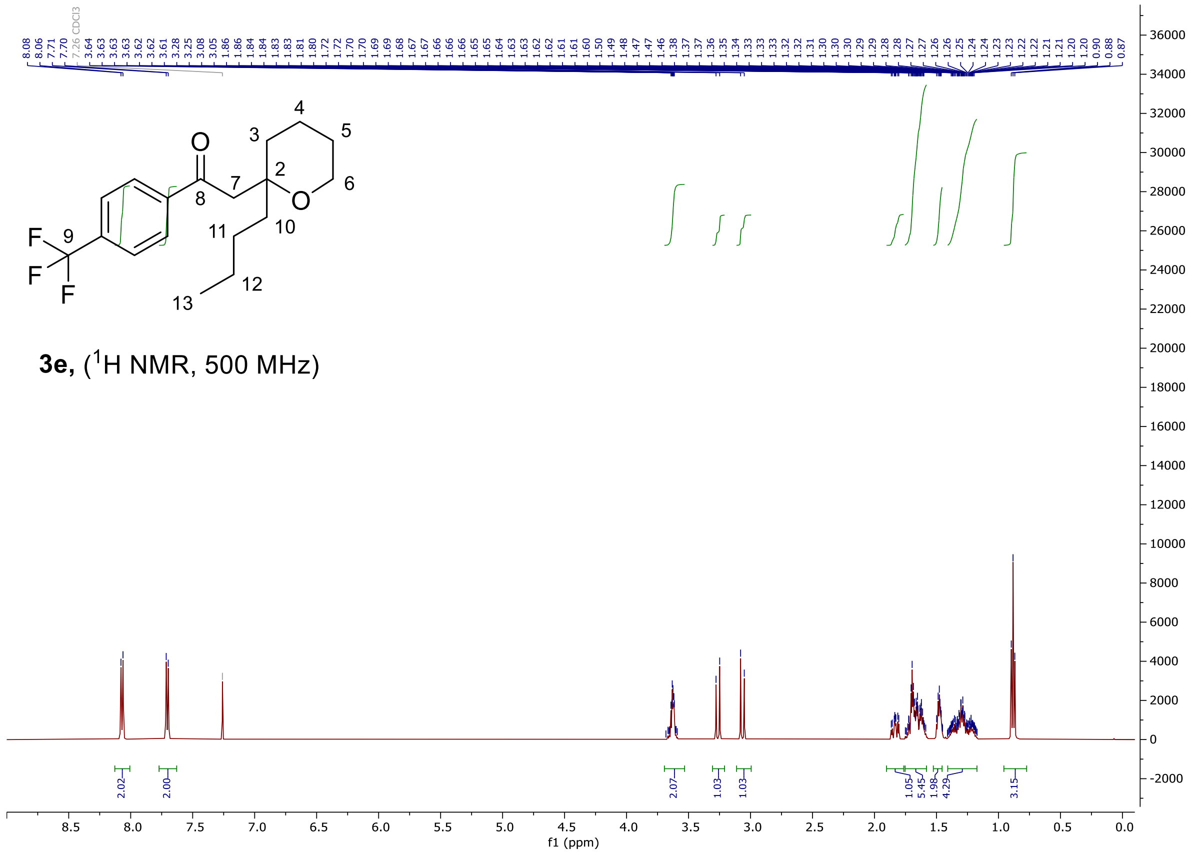
3e, (1H NMR, 500 MHz)
f1 (ppm)

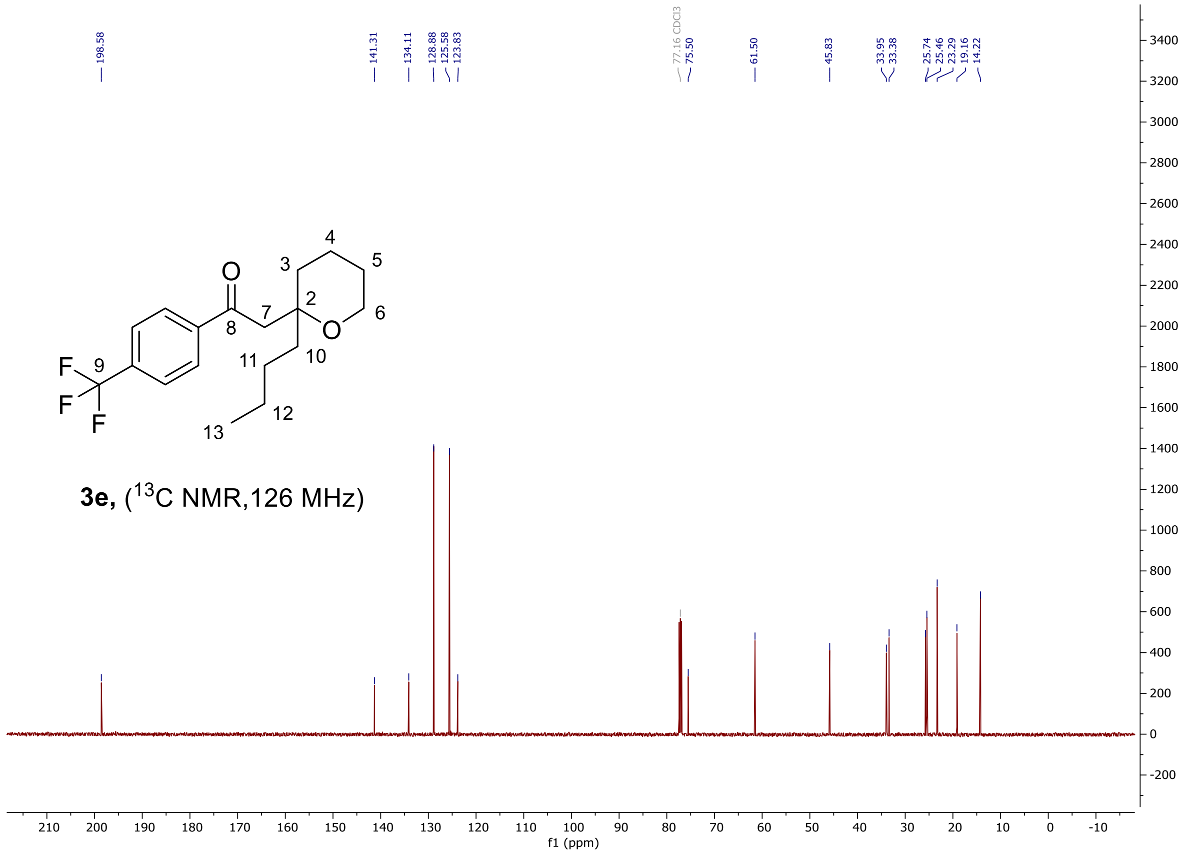
3e, (13C NMR,126 MHz)
198.58
141.31
134.11
128.88
125.58
123.83
77.16 CDCl3
75.50
61.50
45.83
33.95
33.38
25.74
25.46
23.29
19.16
14.22
f1 (ppm)

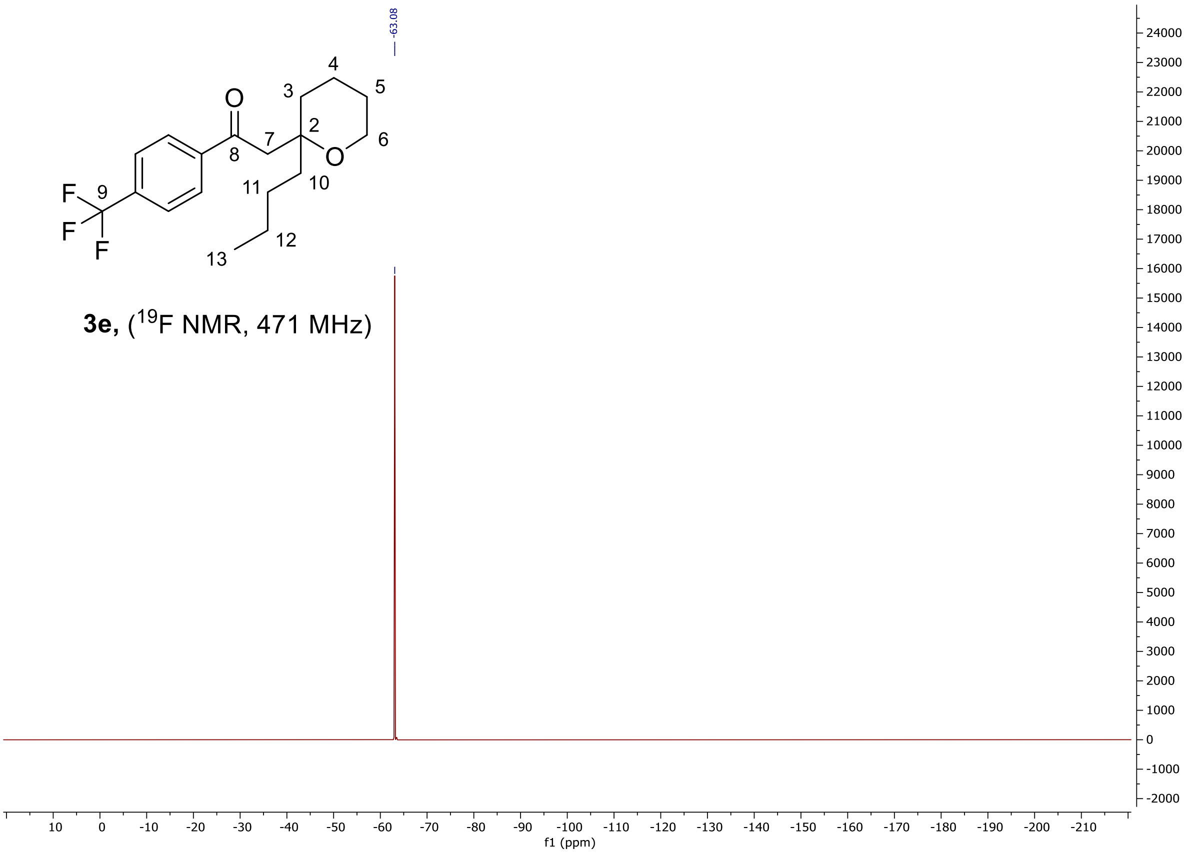

3e, (^{19}F NMR, 471 MHz)

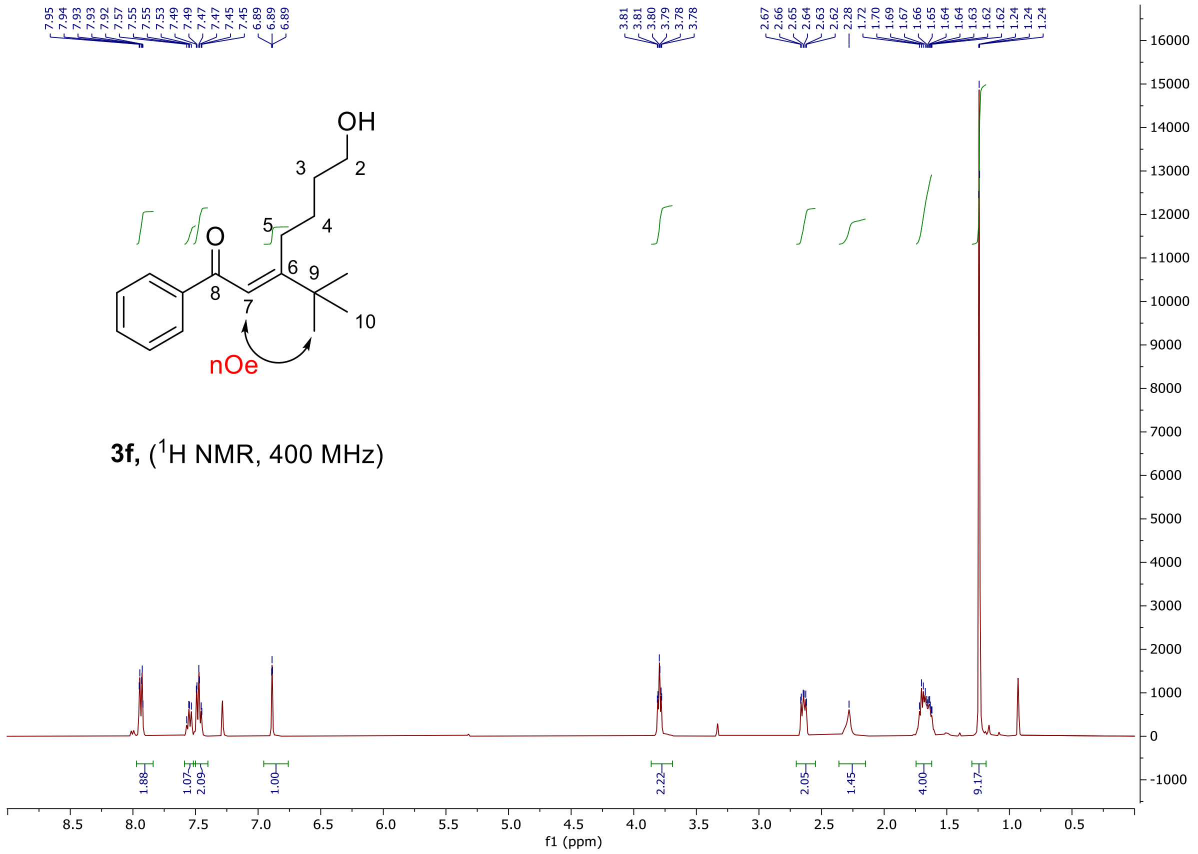
7.95 7.94 7.93 7.93 7.92 7.57 7.55 7.55 7.53 7.49 7.49 7.47 7.47 7.45 7.45 6.89 6.89 6.89
3.81 3.81 3.80 3.79 3.78 3.78
2.67 2.66 2.65 2.64 2.63 2.62 2.28 1.72 1.70 1.69 1.67 1.66 1.65 1.64 1.64 1.63 1.62 1.62 1.24 1.24 1.24
OH
nOe
3f, (1H NMR, 400 MHz)
1.88 1.07 2.09 1.00 2.22 2.05 1.45 4.00 9.17
f1 (ppm)

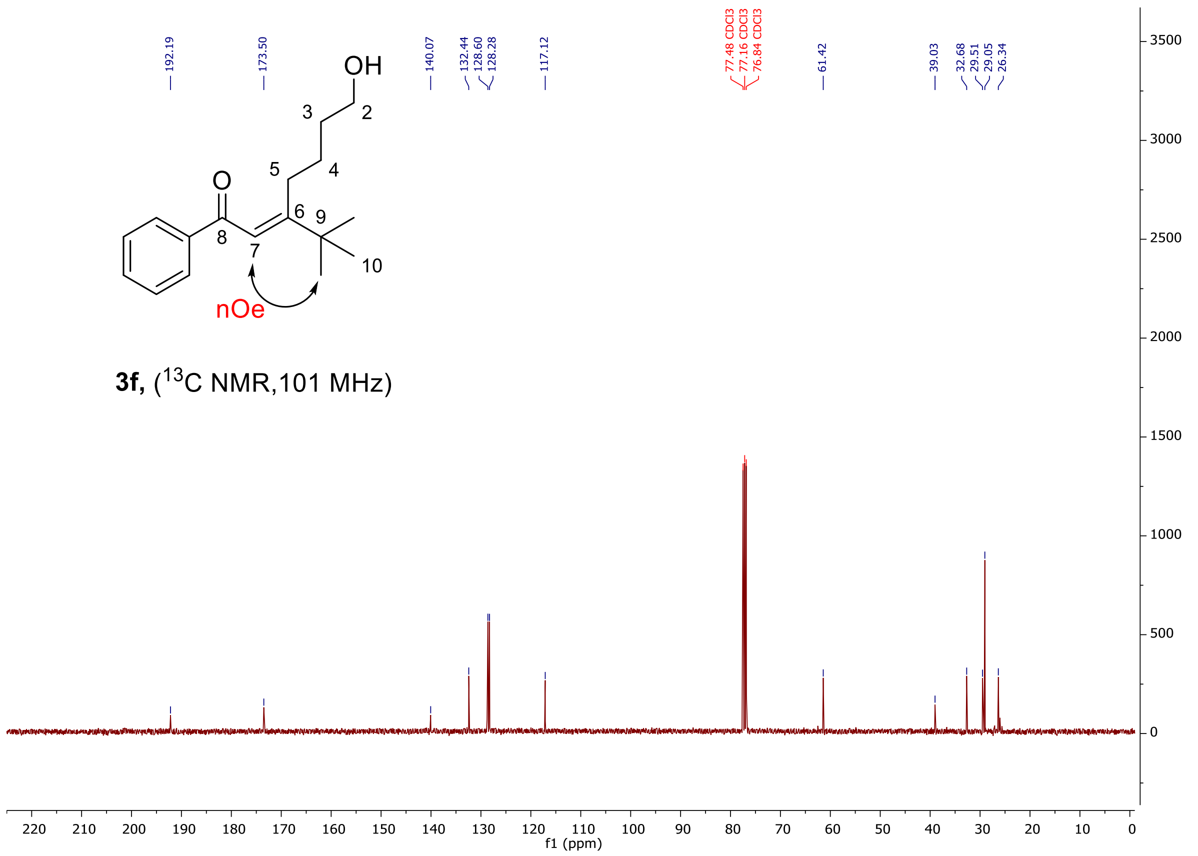
192.19 173.50 140.07 132.44 128.60 128.28 117.12
77.48 CDCl3 77.16 CDCl3 76.84 CDCl3
61.42 39.03 32.68 29.51 29.05 26.34
OH
nOe
3f, (13C NMR,101 MHz)
f1 (ppm)

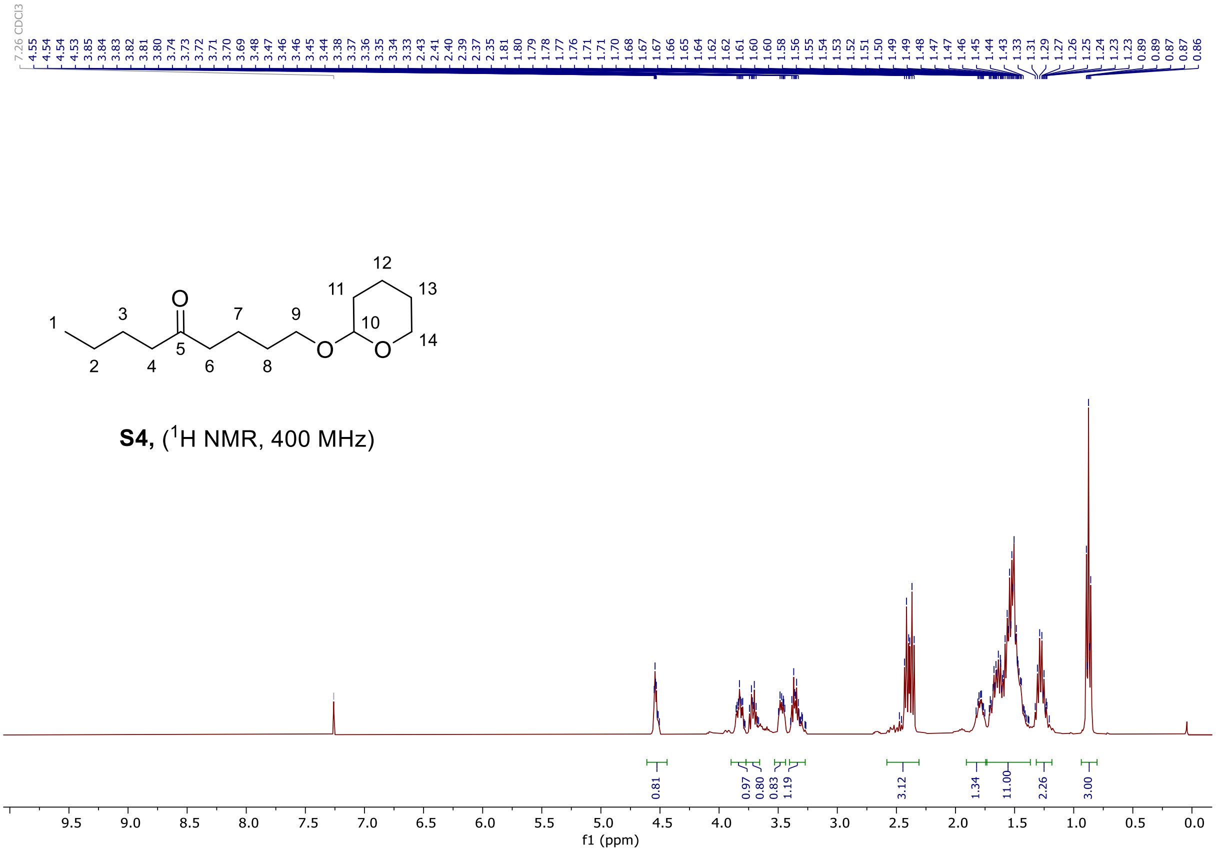
7.26 CDCl3
S4, (^{1}H NMR, 400 MHz)
0.81
0.97
0.80
0.83
1.19
3.12
1.34
11.00
2.26
3.00
f1 (ppm)

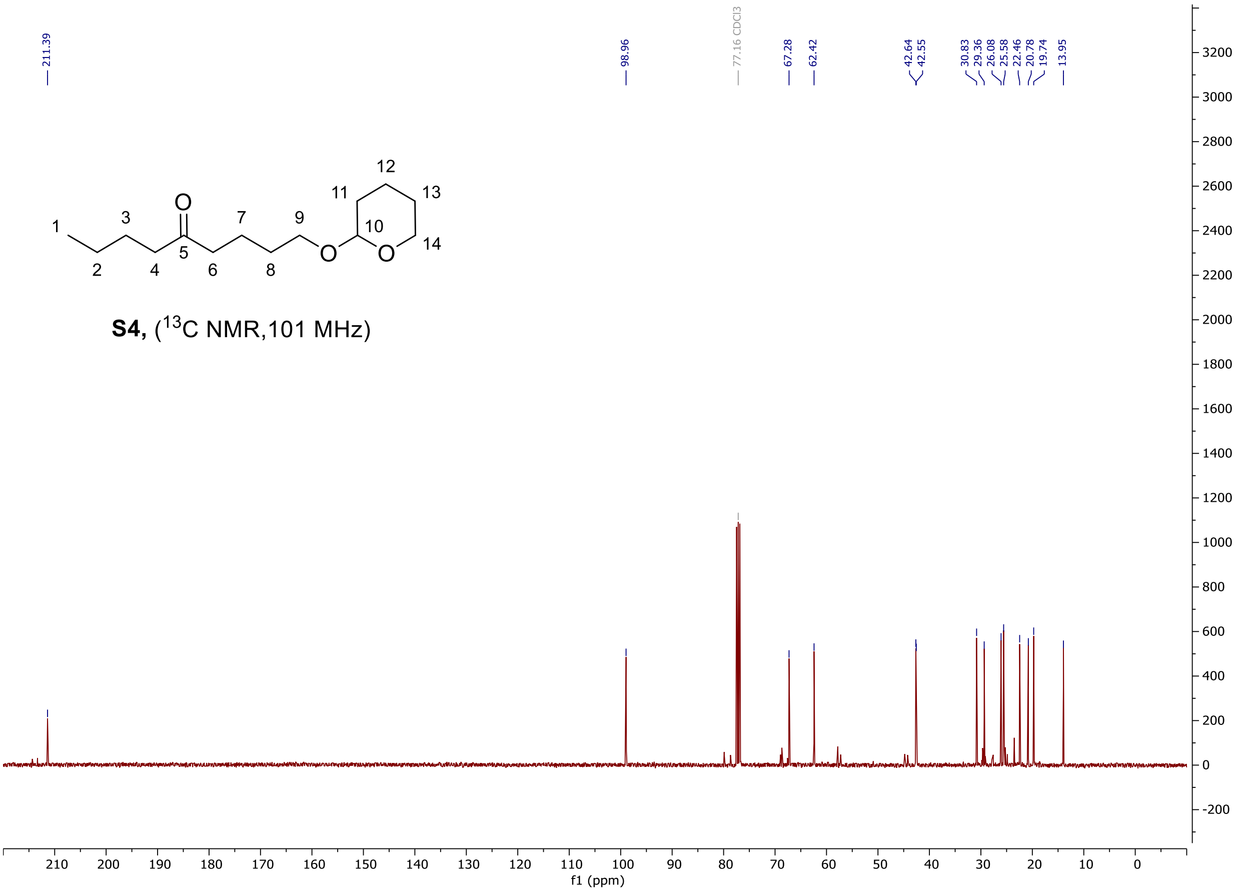
211.39
98.96
77.16 CDCl3
67.28
62.42
42.64
42.55
30.83
29.36
26.08
25.58
22.46
20.78
19.74
13.95
S4, (^{13}C NMR,101 MHz)
f1 (ppm)

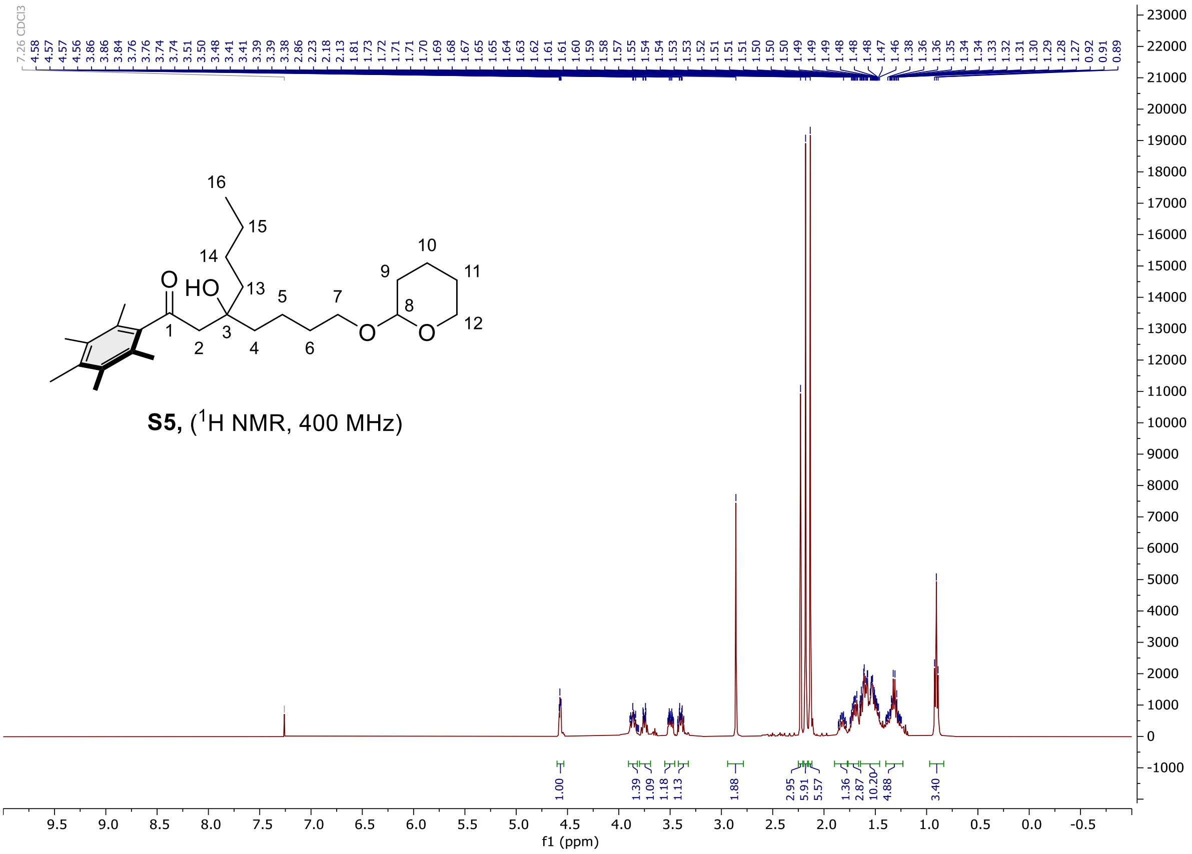
7.26 CDCl3
4.58 4.57 4.57 4.56 3.86 3.86 3.84 3.76 3.76 3.74 3.74 3.51 3.50 3.48 3.41 3.41 3.39 3.39 3.38 2.86 2.23 2.18 2.13 1.81 1.73 1.72 1.71 1.71 1.70 1.69 1.68 1.67 1.65 1.65 1.64 1.63 1.62 1.61 1.61 1.60 1.59 1.58 1.57 1.55 1.54 1.54 1.54 1.53 1.53 1.52 1.51 1.51 1.51 1.50 1.50 1.50 1.49 1.49 1.49 1.48 1.48 1.48 1.47 1.46 1.38 1.36 1.36 1.35 1.34 1.34 1.33 1.32 1.31 1.30 1.29 1.28 1.27 0.92 0.91 0.89
S5, (¹H NMR, 400 MHz)
1.00 1.39 1.09 1.18 1.13 1.88 2.95 5.91 5.57 1.36 2.87 10.20 4.88 3.40
f1 (ppm)

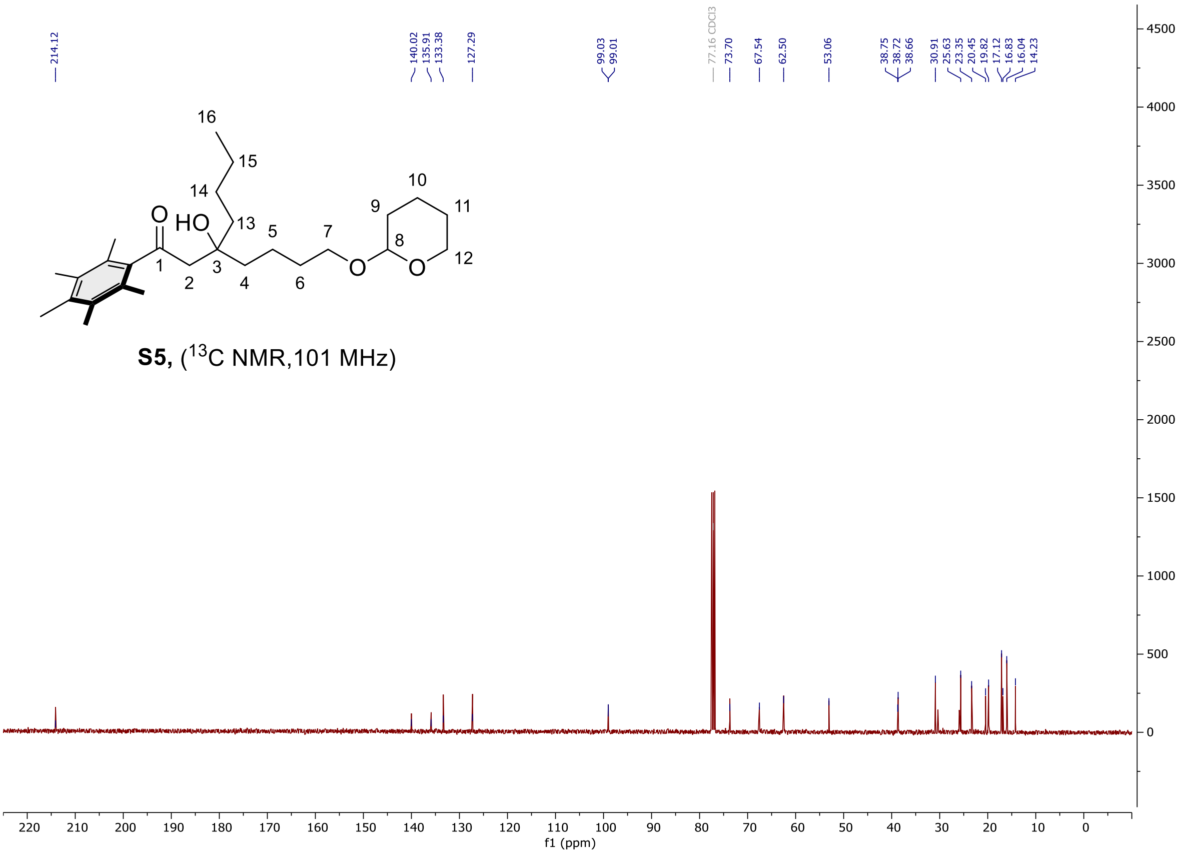
214.12 140.02 135.91 133.38 127.29 99.03 99.01 77.16 CDCl3 73.70 67.54 62.50 53.06 38.75 38.72 38.66 30.91 25.63 23.35 20.45 19.82 17.12 16.83 16.04 14.23
S5, (¹³C NMR,101 MHz)
f1 (ppm)

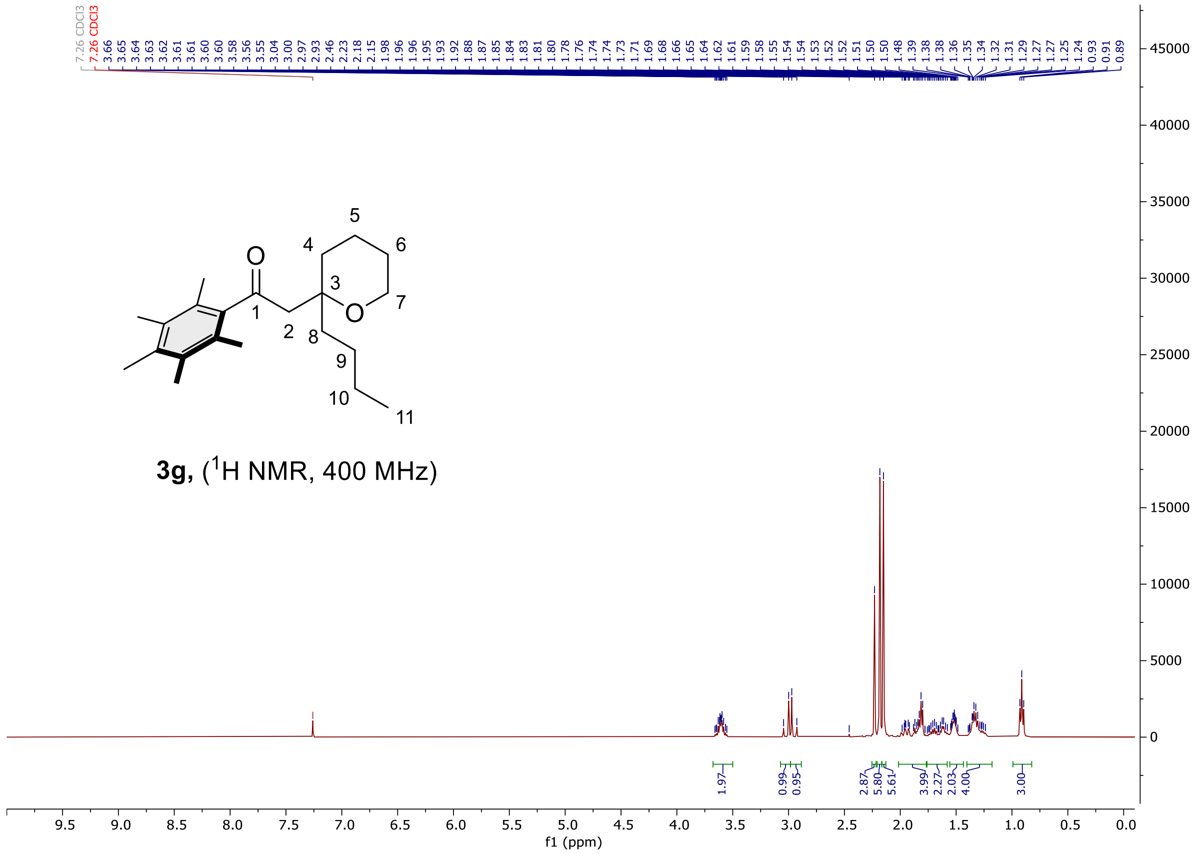

3g, (^{1}H NMR, 400 MHz)

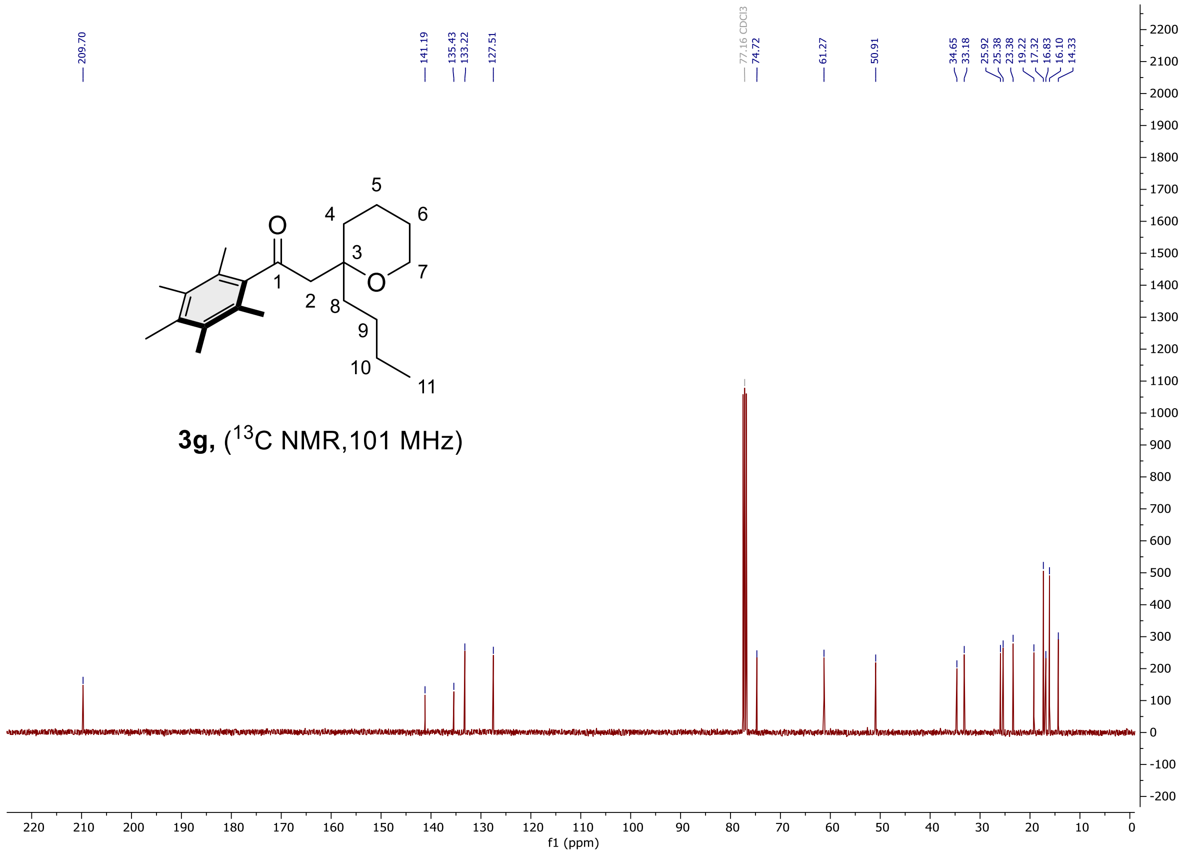

3g, (^{13}C NMR,101 MHz)

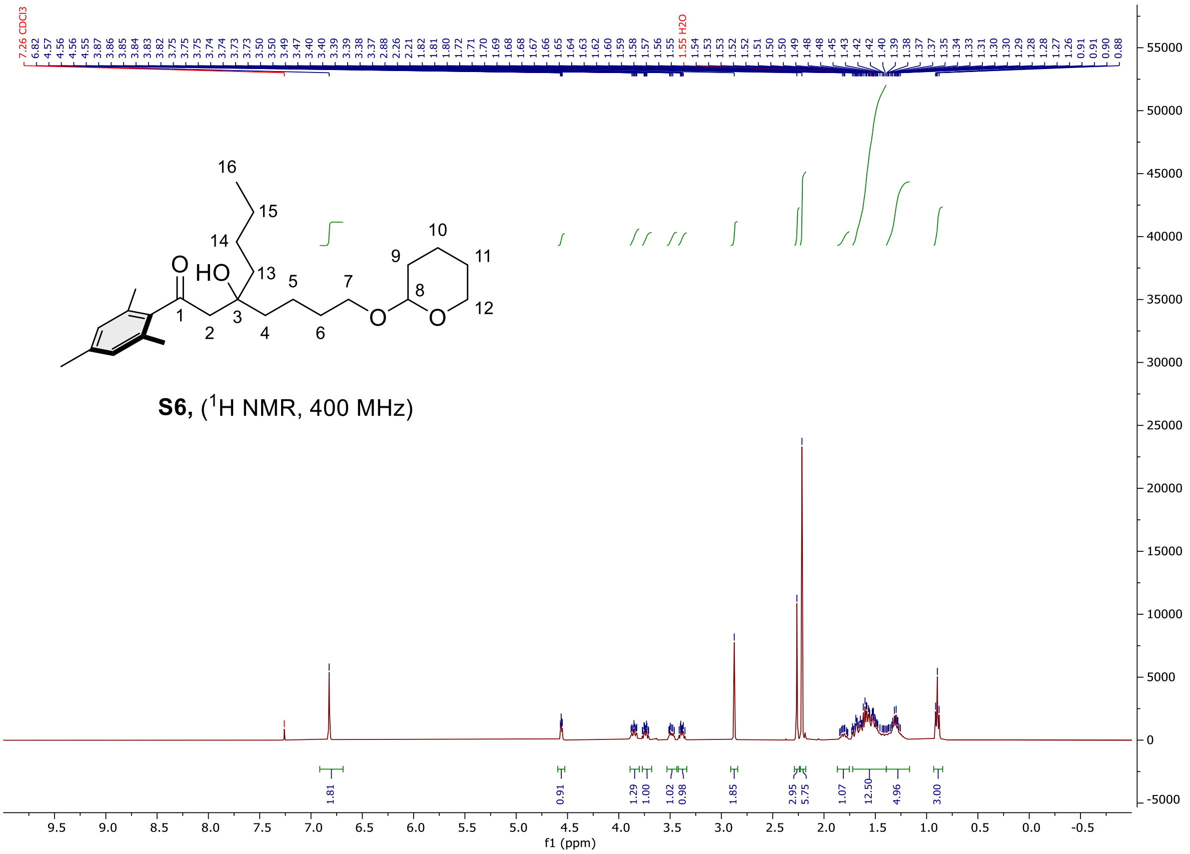

S6, (^{1}H NMR, 400 MHz)

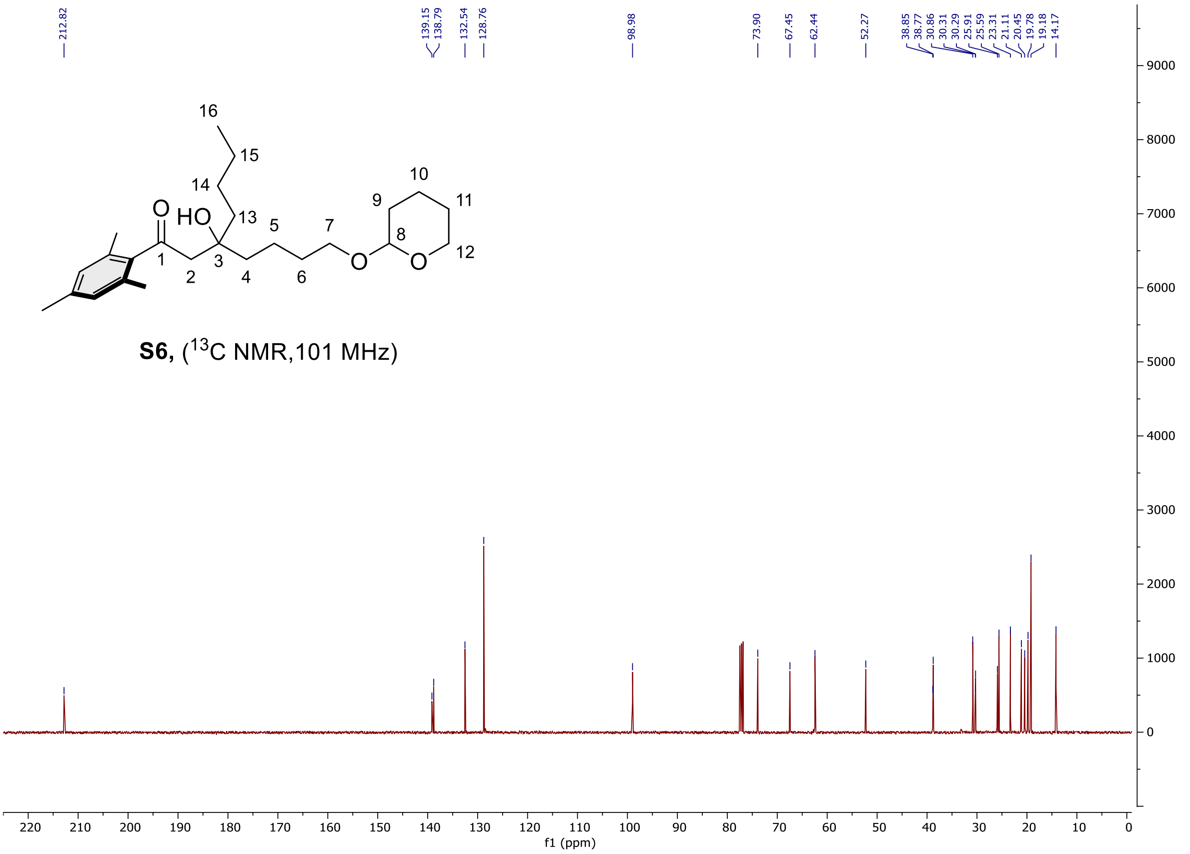

S6, (^{13}C NMR,101 MHz)

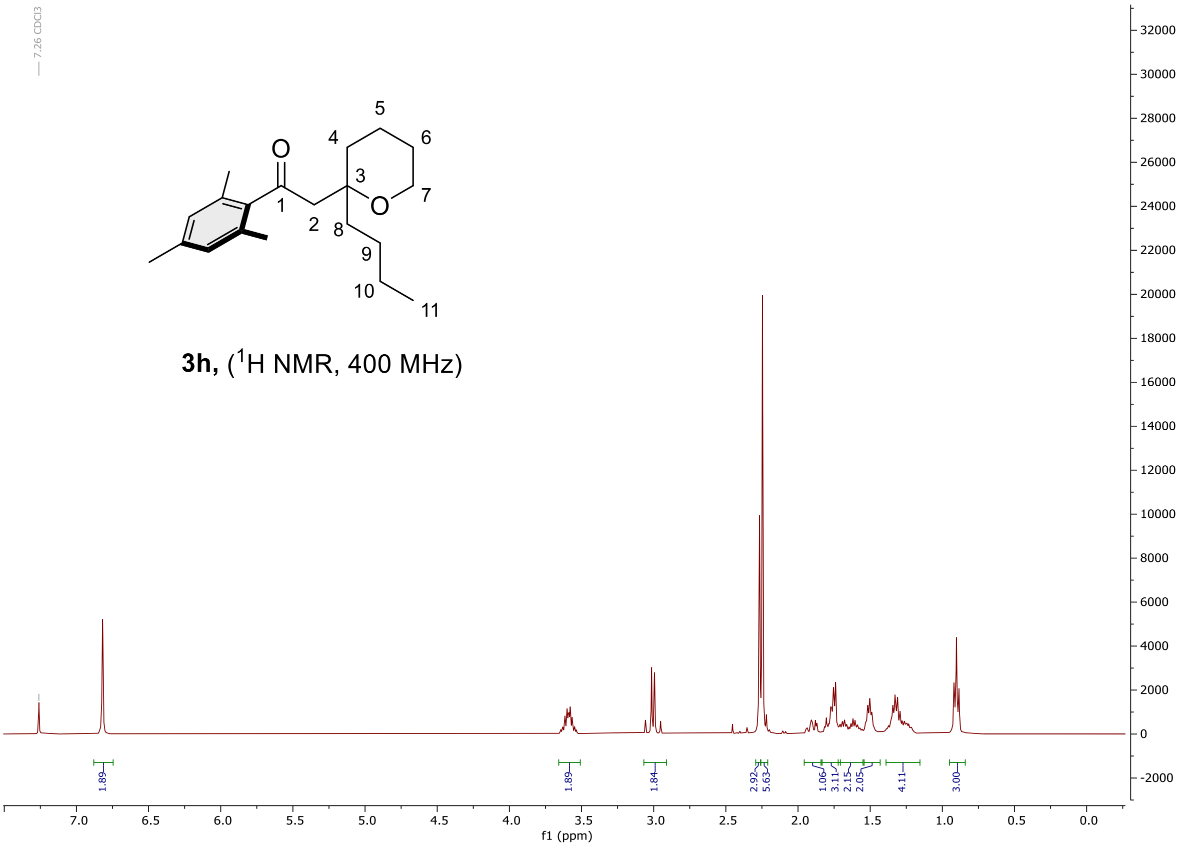
7.26 CDCl3
3h, (1H NMR, 400 MHz)
1.89
1.89
1.84
2.92
5.63
1.06
3.11
2.15
2.05
4.11
3.00
f1 (ppm)

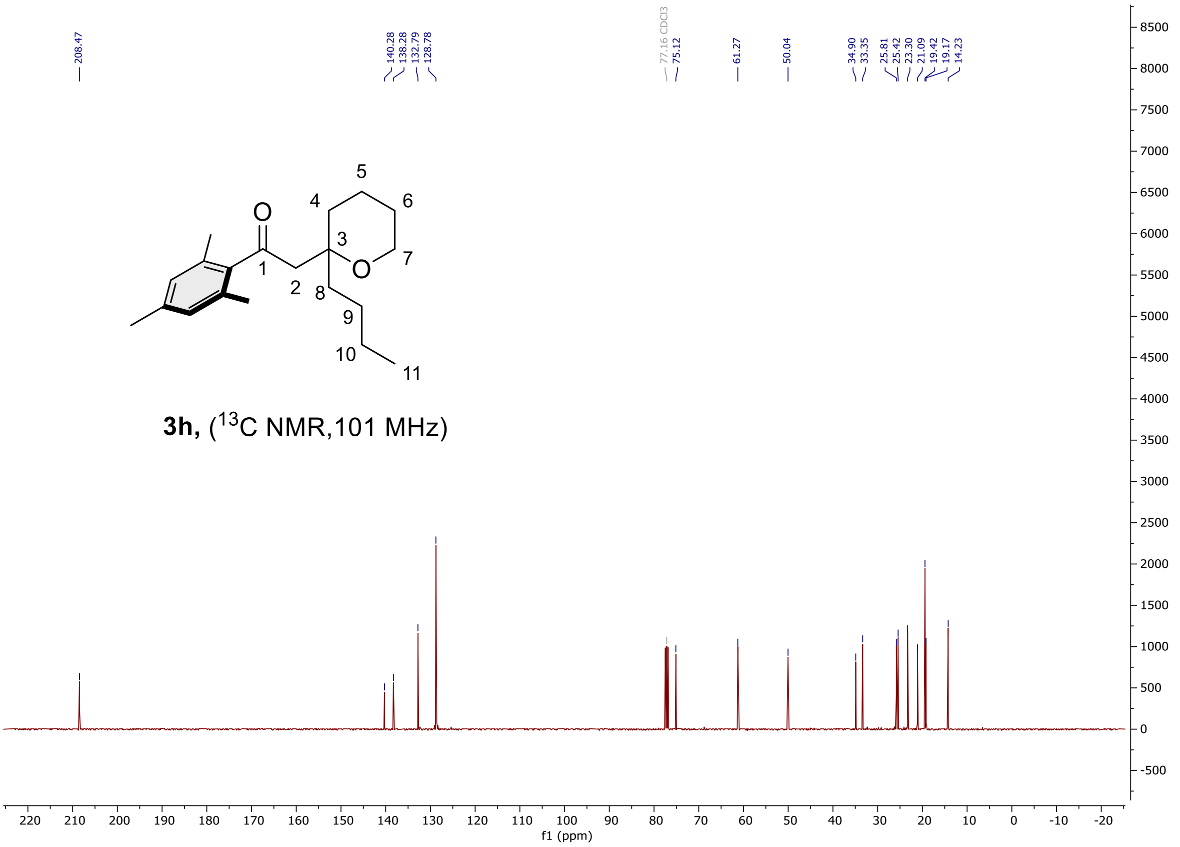
208.47
140.28
138.28
132.79
128.78
77.16 CDCl3
75.12
61.27
50.04
34.90
33.35
25.81
25.42
23.30
21.09
19.42
19.17
14.23
3h, (13C NMR,101 MHz)
f1 (ppm)

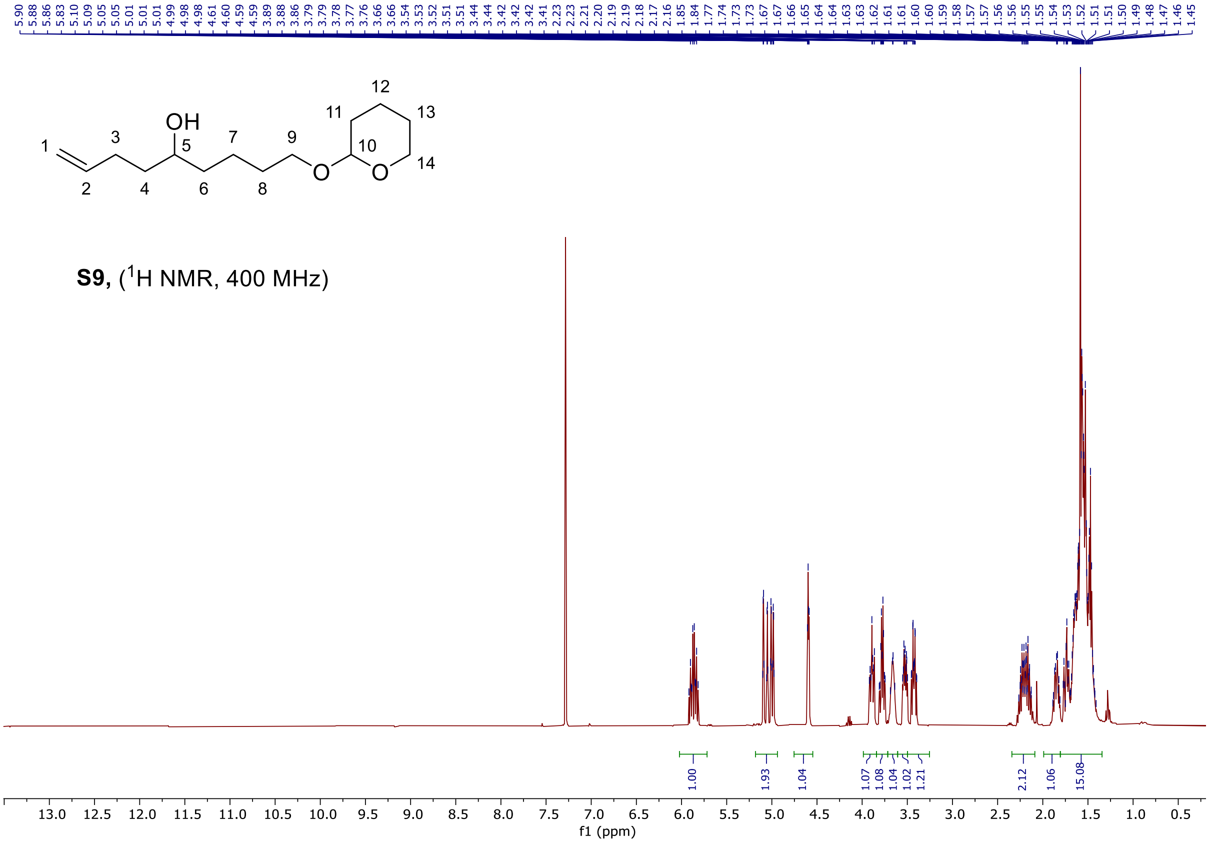

S9, (1H NMR, 400 MHz)
f1 (ppm)

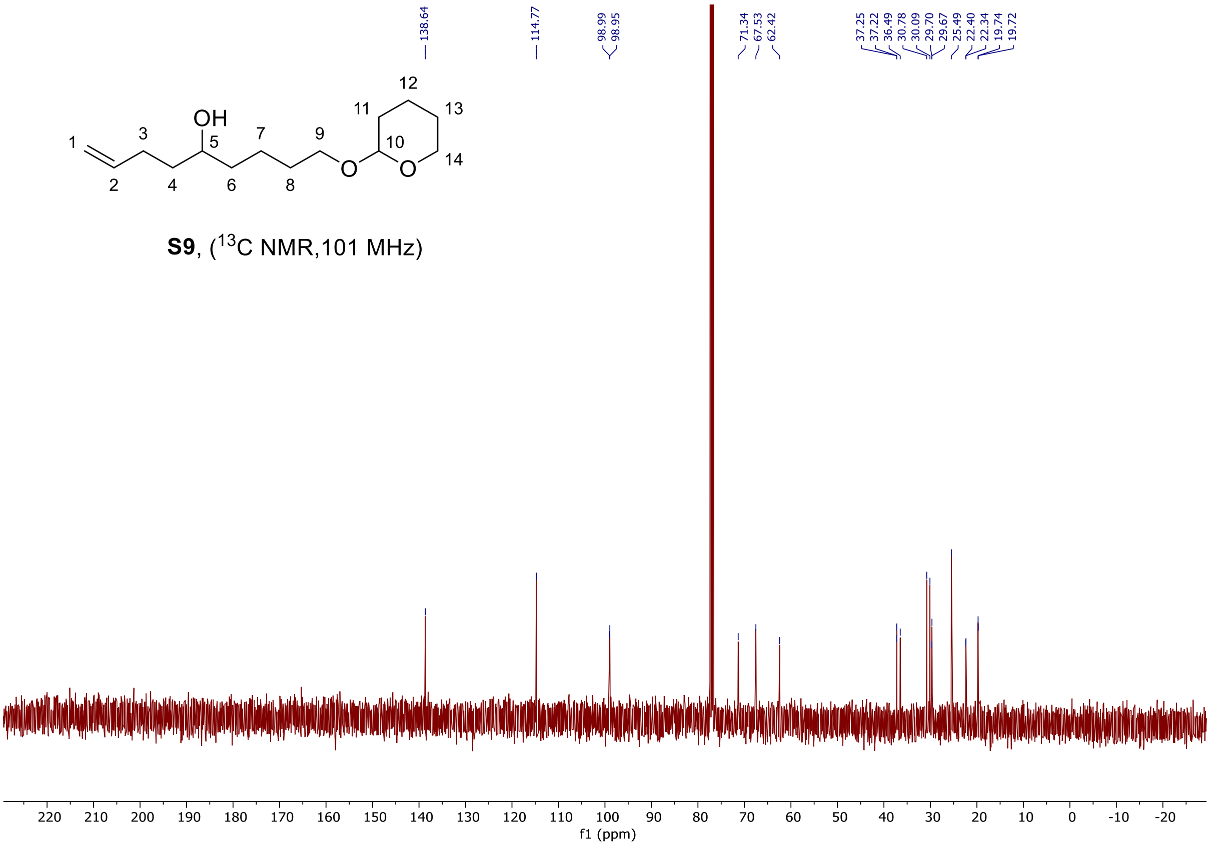

S9, (13C NMR,101 MHz)
138.64
114.77
98.99
98.95
71.34
67.53
62.42
37.25
37.22
36.49
30.78
30.09
29.70
29.67
25.49
22.40
22.34
19.74
19.72
f1 (ppm)

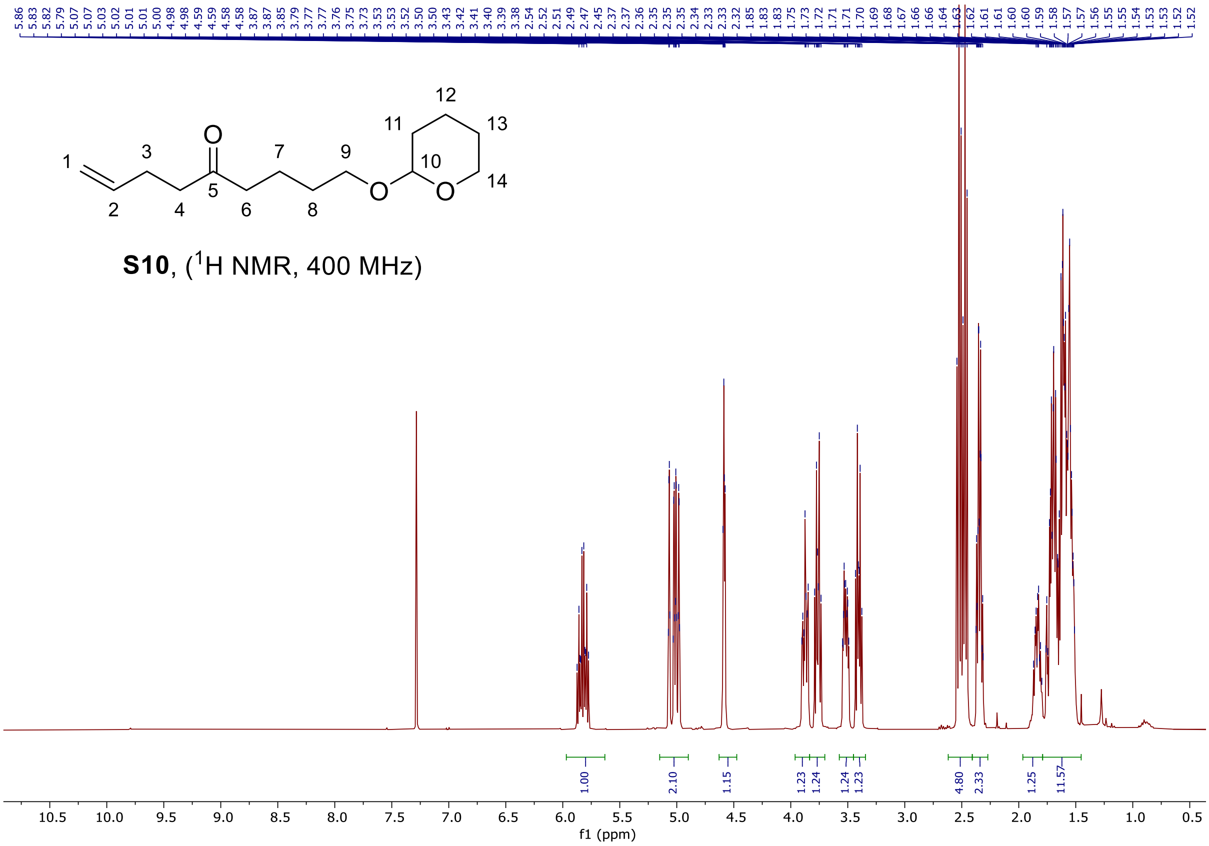
S10, (^{1}H NMR, 400 MHz)
f1 (ppm)

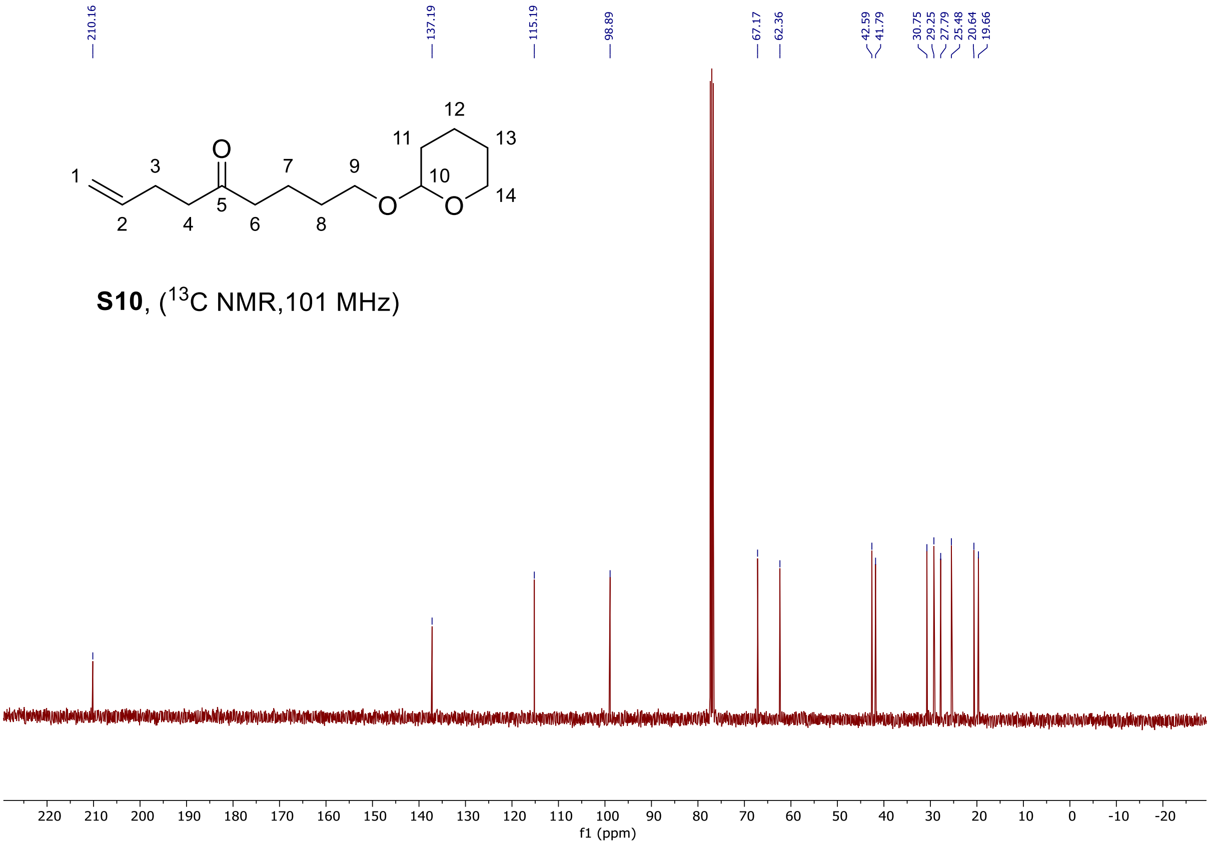
S10, (^{13}C NMR,101 MHz)
f1 (ppm)

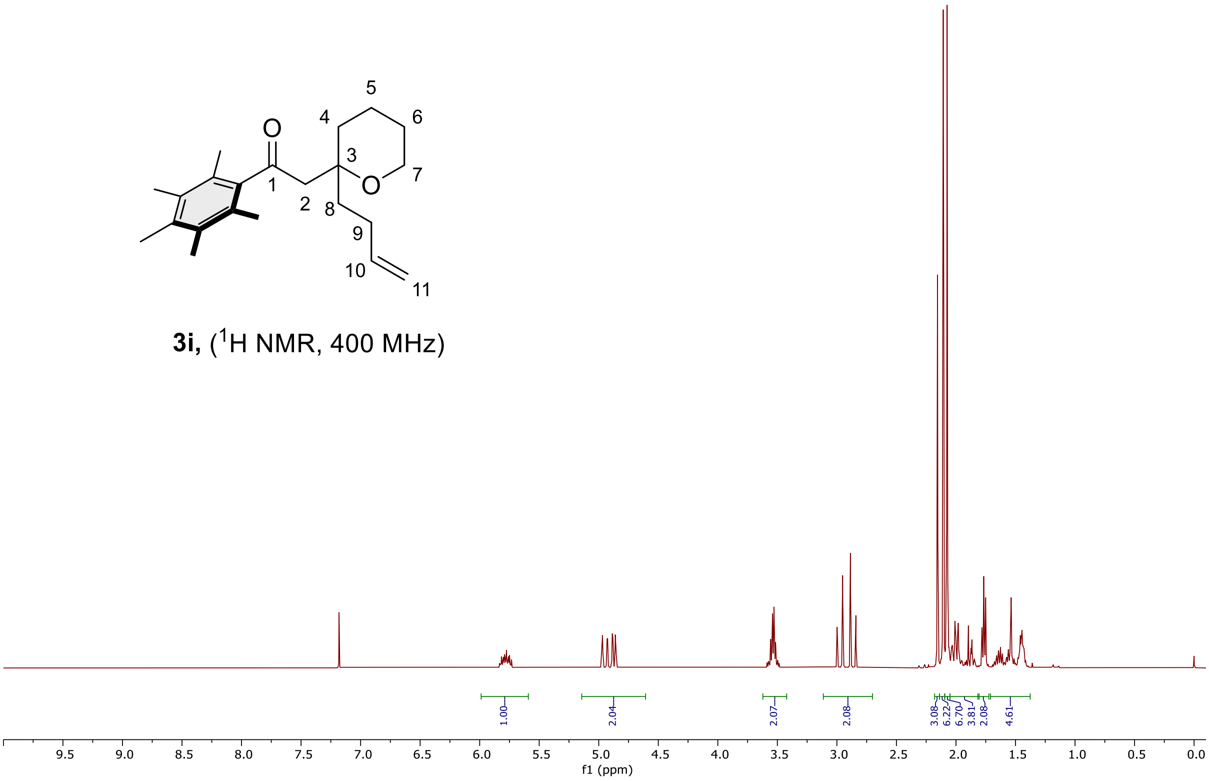

O
1
2
3
4
5
6
7
8
9
10
11
O
3i, (^{1}H NMR, 400 MHz)
1.00
2.04
2.07
2.08
3.08
6.22
6.70
3.81
2.08
4.61
9.5 9.0 8.5 8.0 7.5 7.0 6.5 6.0 5.5 5.0 4.5 4.0 3.5 3.0 2.5 2.0 1.5 1.0 0.5 0.0
f1 (ppm)

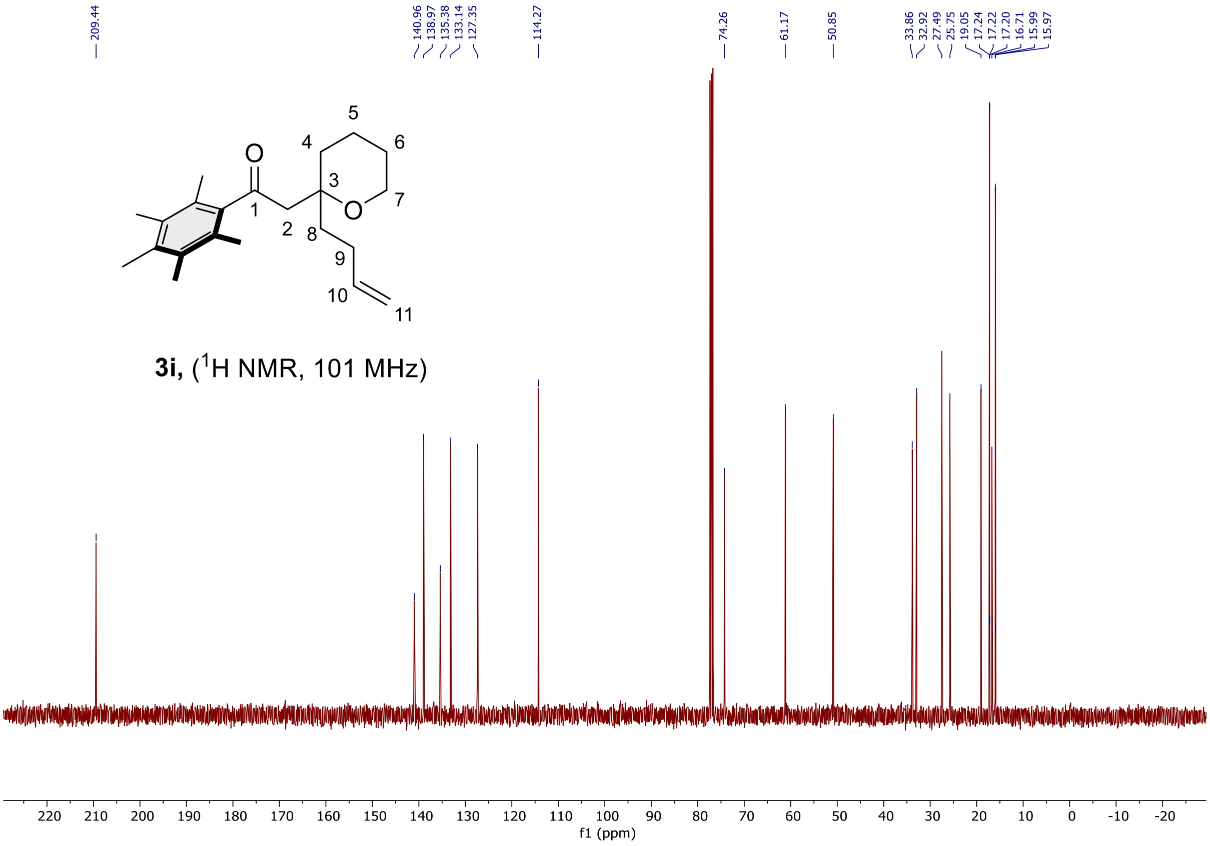

209.44
140.96
138.97
135.38
133.14
127.35
114.27
74.26
61.17
50.85
33.86
32.92
27.49
25.75
19.05
17.24
17.22
17.20
16.71
15.99
15.97
O
1
2
3
4
5
6
7
8
9
10
11
O
3i, (^{1}H NMR, 101 MHz)
220 210 200 190 180 170 160 150 140 130 120 110 100 90 80 70 60 50 40 30 20 10 0 -10 -20
f1 (ppm)

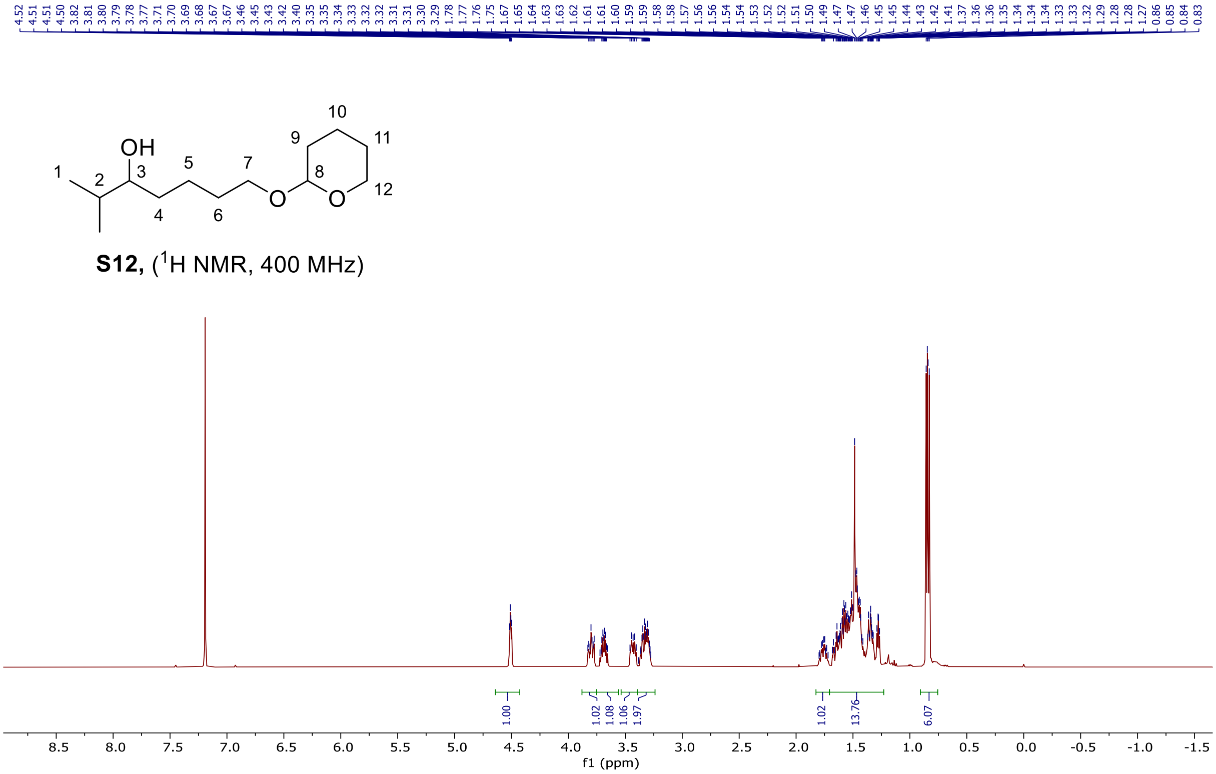

S12, (1H NMR, 400 MHz)
f1 (ppm)

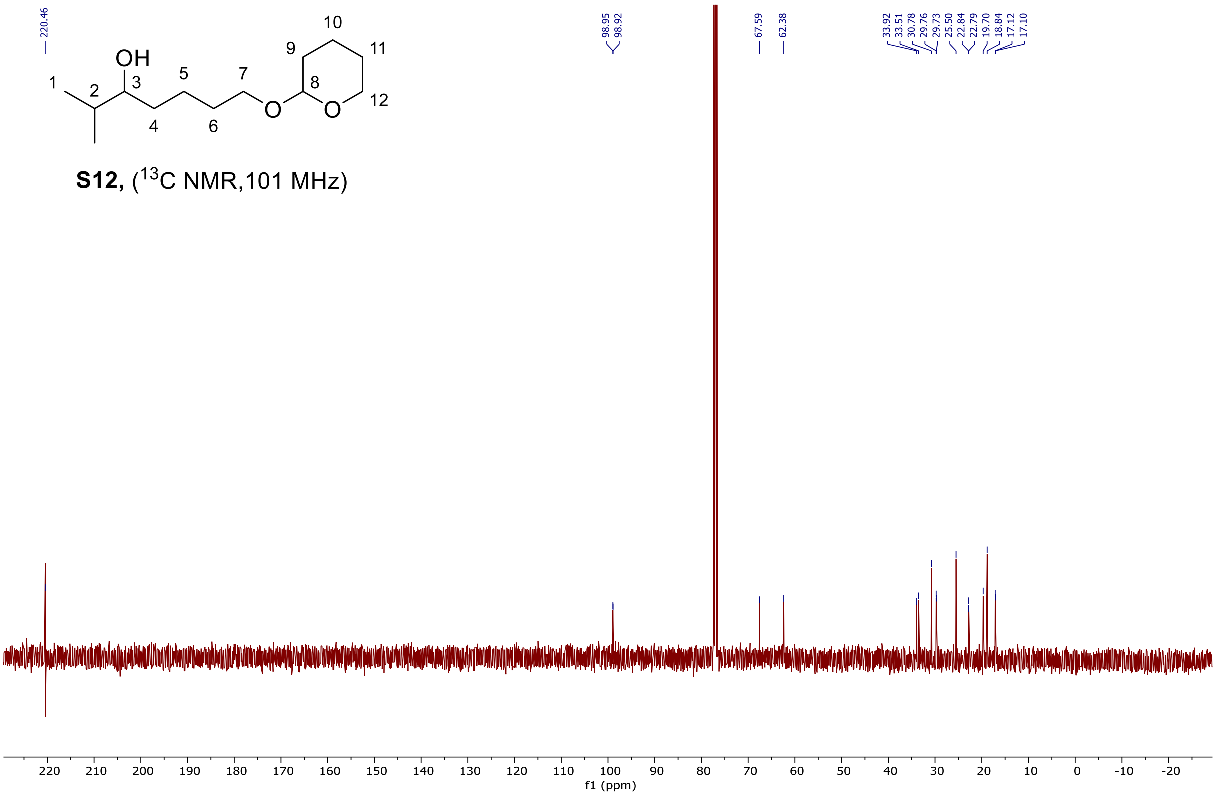

220.46
98.95
98.92
67.59
62.38
33.92
33.51
30.78
29.76
29.73
25.50
22.84
22.79
19.70
18.84
17.12
17.10
S12, (13C NMR,101 MHz)
f1 (ppm)

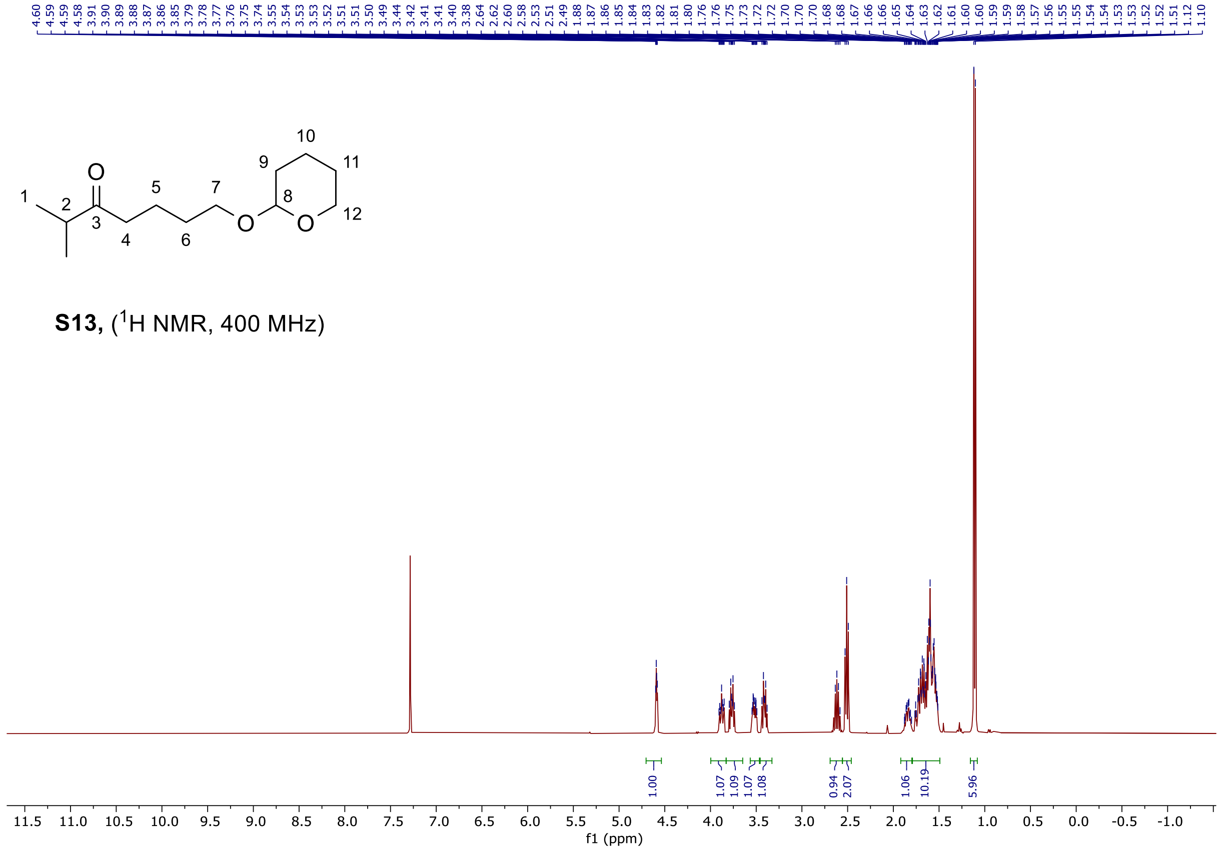
S13, (1H NMR, 400 MHz)
f1 (ppm)

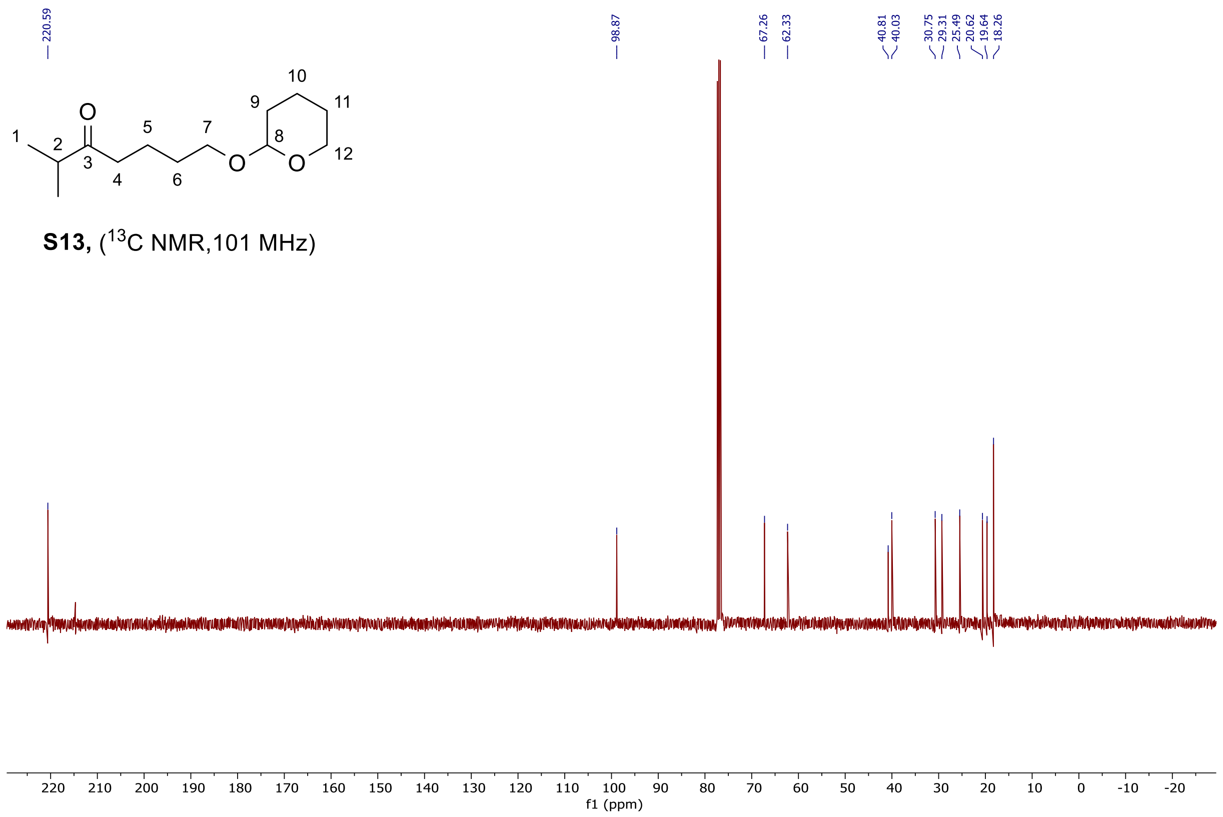
220.59
98.87
67.26
62.33
40.81
40.03
30.75
29.31
25.49
20.62
19.64
18.26
S13, (13C NMR,101 MHz)
f1 (ppm)

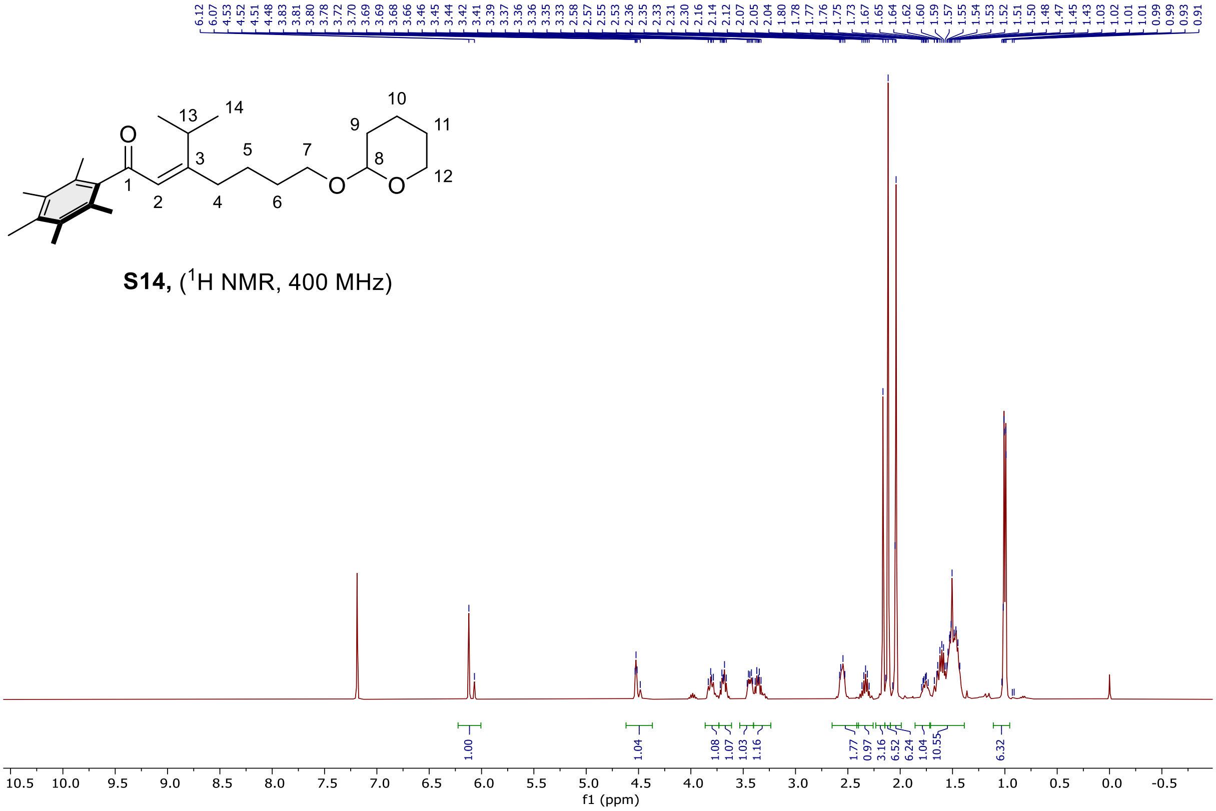

S14, (^{1}H NMR, 400 MHz)

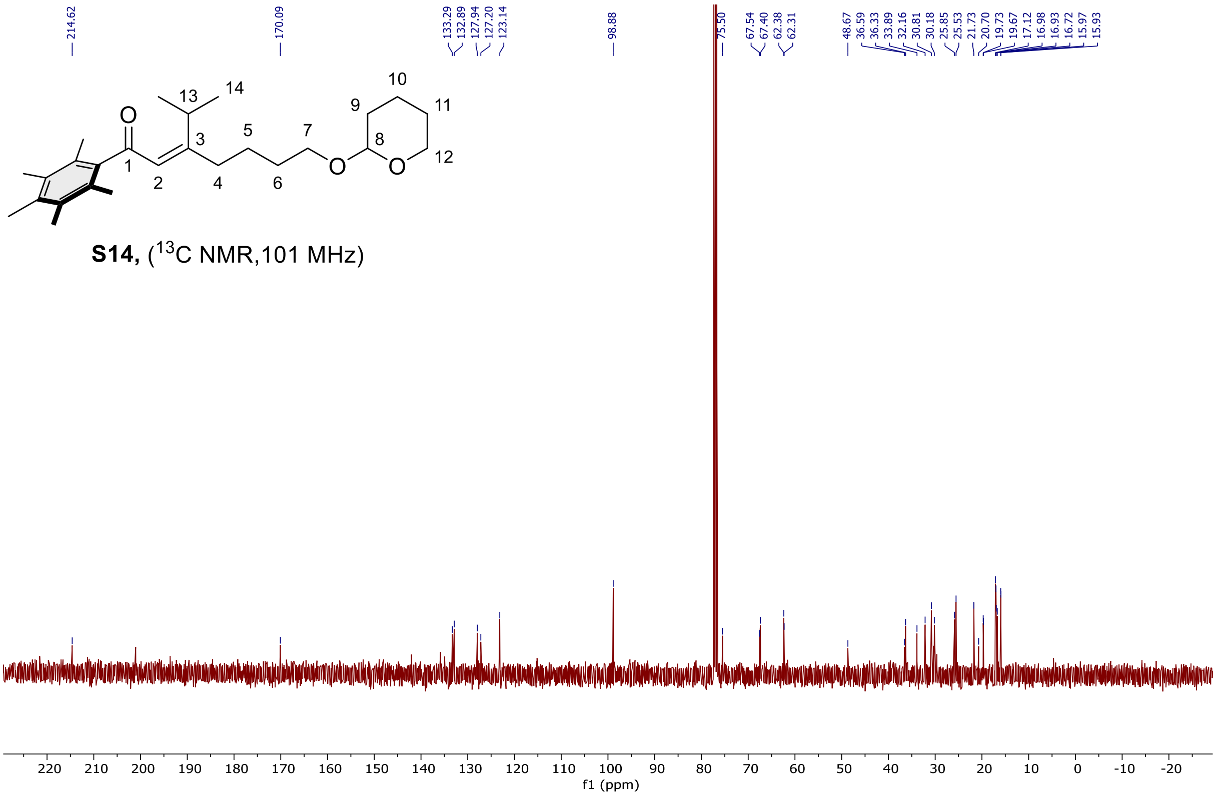

S14, (^{13}C NMR,101 MHz)

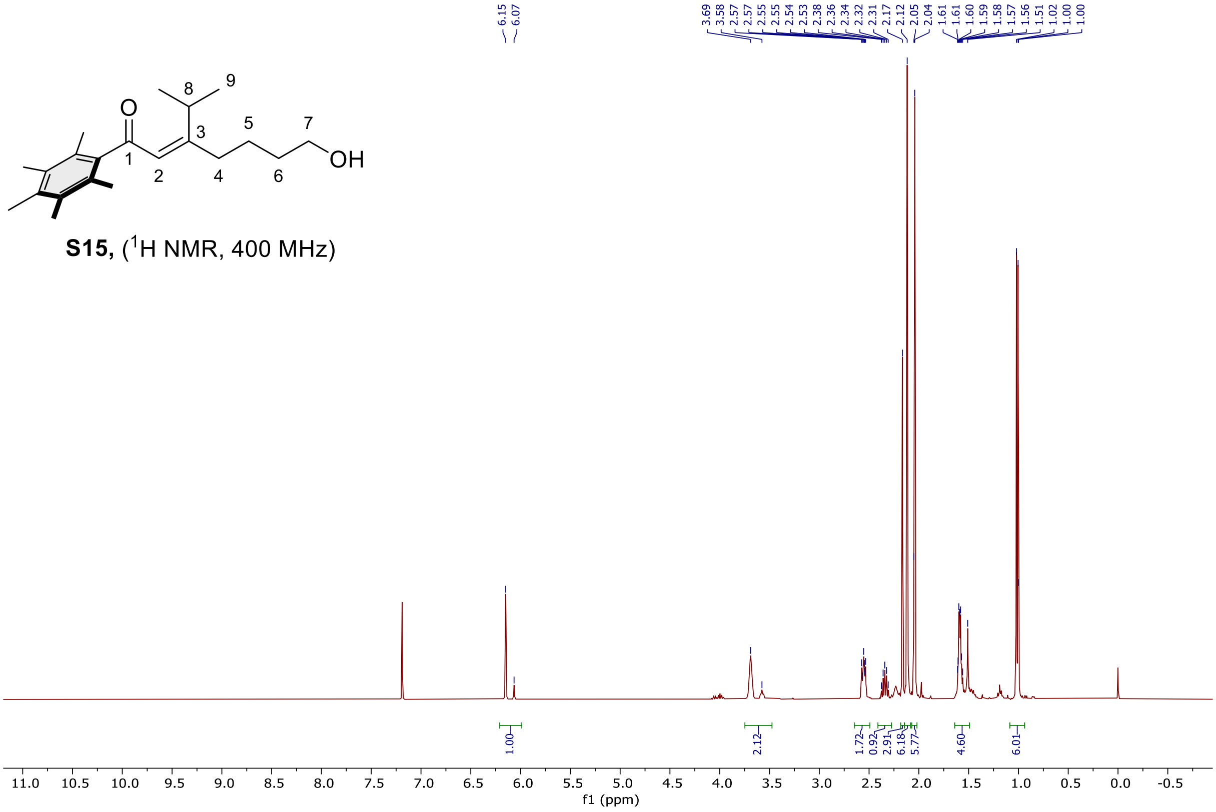

S15, (^{1}H NMR, 400 MHz)

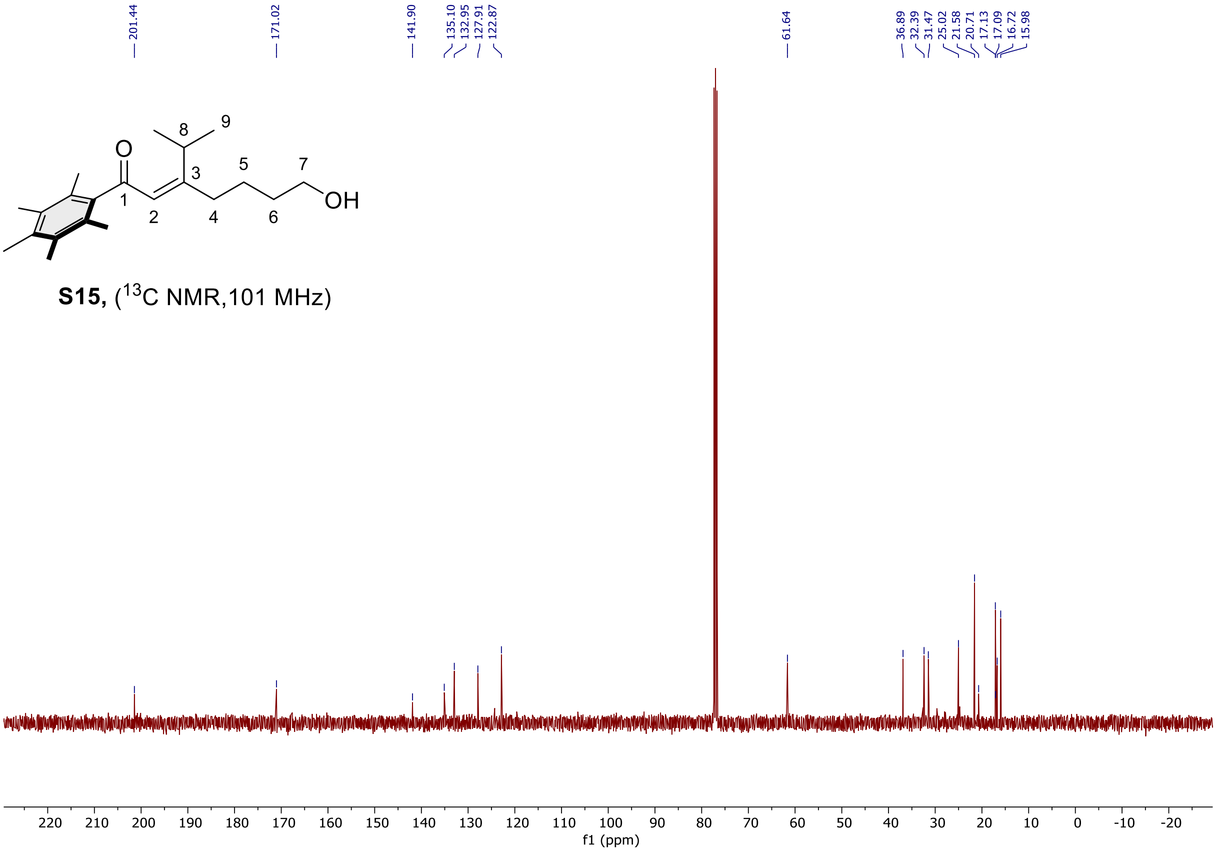

S15, (^{13}C NMR,101 MHz)

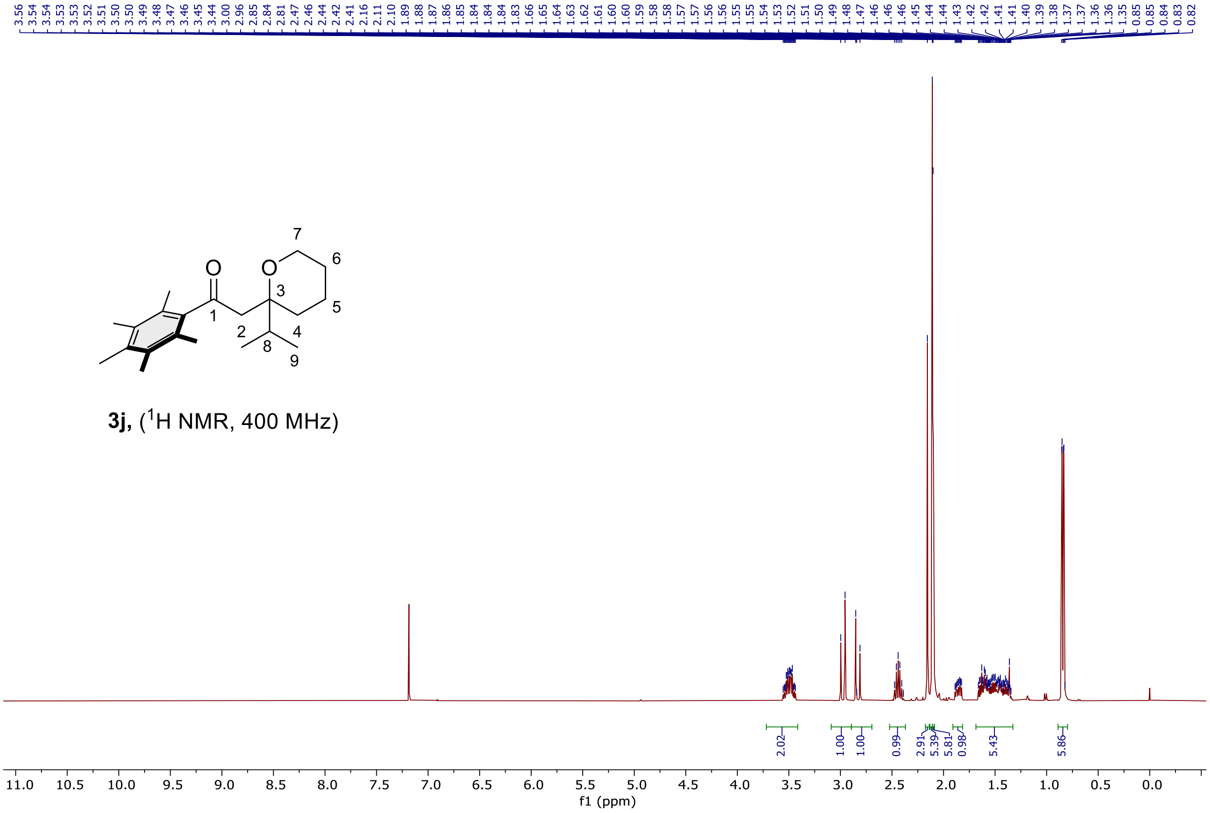
3.56 3.54 3.54 3.53 3.53 3.52 3.51 3.50 3.50 3.49 3.48 3.47 3.46 3.45 3.44 3.00 2.96 2.85 2.84 2.81 2.47 2.46 2.44 2.42 2.41 2.16 2.11 2.10 1.89 1.88 1.87 1.86 1.85 1.84 1.84 1.84 1.83 1.66 1.65 1.64 1.63 1.62 1.61 1.60 1.60 1.59 1.58 1.58 1.57 1.57 1.56 1.56 1.55 1.55 1.54 1.53 1.52 1.51 1.50 1.49 1.48 1.47 1.46 1.46 1.45 1.44 1.44 1.43 1.42 1.42 1.41 1.41 1.40 1.39 1.38 1.37 1.37 1.36 1.36 1.35 0.85 0.85 0.84 0.83 0.82
O
O
1
2
3
4
5
6
7
8
9
3j, (¹H NMR, 400 MHz)
2.02
1.00
1.00
0.99
2.91
5.39
5.81
0.98
5.43
5.86
11.0 10.5 10.0 9.5 9.0 8.5 8.0 7.5 7.0 6.5 6.0 5.5 5.0 4.5 4.0 3.5 3.0 2.5 2.0 1.5 1.0 0.5 0.0
f1 (ppm)

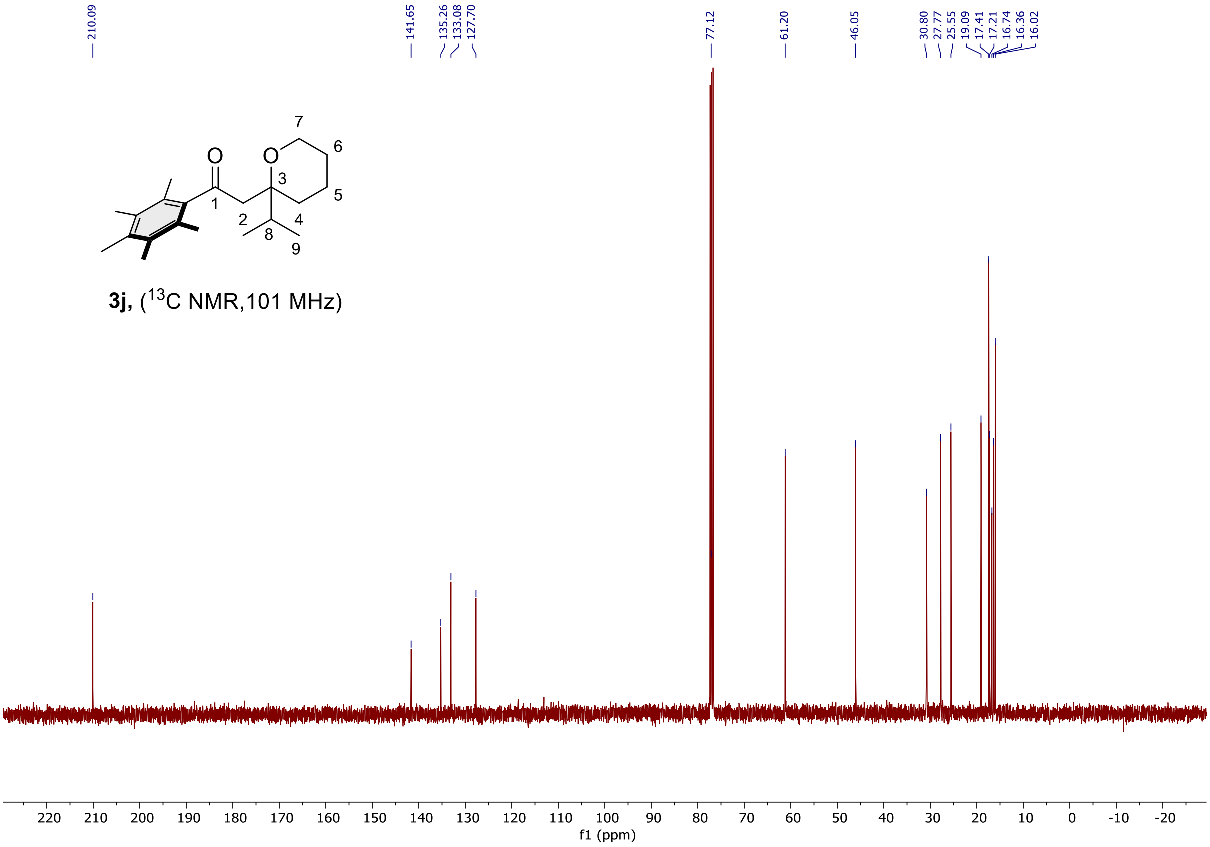
210.09
141.65
135.26
133.08
127.70
77.12
61.20
46.05
30.80
27.77
25.55
19.09
17.41
17.21
16.74
16.36
16.02
O
O
1
2
3
4
5
6
7
8
9
3j, (¹³C NMR,101 MHz)
220 210 200 190 180 170 160 150 140 130 120 110 100 90 80 70 60 50 40 30 20 10 0 -10 -20
f1 (ppm)

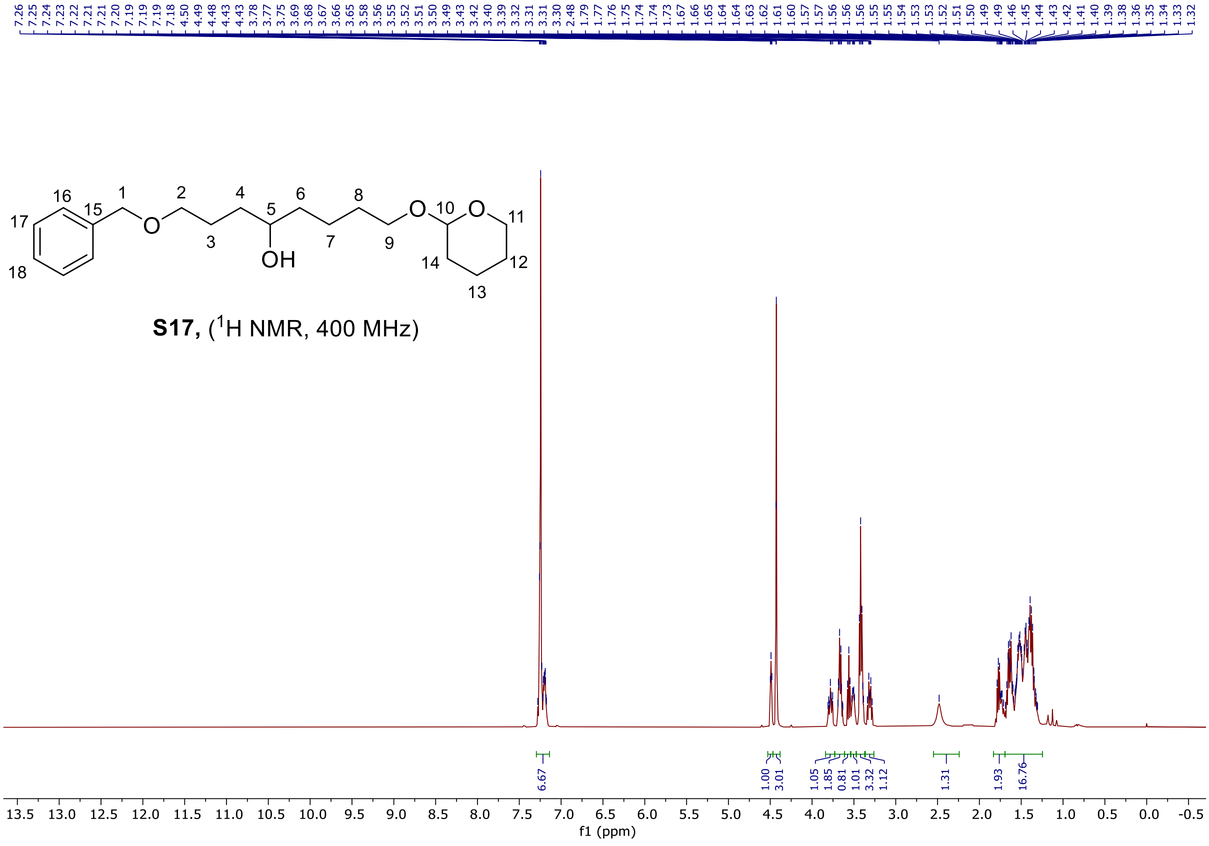
S17, (1H NMR, 400 MHz)
f1 (ppm)

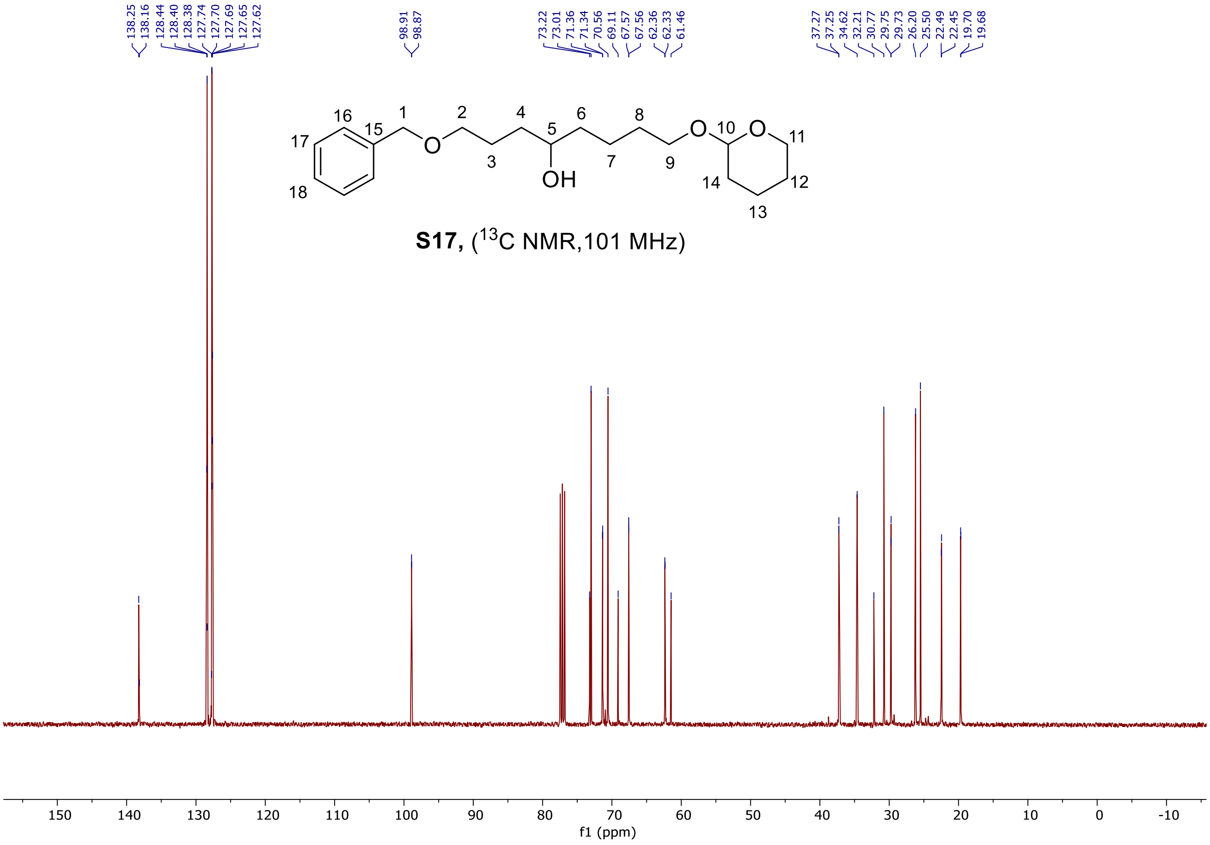
S17, (13C NMR,101 MHz)
f1 (ppm)

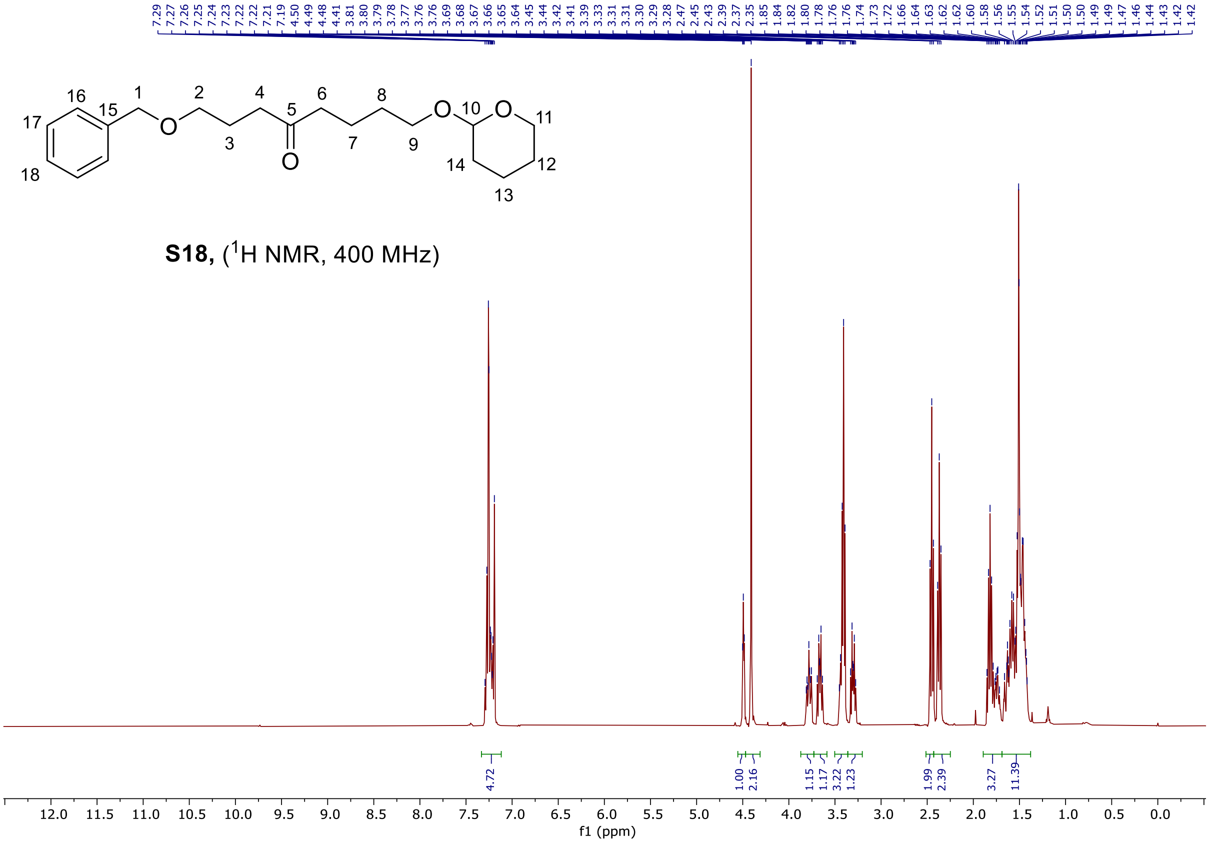
S18, (¹H NMR, 400 MHz)
f1 (ppm)

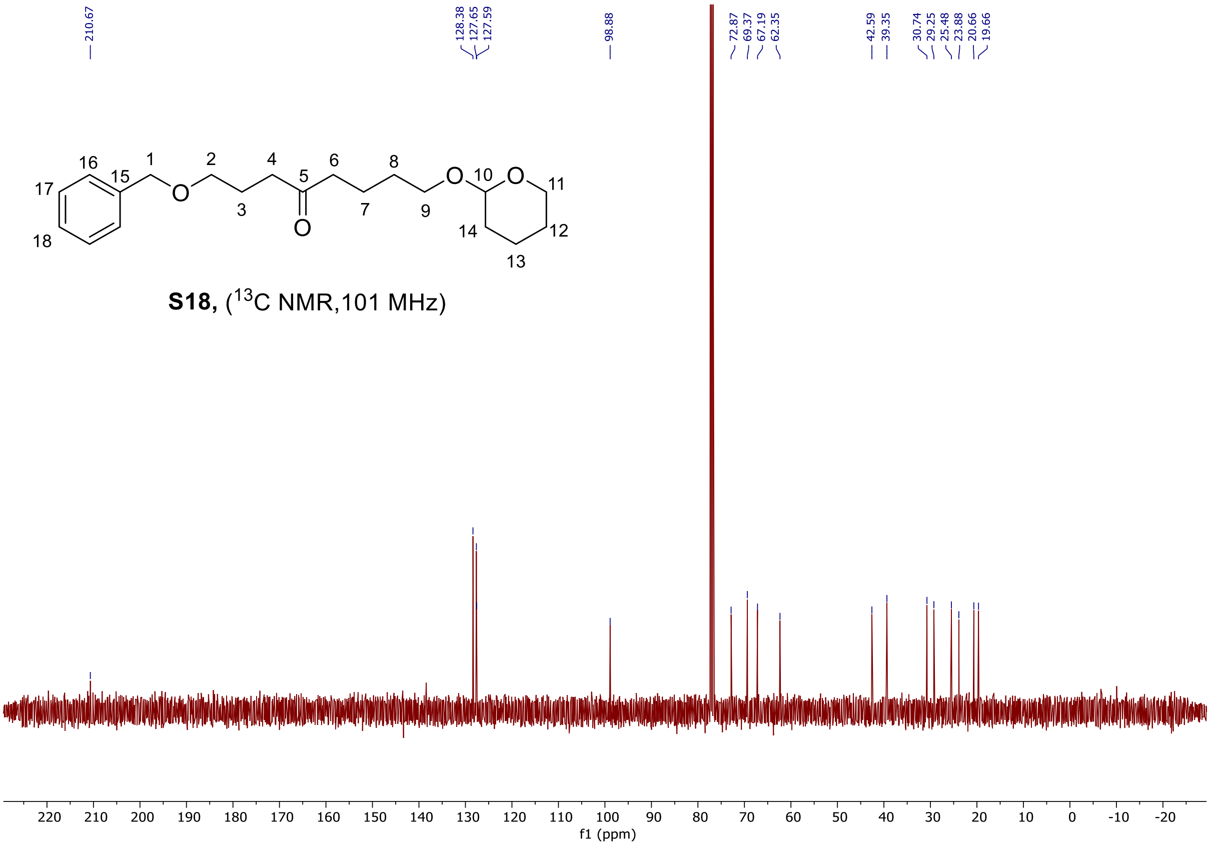
S18, (¹³C NMR,101 MHz)
f1 (ppm)

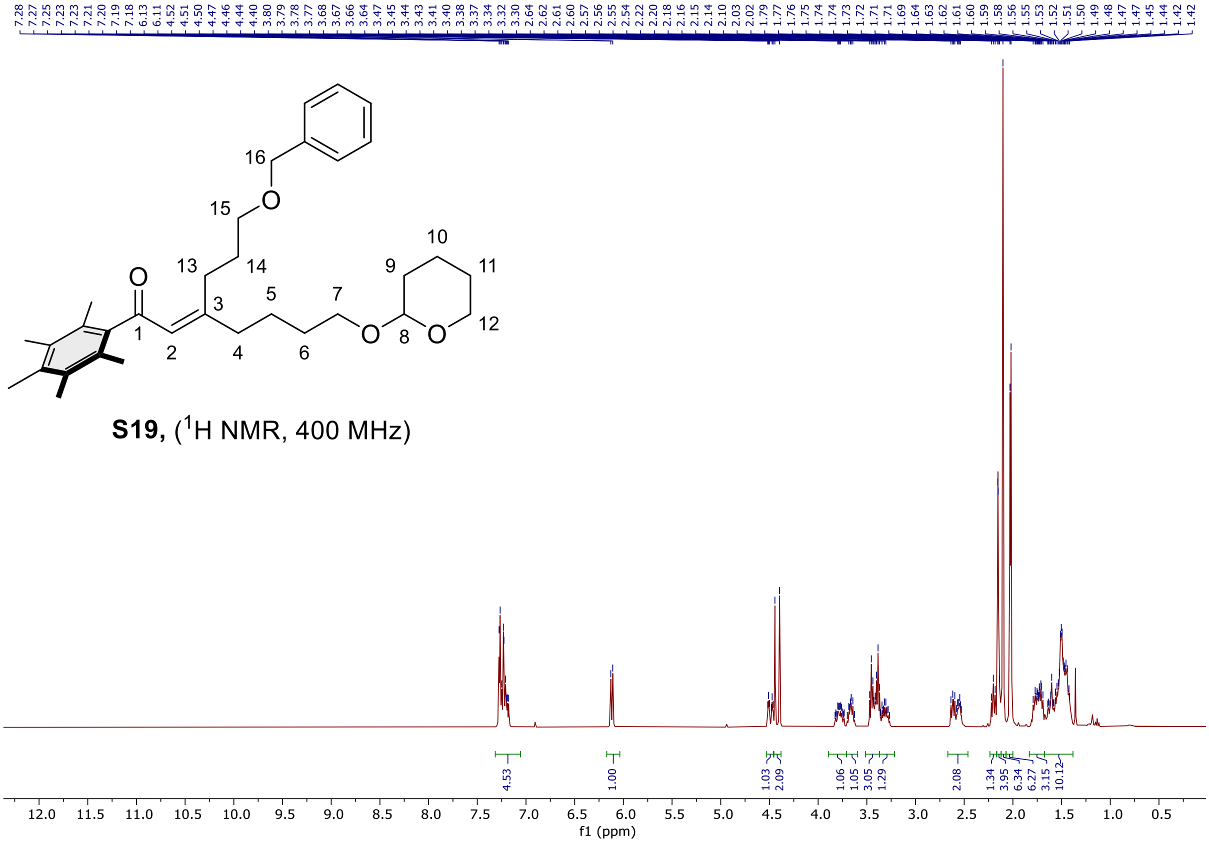
S19, (1H NMR, 400 MHz)
f1 (ppm)

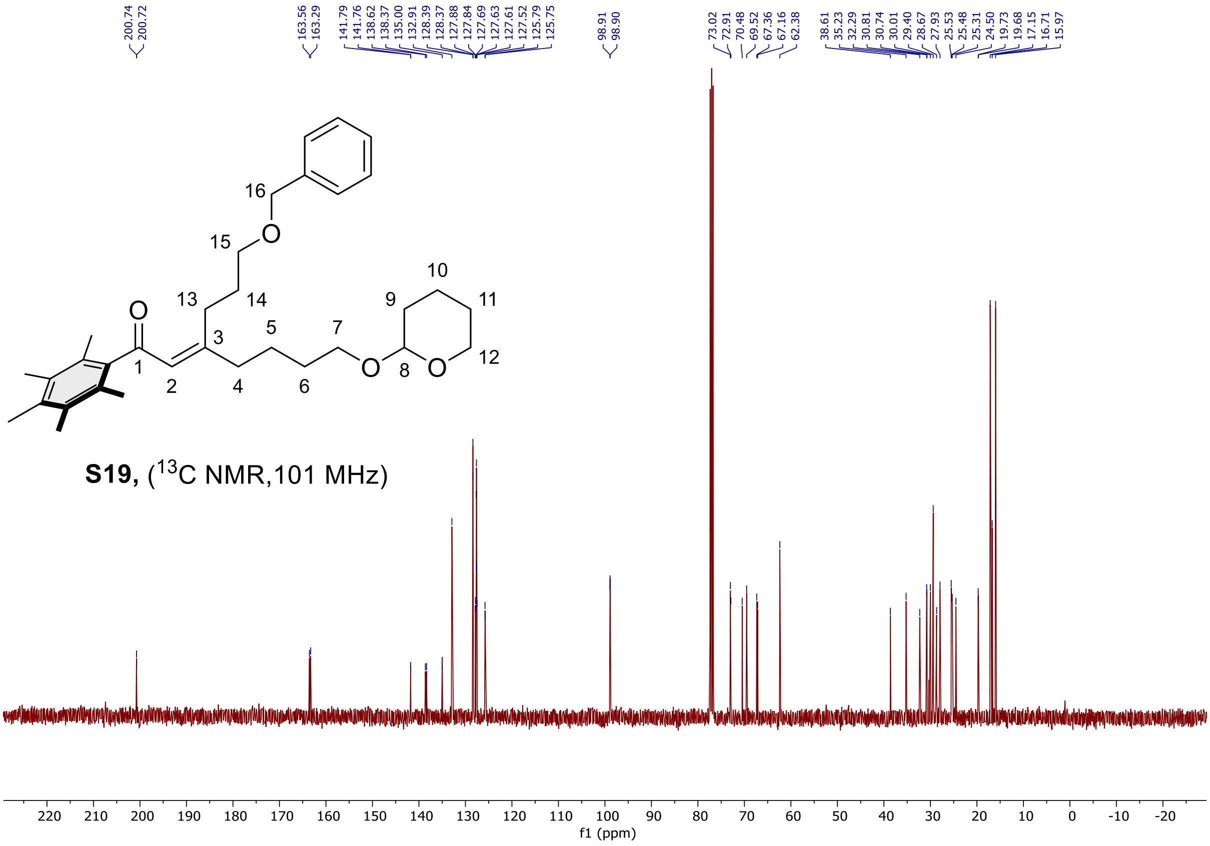
S19, (13C NMR,101 MHz)
f1 (ppm)

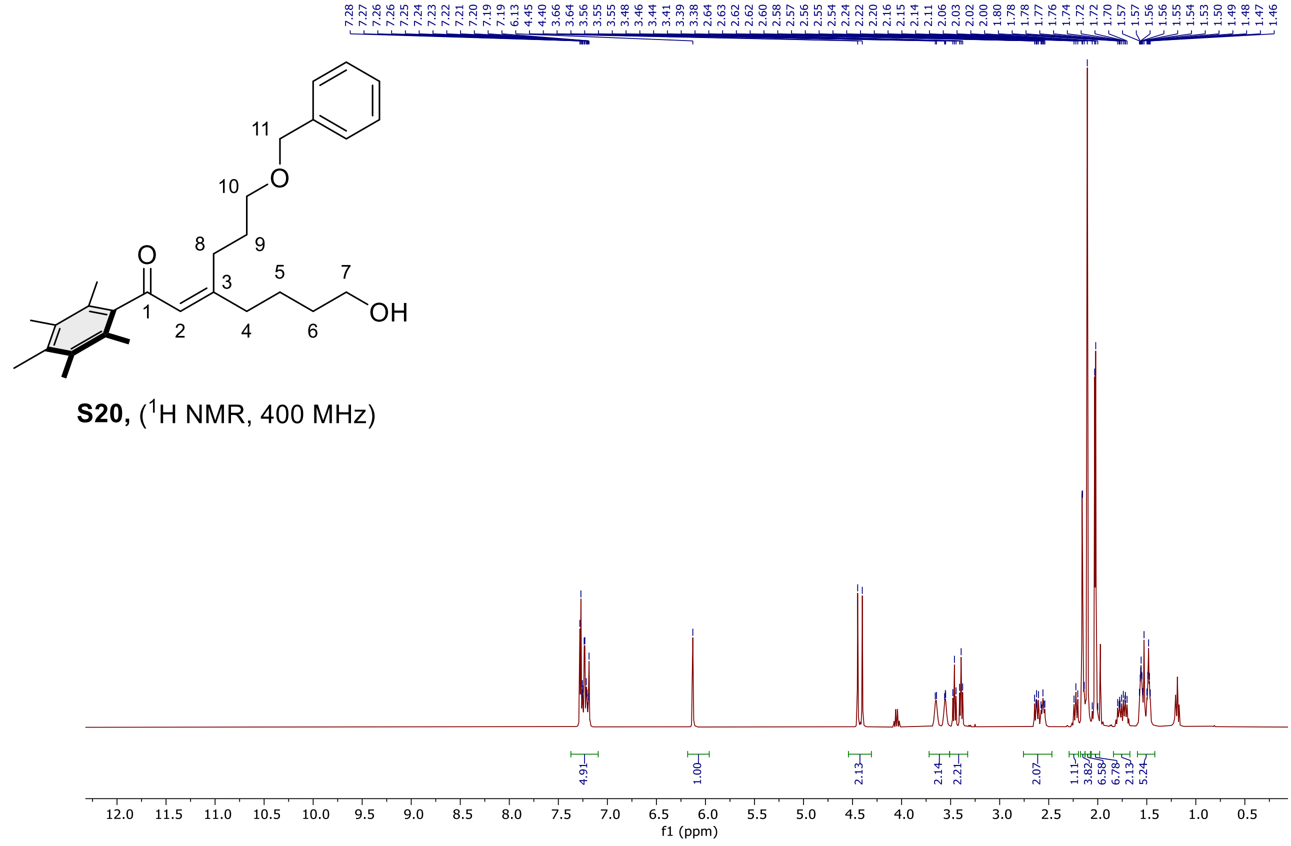
S20, (^{1}H NMR, 400 MHz)
f1 (ppm)

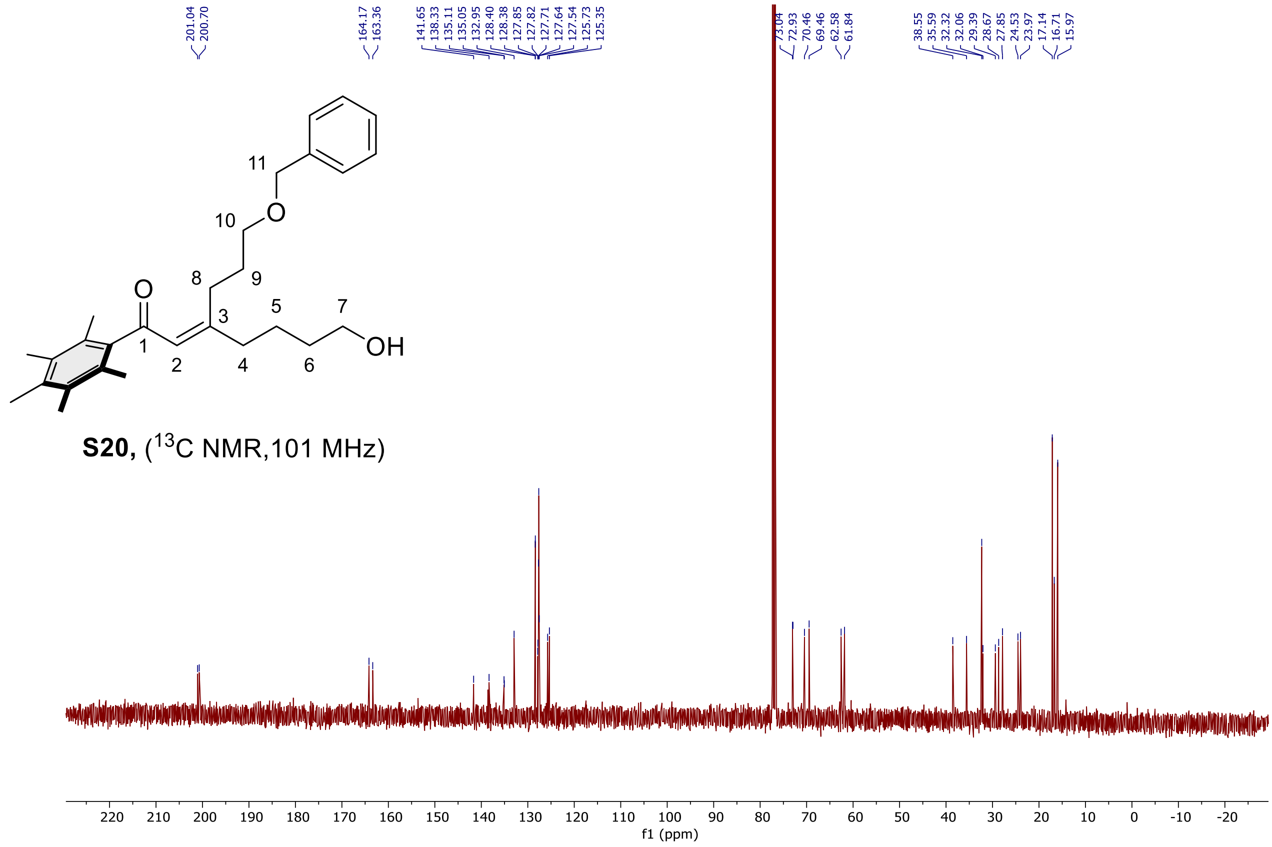
S20, (^{13}C NMR,101 MHz)
f1 (ppm)

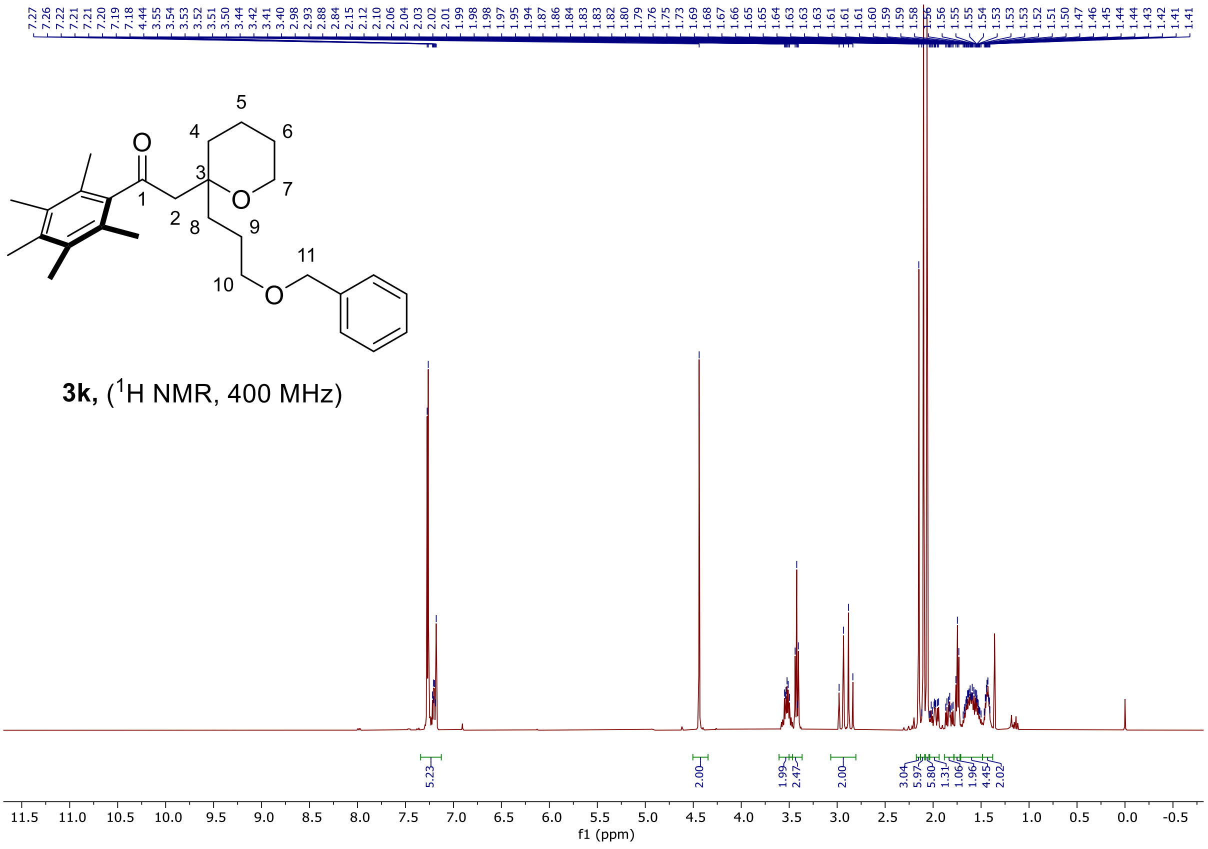

3k, (^{1}H NMR, 400 MHz)

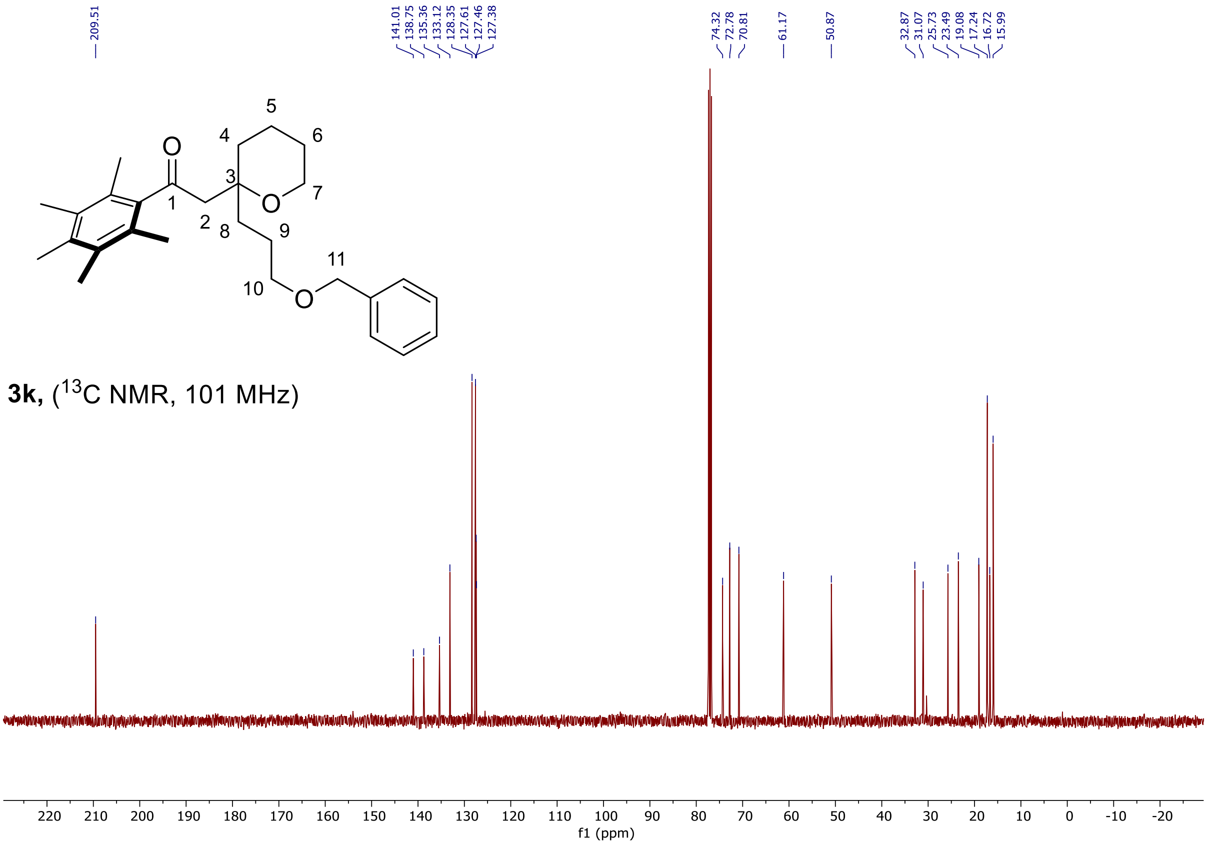

3k, (^{13}C NMR, 101 MHz)

NMR spectra of cyclohexenes

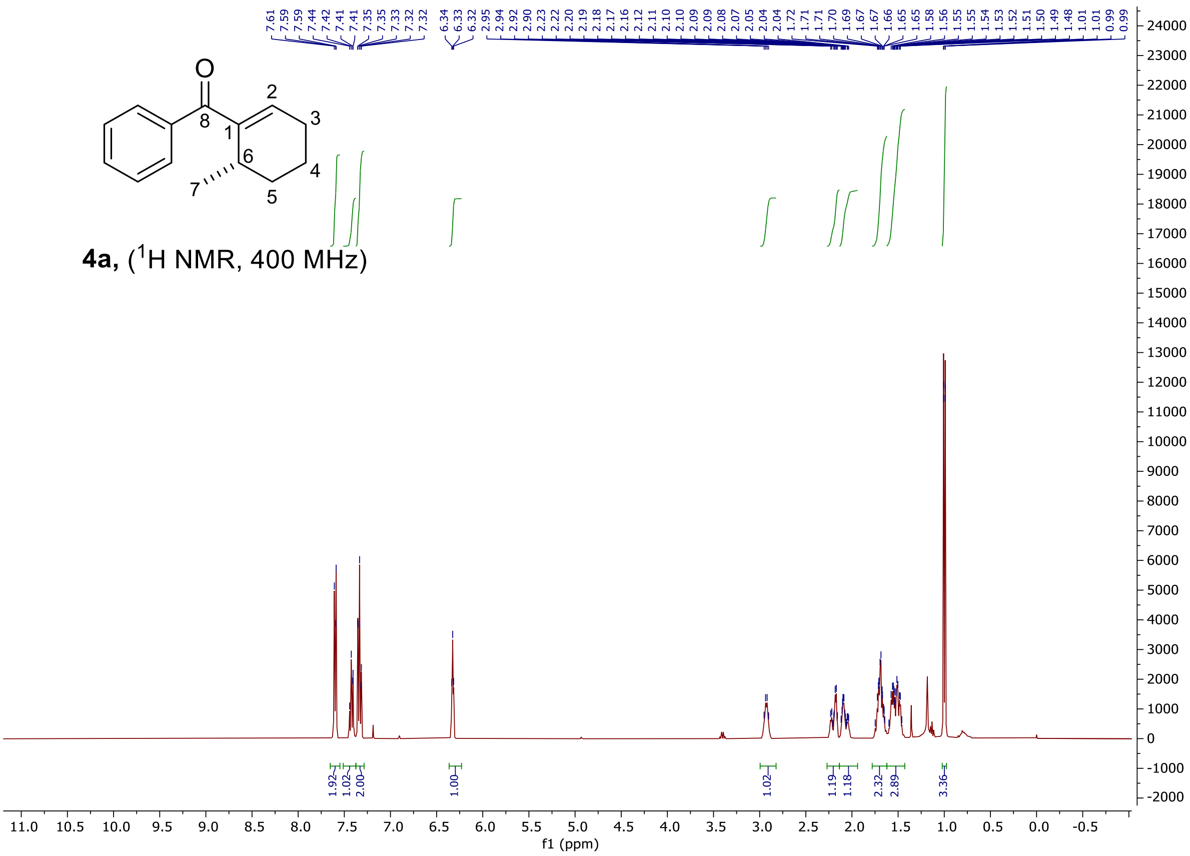

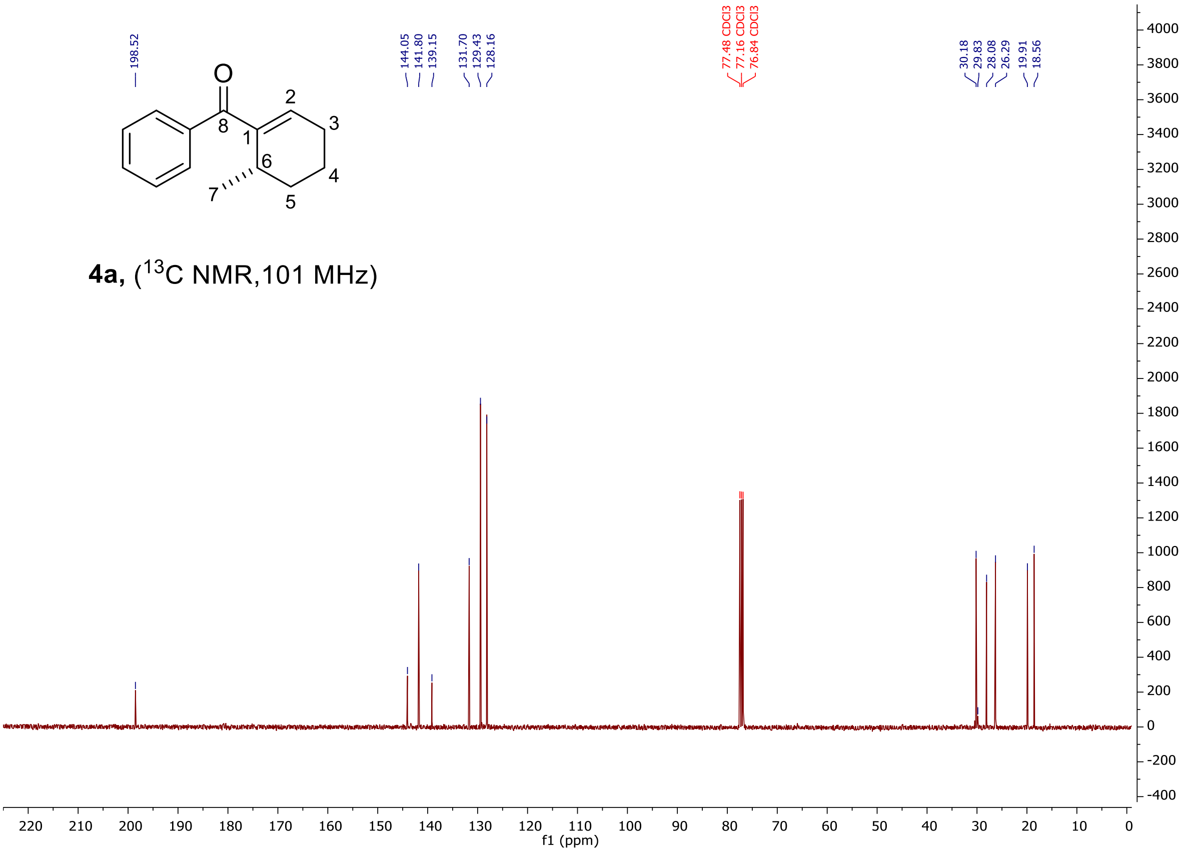

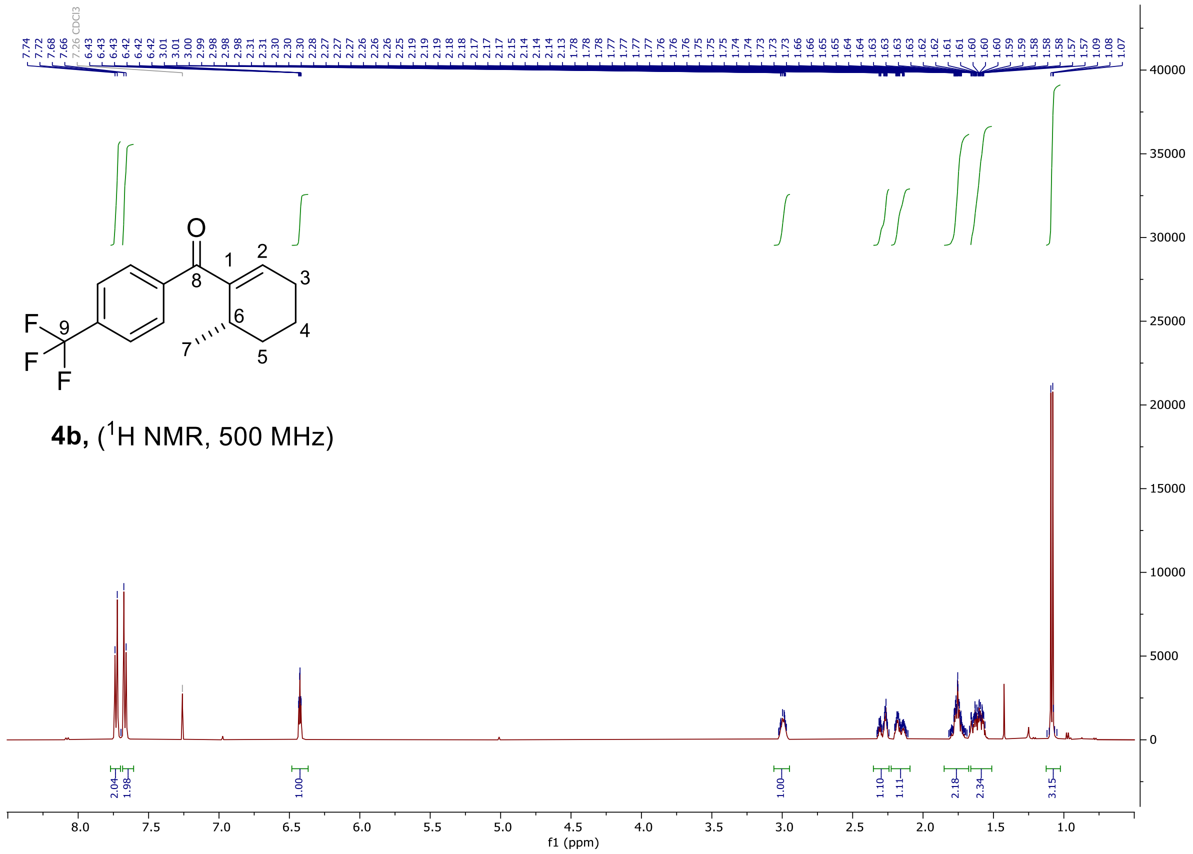
4b, (^{1}H NMR, 500 MHz)
f1 (ppm)

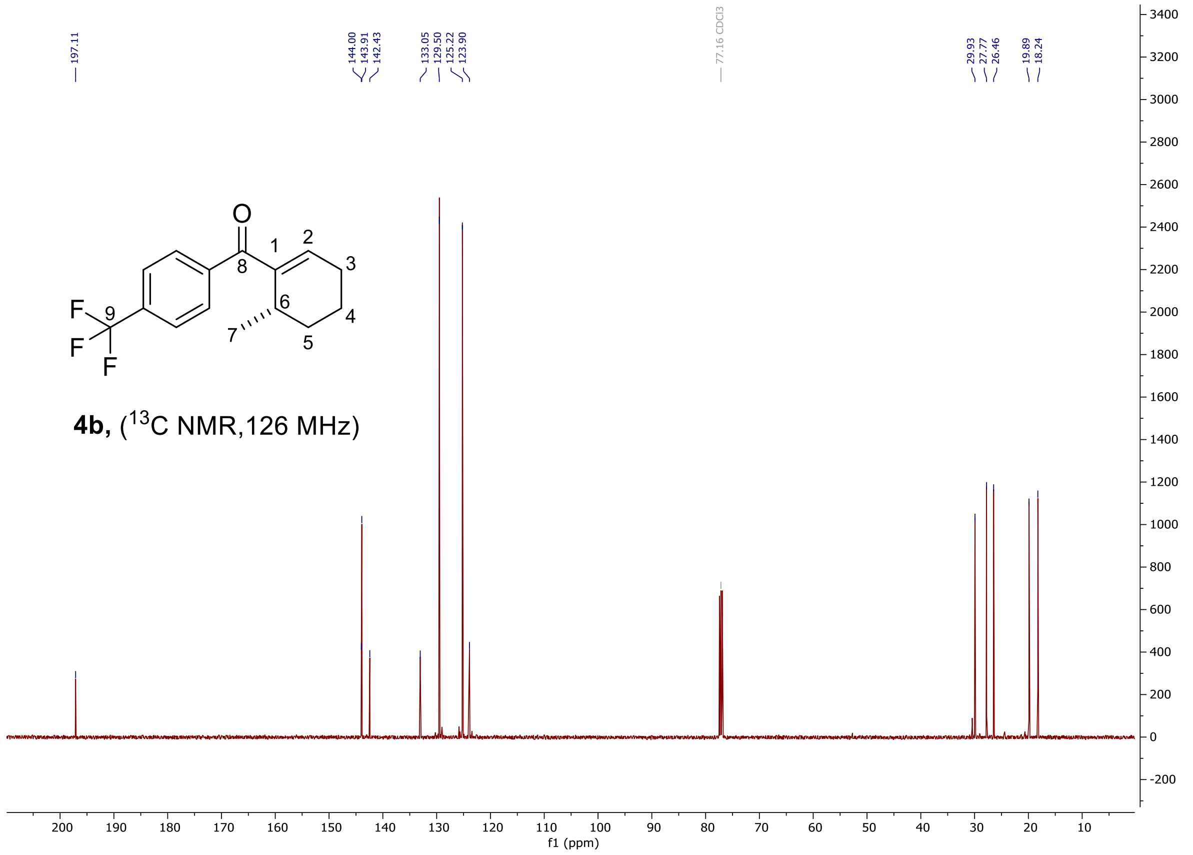
4b, (^{13}C NMR,126 MHz)
f1 (ppm)

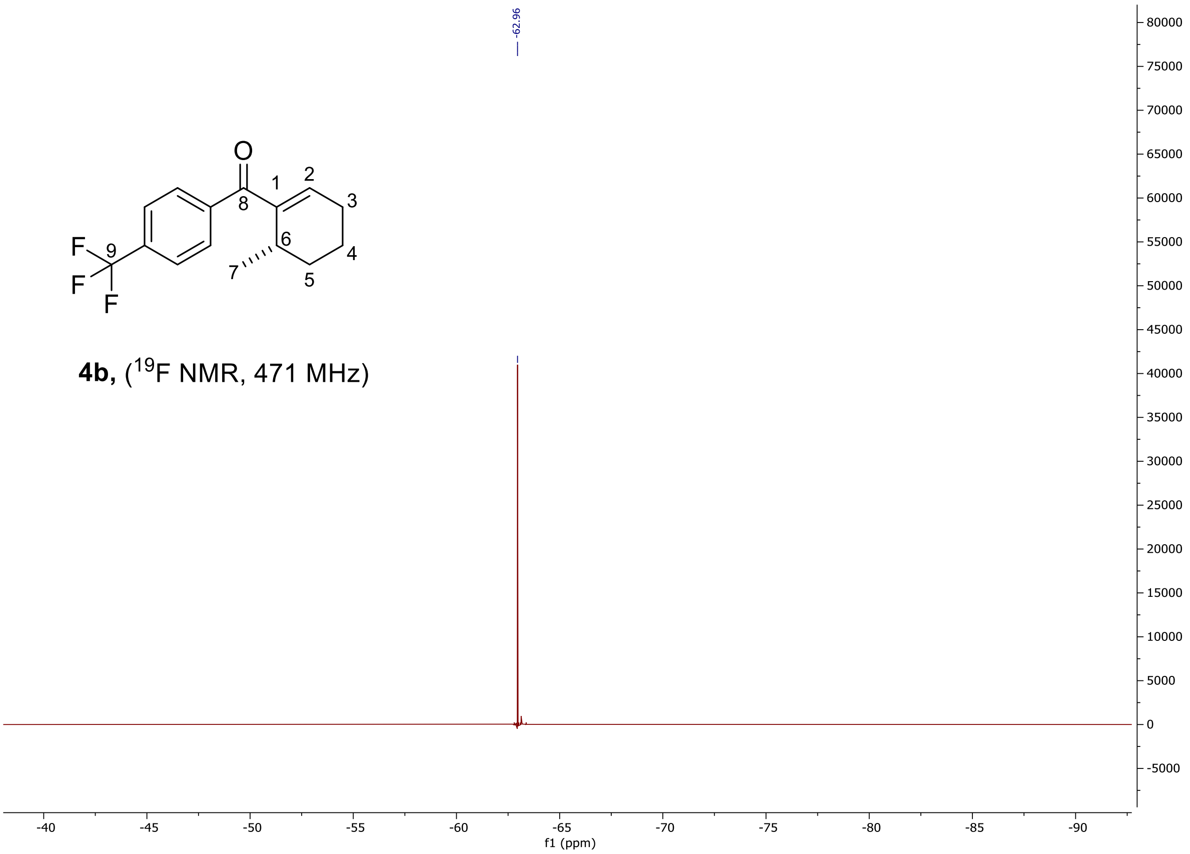
-62.96
O
1
2
3
4
5
6
7
8
9
F
F
F
4b, (^{19}F NMR, 471 MHz)
80000
75000
70000
65000
60000
55000
50000
45000
40000
35000
30000
25000
20000
15000
10000
5000
0
-5000
-40
-45
-50
-55
-60
-65
-70
-75
-80
-85
-90
f1 (ppm)

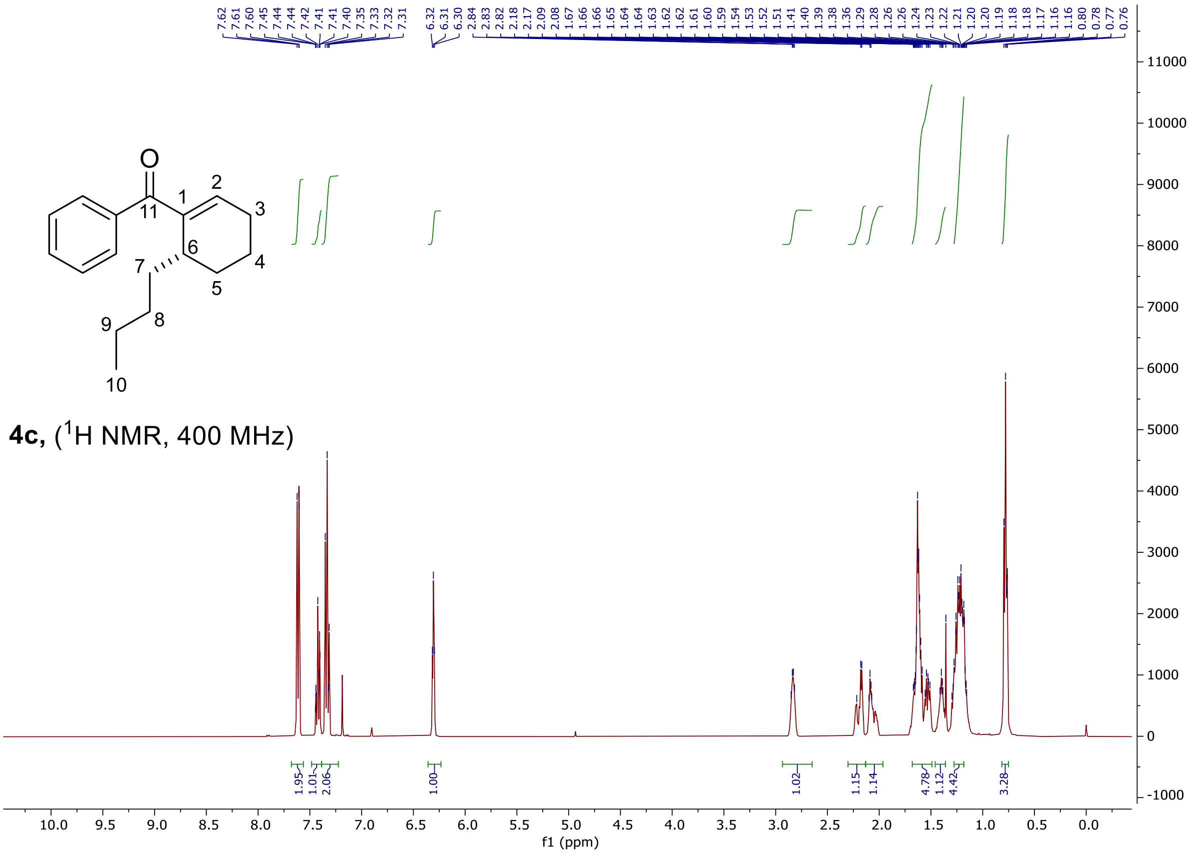
4c, (^{1}H NMR, 400 MHz)
f1 (ppm)

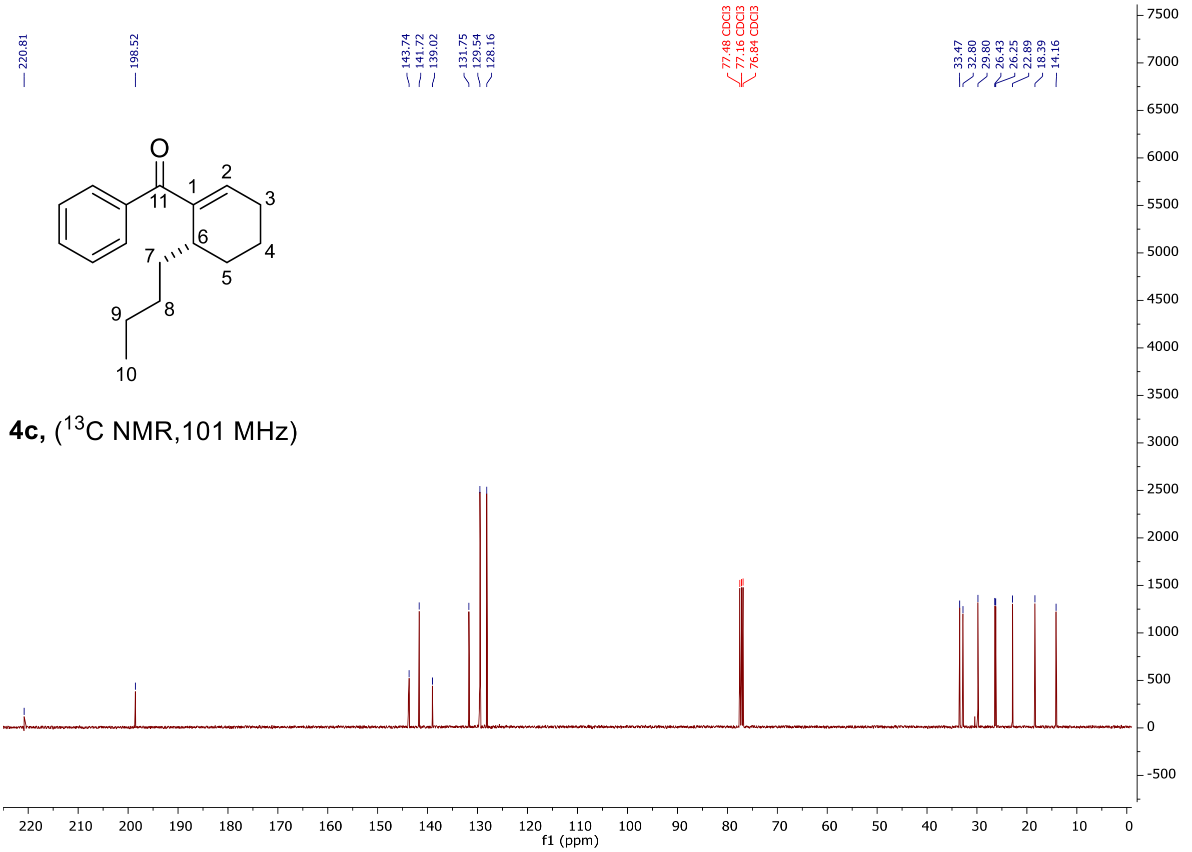
4c, (^{13}C NMR,101 MHz)
f1 (ppm)

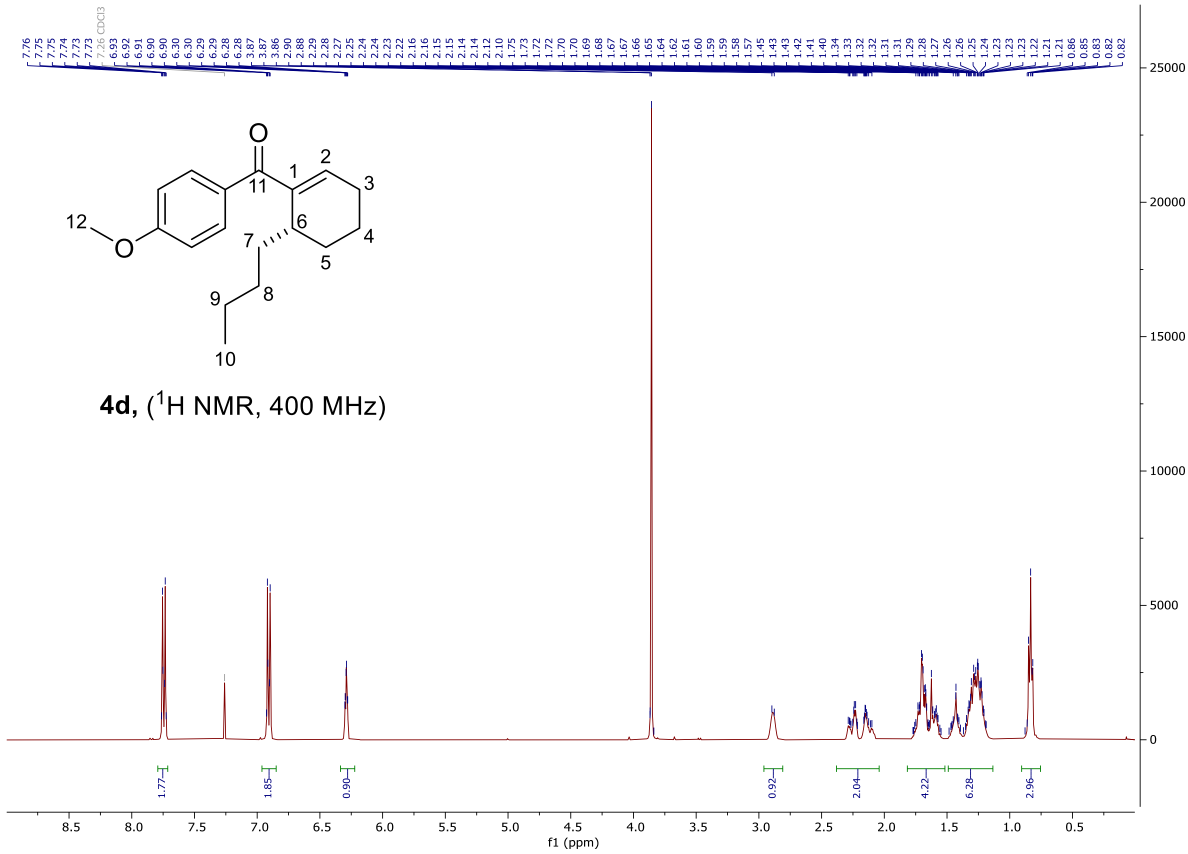
4d, (¹H NMR, 400 MHz)

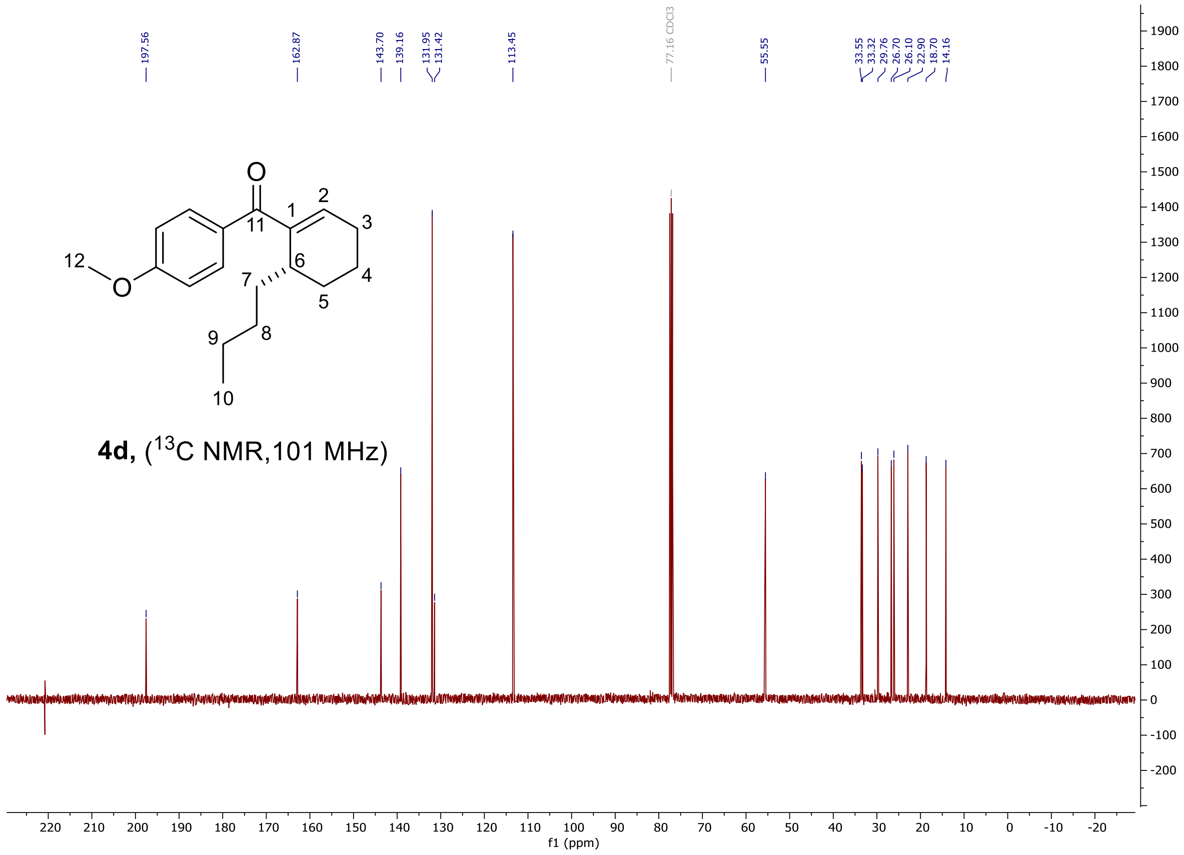
4d, (¹³C NMR,101 MHz)

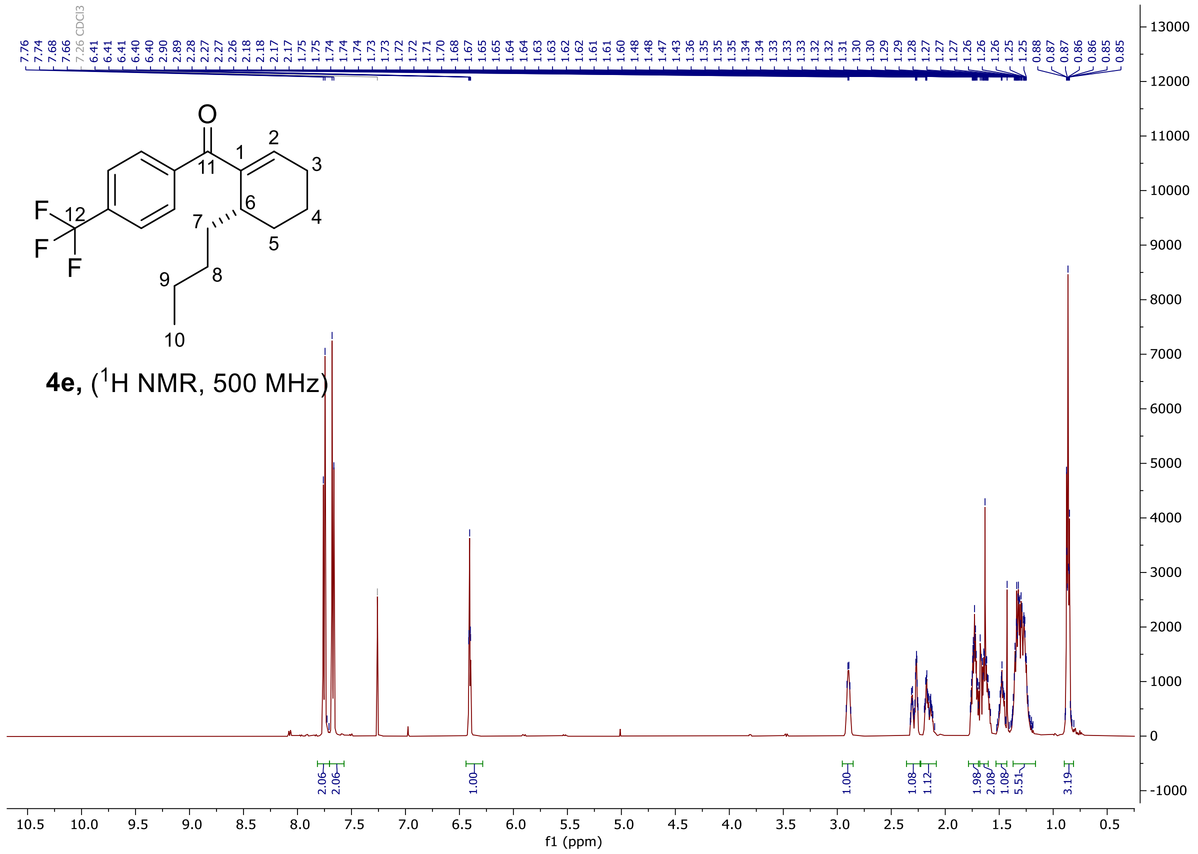
7.76
7.74
7.68
7.66
7.26 CDCl3
6.41
6.41
6.41
6.40
6.40
2.90
2.89
2.28
2.27
2.27
2.26
2.18
2.18
2.17
2.17
1.75
1.75
1.74
1.74
1.74
1.73
1.73
1.72
1.72
1.71
1.70
1.68
1.67
1.65
1.65
1.64
1.63
1.63
1.62
1.62
1.61
1.61
1.60
1.48
1.48
1.47
1.43
1.36
1.35
1.35
1.35
1.34
1.34
1.33
1.33
1.33
1.33
1.32
1.32
1.31
1.30
1.30
1.29
1.29
1.28
1.27
1.27
1.27
1.26
1.26
1.26
1.25
1.25
0.88
0.87
0.87
0.86
0.86
0.85
0.85
O
1
2
3
11
F
12
6
F
7
4
F
5
9
8
10
4e, (1H NMR, 500 MHz)
2.06
2.06
1.00
1.00
1.08
1.12
1.98
2.08
1.08
5.51
3.19
f1 (ppm)

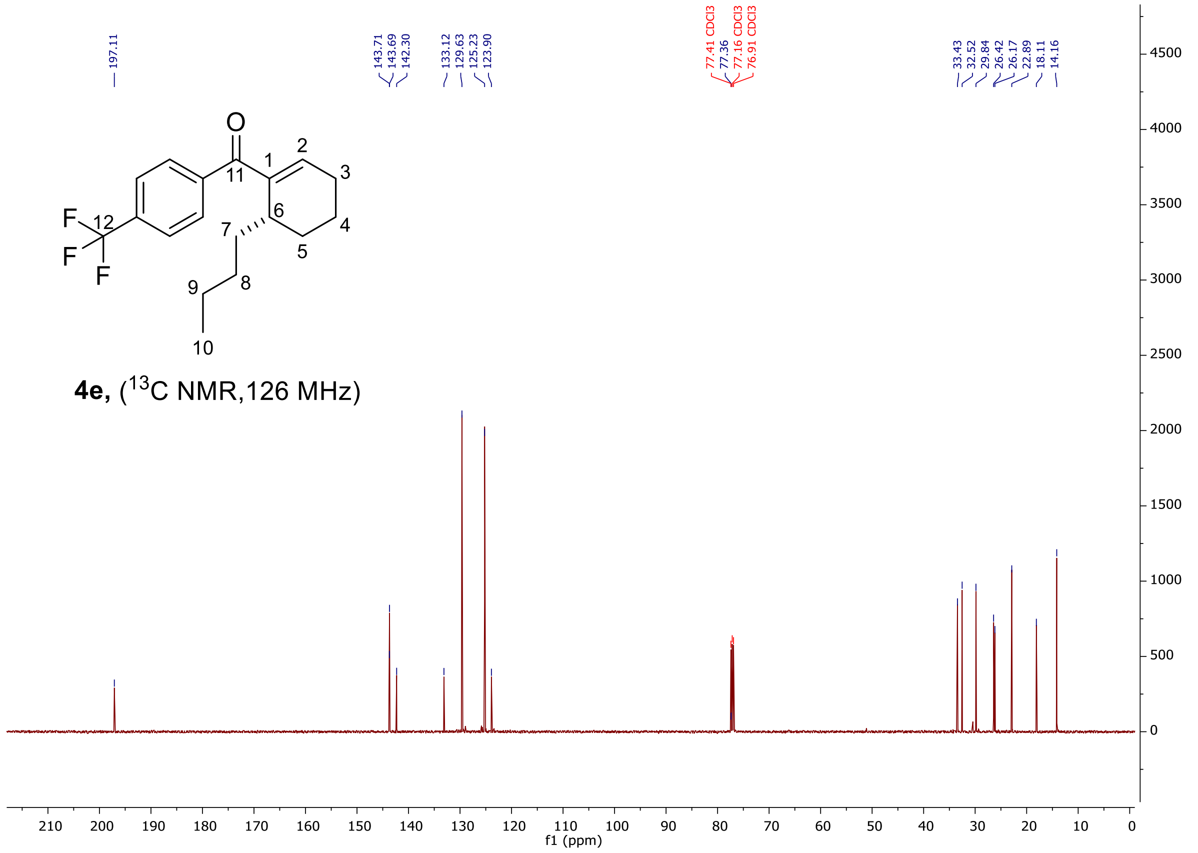
197.11
143.71
143.69
142.30
133.12
129.63
125.23
123.90
77.41 CDCl3
77.36
77.16 CDCl3
76.91 CDCl3
33.43
32.52
29.84
26.42
26.17
22.89
18.11
14.16
O
1
2
3
11
F
12
6
F
7
4
F
5
9
8
10
4e, (13C NMR,126 MHz)
f1 (ppm)

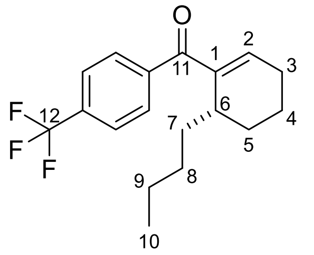

4e, (^{19}F NMR, 471 MHz)

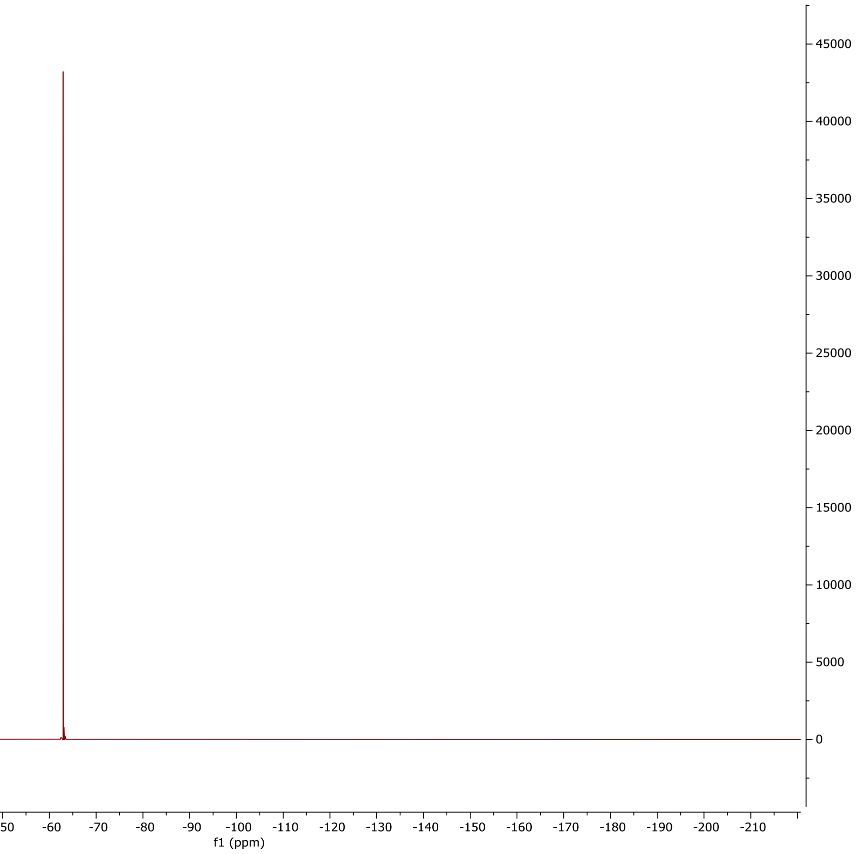

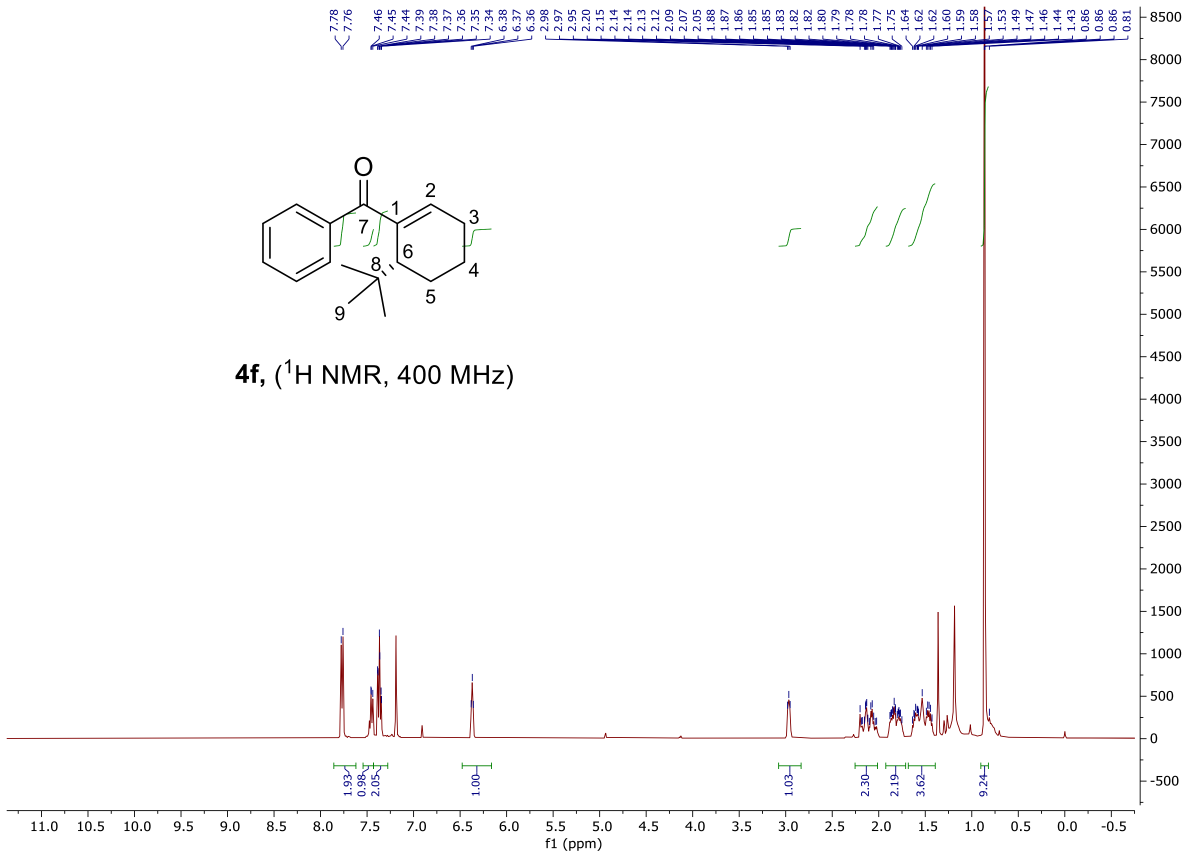
4f, (^{1}H NMR, 400 MHz)
7.78 7.76 7.46 7.45 7.44 7.39 7.38 7.37 7.36 7.35 7.34 6.38 6.37 6.36 2.98 2.97 2.95 2.20 2.15 2.14 2.14 2.13 2.12 2.09 2.07 2.05 1.88 1.87 1.86 1.85 1.85 1.83 1.82 1.82 1.80 1.79 1.78 1.78 1.77 1.75 1.64 1.62 1.62 1.60 1.59 1.58 1.57 1.53 1.49 1.47 1.46 1.44 1.43 0.86 0.86 0.86 0.81
1.93 0.98 2.05 1.00 1.03 2.30 2.19 3.62 9.24
f1 (ppm)

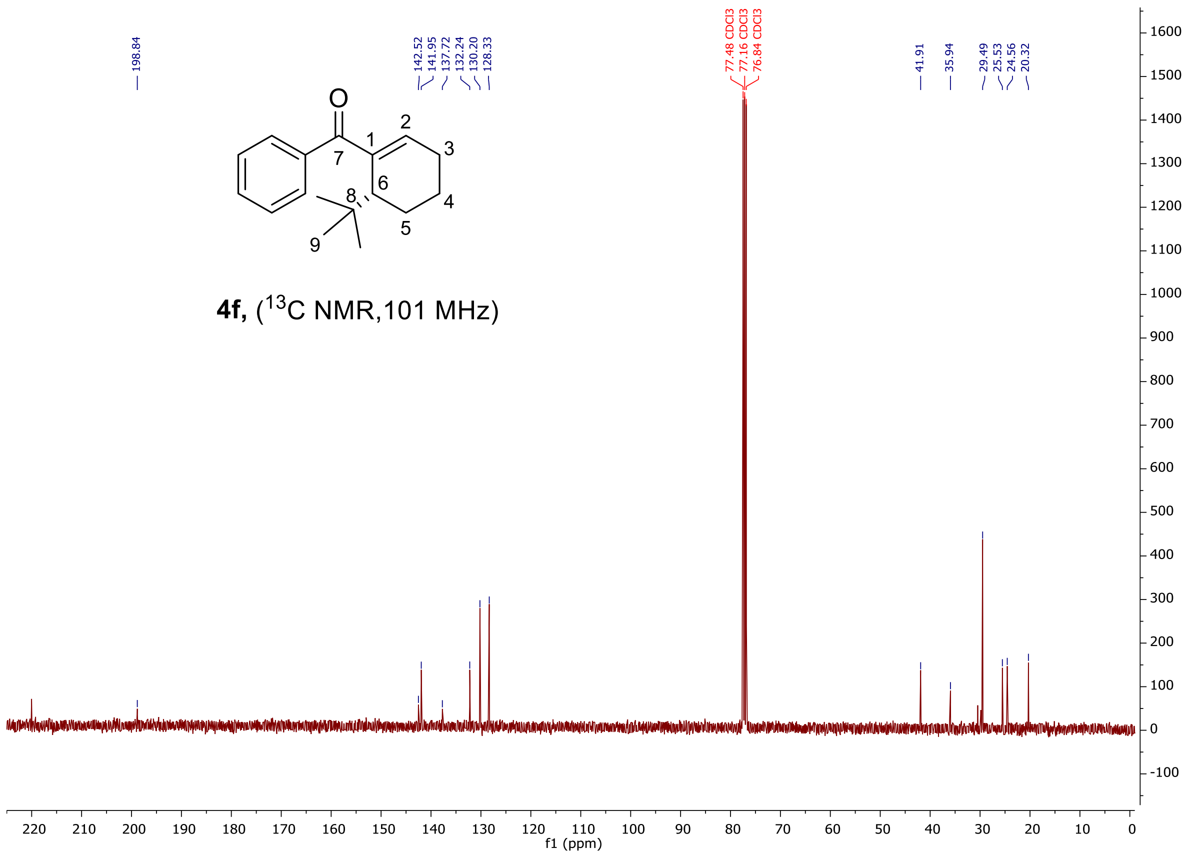
4f, (^{13}C NMR,101 MHz)
198.84
142.52 141.95 137.72 132.24 130.20 128.33
77.48 CDCl3 77.16 CDCl3 76.84 CDCl3
41.91 35.94 29.49 25.53 24.56 20.32
f1 (ppm)

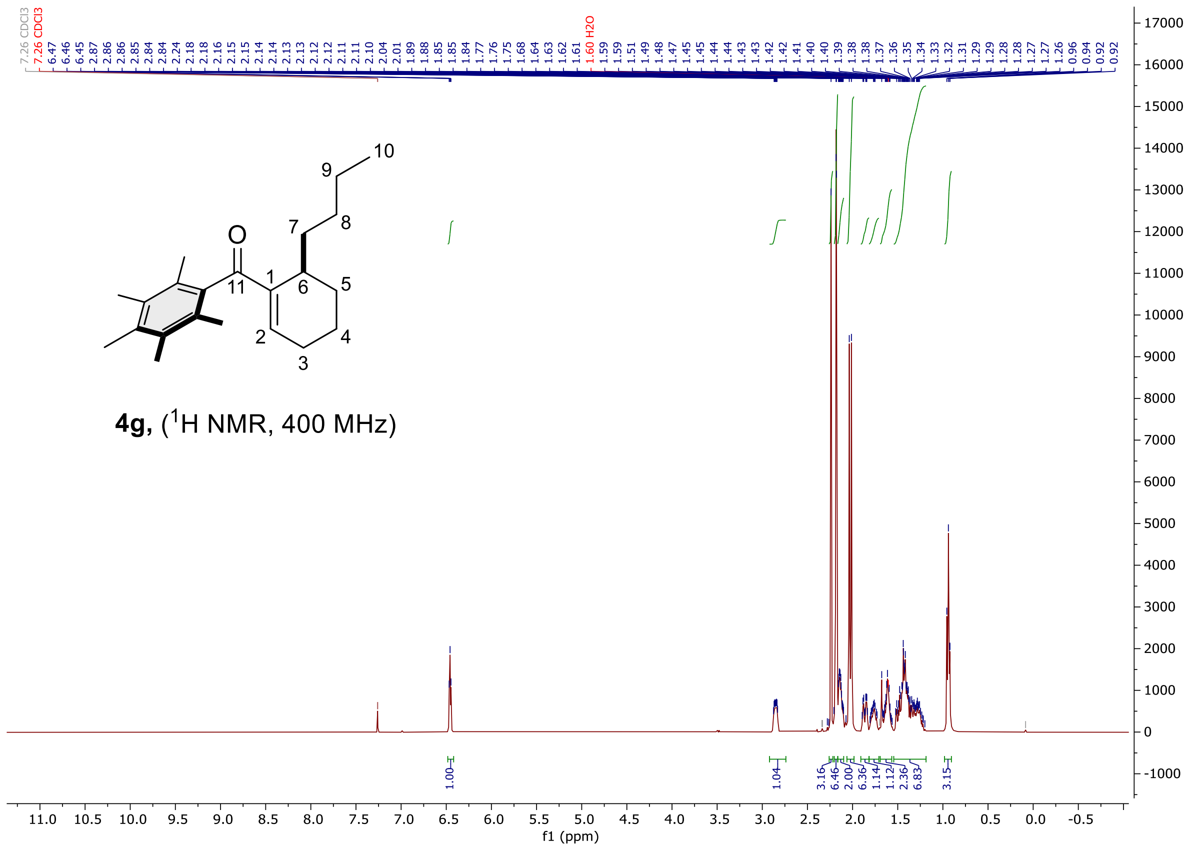

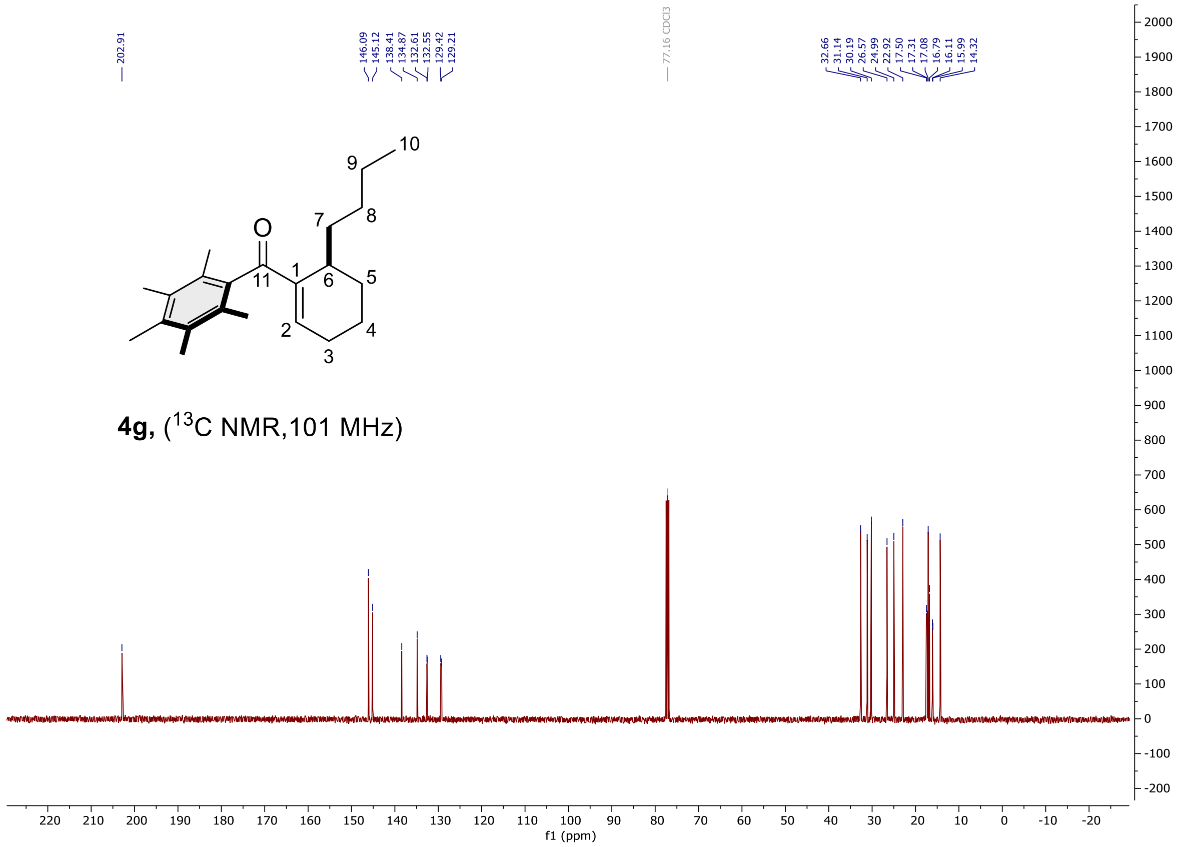

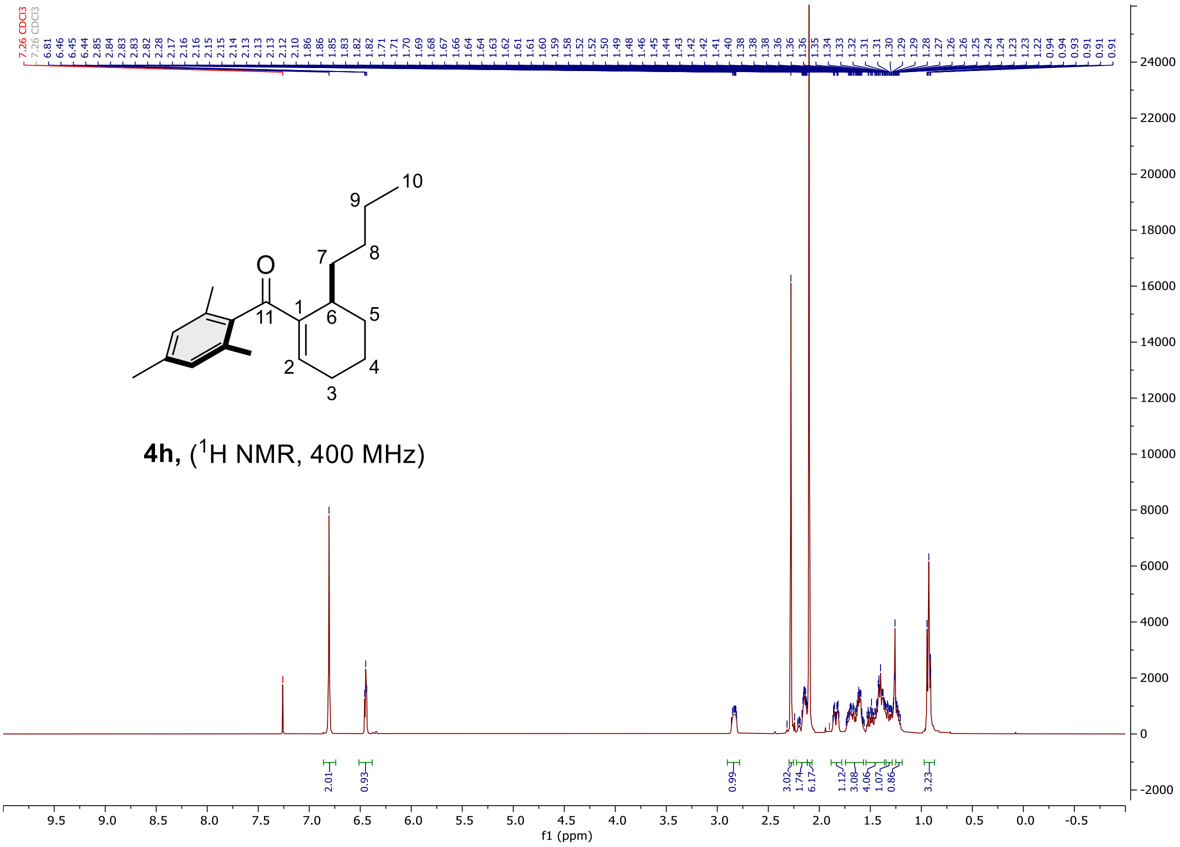
4h, (1H NMR, 400 MHz)
f1 (ppm)

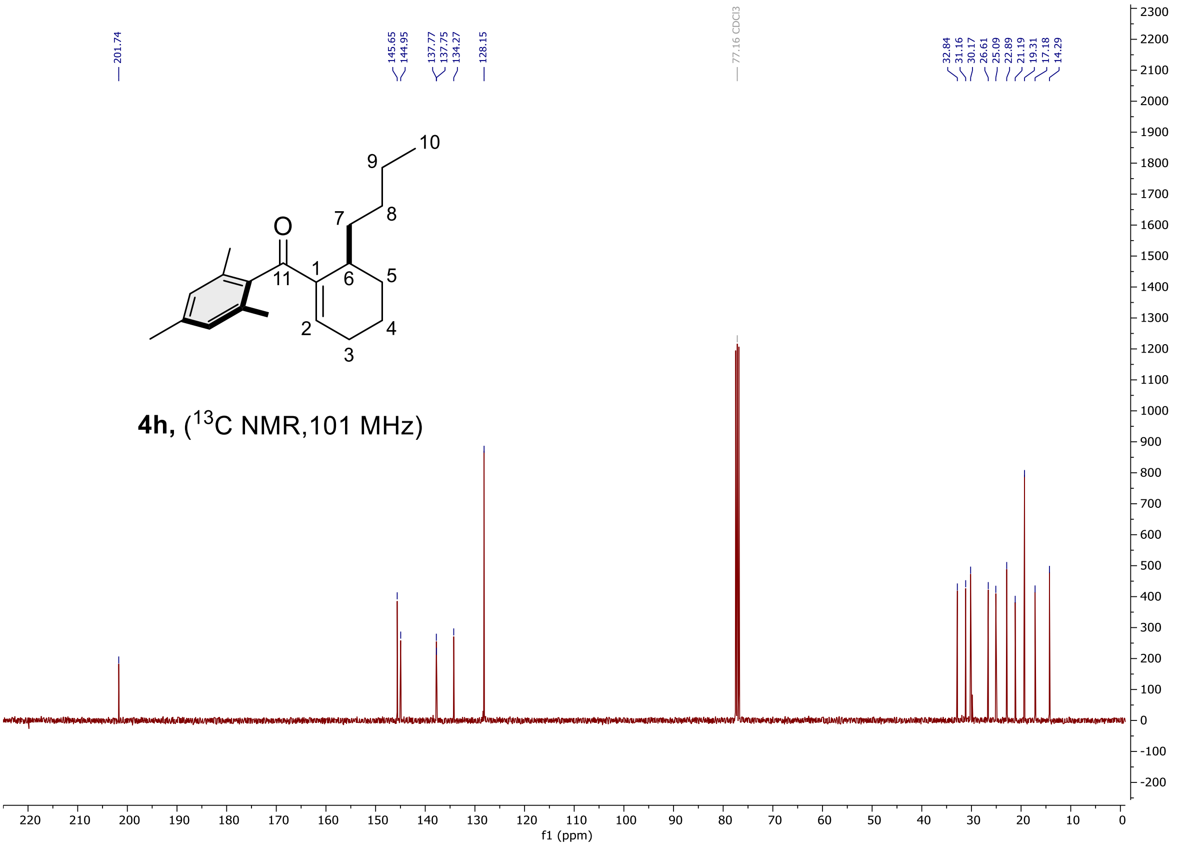
4h, (13C NMR,101 MHz)
201.74
145.65
144.95
137.77
137.75
134.27
128.15
77.16 CDCl3
32.84
31.16
30.17
26.61
25.09
22.89
21.19
19.31
17.18
14.29
f1 (ppm)

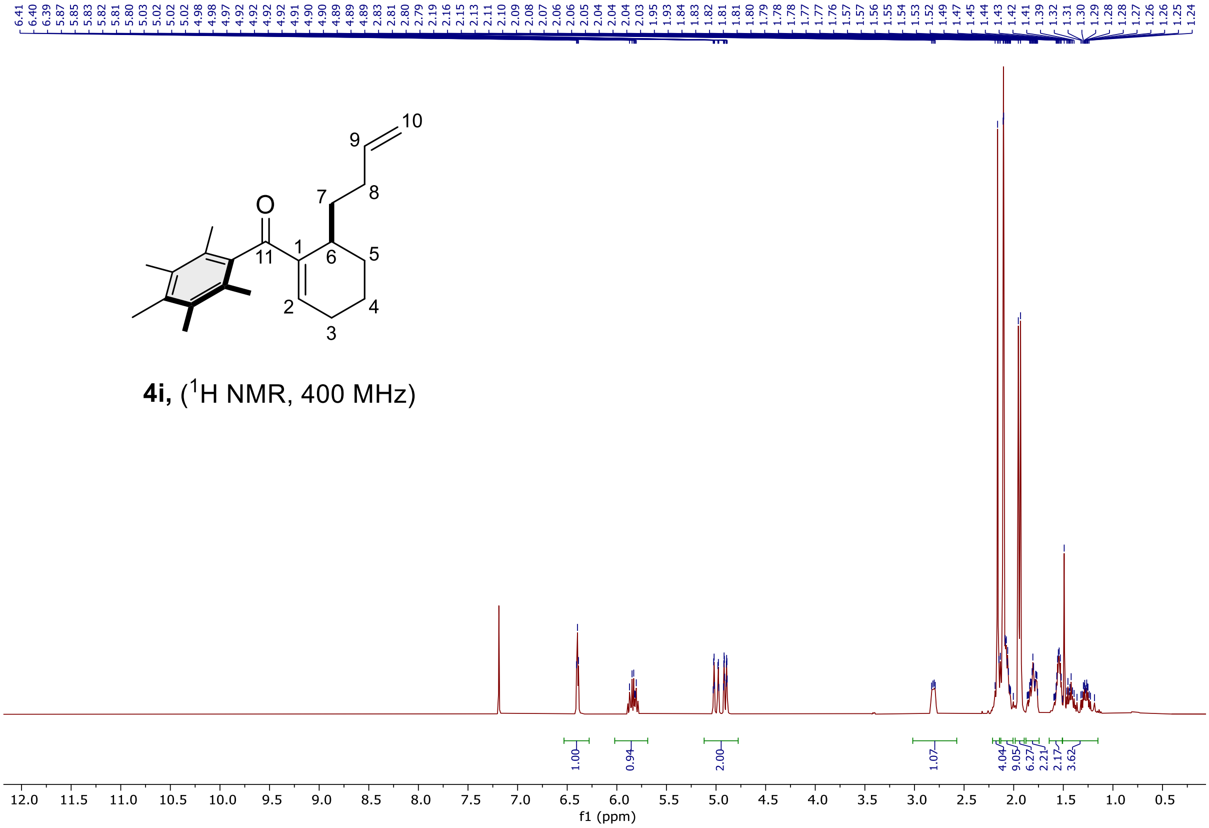
4i, (1H NMR, 400 MHz)
f1 (ppm)

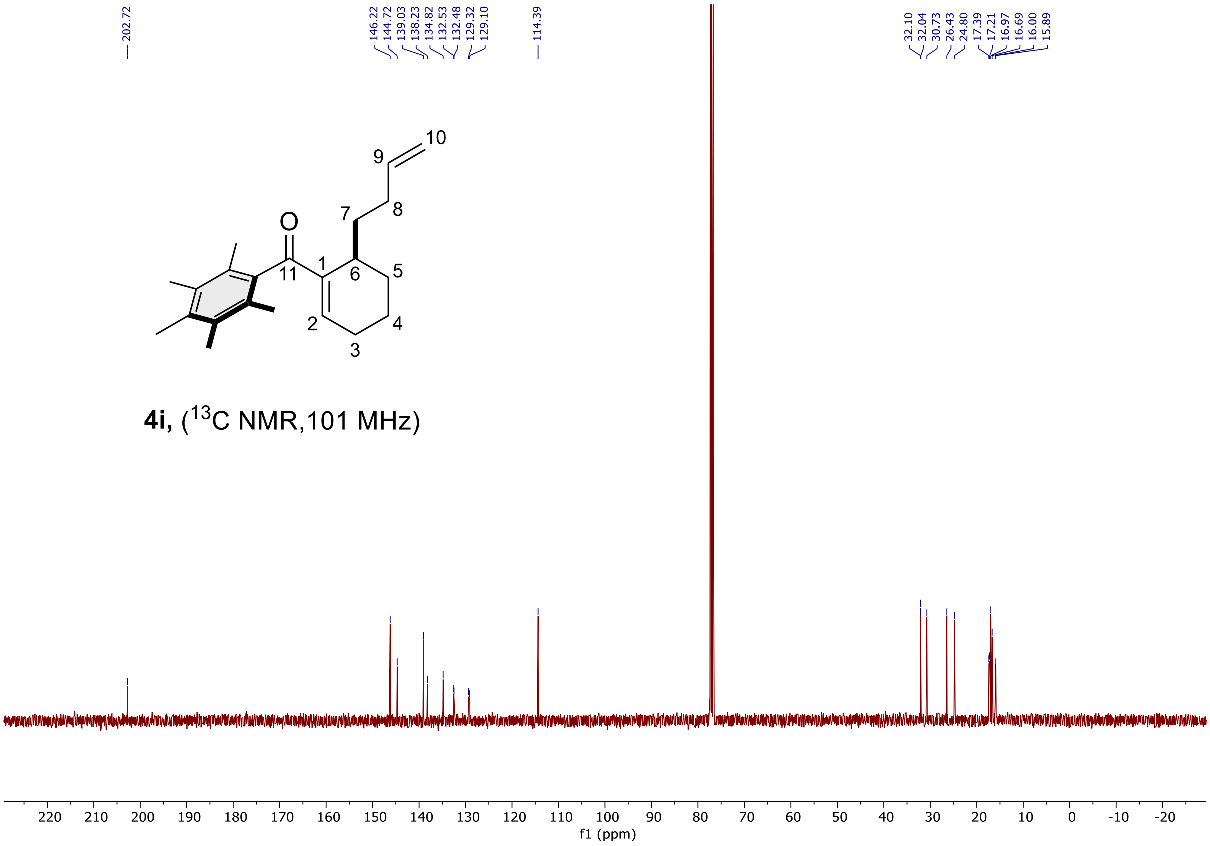
4i, (13C NMR,101 MHz)
f1 (ppm)

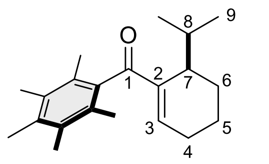

4j, (^{1}H NMR, 400 MHz)

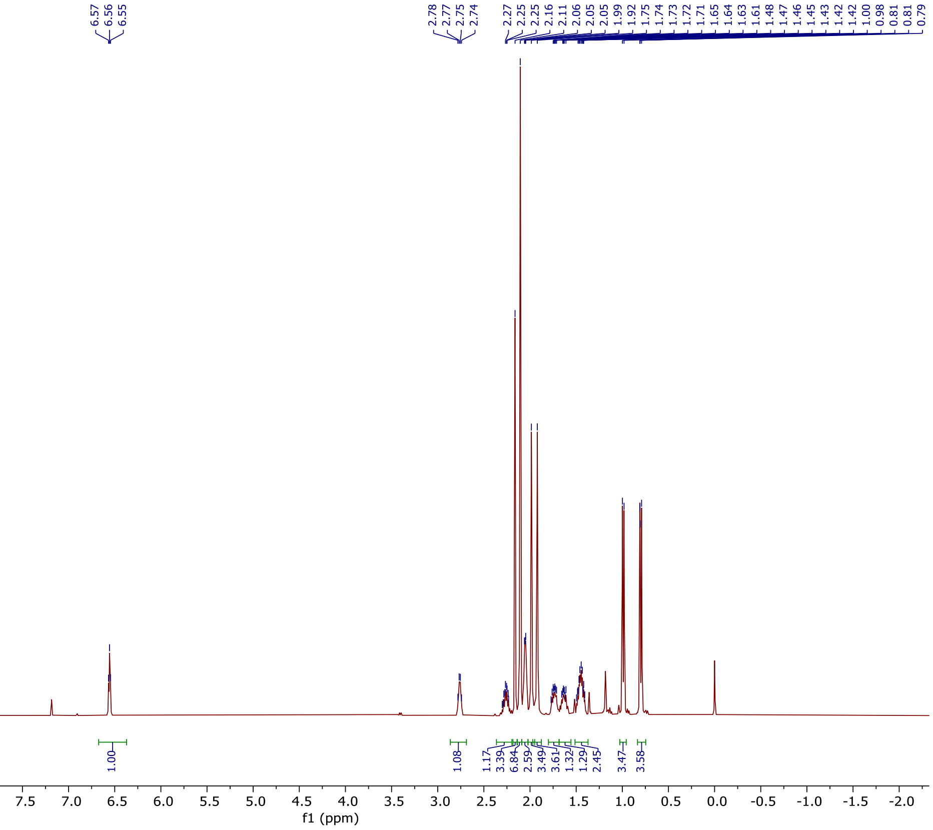

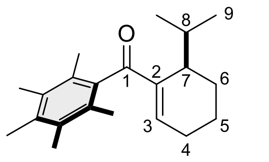

4j, (^{13}C NMR,101 MHz)

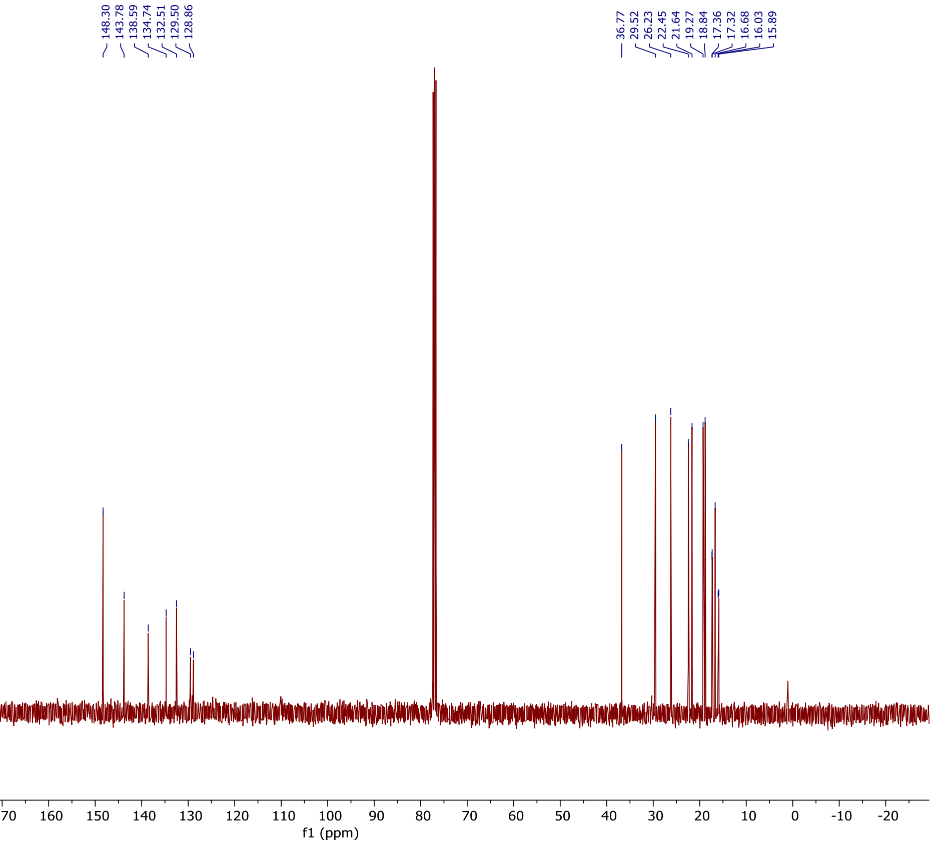

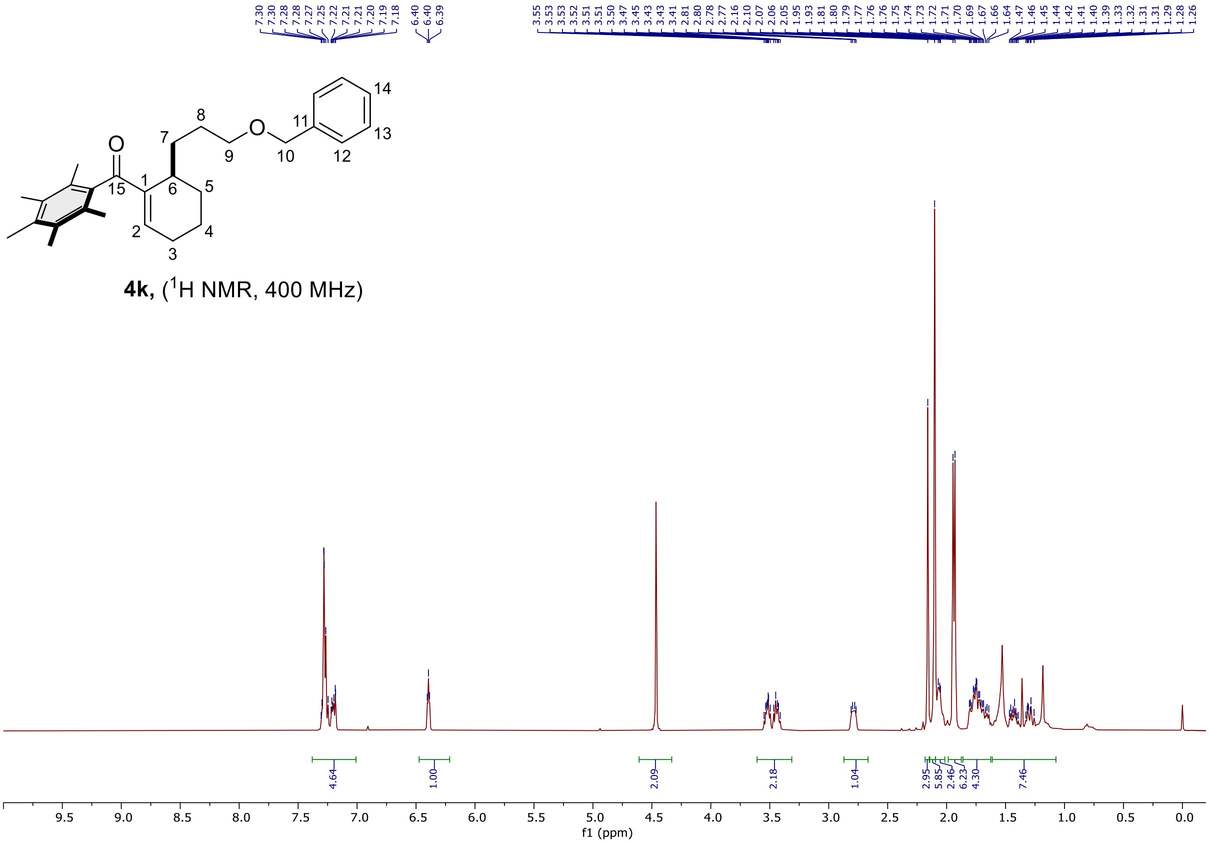

4k, (^{1}H NMR, 400 MHz)

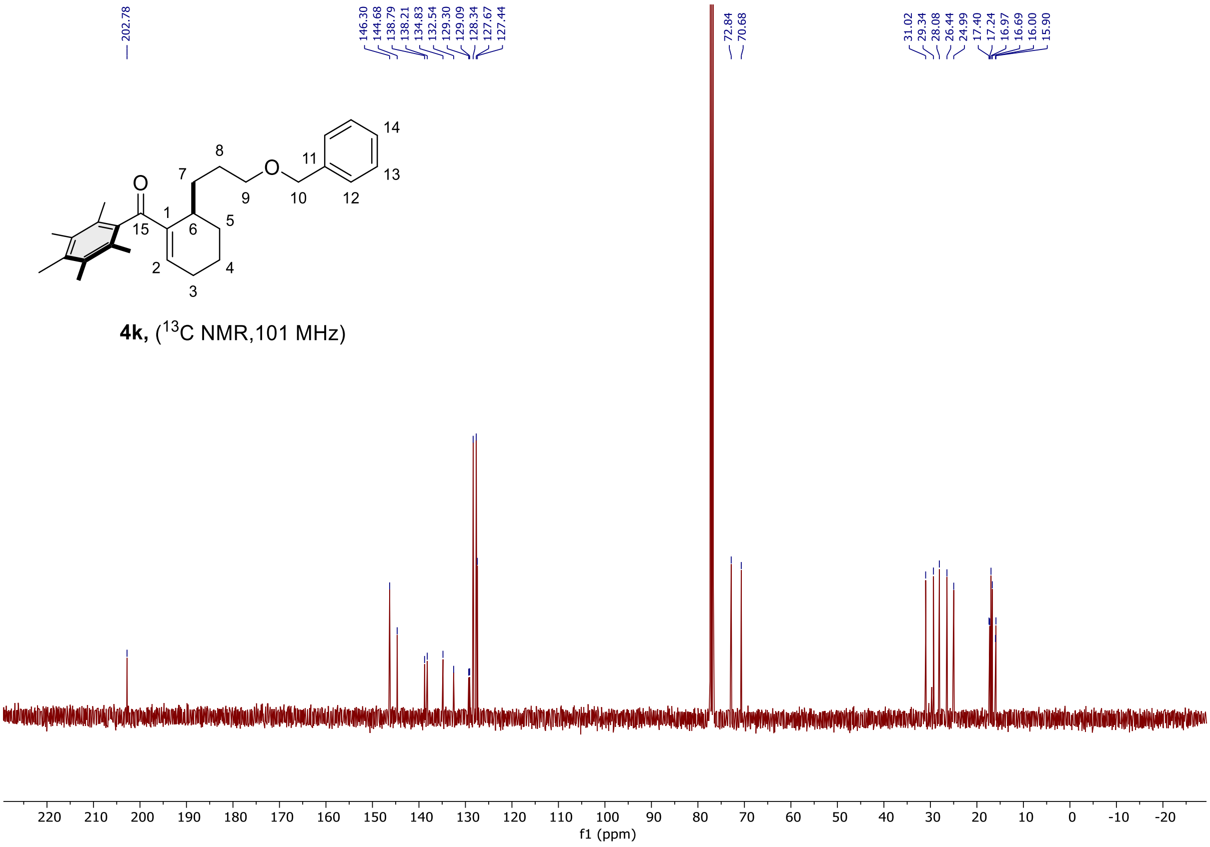

4k, (^{13}C NMR,101 MHz)

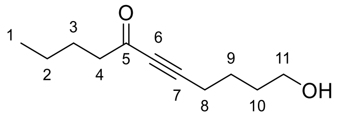

S21, (^{1}H NMR, 400 MHz)

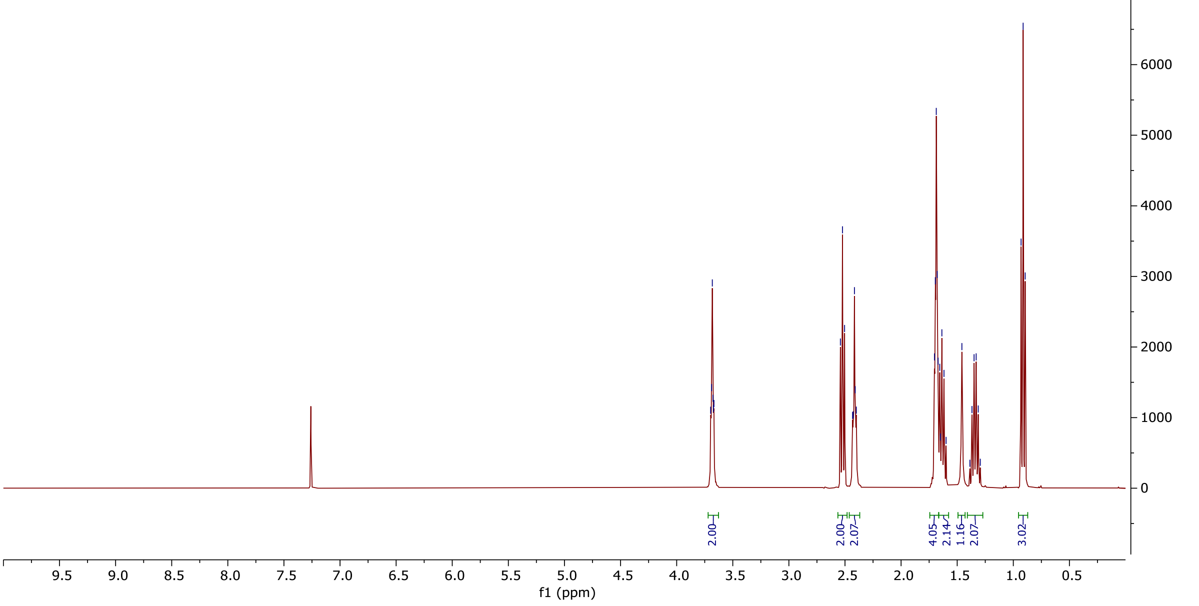

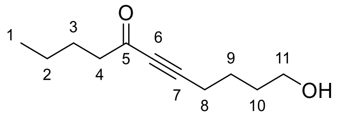

S21, (^{13}C NMR,101 MHz)

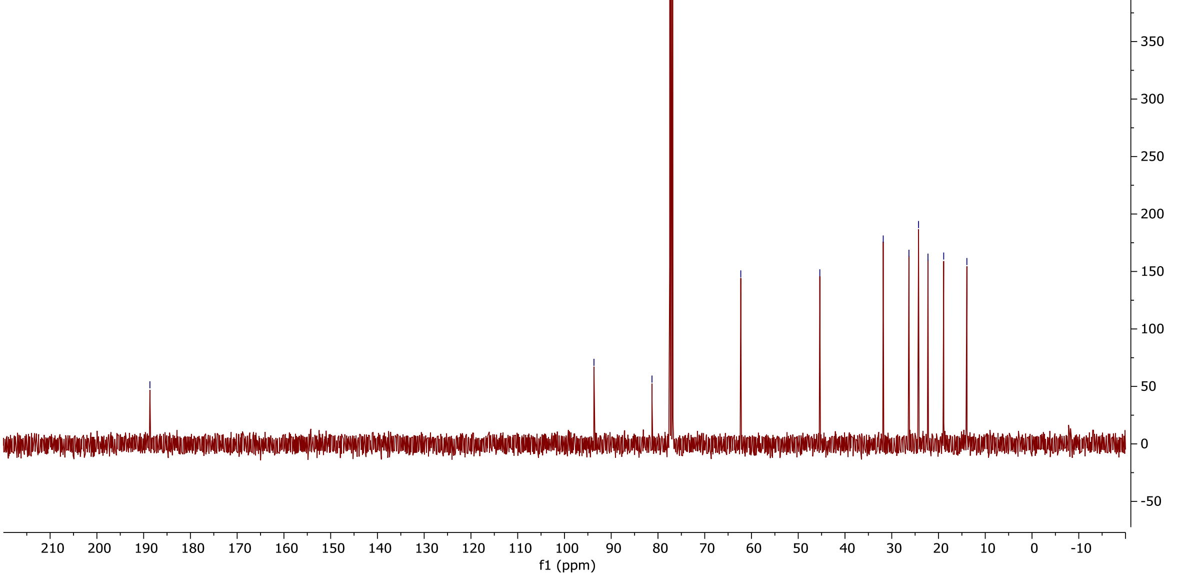

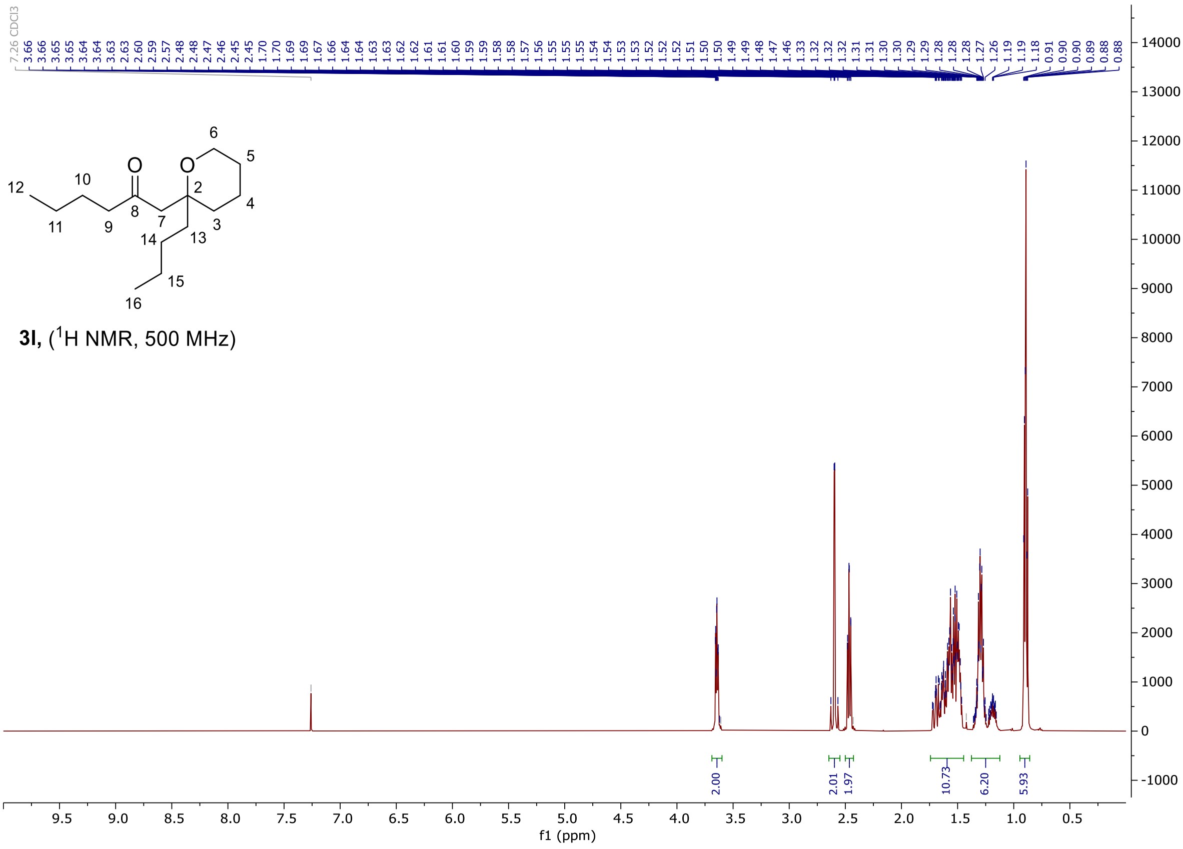

3l, (^{1}H NMR, 500 MHz)

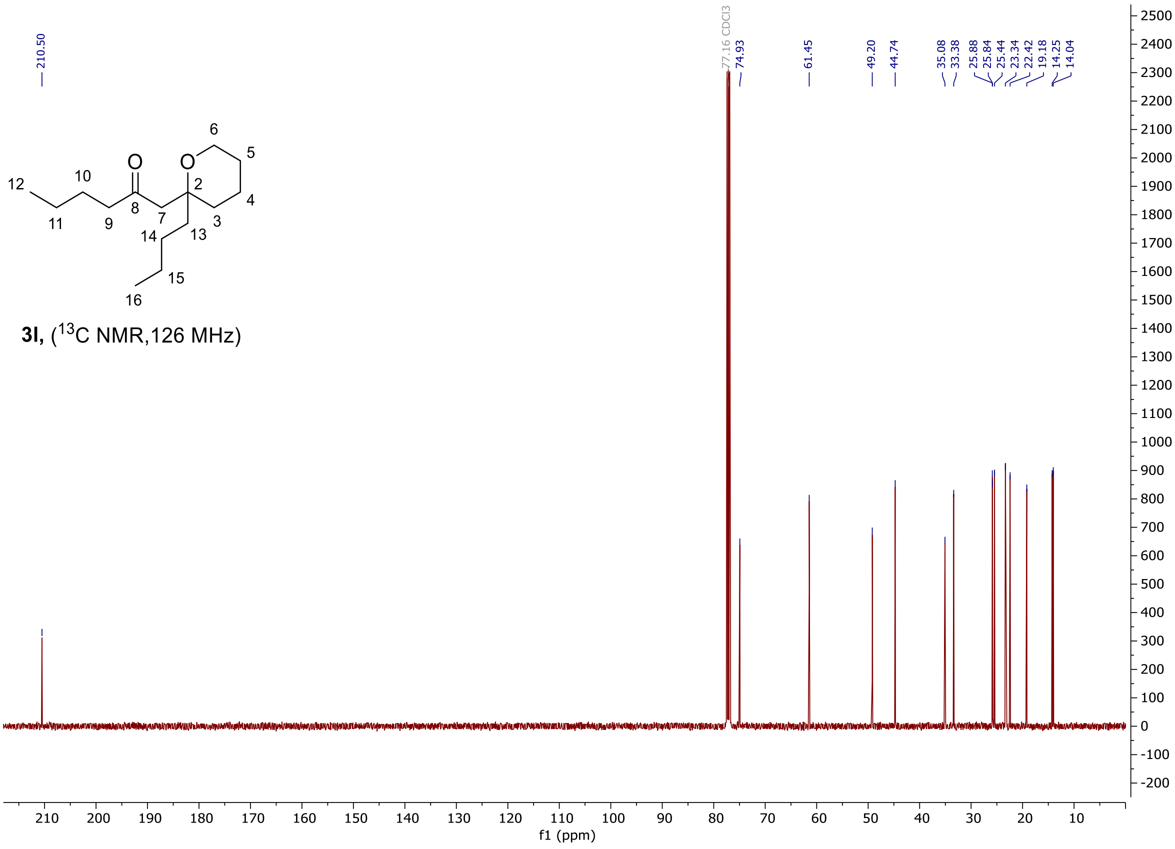

3l, (^{13}C NMR,126 MHz)

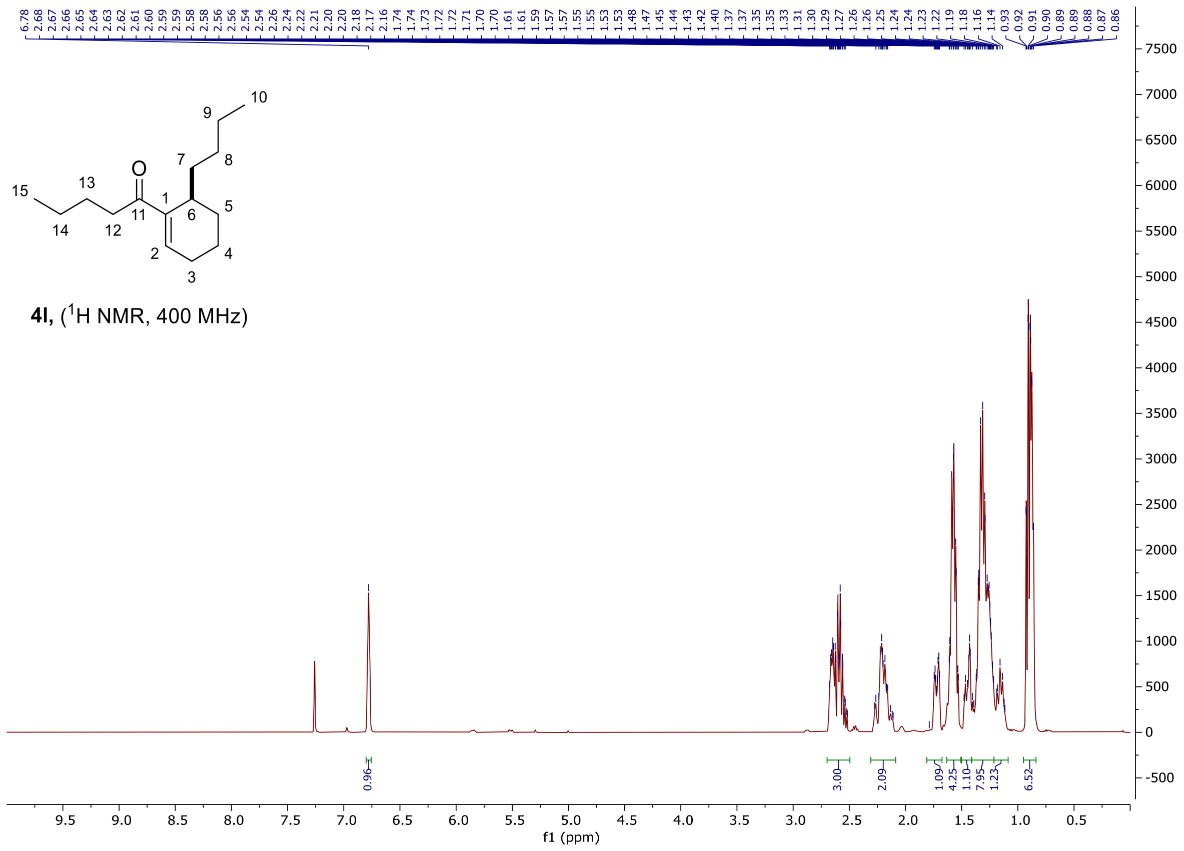

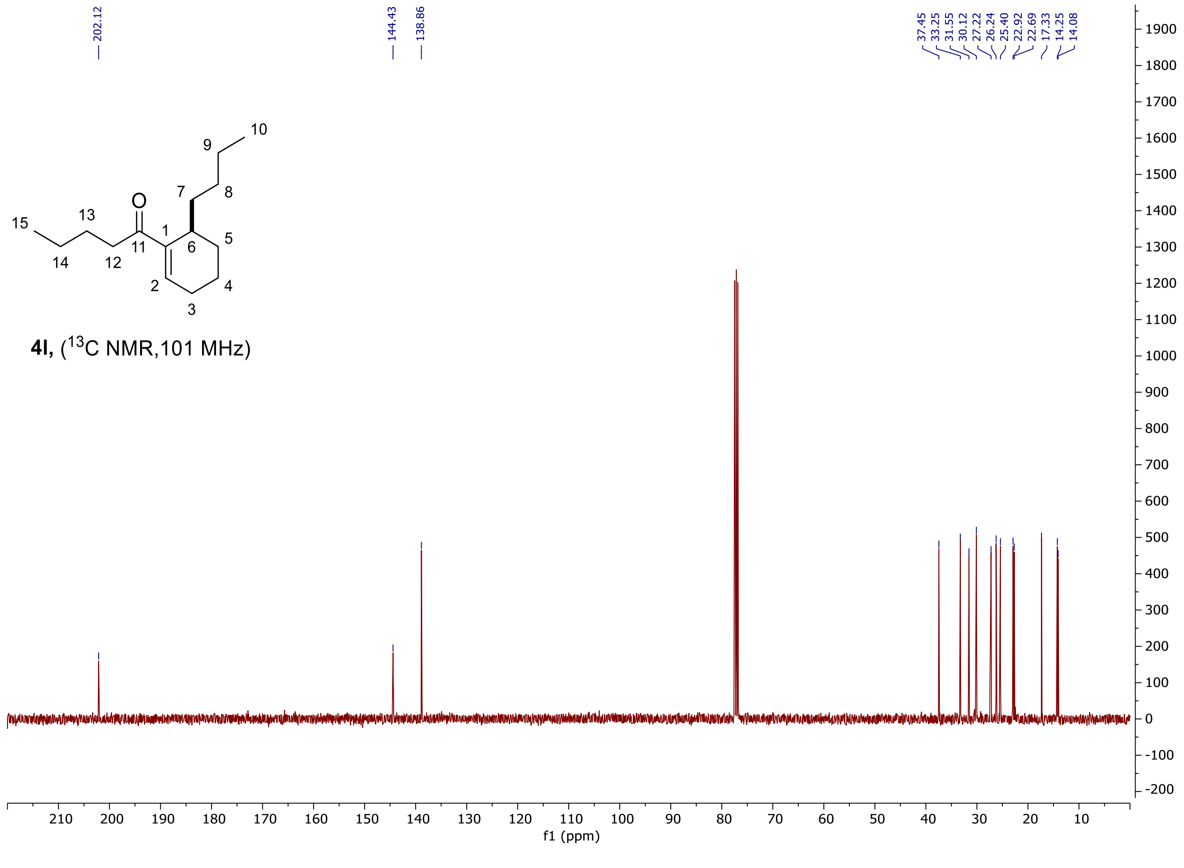

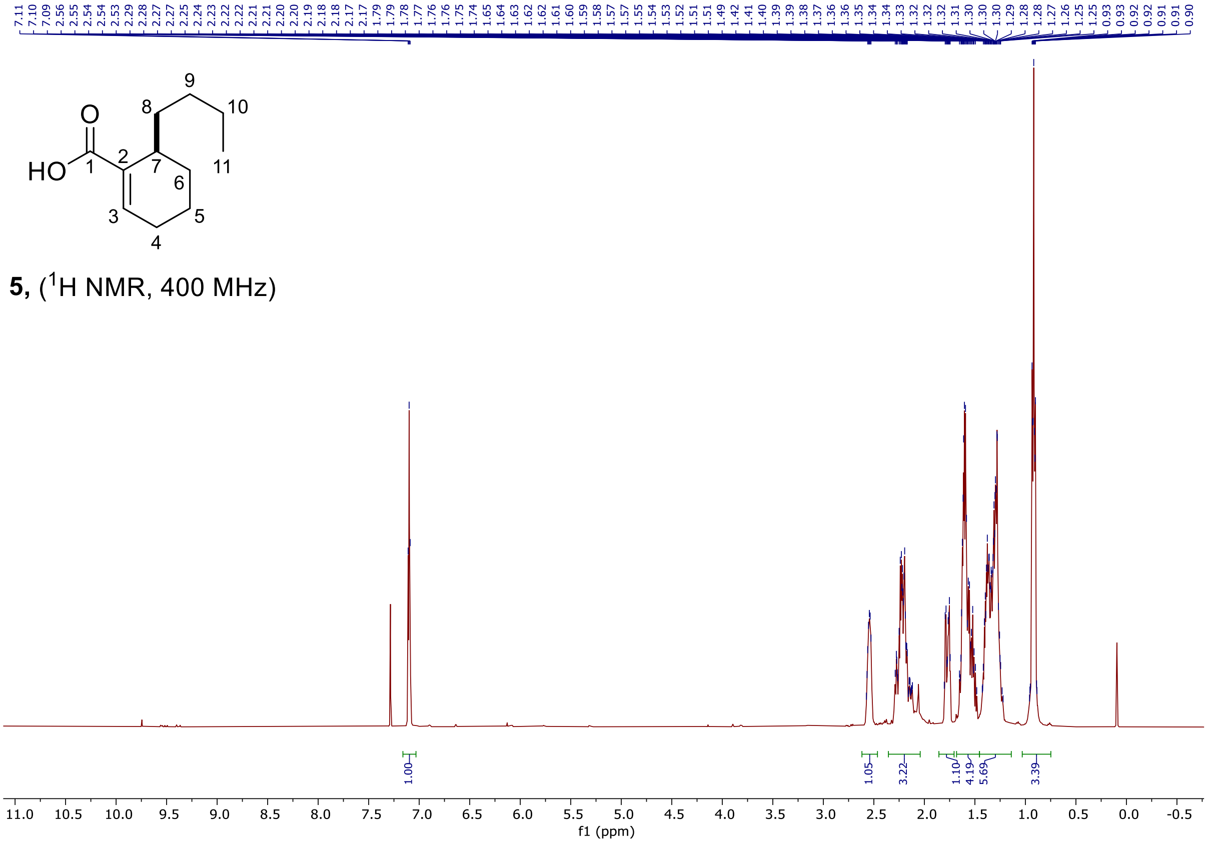

5, (^{1}H NMR, 400 MHz)

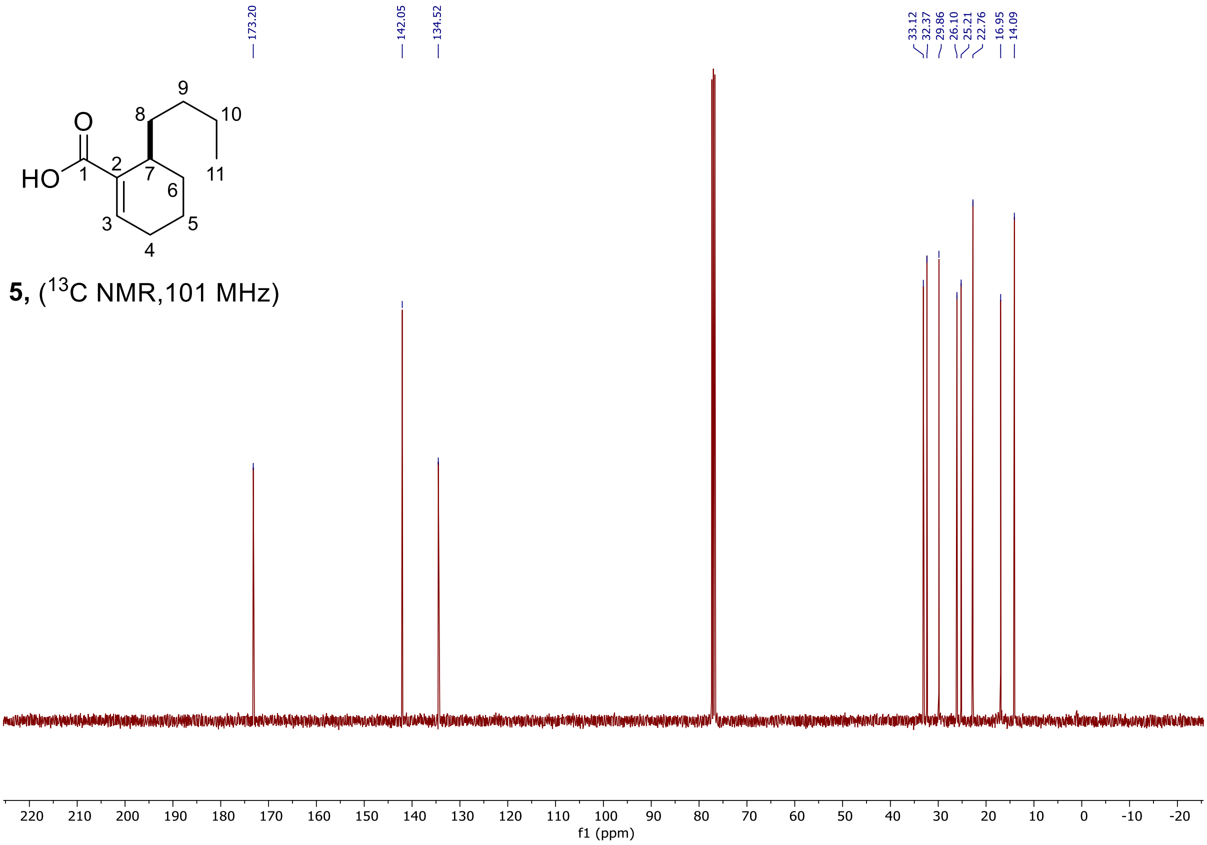

5, (^{13}C NMR,101 MHz)

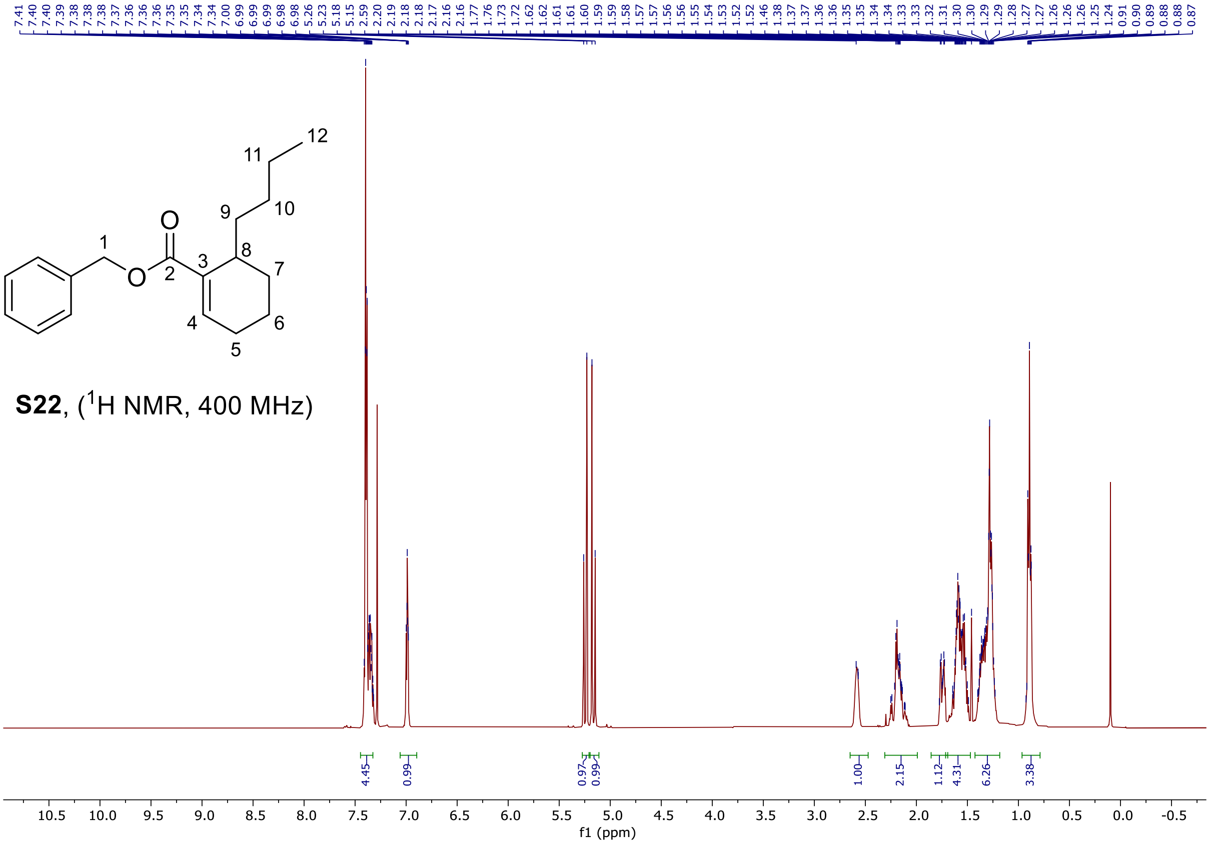
7.41 7.40 7.40 7.39 7.38 7.38 7.38 7.37 7.36 7.36 7.36 7.35 7.35 7.34 7.34 7.00 6.99 6.99 6.99 6.98 6.98 5.26 5.23 5.18 5.15 2.59 2.20 2.19 2.18 2.18 2.17 2.16 2.16 1.77 1.76 1.73 1.72 1.62 1.62 1.61 1.61 1.60 1.59 1.59 1.58 1.57 1.57 1.56 1.56 1.55 1.54 1.53 1.52 1.52 1.46 1.38 1.37 1.37 1.36 1.36 1.35 1.35 1.34 1.34 1.33 1.33 1.32 1.31 1.30 1.30 1.29 1.29 1.28 1.27 1.27 1.26 1.26 1.25 1.24 0.91 0.90 0.89 0.88 0.87
1
O
2
3
4
5
6
7
8
9
10
11
12
S22, (1H NMR, 400 MHz)
4.45
0.99
0.97
0.99
1.00
2.15
1.12
4.31
6.26
3.38
10.5 10.0 9.5 9.0 8.5 8.0 7.5 7.0 6.5 6.0 5.5 5.0 4.5 4.0 3.5 3.0 2.5 2.0 1.5 1.0 0.5 0.0 -0.5
f1 (ppm)

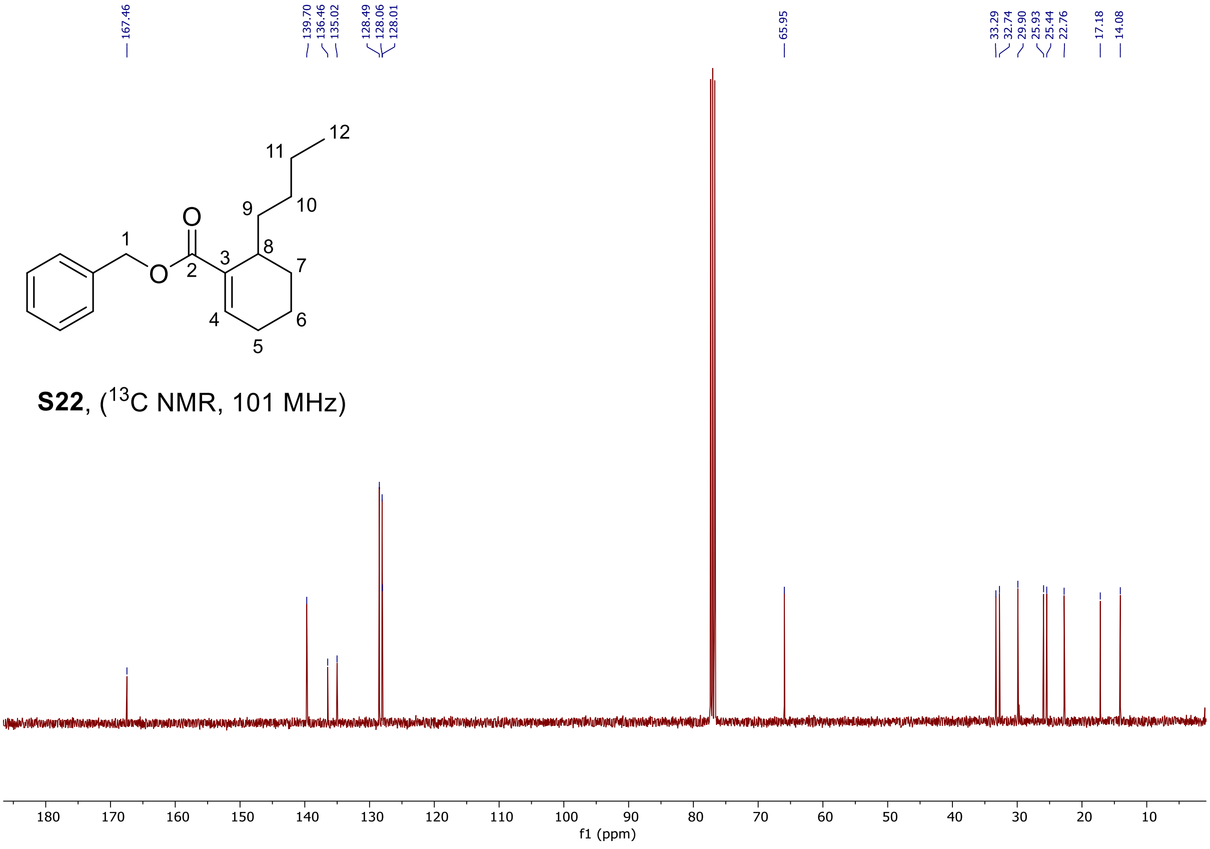
167.46
139.70 136.46 135.02
128.49 128.06 128.01
65.95
33.29 32.74 29.90 25.93 25.44 22.76
17.18
14.08
1
O
2
3
4
5
6
7
8
9
10
11
12
S22, (13C NMR, 101 MHz)
180 170 160 150 140 130 120 110 100 90 80 70 60 50 40 30 20 10
f1 (ppm)

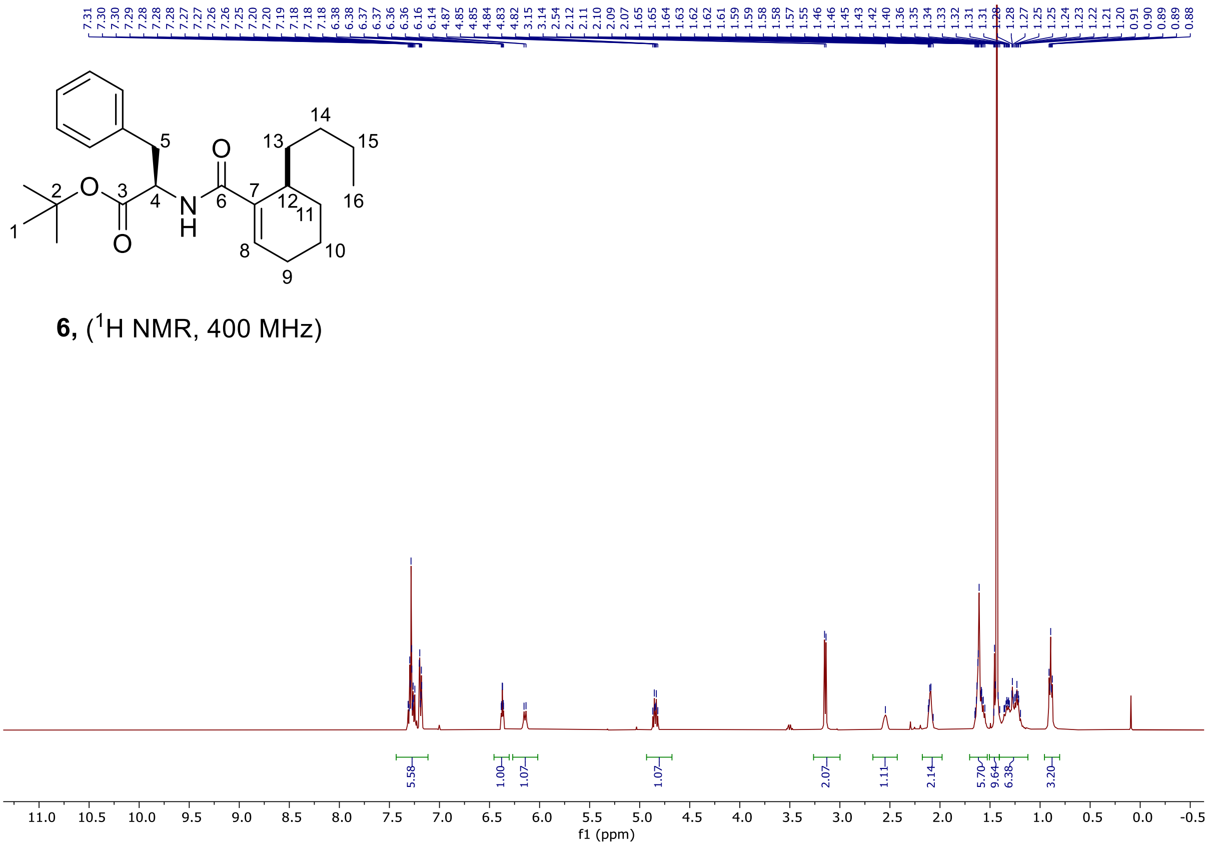

6, (^{1}H NMR, 400 MHz)

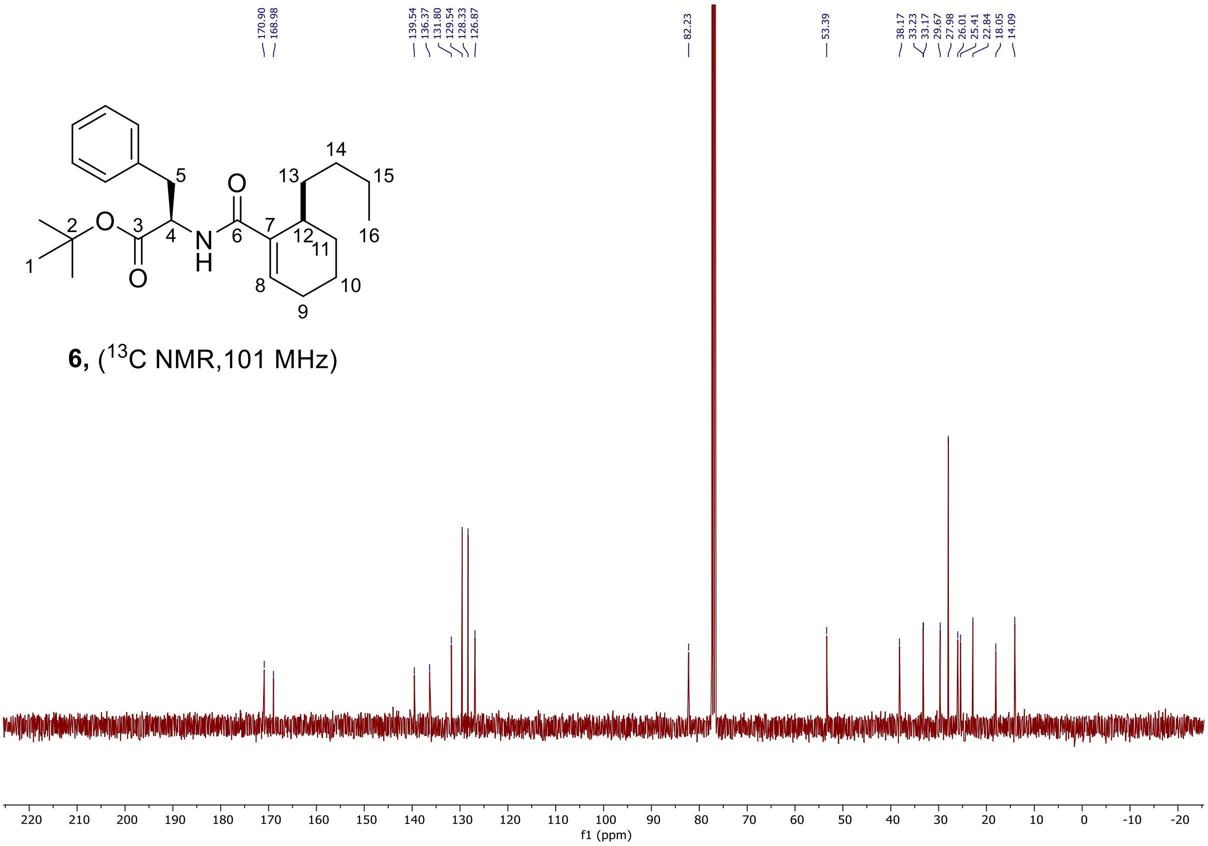

6, (^{13}C NMR,101 MHz)

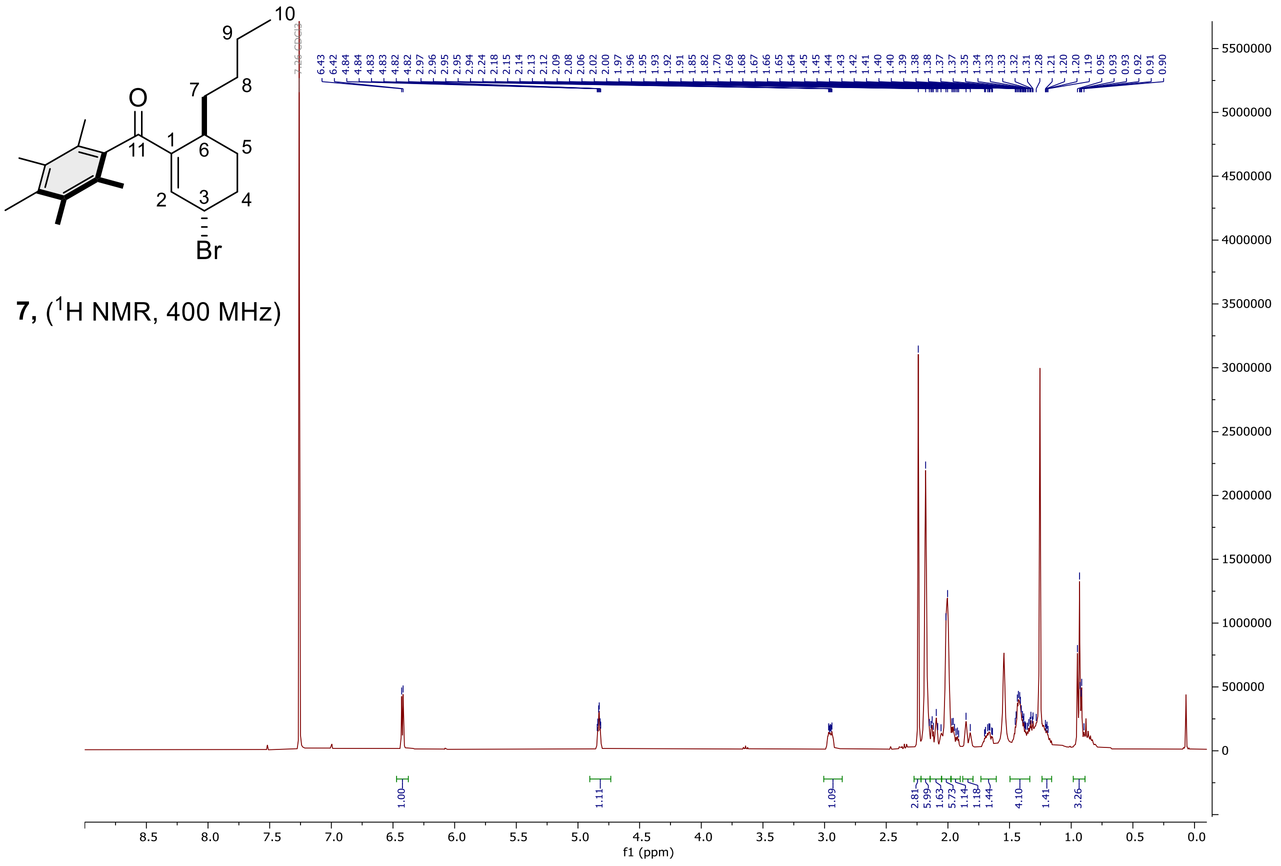

7, (¹H NMR, 400 MHz)

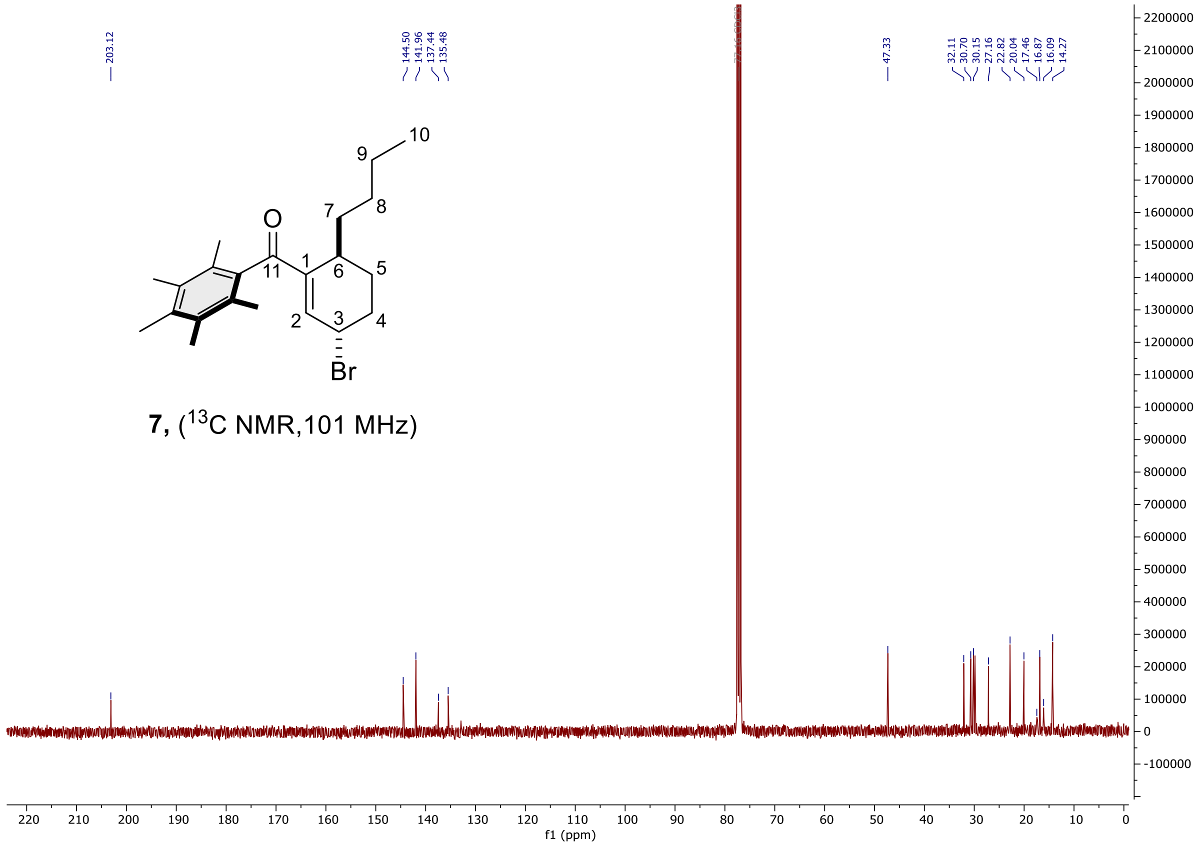

7, (¹³C NMR,101 MHz)

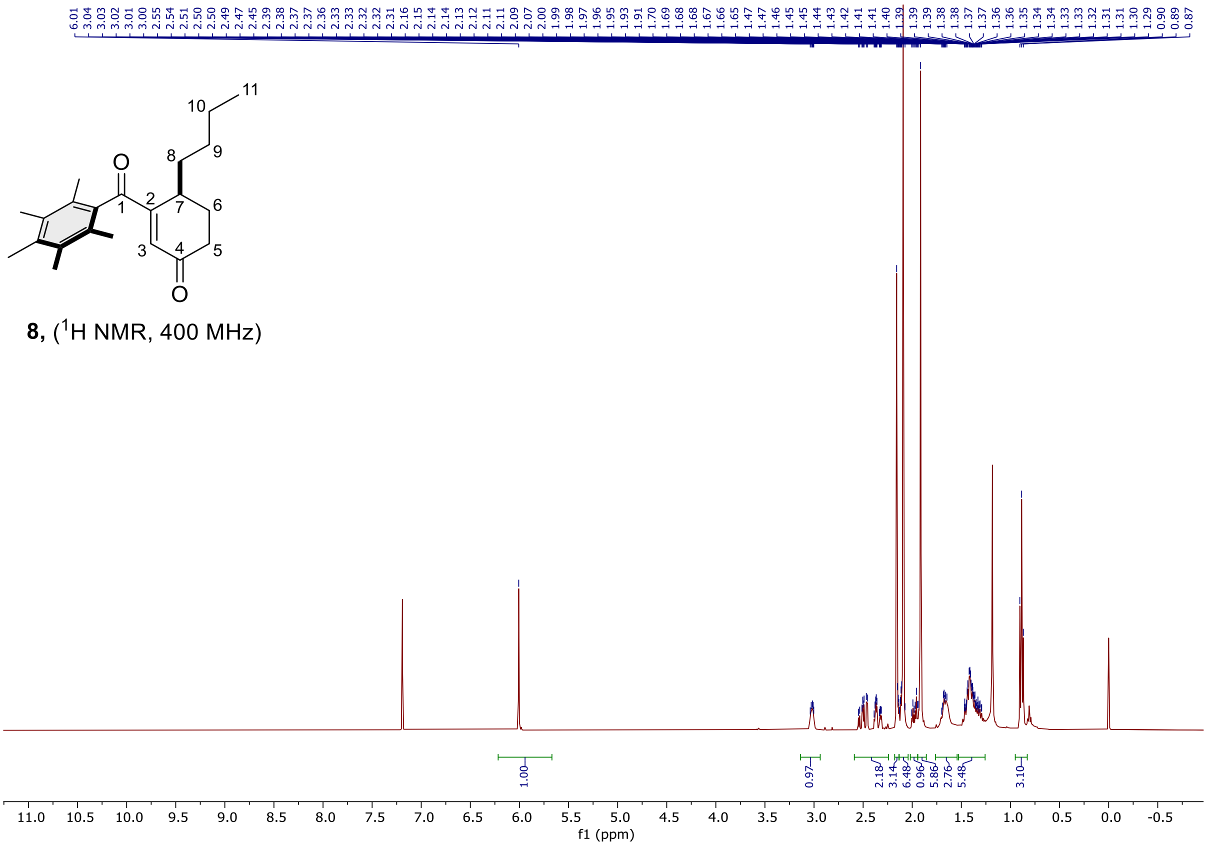
6.01 3.04 3.03 3.02 3.01 3.00 2.55 2.54 2.51 2.50 2.50 2.49 2.47 2.45 2.39 2.38 2.37 2.37 2.36 2.33 2.33 2.32 2.32 2.31 2.16 2.15 2.14 2.14 2.13 2.12 2.11 2.11 2.09 2.07 2.00 1.99 1.98 1.97 1.96 1.95 1.93 1.91 1.70 1.69 1.68 1.68 1.67 1.66 1.65 1.47 1.47 1.46 1.45 1.45 1.44 1.43 1.42 1.41 1.41 1.40 1.39 1.39 1.39 1.38 1.38 1.37 1.37 1.36 1.36 1.35 1.34 1.34 1.33 1.33 1.32 1.31 1.31 1.30 1.29 0.90 0.89 0.87
1 2 3 4 5 6 7 8 9 10 11
O
O
8, (1H NMR, 400 MHz)
1.00 0.97 2.18 3.14 6.48 0.96 5.86 2.76 5.48 3.10
11.0 10.5 10.0 9.5 9.0 8.5 8.0 7.5 7.0 6.5 6.0 5.5 5.0 4.5 4.0 3.5 3.0 2.5 2.0 1.5 1.0 0.5 0.0 -0.5
f1 (ppm)

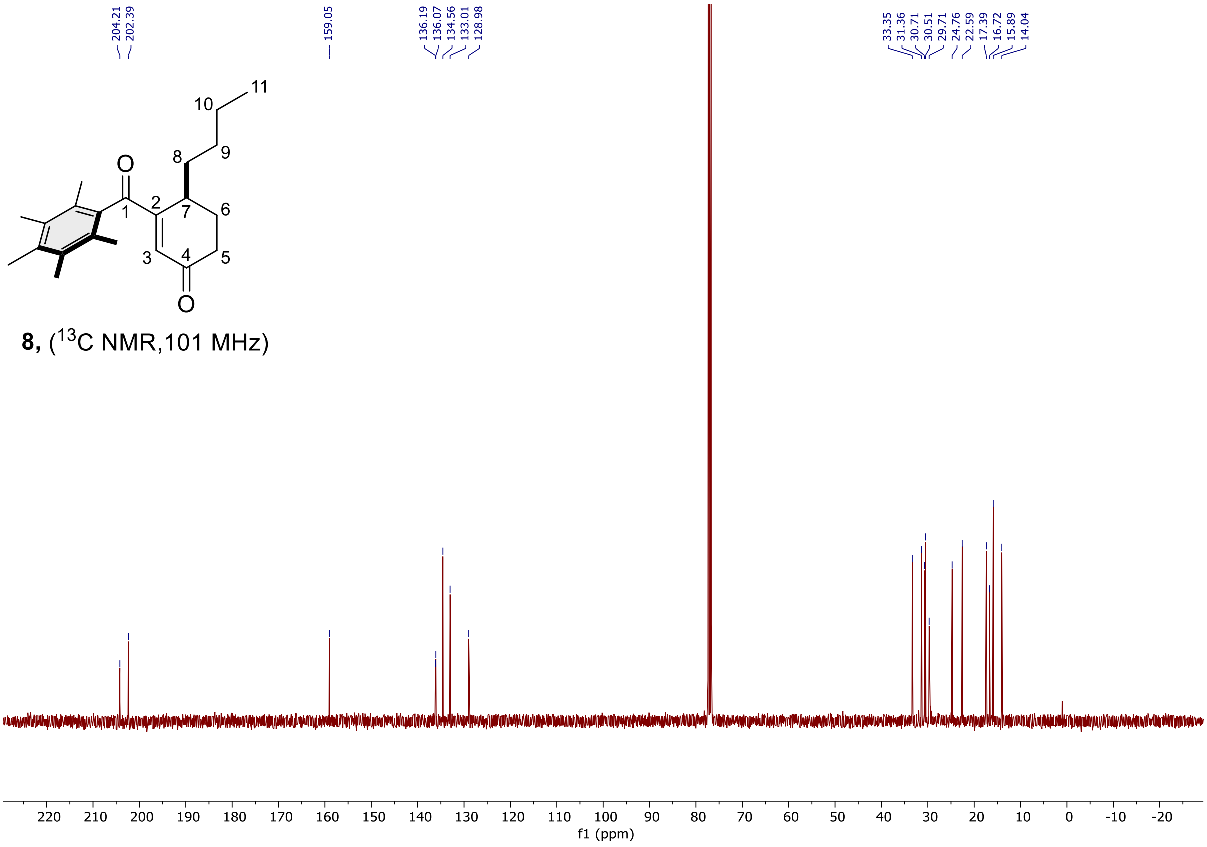
204.21 202.39 159.05 136.19 136.07 134.56 133.01 128.98 33.35 31.36 30.71 30.51 29.71 24.76 22.59 17.39 16.72 15.89 14.04
1 2 3 4 5 6 7 8 9 10 11
O
O
8, (13C NMR,101 MHz)
220 210 200 190 180 170 160 150 140 130 120 110 100 90 80 70 60 50 40 30 20 10 0 -10 -20
f1 (ppm)

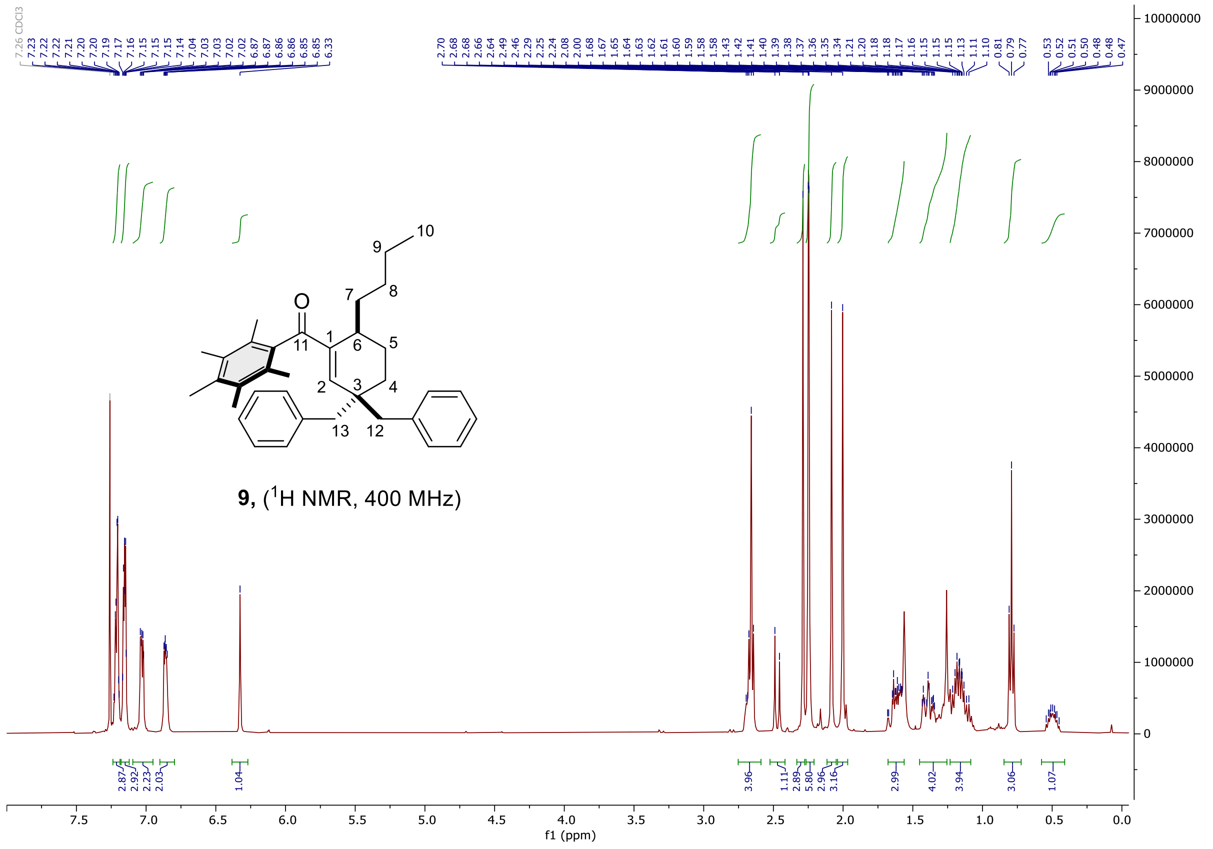

9, (^{1}H NMR, 400 MHz)

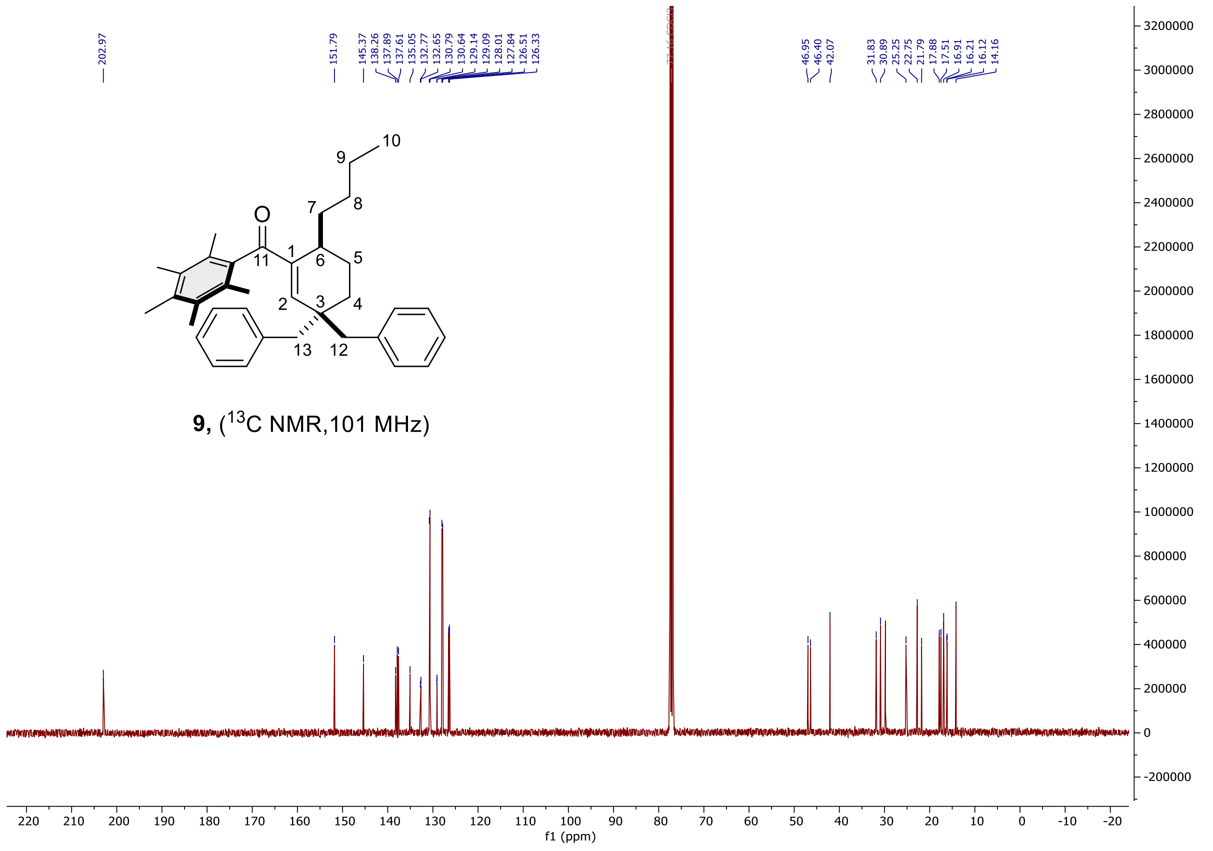

9, (^{13}C NMR, 101 MHz)
